# Genomic Basis and Climate Change Vulnerability of Migration Timing in Atlantic Salmon (*Salmo salar*)

**DOI:** 10.1111/eva.70148

**Published:** 2025-09-26

**Authors:** Samantha V. Beck, Tony Kess, Cameron M. Nugent, J. Brian Dempson, Gerald Chaput, Hallie E. Arno, Steve Duffy, Nicole Smith, Paul Bentzen, Matthew Kent, Victoria L. Pritchard, Ian R. Bradbury

**Affiliations:** ^1^ UHI Institute for Biodiversity and Freshwater Conservation UHI Inverness Inverness UK; ^2^ Fisheries and Oceans Canada Northwest Atlantic Fisheries Centre St. John's Newfoundland Canada; ^3^ Biology Department Dalhousie University Halifax Canada; ^4^ Fisheries and Oceans Canada Moncton New Brunswick Canada; ^5^ Centre for Integrative Genetics, Department of Animal and Aquacultural Sciences, Faculty of Biosciences Norwegian University of Life Sciences Ås Norway

**Keywords:** age‐at‐maturity, biodiversity, conservation, genomic offset, life history, local adaptation, phenology, run timing

## Abstract

With global environmental change, mismatches between seasonal movements of species and environmental conditions are increasingly impacting survival and persistence. Atlantic salmon ( *Salmo salar* ) perform long‐distance marine migrations culminating in a return to natal rivers, the timing of which varies among and within populations. Global declines of salmon raise the possibility that phenological mismatches could be a contributing factor; however, the underlying genetic architecture of run timing remains poorly understood. Here, we use a 220 K SNP Array to examine the association of genetic variation with run timing at a population level for 11 North American rivers. We also ask what the potential vulnerability of run timing is to future climate change by estimating trait‐specific genomic offsets, i.e., predicted shifts in allele frequencies at loci associated with run timing under projected climate change, yielding relative estimates for each population. Detected associations suggest a polygenic basis for run timing, including a large structural variant and maturation‐associated genes previously characterised in Atlantic salmon (*six6*, *vgll3*), and *ppfia2*, a migration‐timing gene conserved across vertebrates. Genomic offsets associated with climate change impacts for run timing were highest in more northern populations, suggesting potential maladaptation in future migrations. By describing the genetic architecture of run timing in North American Atlantic salmon and possible impacts of climate change on the persistence of life‐history strategies, results from this study contribute towards a better understanding of this complex life‐history trait to inform future conservation management.

## 1 Introduction

Seasonal movements of animals across large distances can have profound effects on community processes and ecosystem functions (Holdo et al. 2011), as well as contributing to ecosystem diversity, resilience, and stability (Bauer and Hoye 2014; Des Roches et al. 2018; Figge 2004). Such migratory events enable species to cope with environmental change while maintaining optimal resource availability through a complex interplay between behaviour, genetics, physiology, morphology, biomechanics, and the environment (Dingle 2006; Lohmann 2018). Local adaptation has finely tuned internal physiological processes with external environmental cues (e.g., photoperiod and temperature) so that they both reliably co‐vary with destination conditions, allowing individuals to arrive at the most optimal time. However, rapid environmental change is altering habitats in ways that are no longer predictable and may result in mismatches between migration timing and destination conditions, including prey availability, which could be a potential driver contributing to declines in migratory species (Joly et al. 2021; Visser and Gienapp 2019).

Migration phenotype in ectotherms is particularly vulnerable to the impacts of warming temperatures as their physiological processes are directly influenced by surrounding environmental conditions (Seebacher and Post 2015). Numerous studies of diadromous fishes have documented changes in migration phenology, with general patterns showing how migration has advanced in response to climate change and other anthropogenic pressures (de Eyto et al. 2022; Dempson et al. 2017; Kennedy and Crozier 2010; Kovach et al. 2013; Legrand et al. 2021; Otero et al. 2014). How and whether migration phenologies can respond not only depends upon physiological constraints, but also upon the underlying genetic architecture. For example, in several Pacific salmon species, studies have documented a single genomic region of large effect situated between two adjacent candidate genes (*greb1L* and *rock1*) that is strongly associated with early return timing in adult steelhead trout ( *Oncorhynchus mykiss* ; Hess et al. 2016; Micheletti et al. 2018; Prince et al. 2017) and Chinook salmon ( *Oncorhynchus tshawytscha* ; Koch and Narum 2020; Prince et al. 2017; Thompson et al. 2019). Evidence suggests that earlier migrators may be at a higher risk from anthropogenic changes than later migrating conspecifics (Thompson et al. 2019) but they are also suspected to have adaptive importance for coping with climate change and the advancing onset of spring in the future (Wang et al. 2021), as earlier migration can help populations synchronize with shifting environmental conditions (Clausen and Clausen 2013; Lameris et al. 2018).

Across the North Atlantic, the return time of adult anadromous Atlantic salmon to their natal rivers has advanced with the effects of climate change (Dempson et al. 2017), at the same time as many populations have declined (ICES 2024; Lehnert, Bentzen, et al. 2019; Lehnert, Kess, et al. 2019). Complex interactions between sex and age‐at‐maturity can influence the timing of adult returns, with females tending to mature at an older age than males, and older‐ and younger‐maturing individuals often showing different temporal patterns of return (Fleming 1996; Jutila et al. 2003; Miettinen et al. 2024; Mobley et al. 2021). Independent of sex and age‐at‐maturity, run timing remains variable both within and between rivers (Dempson et al. 2017; Klemetsen et al. 2003; Vähä et al. 2011) and has been shown to be heritable based on previous transplant experiments (Hansen and Jonsson 1991; Jonsson et al. 2007; Stewart et al. 2002), as well as relatively high heritability estimates of phenological traits across salmonids (0.51, albeit with low sample size: Carlson and Seamons 2008). The few studies that have investigated the genetic associations of adult run timing in Atlantic salmon (Cauwelier et al. 2018; Miettinen et al. 2024; Pritchard et al. 2018) have found *six6* to be associated with run timing to a variable extent in Atlantic, Baltic, and Barents Sea lineages of European Atlantic salmon. Miettinen et al. (2024) additionally demonstrated a strong association of *vgll3* with run timing of Baltic salmon, which appeared partly independent of its effect on age‐at‐maturity. However, the genomic basis for adult run timing in North American Atlantic salmon populations remains unidentified, impeding our understanding of the genomic architecture of run timing range‐wide, and limiting our capacity to develop a genomics‐informed conservation approach for this trait. The timing at which adults return to their natal rivers can dictate the thermal and flow regimes they encounter en route, synchronize arrival with favorable spawning‐ground conditions, and ultimately influence reproductive success, making run timing a key determinant of fitness and population resilience (Foldvik et al. 2024; Quinn et al. 2016).

This work takes advantage of long‐term monitoring data on run timing and a genomic dataset based on a 220 k SNP Array to characterize the genetic architecture of migration timing in returning North American anadromous Atlantic salmon and to assess the vulnerability of this trait to climate change. We use trait‐specific genomic offsets—which quantifies the mismatch between current and predicted allele frequencies under environmental change—to assess maladaptation potential (Bay et al. 2018; Fitzpatrick and Keller 2015; Lotterhos 2024). First, we identify loci and genomic regions associated with run timing at the population level across Atlantic Canada. Second, using the run‐timing‐associated loci identified above, we calculate trait‐specific genomic offset to identify those run timing phenotypes and populations with the highest predicted genetic mismatch under future climate change. This study directly builds on genomic work done on the sea age of returning Atlantic salmon (Ayllon et al. 2015; Barson et al. 2015; Kess et al. 2024; Sinclair‐Waters et al. 2020) and date of return (Cauwelier et al. 2018; Miettinen et al. 2024; Pritchard et al. 2018), providing a trans‐Atlantic comparison to the latter. Results from this study can contribute towards more effective conservation management of different migratory phenotypes, whilst also highlighting the utility of long‐term population‐level monitoring data in genome‐wide association (GWA) studies.

## 2 Methods

### 2.1 Study System

**FIGURE 1:**
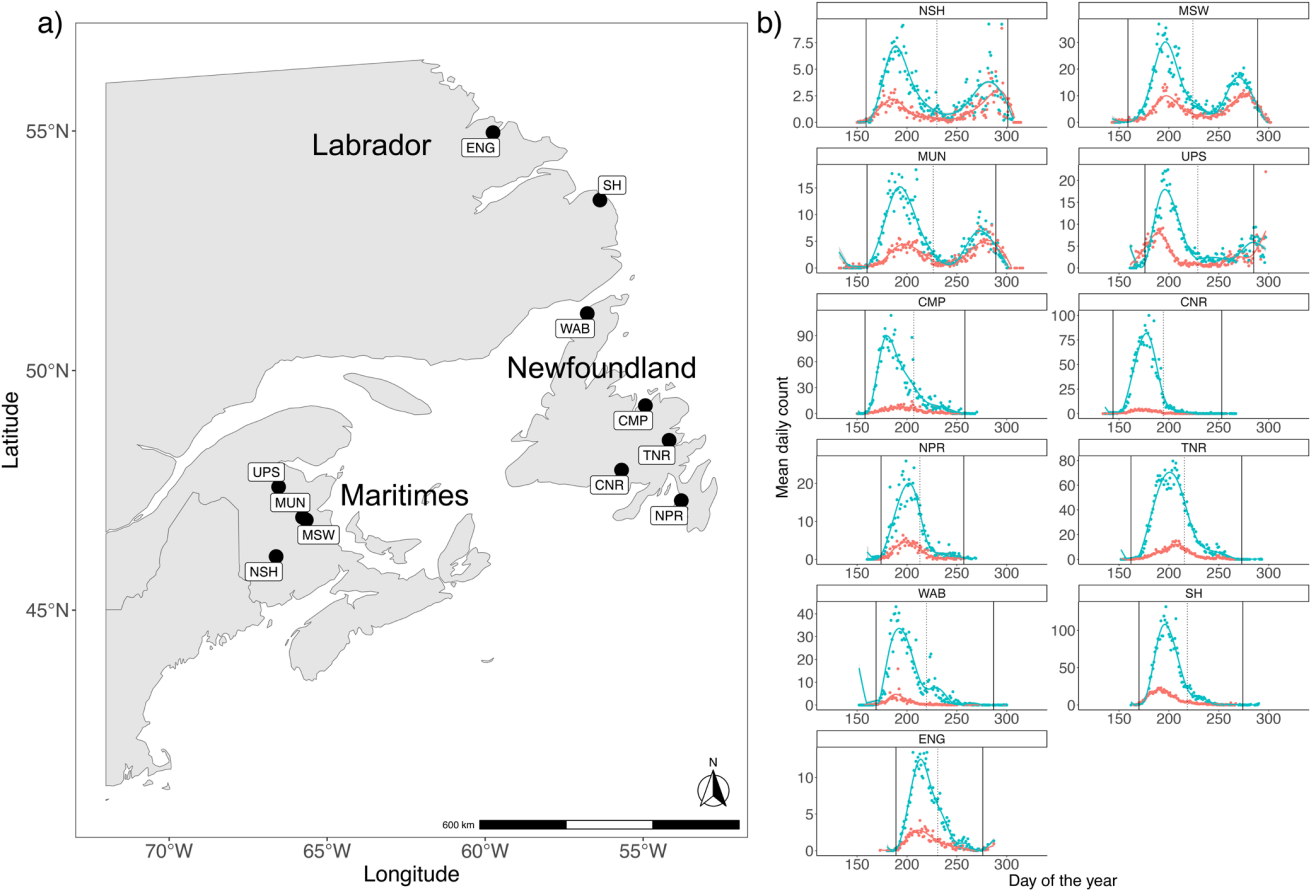
Distribution and run timing patterns of 11 North American Atlantic salmon populations: (a) Locations of 11 North American Atlantic salmon populations and (b) their respective mean daily count of adult returns across a 28‐year period (1994–2021), coloured according to size (red = > 63 cm, green = < 63 cm), with central dashed line representing mean run timing, and solid lines on either side representing the 5th and 95th percentile (calculated from a cumulative fraction and assigned to its corresponding Julian day). NSH, Nashwaak; MSW, Southwest Miramichi; MUN, Miramichi Upper Northwest; UPS, Upsalquitch; CMP, Campbellton; CNR, Conne River; NPR, Northeast Placentia River; TNR, Terra Nova River; WAB, Western Arm Brook; SH, Sand Hill; ENG, English River.

Population‐level run timing data comprised daily Atlantic salmon counts collected from fishways, counting fences, or traps in multiple years across 9 rivers and 2 tributaries in the Maritimes (*n* = 4), Newfoundland (*n* = 5) and Labrador (*n* = 2) regions (as defined by Nugent et al. 2024) of Atlantic Canada (Figure 1a; Table 1). To avoid bias due to observed global advances in salmon migration over the past decades (Dempson et al. 2017), we selected those populations with run timing data from approximately the same 28‐year period (1994–2021). The location of fish counting facilities varied by region, with those in Newfoundland and Labrador positioned < 10 km from the river mouth, while Maritime monitoring facilities were 0 to 148 km upstream from the river mouth (Table 1). Counted adult salmon were categorized into small (< 63 cm fork length) and large (≥ 63 cm fork length) size groups. In Newfoundland rivers, most salmon make their first return to freshwater after spending just one winter at sea (1SW) and the small size group mostly consists of these 1SW fish, whilst the large size group mainly comprises repeat spawning fish (Dempson et al. 2017, 2004). In contrast, for rivers in Labrador and the Maritimes, most of the large group are salmon returning to spawn for the first time after 2 or more winters at sea (2+ SW), with a fraction being repeat spawners. Run timing patterns were similar for both size classes within each population (Figure 1b), and we therefore combined small and large size classes to increase the power of our population‐level genomic associations.

**TABLE 1:** Sample table describing each trapping location, approximate distance of trap from river mouth (km), numbers (*n*) of samples genotyped using the 220 k SNP Array, number of individuals used to describe run timing and the source of where genomic data originated.

Name	Code	Region	River/Tributary	Distance from estuary (km)	Latitude	Longitude	*n* genetic	*n* run timing (years)	5th percentile	95th percentile	Genomic data source
English River	ENG	Labrador	River	0.5	54.966667	−59.75	27	4320 (23)	188 ± 0.15	257 ± 0.19	Bradbury et al. (2022)
Campbellton River	CMP	Newfoundland	River	0.5	49.268516	−54.929383	25	6039 (28)	163 ± 0.07	242 ± 0.13	Bradbury et al. (2022)
Western Arm Brook	WAB	Newfoundland	River	0.5	51.19	−56.768333	18	6786 (28)	172 ± 0.10	239 ± 0.14	Bradbury et al. (2022)
Conne River	CNR	Newfoundland	River	2	47.923333	−55.683333	30	7220 (28)	146 ± 0.10	210 ± 0.14	Lehnert, Bentzen, et al. 2019; Lehnert, Kess, et al. 2019; Bradbury et al. (2022)
Northeast Placentia River	NPR	Newfoundland	River	4	47.285	−53.793333	30	3369 (15)	178 ± 0.15	234 ± 0.21	Bradbury et al. (2022)
Sand Hill	SH	Labrador	River	6	53.559196	−56.367329	19	4738 (20)	169 ± 0.12	241 ± 0.16	Bradbury et al. (2022)
Terra Nova River	TNR	Newfoundland	River	8	48.545	−54.18	29	7327 (27)	167 ± 0.10	246 ± 0.15	Bradbury et al. (2022)
Miramichi Southwest	MSW	Maritimes	River	0	46.881667	−65.66	23	7401 (27)	159 ± 0.08	288 ± 0.06	Bradbury et al. (2022)
Upsalquitch	UPS	Maritimes	Tributary	99	47.57175	−66.5354	28	4786 (22)	180 ± 0.11	276 ± 0.17	Bradbury et al. (2022)
Nashwaak	NSH	Maritimes	Tributary	148	46.118333	−66.613333	44	5875 (24)	159 ± 0.17	283 ± 0.20	Lehnert, Bentzen, et al. 2019; Lehnert, Kess, et al. 2019; Bradbury et al. (2022)
Miramichi Upper Northwest	MUN	Maritimes	River	0	46.936667	−65.778333	24	6995 (23)	169 ± 0.26	286 ± 0.06	Bradbury et al. (2022)

*Note:* 5th Percentile (±SE), the average day, computed over a 28‐year period, when the first 5% of adult migration commences within each population. This calculation is derived from cumulative fraction analysis. 95th Percentile (±SE), the average day, computed over a 28‐year period, when the first 95% of adult migration commences within each population. This calculation is derived from cumulative fraction analysis.

Abbreviation: *n*, number of individuals.

### 2.2 Population‐Level Run Timing Descriptors

Here, we define population‐level run timing estimators and describe how each estimator was generated: (1) (per‐population and per‐year) *run timing trends* = represent the date (day of year, DOY) at which half of the total counted migrating fish had entered the river; (2) (per‐population) *early* run timing = representing the time of the start of the return migration, calculated for each population by summing the cumulative daily counts across the 28 years and taking the DOY corresponding to the 5th percentile; (3) (per‐population) *late* run timing = indicative of the end of the return migration, calculated similarly to *early* run timing but instead uses the 95th percentile to determine the day by which 95% of adults have completed migration; and (4) (per‐population) *modality* = whether a population has a 'single' or 'multiple' run timing peaks. *Early* and *late* run timing estimators, as well as *modality*, were used as predictors for associations with genotype as they capture both the timing and the duration of migration.

Boxplots were produced to visualise within‐population variation in *early* and *late* run timing across the 28‐year period. Unless otherwise stated, all statistical tests were conducted using base R (v4.3.1; R Core Team 2023). A multiple linear regression model was used to understand how *trends* in run timing (response) have changed over time (Year) for each population (Year × Site). Given the likely latitudinal gradient of run timing, we also correlate the linear model coefficients with latitude to explore how latitude might alter the changes in timing of adult returns. Significance of tests were conducted using an ANOVA (analysis of variance) from the *car* R package (v3.1.3; Fox and Weisberg 2019).

### 2.3 SNP Data

#### 2.3.1 SNP Genotyping—SNP Array

To characterise genetic variation within each river, we roughly equalised the sample size of a set of previously genotyped individuals to a total of 297 individuals from 11 populations (see above), sampled between 2009 and 2019 (Table 1; Bradbury et al. 2022; Lehnert, Bentzen, et al. 2019; Lehnert, Kess, et al. 2019). All individuals were sampled as parr, except for Western Arm Brook (WAB, Newfoundland) where adults were sampled. Details on DNA extraction, genotyping, and bioinformatic pipelines for single nucleotide polymorphism (SNP) quality control can be found in Bradbury et al. (2022). Briefly, samples were genotyped at the Center for Integrative Genetics, Norwegian University of Life Sciences, Ås, Norway (CIGENE) using an Affymetrix Axiom genotyping array developed for European Atlantic salmon (Barson et al. 2015), containing 220 K biallelic SNPs with known locations on the Atlantic salmon genome (v3.1; European Nucleotide Archive assembly: GCA_905237065.2 (https://www.ebi.ac.uk/ena/browser/view/GCA_905237065.2)). Genotype data was then filtered for high‐quality SNPs based on their clustering patterns, only retaining highly polymorphic SNPs classed as “PolyHighRes” with call rates > 0.99. Using *PLINK* (v1.9; Chang et al. 2015; Purcell et al. 2007), SNPs were then filtered for a minor allele frequency (MAF) cut‐off of 0.01, retaining a total of 141,263 SNPs for downstream analyses.

### 2.4 Population Structure

For population structure analysis only, SNPs were pruned for linkage disequilibrium using *PLINK* with parameters *‐‐indep‐pairwise 50 5 0*.*5* (i.e., 50 SNP windows, five SNP sliding windows and an *R* ^2^ threshold of 0.5), resulting in 57,611 SNPs. Population structure was then explored using principal component analysis (PCA) in R (R Core Team 2022) package *pcadapt* (v4.4.0; Privé et al. 2020), with the most suitable number of PCs inferred from examination of scree plots. The most likely number of ancestral populations was also inferred by running *snmf* (v3.14.0; Frichot et al. 2014) from the R package LEA (Frichot and François 2015) for *K* = 1–11 and examining the distribution of cross‐entropy criteria. To explore how different parts of the genome contributed to the differentiation among identified groups, we used *pcadapt* with a MAF threshold of 0.01 and applied the ‘componentwise’ option to identify variants with the highest contributions to each PC, using the non‐pruned dataset.

### 2.5 Association of Genetic Variation With Run Timing

We performed genome‐wide association (GWA) analyses using all unpruned SNPs (*n* = 141,263), adjusting for population structure, and where appropriate, also report unadjusted results for comparison. GWA analyses were conducted on population‐level run‐timing traits by assigning each fish the mean *early*‐ and *late*‐run timing values for its population, and classifying populations' *modality* based on whether they have single versus multiple timing peaks (determined from Figure 1b and coded as a binary factor). Associations with climate were also explored to inform downstream genomic offset calculations (see below).

Many studies typically overlap associations detected by multiple methods, but this approach often results in associations detected solely by the least sensitive GWA method, leading to numerous false negatives (Lotterhos 2023). To address this, we employed a two‐step approach. First, we conducted GWA using Redundancy Analysis (RDA). Secondly, to provide additional support for the associations identified by RDA, we conducted GWA using univariate latent factor mixed models (LFMM; v1.1; (Caye et al. 2019)) and highlighted those SNPs in the RDA that overlapped genes detected by both methods. This approach enhances our confidence in the identified associations by balancing the false negative and positive rates of each method, diminishing the likelihood of overlooking important SNPs due to false negatives while highlighting loci with multiple sources of evidence of association.

To conduct our GWA analyses, we performed multi‐locus associations using separate partial RDAs (pRDA) for each of the three phenotypes using the R package *vegan* (v2.6.8; Oksanen et al. 2022), conditioning the pRDA with the first three PCs characterizing population structure. Significance of the pRDA was tested using the *anova.cca* function with 999 permutations. Given that this is an exploratory analysis, and in accordance with other studies (e.g., Kess et al. 2024), associated SNPs were determined as those in the top 1% of pRDA. For LFMM, per‐SNP associations with each phenotype were conducted with population structure correction (K latent factors = 3; according to *snmf* results, see below). After applying the false discovery rate correction to account for multiple testing, associated SNPs were determined as those with *q* < 0.05. Genes overlapping candidate SNPs from both pRDA and LFMM were identified using the ‐‐closest function from bedtools (v2.30.0; Quinlan and Hall 2010), restricting overlaps to only those genes in the annotated Atlantic salmon genome (v3.1) located within 50 kb upstream or downstream of SNPs. Although the ‐‐closest function identifies the nearest genes for all SNPs, only those genes with direct SNP overlap (0 bp distance) are emphasized in the Discussion, where we focus on the top 1% (i.e., the top two genes per run timing phenotype).

To aid in understanding the biological processes that might be associated with candidate genes identified by pRDA for each run timing phenotype, we performed a Gene Ontology enrichment analysis using ShinyGO (v0.80; Ge et al. 2020). We used the closest genes (*n* = 30,898) to the complete set of filtered 220 K SNPs to reflect our sampling of the genome as our genomic background. However, it must be noted that ShinyGO can only handle a maximum of 30,000 genes. As such, to reduce the risk of false negatives, and in accordance with other studies using the same array (Nugent et al. 2024), we also used a default genomic background (Ssal_v3.1 Atlantic salmon genome). Both enrichment analyses applied the same false discovery rate (FDR) of *p* < 0.05 and searched two different databases: GO biological processes and KEGG.

To visualize patterns of linkage disequilibrium (LD) among associated SNPs, we used a heatmap of the top outlying SNP for each gene identified from associations with *early* and *late* run timing, as well as *modality*. Pairwise LD values were calculated using ‐‐*r* ^2^ square in *PLINK* and visualized using the R package superheat (Barter and Yu 2018).

### 2.6 Trait‐Specific Genomic Offset Calculations and Diversity Metrics

We carried out genomic offset analyses using the gradientForest R package (v0.1.36; Bay et al. 2018; Ellis et al. 2012; Fitzpatrick and Keller 2015) on significant run‐timing associated SNPs to estimate the predicted change in run‐timing loci under future climate conditions. To understand the extent to which the genomic basis of run timing varies with climate, we first conducted an RDA for genotype‐environment associations (GEA) using all unpruned SNPs (*n* = 1,41,263) against PC1–3 of 19 bioclimatic variables extracted for each population (see Table 1 for coordinates) from the WorldClim v2 database (2.5 arc‐minute resolution; Fick and Hijmans 2017), representing the mean present‐day climate conditions (1970–2000). All temperature‐related bioclimatic variables were included since air temperature is a well‐established proxy for water temperature (Bradbury et al. 2014; Caissie et al. 2014; Kaushal et al. 2010; Pekarova et al. 2008; Shrestha et al. 2024; van Vliet et al. 2011) and is also closely linked with changes in river flow dynamics (Pekarova et al. 2008; van Vliet et al. 2011). Because changes in flow influence the upstream migration of adult Atlantic salmon, we likewise retained all precipitation variables to capture variation in river discharge and flow regime, which directly affects migratory passage and spawning habitat. Loadings on the first (p)RDA axis for climate‐associated SNPs were correlated with loadings on the first (p)RDA axis for SNPs associated with each run‐timing phenotype (*early, late*, and *modality*), using only those loci common to both analyses. Mixed structure corrections were also conducted to explore how removing population structure from only one dataset affects the climate–run‐timing association. Genomic offsets were calculated only for those run‐timing phenotypes whose pRDA1 loadings showed a significant correlation with climate‐associated pRDA1 loadings (Pearson's correlation for parametric data and Spearman's rank correlation for non‐parametric data), using a Bonferroni‐corrected significance threshold of *p <* 0.0167 (*α* = 0.05/3, reflecting the three run‐timing phenotypes tested).

We conducted trait‐specific genomic offset analysis twice for each run timing phenotype: first using loci uncorrected for population structure, and again using loci corrected for population structure. This approach was used to avoid false negatives, as structure correction will also remove climate‐associated loci, especially when examining a trait linked to climate (Lind and Lotterhos 2024; Lotterhos 2023). Gradient forests were modeled for each run timing phenotype separately. This method utilizes a decision tree approach to identify climate predictor variables associated with changes in allele frequency. For climate data, the initial goal was to identify which of the 19 standardized present‐day bioclim variables were most important in explaining variation in run‐timing associated loci. Environmental variables are often highly correlated; therefore, those with a high correlation (> 0.7) were iteratively removed in order of their importance in explaining genomic variation at run timing loci. Subsequently, separate gradient forest models were re‐run for each run‐timing locus, fitting a gradient forest of 500 regression trees to model its allele‐frequency response to the reduced, uncorrelated set of climate predictors. Cross‐validated *R* ^2^ importance scores were then extracted to identify run timing loci with meaningful climate associations, while loci with no predictive signal were omitted from further downstream analyses (Ellis et al. 2012; Fitzpatrick and Keller 2015). Any predictor variables that did not have any importance on allele frequency turnover were removed, and forests rebuilt. These models were used as a baseline to predict expected allele frequency changes needed to cope with future climate conditions. Here, we used the “middle‐of‐the‐road” climate scenario (SSP245), based on an intermediate emissions pathway with intermediate challenges to adaptation and mitigation (Riahi et al. 2017), for the period 2061–2080. Climate data for this scenario was extracted from the CanESM5 CMIP6 climate projections from the WorldClim database with 2.5 arc‐minutes spatial resolution (Eyring et al. 2016; Meinshausen et al. 2020; Swart et al. 2019) and standardized relative to present‐day climate data across most of the distribution of Atlantic salmon in North America (Nugent et al. 2024), enabling direct comparisons through time and minimizing spatial bias. We initially performed three independent gradient forest runs and compared their top‐ranked climate predictors. If the identity of the most important variable differed between the three runs, we ran a total of 10 to determine the most important climate variable, as well as averaging genomic offsets across all runs to generate robust estimates for each population. To test whether run‐timing loci were more strongly explained by climate than the average climate‐associated SNP, we fitted a gradient forest model that included all GEA SNPs, extracted the cross‐validated *R* ^2^ for each locus, and compared the distribution of per‐SNP cross‐validated *R* ^2^ values for run‐timing loci versus all other loci using a Wilcoxon rank‐sum test (sensu Rhoné et al. 2020). Trait‐specific genomic offsets were then calculated by taking the Euclidean distance between current allele frequencies at run timing loci and predicted allele frequency turnover required to track climate projections (Fitzpatrick and Keller 2015).

To identify the extent that environmental variation is expected to change without consideration of genomic variation, we calculated range‐wide'environmental offset'. Specifically, we extracted PC1 and PC2 from the 19 present‐day bioclimatic variables and from their matching future projections across the North American Atlantic salmon climate envelope; then calculated the Euclidean distance between present and future PC scores to facilitate interpretation of our genomic offset estimates. To understand how standing levels of diversity at putative run timing loci compare to genomic offset estimates, we calculated average among‐population *F*_*ST*_, average nucleotide diversity per SNP, and observed heterozygosity for each population using hierfstat (v0.5.11; Goudet and Jombart 2022) and *PLINK*. These measures provide insights into population differentiation, genetic variation, and adaptive potential, helping to contextualize genomic offsets in relation to standing genetic variation at key loci.

## 3 Results

### 3.1 Population‐Level Run Timing Descriptors

**FIGURE 2:**
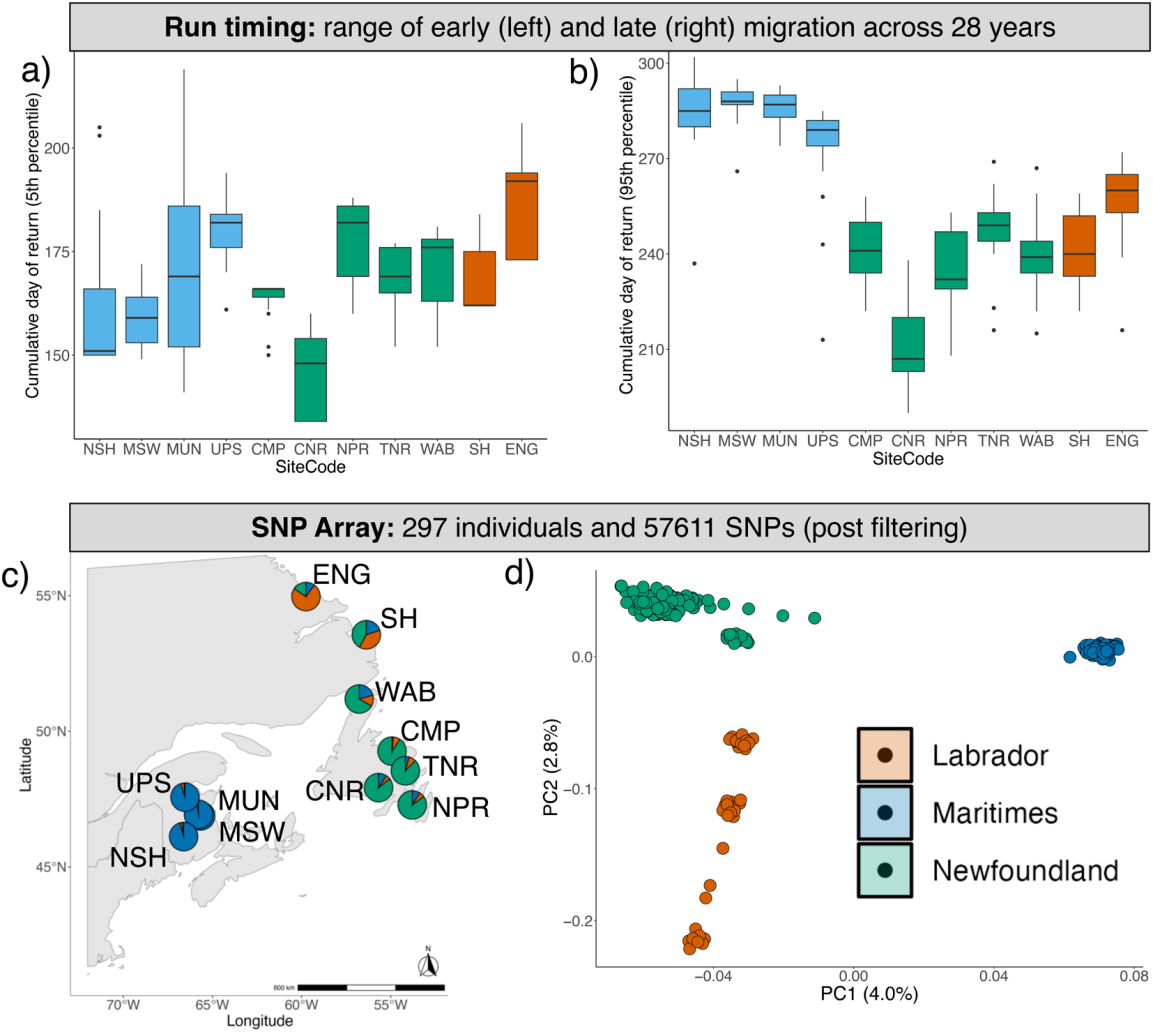
Range of (a) early (5th percentile) and (b) late (95th percentile) run timing per population of North American Atlantic salmon, calculated by summing the daily count data over a 28‐year period to create a cumulative fraction. The 5th and 95th percentiles are then determined from this cumulative fraction alongside their corresponding Julian day. Boxplots represent median and interquartile ranges of run timing. (c) Geographic distributions of populations with pie charts coloured according to the most likely ancestral cluster, identified using SNMF from a 220 k SNP Array; and (d) principal component analysis (PCA) of genetic variation showing population structure using the first two PC axes and coloured according to geographic location. NSH, Nashwaak; MSW, Southwest Miramichi; MUN, Miramichi Upper Northwest; UPS, Upsalquitch; CMP, Campbellton; CNR, Conne River; NPR, Northeast Placentia River; TNR, Terra Nova River; WAB, Western Arm Brook; SH, Sand Hill; ENG, English River.

Across the 28‐year period, populations had on average 24 years' worth of run timing data (Table 1), resulting in a total of 265 observations across 11 populations and 28 years. Conne River (CNR, Newfoundland) had the earliest run timing with 5% of the returning adults passing the count station by day 146 (average ± 0.10 standard error (SE), May 26th, Table 1; Figures 1b, 2a,b). In contrast, the English River (ENG, Labrador) population completed 5% of its return migration on average 42 days later (DOY = 188 ± 0.15 SE, July 7th). CNR was also the first population to complete 95% of its migration (DOY = 210 ± 0.14 SE; July 29th), while Southwest Miramichi (MSW, Maritimes) had the latest date for completing 95% of its migration (DOY = 288 ± 0.06 SE, October 15th). Almost all populations from the Maritimes region exhibited a bi‐modal pattern of run timing (‘*modality*’, Figure 1b); however, this was not observed in any of the other regions studied here.

Run timing *trends* within the 28‐year period varied among populations (evidenced by a significant Year * Site interaction (*F*_10,23_ = 4.10, *p* < 0.0001); Table S1; Figure S1). Pairwise comparisons found that significant multi‐annual trends in return timing were only evident for four populations, all of which have advanced their run timing: two from the Maritimes advanced their return timing by 2.4 and 1.5 days per year (Nashwaak (NSH) and Miramichi Upper Northwest (MUN), respectively) and two from Newfoundland (NPR and Terra Nova River (TNR)) advanced their return timing by 0.8 and 1 day per year (Table S1). Although not statistically significant, the only populations to return later over time were the two populations from Labrador (English River (ENG; 0.1 days per year) and Sand Hill River (SH; 0.5 days per year)) and a single population from Newfoundland (Campbellton River (CMP; 0.2 days per year)).

### 3.2 Genomic Data

#### 3.2.1 Population Structure

Analysis of population structure using PCA and ancestry analysis revealed three distinct groups (Figure S2), which mostly cluster according to geographic regions: Maritimes, Newfoundland and Labrador (Figure 2c,d). Cross‐entropy results show steep declines to *K* = 3 (Figure S2b), after which there were marginal improvements until *K* = 6; therefore, *K* = 3 was chosen as the most likely number of ancestral populations (as per Nugent et al. 2024). The first three PC axes explained 9% of genetic variation, with PC1 (which explains 4.0%) separating the Maritimes from Newfoundland and Labrador, PC2 (2.8%) separating Labrador from Maritimes and Newfoundland (Figure 2c,d) and PC3 (1.6%) mostly separating populations within Newfoundland (Figure S3). These first three PCs were used for population structure correction in downstream analyses. Differentiation between the three genetic groups varied across the genome, with highly differentiated regions including the *six6* locus on Ssa09 and a known chromosomal translocation (Ssa01/23; Figure S4). The latter primarily contributes to PC3, as indicated by the ‘componentwise’ results detailed in Table S2.

### 3.3 Genotype Associations With Population‐Level Run Timing

The top 1% of pRDA associations (1413 unique SNPs, including those within intergenic regions) overlapped 796, 715, and 812 genes for *early* (Table S3), *late* (Table S4) and *modality* (Table S5).

**FIGURE 3:**
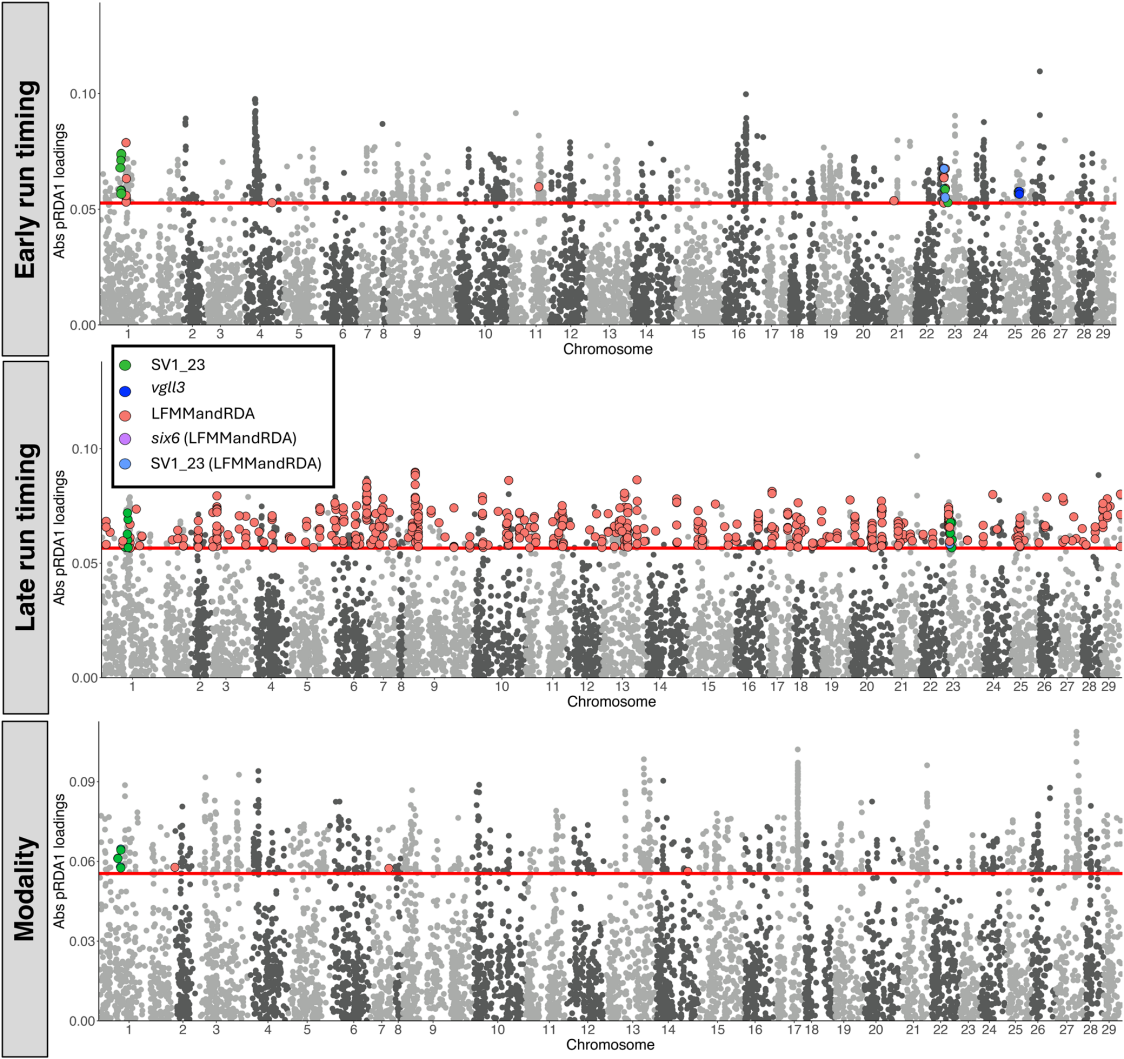
Manhattan plots showing associations with early (top) and late (middle) run timing, as well as modality (single versus multiple run timing peaks; bottom) in Atlantic salmon after correcting for population structure using a partial redundancy analysis (pRDA). SNPs above the red line represent the top 1% of SNPs based on absolute value of per‐SNP pRDA scores. SNPs coloured red are those that are also lay within genes that also overlap the univariate latent factor mixed model (LFMM; Figure S3). Where indicated in brackets (LFMMandRDA), candidate loci previously found to be potentially important in run timing were detected using both methodologies. Otherwise, candidate loci were detected by pRDA only. Those SNPs overlapping regions of interest are highlighted in a different colour, as shown in legend.

For *early* run timing, multiple peaks were present across the genome (Figure 3), with Ssa04 containing the top 1% (*n* = 2) of gene associations: *ankrd37* and *trim2a*. A total of 3 run‐timing associated SNPs from pRDA overlapped *vgll3*. Results from LFMM found large concentrations of SNPs within a chromosomal translocation spanning Ssa01 and Ssa23 (Figure S5), a region previously associated with post‐glacial secondary contact between European and North American lineages (Lehnert, Bentzen, et al. 2019; Lehnert, Kess, et al. 2019). Multiple SNPs within this karyotype‐variant region were supported by both methods (pRDA and LFMM) as being associated with *early* run timing, overlapping a total of 15 genes (Table S6). However, whilst *vgll3* was detected by pRDA, it was not detected by LFMM (Figure 3).

*Late* run timing associations were spread across the genome with the most prominent peaks on Ssa06 and Ssa09 (Figure 3) where the top 1% (*n* = 2) of gene associations were also found: *msh4* (Ssa09) and *actc1a* (Ssa06). A quarter of significant gene associations found by pRDA was also found by LFMM (*n* = 347; Figure 3; Table S6), with 23 genes within the karyotype region also detected by both methodologies (Table S6).

Peaks of associations with *modality* were evident across the genome (Figure 3), with the most prominent peak being on Ssa17, where the top 1% (*n* = 2) of gene associations were also found: *pawr* and *ppfia2*. Only three genes were detected by both pRDA and LFMM (Figure 3; Table S6).

Genes that overlapped all three run timing phenotypes were LOC106574054 (Ssa05) and LOC106577804 (Ssa18), Figure S6. Associations with *six6* (a gene previously associated with run timing) were only present before correcting for population structure in both *late* run timing and *modality* using the RDA method (Figure S7) but were present both before and after correcting for population structure when using LFMM (Figure S5). Other than *msh4* for *late* run timing, none of the other top genes were present before correcting for population structure (Table S7–S9).

Enrichment analyses using the genomic background limited to genes within the filtered 220 K array found a single KEGG pathway linked with *modality*, the carbon metabolism pathway (Table S7). Extending the genomic background to the whole genome found a total of 13 and 6 KEGG pathways linked with *early* and *late* run timing genes, whilst 2 and 18 genes linked with biological processes for *early* run timing and *modality*, respectively (Table S7).

Heatmaps of LD between the top associated SNPs for each gene found high LD in the karyotype regions of Ssa01/23 associated with *late* run timing in both the Maritimes and Newfoundland (Figure S8). We find few areas of high LD for SNPs associated with *early* run timing (Figure S8); only Ssa17 showed evidence of LD for *modality* in the Maritimes and Newfoundland.

### 3.4 Trait‐Specific Genomic Offset Estimates and Diversity Metrics

**FIGURE 4:**
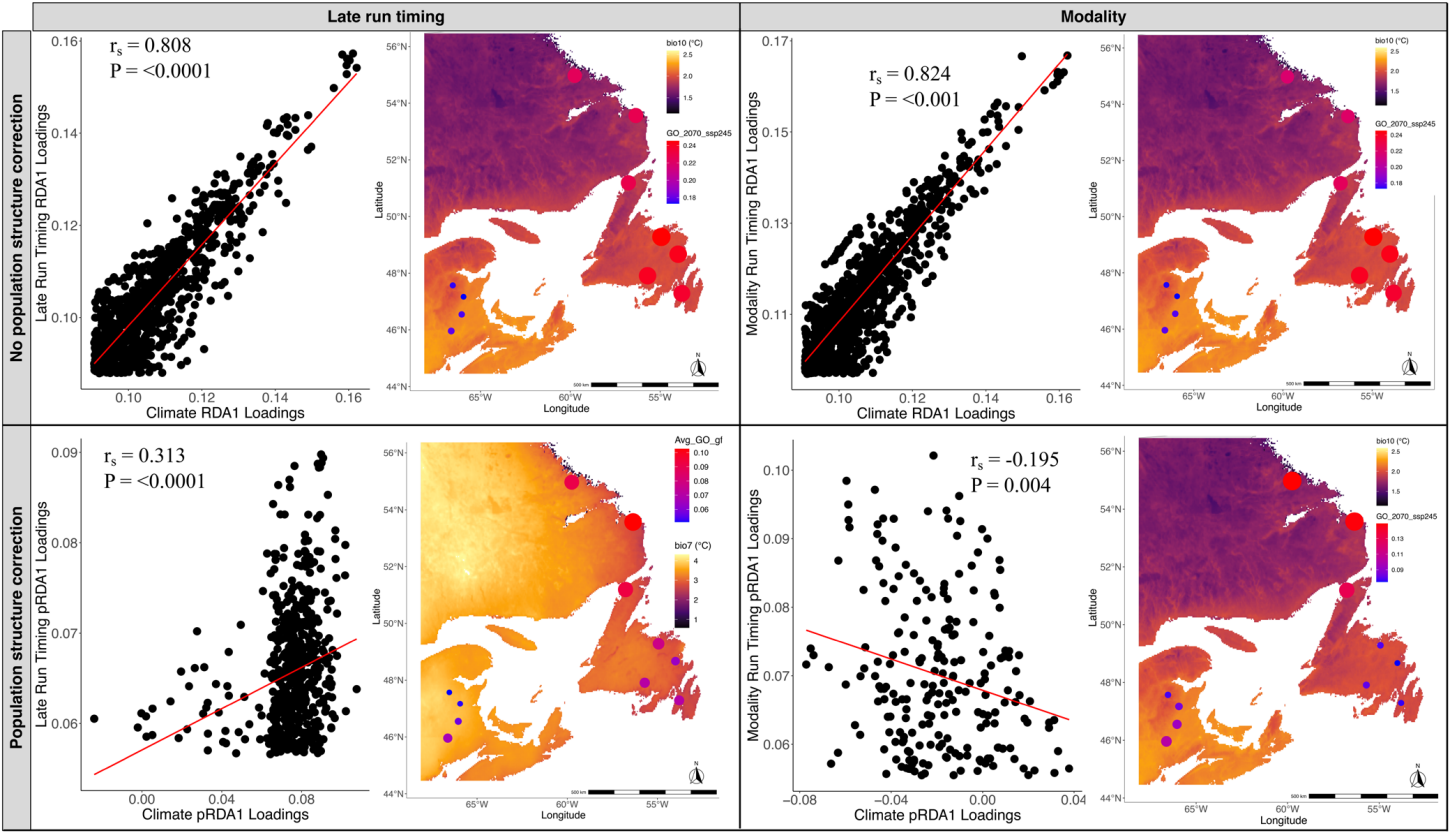
Climate‐associated run timing loci (left: *Late* run timing; right: *Modality*) and respective population‐level genomic offsets (GO) both before (top) and after (bottom) correcting for population structure. Left‐side of each individual panel shows correlation between loadings of the first RDA axes for SNPs that were associated with both climate (x‐axis) and run timing phenotype (y‐axis). Right‐side of each individual panel shows GO results for each populations' climate‐associated run timing loci. Future (2060–2080) climate projections were from the CanESM5 CMIP6 model using the “middle‐of‐the‐road” scenario (SSP245) (Eyring et al. 2016; Meinshausen et al. 2020; Swart et al. 2019), with background map based on most important climate variable as determined from gradient forest results: BIO10 (mean temperature of the wettest quarter) was the most important climate variable for all results except *late* run timing after correcting for population structure, where BIO7 (annual temperature range) was the most important climate variable (as determined from averaging results from 10 gradient forest runs). For GO, the size of the points represents the magnitude of allele frequency turner, with larger points (indicating greater change) shown in red, and smaller points (indicating lesser change) shown in blue. Spearman *r*_*s*_ and *p*‐values are given inside each panel.

Environmental offsets show that northern populations in the Northwest Atlantic are projected to experience greater discrepancies between present and future climate conditions compared to southern populations (Figure S9). Only loci associated with *late* run timing and *modality* were significantly correlated with climate‐associated loci (Figure 4), the strength of which was stronger before correcting for population structure (*r*_*s*_ = ~0.8, *p* = < 0.0001) than after (*r*_*s*_ = 0.313 and −0.195 for *late* run timing and *modality*, respectively; *p* = < 0.01). Consistent with this pattern, the direct overlap between run timing and climate SNPs also dropped after correction for population structure: of the 1413 SNPs per run timing phenotype, 1143 *late* (80.9%) and 1187 *modality* (84.0%) loci overlapped with climate SNPs in the uncorrected RDA, whereas only 741 *late* (52.4%) and 277 *modality* (19.6%) loci overlapped after population structure was accounted for. Gradient‐forest models were then trained on all 1413 loci per phenotype, where loci with a cross‐validated importance of *R* ^2^ > 0 were retained for genomic offset calculations: 1175 *late* (83.2%) and 1199 *modality* (84.9%) loci in the uncorrected data, but only 486 *late* (34.4%) and 403 *modality* (28.5%) loci after structure correction. Only these climate‐responsive loci entered the offset projections. Uncorrected run‐timing loci exhibited stronger climate predictability (mean cross‐validated *R* ^2^ = 0.47) than that of background climate loci (mean *R* ^2^ = 0.33, Wilcoxon rank‐sum test, *p* < 2 × 10^−16^). Correcting for population structure removed this difference (mean *R* ^2^ for run‐timing loci = 0.205 vs. 0.197 for climate loci; Wilcoxon rank‐sum test, *p* = 0.10). Mixed‐correction tests reinforced this pattern: when only run‐timing loci were structure‐corrected the correlation collapsed for *modality* (*r*_*s*_ = −0.04, *p* = 0.80), and was weak, albeit significant, for *late* run timing (*r*_*s*_ = −0.20, *p* = < 0.0001), whereas correcting only the climate set of loci reduced but did not eliminate the association (*late* run timing *r*_*s*_ = −0.272, *p* = 0.026; *modality r*_*s*_ = −0.457, *p* = < 0.001; Figure S10).

Multiple gradient forest runs were conducted for *late* run timing after correcting for population structure correction only due to instability between runs, where BIO16 (precipitation of the driest quarter) was the most important overall climatic variable (Figures S11, S12). In comparison, BIO10 (mean temperature of the warmest quarter) was the most important climate variable for all remaining run timing loci (Figure S11). Although correlations were stronger before correcting for population structure (Figure 4), genomic offsets appear to be strongly confounded by population structure, with the Maritimes—showing low genomic offset—being pulled out in contrast to other regions with relatively high genomic offsets across both run timing phenotypes (Figure 4). When comparing population‐structure corrected loci, genomic offsets for *late* run timing loci (average of 10 gradient forest runs, which showed little qualitative variation among individual runs; Figure S13) were highest in Labrador and northern Newfoundland, followed by southeast Newfoundland but remained low in the Maritimes region, especially for those populations in the north. For loci associated with *modality*, genomic offsets in the Maritimes, Labrador, and northern Newfoundland showed similar patterns to *late* run timing loci, whereas southern Newfoundland differed, having the lowest genomic offsets (Figure 4).

Diversity metrics were more closely aligned with genomic offsets of SNPs identified before correcting for population structure, where high genomic offsets were reflected by low heterozygosity and nucleotide diversity but high among‐population F_ST_ (Figure S14; Tables S8–S11). Correcting for population structure reduced this association (Figure S14).

## 4 Discussion

The effects of climate change and other anthropogenic pressures on migratory behaviours have raised concerns about the adaptive potential of species to cope with rapidly changing environments (Robinson et al. 2009; Tamario et al. 2019). Here, we took advantage of run timing data collected over multiple decades to examine the genomic basis of *early* and *late* run timing, as well as single versus multiple run timing peaks (i.e., *modality*), at a population level in anadromous North American Atlantic salmon, a species which is experiencing declines across its range (ICES 2024; Lehnert, Bentzen, et al. 2019; Lehnert, Kess, et al. 2019). We find that all three run timing phenotypes exhibit a polygenic basis, with peaks on multiple chromosomes. *Early* and *late* run timing had strong associations within a karyotype‐variant region that tracks trans‐Atlantic post‐glacial introgression (Ssa01/23; Lehnert, Bentzen, et al. 2019; Lehnert, Kess, et al. 2019), suggesting a potential role of genetic variation introduced from Europe to North America in adult return timing. Both *late* run timing and *modality* were indicated as having a greater risk of not being able to genetically track climate change in the north, reflecting the increased impacts of climate change in northern populations (Rantanen et al. 2022). These findings provide insights into conserved evolutionary processes and highlight how climate change might disrupt long‐evolved associations by rapidly altering environmental cues, outpacing a populations' ability to adapt in situ.

### 4.1 Polygenic Basis and Conserved Genes Underlying Migration Timing and Life History Adaptation

Many loci were associated with *early* and *late* run timing, as well as *modality*, suggesting a complex and multifaceted nature of this important life history trait. Migration has evolved as a 'syndrome' of multiple correlated responses in traits associated with physiology or early life history (Dingle 2014). Both *vgll3* and *six6* have been strongly linked with age at maturity, with the association of s*ix6* often being lost following population structure correction (Barson et al. 2015; Kess et al. 2024; Pritchard et al. 2018), suggesting the involvement of this gene correlated with a trait associated with an environmental gradient (Barson et al. 2015; Sinclair‐Waters et al. 2020), such as migration timing (Cauwelier et al. 2018; Miettinen et al. 2024; Pritchard et al. 2018). Using population‐level genomic associations, we provide further evidence for the role of *six6* in run timing (specifically *late* run timing and *modality*; Figures S5, S7), as well as the sensitivity of this association to corrections for population structure in North American Atlantic salmon (Figure 3). Additional support for a shared genetic basis between sea age at maturity and migration timing can be found in the association of *vgll3* with *early* run timing (Figure 3; Barson et al. 2015), as well as shared enriched processes such as the mapK signaling and adherens junction KEGG pathways (Table S7; Kess et al. 2024). Caution is warranted in the interpretation of enrichment analyses as they are primarily for the purpose of exploration of potential molecular function and generation of novel hypotheses about the molecular bases of traits. With this caveat in mind, these findings highlight the potential importance of shared processes associated with life history events. Life history traits require sensitivity to seasonal cues so that phenologies accurately correspond to environmental conditions. The interconnectedness of genes and external signals (e.g., photoperiod) highlights potential constraints on life history traits to evolve independently in stochastic environments (Garcia‐Costoya et al. 2023; Hansen 2003; Hughes and Leips 2017; Mauro and Ghalambor 2020; Melo et al. 2016).

Conserved process of genes involved in key life history traits is further shown by one of the top genes associated with *modality*, *ppfia2*. This gene is also associated with migration timing in a long‐distance migratory songbird, the purple martin ( *Progne subis subis* ; de Greef et al. 2023), highlighting the potential conserved nature of this gene and its importance in regulating crucial seasonal behaviors across taxa. The other top gene associated with modality was *pawr*, which has been related to behavioral issues due to its involvement in regulating dopamine (Anney et al. 2008). Differences in dopamine production have been linked to alternative behaviors, such as gregarious versus solitary phases in migratory locusts (*Locusta migratoria*; Guo et al. 2015), as well as stimulating upstream migratory behavior in chum salmon ( *Oncorhynchus keta* ; Haraguchi et al. 2015). A region of the brain that can inhibit dopamine neurons is the habenula (Metzger et al. 2021), a key modulator of motivation and body movement under learned adverse conditions, as well as sleep regulation (Hikosaka 2010). Due to its coevolution with the pineal gland, which contains photosensitive cells and regulates circadian and seasonal rhythms, Hikosaka (2010) suggests that the habenula may play an essential role in minimizing energy expenditure through the suppression of body movements (Tavakoli‐Nezhad and Schwartz 2006). In zebrafish, the habenula has also been linked to learned navigation through external cues (Cherng et al. 2020). While speculative, *pawr* association with *modality* could present a mechanism by which alternative behaviors (single versus multiple run timing peaks) have evolved through the production of dopamine and the learned responses to external environmental cues via the habenula (Loonen 2024).

The top genes associated with *early* run timing, *ankrd37* (ankyrin repeat domain 37) and *trim2a* (tripartite motif containing 2a), are both linked to oxygen availability, highlighting their potential importance in supporting the physiological demands of early migration in Atlantic salmon. For instance, *ankrd37* is induced under hypoxic conditions, with a study in zebrafish showing that its upregulation enhances protection against cold stress (Long et al. 2015). Similarly, evidence from songbirds suggests that tolerance to hypoxia increases during migration, enabling more efficient oxygen utilization under challenging environmental conditions (Ivy and Guglielmo 2023). The role of *Trim2a* in the production of red blood cells (Cannon et al. 2013) adds to the importance of mechanisms that enhance oxygen availability and stress tolerance in early migrating Atlantic salmon.

One of the top genes associated with *late* run timing was *actc1a* (Actin alpha cardiac muscle 1a) which not only plays a crucial role in cardiac function and integrity but is also involved in protection against oxidative stress (Angelini et al. 2020) and restoration of skeletal muscle function (Sztal et al. 2018). Given the combined challenges of elevated temperatures and reduced oxygen availability that late migrating Atlantic salmon experience, *actc1a* may help support physiological resilience in these demanding conditions. The other top gene associated with *late* run timing was *msh4*, a gene associated with sterility due to meiotic failure (Kneitz et al. 2000) as well as diminished ovarian reserves (Wan et al. 2023). The production of less viable gametes due to increased energy expenditure required for migrating upstream during high temperatures presents a trade‐off between allocating energy to producing gametes and fueling migration to reach spawning grounds (Fenkes et al. 2016).

### 4.2 Genomic Vulnerability of Run Timing Phenotypes to Climate Change

Global climate models predict that temperatures in the 21st century (2081–2100) will increase from 'pre‐industrial' (1850–1900) levels by 1.5°C to 4.4°C for the best and worst‐case greenhouse gas emission scenarios, respectively (Calvin et al. 2023). Predictions also suggest changes in the frequency, duration, and intensity of climate extremes (Bathiany et al. 2018; Calvin et al. 2023; Ridder et al. 2022; Seneviratne et al. 2012). As a consequence, populations will face escalating threats such as altered river temperatures (Geissinger et al. 2024; Isaak et al. 2012), shifts in precipitation patterns (Trenberth 2011), and changes in habitat availability (Platts et al. 2019; van Vliet et al. 2013). In concordance with Dempson et al. (2017), we find that four of 11 populations have shifted migration timing to return earlier, ranging from 0.8 to 2.4 days per year for Northeast Placentia River (Newfoundland) and Nashwaak (Maritimes), respectively. Northerly distributed populations had the highest trait‐specific genomic offsets for loci associated with *late* run timing and *modality* (Figure 4), likely reflecting the approximately fourfold acceleration of climate change at higher latitudes (Rantanen et al. 2022). However, because genomic offset models assume closed populations and do not take into consideration potential gene flow from more southerly populations, it may overestimate mismatch in the north, where gene flow from warmer southern rivers could introduce beneficial alleles and speed adaptive responses in northern populations (Jump and Peñuelas 2005).

Our results indicate that alleles underlying migration timing contribute disproportionately to predicted genomic offsets of North American Atlantic salmon populations, reinforcing our inference that migration timing is a key trait of climate adaptation in this species. Before correcting for population structure, run‐timing SNPs emerged as the strongest climate predictors compared with all other climate‐associated loci (mean *R* ^2^ = 0.47 vs. 0.33; Wilcoxon *p* < 2 × 10^−16^) but this difference disappears when population structure is removed (mean *R* ^2^ = 0.205 vs. 0.197; *p* = 0.10), illustrating how tightly geography, climate, and run timing allele frequencies are intertwined in Atlantic salmon (Figure 4; Figure S10).

The higher trait‐specific genomic offsets in populations in the north (Figure 4), that also have reduced run timing variability (Figure 1b), lead to questions surrounding potential response mechanisms of English River, Sand Hill River, and Western Arm Brook to cope with environmental perturbations, e.g., can run timing be altered (Waddington 1942; West‐Eberhard 2003), how sensitive are these populations to phenological cues (such as temperature; Kelly 2019), and what is the viability of range shifts and habitat availability as adaptive strategies to cope with climate change (Eckert et al. 2008)? The population with the earliest run timing studied here was Conne River (Newfoundland). The current status of salmon in this river is critical, with record low numbers recorded between 2017 and 2020 (Dempson et al. 2024; DFO 2023). Earlier run timing may expose salmon to numerous increased environmental stressors compared to later migrating individuals (Thompson et al. 2019), such as reduced growth, increased exposure to predation and pathogens, as well as increased temperatures in freshwater, altered flow regimes, and habitat availability (Quinn et al. 2016). Yet the same alleles that drive early migration may become advantageous as spring conditions continue to advance, particularly in northern rivers that likely lack the genomic variation needed to shift migration timing earlier. Populations that harbor early run timing genotypes could therefore serve as important donors of adaptive alleles for more northerly stocks as the climate warms. A global genome‐wide offset analysis, together with habitat availability and demographic connectivity, would be the next step toward a fuller assessment of population vulnerability to climate change across Atlantic Canada.

### 4.3 Evidence for the Influence of Chromosomal Rearrangements on Run Timing

Eastern and Western North Atlantic lineages of Atlantic salmon appear to have been isolated for > 600,000 years in separate glacial refugia (King et al. 2007), accumulating genetic variation and chromosomal differences (Brenna‐Hansen et al. 2012) until the end of the last glacial maximum ~18,000 ybp, when secondary contact between the two lineages occurred (Bradbury et al. 2015; King et al. 2007; Lehnert, Bentzen, et al. 2019; Lehnert, Kess, et al. 2019). A chromosomal translocation between Ssa01/23 in North American Atlantic salmon retains evidence of trans‐Atlantic introgression (Lehnert, Bentzen, et al. 2019; Lehnert, Kess, et al. 2019), with variation of this karyotype showing a strong correlation with temperature in North American Atlantic salmon (Watson et al. 2022). The potential adaptive significance of this region is highlighted here, with strong population‐level associations with *early* and *late* run timing, primarily driven by populations in Newfoundland where there are particularly high levels of European introgression (Bradbury et al. 2015). The high correlation of Ssa01/23 with temperature, its increased frequency in areas that experienced greater variability in temperature (Watson et al. 2022), and its associations with run timing suggest a potential role for this genomic region in (mal)adaptation of migratory phenotypes to temperature changes. Studies on other species suggest that introgression from divergent lineages can result in greater resilience to changes in environmental conditions and contribute to population range expansion (Gramlich et al. 2016; Rius and Darling 2014), or in our case, the ability to alter migratory behaviour as temperatures change. Alternatively, introgression may reduce fitness (Burke and Arnold 2001). Although this hypothesis may seem unlikely given the time since historical introgression occurred (~10,000 ybp), rapid climate change in recent years may have created conditions where declines in fitness become more apparent as incorporated genetic variation is exposed to natural selection. Further investigation is necessary to address this hypothesis, including assessing whether the influence of European ancestry on run timing is (mal)adaptive and the degree to which it extends beyond Newfoundland.

### 4.4 Future Directions, Limitations and Conservation Implications

Our findings illustrate how run timing is changing over time, with populations in the south migrating earlier each year than those in the north (Figure S1). Earlier migration to breeding grounds is a pattern that is occurring across species and ecosystems (Parmesan and Yohe 2003), with systematic variation in the rate of change across trophic levels, increasing the likelihood of trophic mismatches (Thackeray et al. 2010). Finely tuned migrations have evolved to maximize fitness in a seasonal environment according to resources (Alerstam et al. 2003), disruption of which has resulted in population declines in multiple species (Mayor et al. 2017; Post et al. 2008; Prince et al. 2017; Saino et al. 2011) and may be contributing to ongoing population declines in Atlantic salmon. Although plasticity can buffer the effects of environmental change through the adjustment of phenologies towards an earlier migration, the extent to which phenotypes can be altered (i.e., the elevation and slope of a trait's reaction norm) is ultimately limited by the genotype (Hutchings 2011; Lande 2009). How Newfoundland salmon populations respond to future environmental change is of particular interest given: (1) the increased signals of European ancestry found in this region (Bradbury et al. 2015; Lehnert, Bentzen, et al. 2019; Lehnert, Kess, et al. 2019); (2) the retention of these signatures of secondary contact in Ssa01/23 (Lehnert, Bentzen, et al. 2019; Lehnert, Kess, et al. 2019); (3) the correlation of Ssa01/23 with temperature (Watson et al. 2022); (4) elevated levels of contemporary European aquaculture introgression in southeast Newfoundland (Bradbury et al. 2022); and (5) our findings associating these chromosomal regions with run timing (Figure 2).

Obtaining accurate migratory data for genome‐wide associations can be challenging for a variety of reasons, including (but not limited to): (1) timing of freshwater entry is often not known for each breeding population (Quinn et al. 2016), only known for the entire river basin (e.g., from fishery data) or collected from downstream counting sites (e.g., the population‐level data used here); (2) variation in trap efficiency between years; (3) anomalous climate events that could influence information on run timing (e.g., severe drought and high water temperatures); and (4) river obstructions delaying upstream migration. Whilst we filtered our data to try to reduce these sources of variation, we acknowledge that interpretation of our results must be considered with these caveats in mind. Despite their biases, historical counter data provide a valuable data source for understanding how populations are changing over time (e.g., Crozier et al. 2011).

Whilst we find a polygenic basis to run timing associations, it is important to note that any oligogenic signal could have been missed given the limited coverage of the SNP array and the constraints of using population‐level data. These limitations, combined with the challenges of correcting for environmentally correlated population structure in traits under environmental selection, can contribute to inaccuracies in genomic offset estimates. For example, Lotterhos (2023) found that correcting for population structure disrupted multivariate mapping of genotype to environment, reducing the accuracy of assigning individuals to their original environment. Nevertheless, not correcting for population structure when run timing varied between regions hindered our ability to determine whether observed patterns of vulnerability were genuinely linked to migration timing or simply reflected underlying population structure. Forecasting ecological responses under novel climates, particularly for range‐edge populations and in topographically uniform landscapes, presents serious challenges that warrant caution when interpreting genomic‐offset estimates under scenarios with a high likelihood of novel climates (Lachmuth et al. 2023; Lind and Lotterhos 2025; Mahony et al. 2017). Finally, when considering genomic offset estimates, it is important to note that a high genomic offset does not necessarily mean the most vulnerable and reflects only the observed change of allele frequencies in a function describing the observed relationship between environment and genotype, further highlighting the need for experimental validation of genomic offset estimates, as well as the inclusion of diversity metrics.

## 5 Summary

These findings offer a trans‐Atlantic comparison to previous genomic studies on Atlantic salmon run timing in Europe. Our results imply a polygenic basis for run timing, including associations with genes previously linked to migration timing in birds (de Greef et al. 2023), age‐at‐maturity (Barson et al. 2015; Kess et al. 2024) and run timing in European populations (Cauwelier et al. 2018; Miettinen et al. 2024; Pritchard et al. 2018). Genomic offsets for loci associated with *late* run timing and *modality* demonstrated that populations located farther north require greater genetic turnover to adapt to anticipated climate change. However, understanding species responses to climate change requires a multifaceted approach, taking into consideration phenotypic plasticity, habitat availability, competition, dispersal ability, and evolutionary potential, as well as their interactions. This study provides a baseline to further explore how important life‐history traits that are tightly entwined with the environment can influence species vulnerability to climate change. These results can contribute towards more informed conservation and management to try and preserve the diversity of life histories that promote resilience and stability of populations to rapid environmental change (Figge 2004).

## Conflicts of Interest

The authors declare no conflicts of interest.

## Supporting information


**FIGURES S1–S14:** eva70148‐sup‐0001‐FigureS1‐S14.docx.

**TABLES S1–S11:** eva70148‐sup‐0001‐TableS1‐S11.pdf.

## Data Availability

Data used in these analyses were generated by Lehnert, Bentzen, et al. (2019); Lehnert, Kess, et al. (2019) and Bradbury et al. (2022) and are publicly available at: https://doi.org/10.5061/dryad.hdr7sqvfc. Run timing data from this study are publicly available on Dryad at DOI: https://doi.org/10.5061/dryad.p5hqbzm1h.
