# Supplementary material for: Genomic Basis and Climate Change Vulnerability of Migration Timing in Atlantic Salmon (Salmo salar)
Source: Evol Appl. 2025 Sep 26;18(10):e70148. doi: 10.1111/eva.70148 (PMC12474562; doi:10.1111/eva.70148)
Supplement: Supplementary file 1 — FIGURES S1–S14: eva70148‐sup‐0001‐FigureS1‐S14.docx. [file EVA-18-e70148-s002.docx]

**Figure S1** Annual weighted day of return for each of 11 North American Atlantic salmon populations over the 28-year period (1993–2021). Each point shows the day of year at which the cumulative daily count for that population and year reaches its mean (i.e. the “centre of mass” of the run, weighted by daily counts), colored by region. (NSH, Nashwaak; MSW, Southwest Miramichi; MUN, Miramichi Upper Northwest; UPS, Upsalquitch; CMP, Campbellton; CNR, Conne River; NPR, Northeast Placentia River; TNR, Terra Nova River; WAB, Western Arm Brook; SH, Sand Hill; ENG, English River).

**Figure S2** Scree plots of North American Atlantic salmon genetic variation to determine most likely number of (a) populations using eigenvalues from a principal component analysis, and (b) ancestral populations using change in cross-entropy values from the Sparse Non-Negative Matrix Factorization (snmf) algorithm (Frichot et al., 2014).

**Figure S3** Principal component analysis (PCA) of genetic variation showing population structure associated with a) the first and third PC axis, and b) the second and third PC axis for 11 populations of Atlantic salmon in North America.

**Figure S4** Manhattan plot of *pcadapt* results identifying three genetic clusters defined by principal-component analysis. Those SNPs overlapping regions of interest are highlighted in a different colour: *six6* = red, and chromosomal translocation Ssa01/23 = turquoise.

**Figure S5** Genome-wide associations with run timing identified using the univariate latent factor mixed model (LFMM) method for K = 1 (left) and K = 3 (right). Manhattan plots show associations with early (top) and late (middle) run timing, as well as assocations with modality (single versus multiple run timing peaks; bottom). Red line prepresents genome-wide significance and previously identified candidate genes considered to be important for run timing are highlighted.

**Figure S6** Overlap of genes associated with three run timing phenotypes in North American Atlantic salmon: early, late and modality (i.e. single versus multiple run timing peaks).

**Figure S7** Manhattan plots showing associations with early (top) and late (middle) run timing, as well as modality (single versus multiple run timing peaks; bottom) in Atlantic salmon before correcting for population structure, using a redundancy analysis (RDA). SNPs above the red line represent the top 1% of SNPs based on absolute value of per-SNP RDA scores. SNPs coloured red are those that are also lay within genes that also overlap the univariate latent factor mixed model (LFMM; Appendix 2 Fig. S2). Where indicated in brackets (LFMMandRDA), candidate loci previously found to be potentially important in run timing were detected using both methodologies. Otherwise, candidate loci were detected by RDA only. Those SNPs overlapping regions of interest are highlighted in a different colour, as shown in legend.

**Figure S8** Heat map of linkage disequilibrium (LD) between the top outlying SNPs of each gene for each genetic cluster, identified from genome-wide associations using partial RDA (correcting for population structure) with *early* (left) and *late* (middle) run timing, as well *modality* (right) in 11 populations of North American Atlantic salmon.

**Figure S9** Extent of climate change differences for each population between present climate (1970-2000) and future climate in 2070 (using CanESM5 CMIP6 climate projections using the “middle-of-the-road” socio-economic scenario (SSP245). a) a PCA was first conducted on range-wide North American Atlantic salmon present and future climate data. Populations with run timing data were extracted and distances between present and future climates shown by dashed lines and points coloured according to geographic location. b) Environmental offsets were calculated using the Euclidean distance between present and future climate data (scaled relative to the distribution of Atlantic salmon in Canada), as described using PC axes 1 and 2, with size and colour of circles representing extend of climatic differences between present and future climate (Eyring et al., 2016; Meinshausen et al., 2020; Swart et al., 2019). (NSH, Nashwaak; MSW, Southwest Miramichi; MUN, Miramichi Upper Northwest; UPS, Upsalquitch; CMP, Campbellton; CNR, Conne River; NPR, Northeast Placentia River; TNR, Terra Nova River; WAB, Western Arm Brook; SH, Sand Hill; ENG, English River).

**Figure S10** Correlation between loadings on the first RDA axis (pRDA1) for SNPs associated with climate (x-axis) and with the run-timing phenotype (y-axis). Panels show the two mixed-correction cases: the top row plots climate loci uncorrected for population structure against run-timing loci corrected for structure (left = *late* run timing, right = *modality*), whereas the bottom row plots climate loci corrected for structure against run-timing loci uncorrected for structure. Spearman r_s_ and P-values are given inside each panel.

**Figure S11** The most important climate variables that best descibe allele frequency turnover for loci associated with each run timing phenotype: *late* run timing (left) and modality (right) both with (bottom) and without (top) corrections for populations structure, in response to climate change in 2061-2080 using the “middle-of-the-road” socio-economic scenario (SSP245) taken from CanESM5 CMIP6 climate projections (Eyring et al., 2016; Meinshausen et al., 2020; Swart et al., 2019), using uncorrelated climate variables. Variable importance for l*ate* run timing before correcting for population structure is taken from the average of x10 gradient forest runs (see Fig. S12) due to instability between each run.

**Figure S12** Variability between x10 gradient forest runs and the most important environmental variables that best descibe allele frequency turnover for late run timing loci (corrected for population structure) in response to climate change in 2061-2080 using the “middle-of-the-road” socio-economic scenario (SSP245) taken from CanESM5 CMIP6 climate projections (Eyring et al., 2016; Meinshausen et al., 2020; Swart et al., 2019), using uncorrelated climate variables.

**Figure S13** Variability between x10 gradient forest runs and the most important environmental variables that best descibe allele frequency turnover for late run timing loci (corrected for population structure; Fig. S12). Genomic offsets were calculated for each gradient forest run, documenting extent of allele frequency turnover in response to climate change in 2070 using the worst case socio-economic scenario (SSP245) taken from CanESM5 CMIP6 climate projections for the year 2070 (Eyring et al., 2016; Meinshausen et al., 2020; Swart et al., 2019), using uncorrelated climate variables.

**Figure S14** Diversity metrics, environmental- and genomic- offsets for each of the 11 populations of North American Atlantic salmon (NSH, Nashwaak; MSW, Southwest Miramichi; MUN, Miramichi Upper Northwest; UPS, Upsalquitch; CMP, Campbellton; CNR, Conne River; NPR, Northeast Placentia River; TNR, Terra Nova River; WAB, Western Arm Brook; SH, Sand Hill; ENG, English River) based on loci associated with climate-linked run timing phenotypes: late run timing (left) and modality (right; single versus multiple run timing peaks). Results are shown both before (top) and after (bottom) correcting for population structure. All metrics have been scaled between 0 and 1 for comparisons across populations.

**References**

Eyring, V., Bony, S., Meehl, G. A., Senior, C. A., Stevens, B., Stouffer, R. J., & Taylor, K. E. (2016). Overview of the coupled model intercomparison project phase 6 (CMIP6) experimental design and organization. *Geoscientific Model Development*, *9*(5), 1937–1958. https://doi.org/10.5194/gmd-9-1937-2016

Frichot, E., Mathieu, F., Trouillon, T., Bouchard, G., & François, O. (2014). Fast and Efficient estimation of individual ancestry coefficients. *Genetics*, *196*(4), 973–983. https://doi.org/10.1534/genetics.113.160572

Lehnert, S. J., Kess, T., Bentzen, P., Barson, N., Kent, M. P., Lien, S., Dempson, J. B., & Bradbury, I. R. (in prep.). Large haplotypes linked to climate and life history variation in divergent lineages of Atlantic salmon (*Salmo salar*).

Meinshausen, M., Nicholls, Z. R. J., Lewis, J., Gidden, M. J., Vogel, E., Freund, M., Beyerle, U., Gessner, C., Nauels, A., Bauer, N., Canadell, J. G., Daniel, J. S., John, A., Krummel, P. B., Luderer, G., Meinshausen, N., Montzka, S. A., Rayner, P. J., Reimann, S., … Wang, R. H. J. (2020). The shared socio-economic pathway (SSP) greenhouse gas concentrations and their extensions to 2500. *Geoscientific Model Development*, *13*(8), 3571–3605. https://doi.org/10.5194/gmd-13-3571-2020

Swart, N. C., Cole, J. N. S., Kharin, V. V., Lazare, M., Scinocca, J. F., Gillett, N. P., Anstey, J., Arora, V., Christian, J. R., Jiao, Y., Lee, W. G., Majaess, F., Saenko, O. A., Seiler, C., Seinen, C., Shao, A., Solheim, L., von Salzen, K., Yang, D., … Sigmond, M. (2019). *CCCma CanESM5 model output prepared for CMIP6 ScenarioMIP* [Dataset]. Earth System Grid Federation. https://doi.org/10.22033/ESGF/CMIP6.1317
