# Supplementary material for: Genomic Basis and Climate Change Vulnerability of Migration Timing in Atlantic Salmon (Salmo salar)
Source: Evol Appl. 2025 Sep 26;18(10):e70148. doi: 10.1111/eva.70148 (PMC12474562; doi:10.1111/eva.70148)
Supplement: Supplementary file 2 — TABLES S1–S11: eva70148‐sup‐0001‐TableS1‐S11.pdf. [file EVA-18-e70148-s001.pdf]

Table S1 a) Linear model testing how Atlantic salmon return dates (based on mean cumulative counts per year) has changed across a 28 year period, and b) pairwise comparisons.

a)

	SS	Df	F	P
**(Intercept)**	8787	1	41 40	<0.0001
**Year**	7924	1	37 34	<0.0001
**Site**	8878	10	4 18	<0.0001
**Year * Site**	8695	10	4 10	<0.0001
**Residuals**	51573	243		

b)

SiteCode	Year.trend	SE	df	lower.CL	upper.CL	t.ratio	p.value
**NSH**	**-2 356**	**0 386**	**243**	**-3 116**	**-1 5967**	**-6 111**	**<.0001**
MSW	-0 573	0 357	243	-1 277	0 1298	-1 606	0 1095
**MUN**	**-1 527**	**0 453**	**243**	**-2 419**	**-0 635**	**-3 372**	**0 0009**
UPS	-0 116	0 478	243	-1 058	0 8268	-0 241	0 8094
CMP	0 159	0 341	243	-0 512	0 8306	0 467	0 6407
CNR	-0 433	0 341	243	-1 105	0 2379	-1 272	0 2046
**NPR**	**-0 751**	**0 361**	**243**	**-1 462**	**-0 0393**	**-2 079**	**0 0387**
**TNR**	**-0 954**	**0 346**	**243**	**-1 636**	**-0 2712**	**-2 753**	**0 0064**
WAB	-0 306	0 341	243	-0 978	0 3651	-0 899	0 3698
SH	0 501	0 464	243	-0 414	1 4163	1 079	0 2814
ENG	0 084	0 458	243	-0 818	0 9861	0 183	0 8546

Table S2 Most significant 500 results from 'componentwise' analysis using 11 North American Atlantic salmon. Results identify variants with the highest contributions to each PC describing genomic differentiation between the three genetic clusters, using the non-pruned dataset.

CHROM	BP	SNP	PC1_pvalues	PC1_qvalues	PC2_pvalues	PC2_qvalues	PC3_pvalues	PC3_qvalues	PC1_loading	PC2_loading	PC3_loading
12	23263037	AX-87471906	0 0000017	0 00010	0 29935	0 83553	0 92149	1 00000	0 00598	0 00233	-0 00041
25	33270913	AX-87151551	0 0000017	0 00010	0 27289	0 81796	0 62531	1 00000	0 00590	0 00243	-0 00202
5	68961687	AX-87656801	0 0000017	0 00010	0 75745	0 87480	0 87827	1 00000	-0 00596	-0 00069	-0 00064
12	74848402	AX-87170375	0 0000017	0 00010	0 04720	0 38686	0 89792	1 00000	0 00575	-0 00428	0 00052
21	37717141	AX-87168045	0 0000017	0 00010	0 81762	0 87480	0 86338	1 00000	0 00811	0 00069	0 00031
6	33425128	AX-87530090	0 0000017	0 00010	0 41619	0 87480	0 49359	1 00000	0 00597	-0 00182	-0 00287
18	52098888	AX-87572325	0 0000017	0 00010	0 18765	0 73308	0 05024	0 85718	-0 00945	-0 00450	0 00418
3	19757897	AX-87498055	0 0000017	0 00010	0 40614	0 87480	0 83722	1 00000	-0 00575	0 00179	0 00083
21	37846231	AX-87203479	0 0000017	0 00010	0 67700	0 87480	0 73478	1 00000	0 00789	0 00121	0 00060
5	7084854	AX-87124110	0 0000017	0 00010	0 17953	0 72234	0 94145	1 00000	0 00577	-0 00291	-0 00030
15	28749693	AX-87832561	0 0000017	0 00011	0 54240	0 87480	0 65645	1 00000	-0 00571	-0 00131	0 00178
19	16926766	AX-87345646	0 0000017	0 00011	0 54508	0 87480	0 33060	1 00000	-0 00831	-0 00185	0 00182
25	17594276	AX-87447146	0 0000017	0 00011	0 05523	0 42073	0 06949	0 98317	0 00795	-0 00560	-0 00325
22	8868529	AX-86972139	0 0000017	0 00011	0 35338	0 86401	0 74835	1 00000	-0 00563	0 00196	0 00127
13	103296629	AX-87418222	0 0000017	0 00011	0 00562	0 10196	0 73678	1 00000	0 00530	0 00551	0 00125
1	77495376	AX-87072650	0 0000017	0 00011	0 63657	0 87480	0 78642	1 00000	-0 00627	-0 00112	-0 00120
25	26547883	AX-87083202	0 0000018	0 00011	0 74309	0 87480	0 59739	1 00000	-0 00566	-0 00070	0 00210
25	35314779	AX-86960095	0 0000018	0 00011	0 23062	0 78207	0 43990	1 00000	0 00594	-0 00268	-0 00322
19	19243284	AX-87122725	0 0000018	0 00011	0 11358	0 60193	0 79586	1 00000	0 00585	0 00348	-0 00106
22	34799091	AX-87489037	0 0000018	0 00011	0 06892	0 47307	0 91477	1 00000	0 00572	-0 00391	0 00043
15	47477236	AX-87341393	0 0000018	0 00011	0 44805	0 87480	0 81925	1 00000	0 00578	0 00165	0 00093
20	15758506	AX-87606527	0 0000018	0 00011	0 40492	0 87480	0 03542	0 72789	-0 00833	-0 00255	0 00394
12	75220620	AX-86952369	0 0000018	0 00011	0 45463	0 87480	0 70432	1 00000	0 00622	0 00176	-0 00167
6	56686441	AX-87399628	0 0000018	0 00011	0 28845	0 82988	0 41279	1 00000	-0 00820	-0 00320	0 00151

20	44199867	AX-87609393	0 0000018	0 00011	0 07318	0 48745	0 18845	1 00000	0 00584	-0 00394	-0 00540
15	46785739	AX-87630177	0 0000018	0 00011	0 94053	0 87789	0 26256	1 00000	0 00571	-0 00016	-0 00450
21	37747027	AX-87279505	0 0000018	0 00011	0 81373	0 87480	0 84556	1 00000	0 00812	0 00070	0 00036
3	34318352	AX-87493871	0 0000018	0 00011	0 43793	0 87480	0 37650	1 00000	0 00756	-0 00216	0 00151
6	60603744	AX-87104257	0 0000018	0 00011	0 01426	0 19062	0 19098	1 00000	0 00603	-0 00556	-0 00554
6	60606762	AX-87277549	0 0000018	0 00011	0 01426	0 19062	0 19098	1 00000	0 00603	-0 00556	-0 00554
15	47895428	AX-86995820	0 0000019	0 00011	0 11036	0 59501	0 60917	1 00000	0 00571	0 00335	-0 00205
20	17033613	AX-87106033	0 0000019	0 00011	0 87251	0 87480	0 74208	1 00000	0 00593	0 00036	-0 00137
18	19417973	AX-87409620	0 0000019	0 00011	0 52885	0 87480	0 60360	1 00000	-0 00571	-0 00135	0 00206
19	50181525	AX-87869547	0 0000019	0 00011	0 57560	0 87480	0 14055	1 00000	0 00842	0 00173	0 00280
5	69327439	AX-87192333	0 0000019	0 00011	0 63732	0 87480	0 45450	1 00000	0 00817	-0 00142	-0 00138
5	13520629	AX-87487004	0 0000019	0 00011	0 86342	0 87480	0 98434	1 00000	-0 00597	-0 00039	0 000082
9	67349910	AX-87564573	0 0000019	0 00011	0 03317	0 31787	0 01005	0 37372	0 00534	-0 00428	0 00966
24	23982222	AX-87144766	0 0000019	0 00011	0 37624	0 87325	0 28045	1 00000	-0 00802	-0 00261	-0 00195
17	9777257	AX-87320641	0 0000019	0 00011	0 07978	0 50877	0 99725	1 00000	0 00594	0 00393	0 000014
21	17638736	AX-86962271	0 0000019	0 00011	0 28929	0 83043	0 77342	1 00000	-0 00559	-0 00223	0 00113
6	34023161	AX-87037601	0 0000019	0 00011	0 57436	0 87480	0 00044	0 04663	0 00754	-0 00156	-0 00598
28	27637594	AX-87192910	0 0000019	0 00011	0 52851	0 87480	0 23112	1 00000	0 00803	0 00186	-0 00217
21	22590716	AX-87811935	0 0000019	0 00011	0 05988	0 43888	0 43359	1 00000	0 00588	-0 00420	-0 00326
16	6128019	AX-87521316	0 0000019	0 00011	0 36254	0 86861	0 29362	1 00000	0 00583	-0 00201	0 00432
1	21245065	AX-87869151	0 0000019	0 00011	0 95610	0 88021	0 79127	1 00000	0 00598	0 00012	0 00111
16	47394533	AX-87392698	0 0000019	0 00011	0 69974	0 87480	0 88246	1 00000	-0 00585	-0 00085	0 00061
27	11945257	AX-87392572	0 0000019	0 00011	0 06563	0 46155	0 95093	1 00000	-0 00558	0 00387	-0 00024
4	46044819	AX-87076662	0 0000019	0 00011	0 89804	0 87482	0 95582	1 00000	-0 00556	0 00027	0 00022
12	87132307	AX-87591848	0 0000019	0 00011	0 02631	0 27721	0 71716	1 00000	0 00574	-0 00484	-0 00147
6	59180390	AX-87541179	0 0000020	0 00012	0 19855	0 74805	0 81185	1 00000	0 00577	0 00280	-0 00097
2	51955261	AX-87163301	0 0000020	0 00012	0 54493	0 87480	0 35446	1 00000	0 00585	-0 00134	0 00382

3	33957481	AX-87566968	0 0000020	0 00012	0 99279	0 88652	0 79979	1 00000	0 00571	-0 00002	0 00102
20	28158083	AX-87608816	0 0000020	0 00012	0 01167	0 16755	0 95972	1 00000	0 00550	-0 00523	-0 00020
20	18043265	AX-87089290	0 0000020	0 00012	0 41424	0 87480	0 48440	1 00000	0 00619	-0 00191	-0 00306
20	22215908	AX-87089643	0 0000020	0 00012	0 94804	0 87891	0 38886	1 00000	0 00569	-0 00014	-0 00346
13	90241262	AX-87853080	0 0000020	0 00012	0 65262	0 87480	0 69516	1 00000	0 00581	0 00100	-0 00162
2	35428899	AX-87537895	0 0000020	0 00012	0 07782	0 50264	0 70996	1 00000	0 00555	-0 00370	0 00146
1	24043303	AX-87155543	0 0000020	0 00012	0 33852	0 85829	0 77570	1 00000	-0 00568	-0 00205	-0 00114
10	122195577	AX-87087172	0 0000020	0 00012	0 11246	0 59986	0 31576	1 00000	0 00588	-0 00353	-0 00416
14	66359214	AX-87747269	0 0000020	0 00012	0 92900	0 87681	0 49150	1 00000	0 00568	-0 00019	-0 00277
13	103287624	AX-87155139	0 0000020	0 00012	0 01046	0 15607	0 73143	1 00000	0 00535	0 00518	0 00130
14	22247330	AX-87426238	0 0000021	0 00012	0 61019	0 87480	0 29899	1 00000	-0 00559	-0 00108	0 00411
6	16601136	AX-87596457	0 0000021	0 00012	0 00025	0 01005	0 61630	1 00000	0 00529	0 00732	0 00187
10	22778879	AX-87302711	0 0000021	0 00012	0 37567	0 87274	0 87213	1 00000	-0 00591	-0 00198	0 00067
4	17393287	AX-87503797	0 0000021	0 00012	0 00431	0 08429	0 34174	1 00000	0 00573	0 00620	0 00385
20	34016277	AX-87648398	0 0000021	0 00012	0 04102	0 35843	0 86602	1 00000	0 00548	-0 00424	0 00065
17	9982980	AX-87583564	0 0000021	0 00012	0 04111	0 35902	0 16299	1 00000	0 00563	-0 00436	0 00556
15	47728131	AX-87099605	0 0000021	0 00012	0 06386	0 45482	0 63278	1 00000	0 00564	0 00396	-0 00191
10	5522421	AX-87466090	0 0000021	0 00012	0 69481	0 87480	0 88038	1 00000	-0 00595	-0 00088	0 00063
6	59181481	AX-86970660	0 0000021	0 00012	0 18097	0 72443	0 74538	1 00000	0 00570	0 00289	-0 00131
13	90149131	AX-87251015	0 0000022	0 00013	0 46085	0 87480	0 77126	1 00000	0 00588	-0 00164	0 00121
21	15191061	AX-87135939	0 0000022	0 00013	0 00203	0 04952	0 77936	1 00000	0 00535	-0 00626	0 00106
21	11709771	AX-87186459	0 0000022	0 00013	0 02634	0 27733	0 51013	1 00000	0 00558	0 00470	0 00260
15	47984763	AX-87448362	0 0000022	0 00013	0 35685	0 86557	0 97066	1 00000	-0 00582	0 00203	-0 00015
21	54078044	AX-87225056	0 0000022	0 00013	0 88405	0 87480	0 99076	1 00000	0 00595	-0 00033	0 00005
3	67134305	AX-87438697	0 0000022	0 00013	0 99888	0 88719	0 05163	0 86835	0 00784	0 00000	-0 00347
20	44559306	AX-87648138	0 0000022	0 00013	0 01671	0 21089	0 00261	0 16138	0 00772	-0 00684	-0 00527
9	125687318	AX-87278831	0 0000022	0 00013	0 44734	0 87480	0 90919	1 00000	-0 00593	-0 00171	0 00048

5	6877595	AX-87542047	0 0000022	0 00013	0 09231	0 54732	0 55434	1 00000	0 00769	-0 00480	0 00103
13	71536415	AX-87315404	0 0000022	0 00013	0 04718	0 38673	0 32108	1 00000	0 00531	-0 00399	0 00373
3	34021554	AX-87863940	0 0000022	0 00013	0 04087	0 35762	0 66766	1 00000	0 00547	-0 00424	0 00167
17	17841646	AX-87139764	0 0000022	0 00013	0 44161	0 87480	0 64915	1 00000	0 00573	0 00167	0 00185
13	58862813	AX-87444610	0 0000022	0 00013	0 01691	0 21238	0 91488	1 00000	0 00760	-0 00674	-0 00018
3	67166311	AX-86959991	0 0000023	0 00013	0 61460	0 87480	0 26704	1 00000	0 00783	0 00146	-0 00198
15	49234058	AX-87726921	0 0000023	0 00013	0 84506	0 87480	0 44447	1 00000	0 00556	-0 00041	-0 00302
29	659209	AX-87824052	0 0000023	0 00013	0 52800	0 87480	0 47336	1 00000	0 00759	0 00176	-0 00124
9	83546326	AX-87459185	0 0000023	0 00013	0 31012	0 84312	0 34070	1 00000	0 00600	-0 00231	0 00406
14	66162708	AX-86990943	0 0000023	0 00013	0 74694	0 87480	0 54391	1 00000	0 00552	-0 00068	-0 00238
25	1957643	AX-87718198	0 0000023	0 00013	0 25521	0 80455	0 64404	1 00000	0 00574	0 00249	0 00189
18	74927642	AX-87031462	0 0000023	0 00013	0 69200	0 87480	0 73470	1 00000	-0 00848	-0 00125	0 00065
6	34111579	AX-86970053	0 0000023	0 00013	0 79155	0 87480	0 49757	1 00000	0 00589	-0 00059	-0 00283
17	8370523	AX-86967252	0 0000023	0 00013	0 05063	0 40175	0 96019	1 00000	0 00596	-0 00443	0 00021
20	18678407	AX-87283363	0 0000023	0 00013	0 77996	0 87480	0 19839	1 00000	0 00765	-0 00079	0 00224
1	20426976	AX-87046213	0 0000023	0 00013	0 34242	0 85928	0 15091	1 00000	0 00574	0 00207	0 00586
27	25011190	AX-87438738	0 0000024	0 00013	0 18484	0 72946	0 32101	1 00000	0 00586	-0 00296	0 00414
3	67177427	AX-87817314	0 0000024	0 00013	0 72062	0 87480	0 29548	1 00000	0 00776	0 00104	-0 00186
13	70791149	AX-87143256	0 0000024	0 00013	0 59142	0 87480	0 35969	1 00000	0 00560	0 00114	0 00364
25	24163064	AX-87863364	0 0000024	0 00014	0 03453	0 32492	0 39264	1 00000	-0 00542	-0 00437	0 00330
19	27400776	AX-86914627	0 0000024	0 00014	0 69885	0 87480	0 24387	1 00000	0 00574	0 00085	0 00476
29	34629755	AX-86946520	0 0000024	0 00014	0 27384	0 81878	0 69472	1 00000	0 00585	-0 00244	0 00163
7	44944131	AX-87110881	0 0000024	0 00014	0 09438	0 55315	0 36075	1 00000	0 00581	-0 00370	-0 00377
19	39742762	AX-87831489	0 0000024	0 00014	0 52485	0 87480	0 90075	1 00000	-0 00619	-0 00150	0 00055
5	31806823	AX-87137907	0 0000024	0 00014	0 23511	0 78754	0 76608	1 00000	0 00586	-0 00265	0 00124
21	26882775	AX-87045475	0 0000024	0 00014	0 80459	0 87480	0 88968	1 00000	0 00584	0 00055	-0 00058
13	90364482	AX-87548556	0 0000024	0 00014	0 31989	0 84799	0 19710	1 00000	0 00551	0 00207	0 00503

10	36283083	AX-87541356	0 0000024	0 00014	0 00265	0 06026	0 62525	1 00000	0 00541	-0 00621	-0 00189
3	58813105	AX-87748943	0 0000024	0 00014	0 00284	0 06342	0 17938	1 00000	0 00545	0 00619	-0 00520
19	23636860	AX-87023090	0 0000024	0 00014	0 00779	0 12685	0 84994	1 00000	0 00595	0 00602	0 00080
19	23653893	AX-87514215	0 0000024	0 00014	0 00779	0 12685	0 84994	1 00000	0 00595	0 00602	0 00080
19	23661998	AX-87443737	0 0000024	0 00014	0 00779	0 12685	0 84994	1 00000	0 00595	0 00602	0 00080
4	11201691	AX-86927078	0 0000024	0 00014	0 01176	0 16841	0 69360	1 00000	0 00532	0 00497	0 00149
17	63695055	AX-87547618	0 0000025	0 00014	0 71218	0 87480	0 96963	1 00000	-0 00543	-0 00076	0 00015
20	46594587	AX-87807382	0 0000025	0 00014	0 00693	0 11781	0 51260	1 00000	0 00559	-0 00575	-0 00261
1	78806811	AX-87224299	0 0000025	0 00014	0 11910	0 61545	0 69255	1 00000	0 00581	-0 00345	0 00164
6	60879642	AX-87322713	0 0000025	0 00014	0 71164	0 87480	0 61808	1 00000	0 00562	-0 00079	-0 00199
22	12683111	AX-87266624	0 0000025	0 00014	0 02985	0 29915	0 35545	1 00000	0 00750	-0 00607	-0 00158
13	89489141	AX-87088081	0 0000025	0 00014	0 13022	0 63884	0 05423	0 88706	0 00766	-0 00432	-0 00337
13	91927717	AX-86959834	0 0000025	0 00014	0 32155	0 84903	0 07926	1 00000	0 00612	-0 00231	-0 00764
9	102708059	AX-86905162	0 0000025	0 00014	3.41053734490	0 00206	0 61756	1 00000	0 00526	0 00831	0 00187
29	129082	AX-87176082	0 0000025	0 00014	0 47751	0 87480	0 00445	0 22841	0 00763	-0 00202	-0 00496
20	21449299	AX-87224039	0 0000025	0 00014	0 06599	0 46302	0 64245	1 00000	0 00568	-0 00398	0 00188
16	76339601	AX-87196716	0 0000025	0 00014	0 00415	0 08210	0 30621	1 00000	0 00564	-0 00617	0 00412
5	55445475	AX-87608577	0 0000025	0 00014	0 05832	0 43263	0 80653	1 00000	0 00550	-0 00397	-0 00096
13	102565952	AX-87862309	0 0000026	0 00014	0 74028	0 87480	0 73385	1 00000	-0 00577	-0 00073	0 00140
13	67900615	AX-87172375	0 0000026	0 00014	0 49668	0 87480	0 37376	1 00000	-0 00774	-0 00196	0 00157
3	34476415	AX-87626656	0 0000026	0 00014	0 08299	0 51899	0 91778	1 00000	0 00540	-0 00358	-0 00040
13	89823710	AX-86997725	0 0000026	0 00015	0 03637	0 33442	0 57706	1 00000	0 00773	-0 00604	-0 00099
20	86819334	AX-87140014	0 0000026	0 00015	0 08824	0 53568	0 82551	1 00000	-0 00583	0 00380	-0 00092
14	66381726	AX-87015315	0 0000026	0 00015	0 15281	0 68041	0 70270	1 00000	0 00561	0 00306	-0 00153
9	56473964	AX-87765275	0 0000026	0 00015	0 60622	0 87480	0 86734	1 00000	-0 00582	-0 00115	-0 00069
6	33939918	AX-87475781	0 0000026	0 00015	0 76864	0 87480	0 45614	1 00000	0 00582	-0 00065	-0 00310
7	26994949	AX-87424767	0 0000026	0 00015	0 02168	0 24704	0 00100	0 08454	0 00742	-0 00637	0 00559

1	12232981	AX-87577897	0 0000026	0 00015	0 90802	0 87515	0 22723	1 00000	0 00584	0 00026	0 00503
17	49379365	AX-87193305	0 0000026	0 00015	0 04517	0 37761	0 69585	1 00000	0 00568	-0 00434	0 00158
2	85069556	AX-86941507	0 0000026	0 00015	0 21287	0 76345	0 16712	1 00000	0 00546	-0 00258	0 00538
22	12657593	AX-87858675	0 0000026	0 00015	0 21938	0 77062	0 13063	1 00000	0 00782	-0 00359	0 00270
15	26354577	AX-87572111	0 0000026	0 00015	0 38761	0 87480	0 91029	1 00000	0 00582	-0 00192	-0 00047
29	42668547	AX-87588336	0 0000026	0 00015	0 84760	0 87480	0 23386	1 00000	0 00563	-0 00041	-0 00477
11	82068162	AX-87657359	0 0000026	0 00015	0 91760	0 87564	0 73782	1 00000	-0 00592	0 00023	-0 00142
4	31952291	AX-87860464	0 0000027	0 00015	0 60018	0 87480	0 82945	1 00000	-0 00612	-0 00123	0 00094
7	45562838	AX-87446811	0 0000027	0 00015	0 32187	0 84927	0 76219	1 00000	0 00569	-0 00216	0 00123
1	164477218	AX-87694558	0 0000027	0 00015	0 95897	0 88076	0 40571	1 00000	0 00544	0 00011	-0 00323
1	140276728	AX-87655442	0 0000027	0 00015	0 03173	0 30981	0 94524	1 00000	0 00593	-0 00488	0 00029
14	11645047	AX-87040181	0 0000027	0 00015	0 67078	0 87480	0 82072	1 00000	-0 00576	-0 00094	0 00093
22	6449933	AX-87809801	0 0000027	0 00015	0 70639	0 87480	0 99525	1 00000	-0 00562	-0 00081	0 00002
6	34671704	AX-87136539	0 0000027	0 00015	0 29193	0 83149	0 54041	1 00000	0 00562	0 00227	-0 00243
15	41082111	AX-87177263	0 0000027	0 00015	0 68043	0 87480	0 21992	1 00000	0 00765	0 00118	0 00215
21	37609670	AX-87741599	0 0000027	0 00015	0 77339	0 87480	0 71991	1 00000	0 00796	0 00086	0 00065
13	81672854	AX-87697547	0 0000027	0 00015	0 00999	0 15127	0 66137	1 00000	-0 00550	-0 00542	0 00172
5	23577640	AX-86978920	0 0000027	0 00015	0 92871	0 87674	0 33393	1 00000	0 00564	-0 00019	0 00390
23	3501782	AX-87832206	0 0000027	0 00015	0 00563	0 10211	0 00000	0 00001	0 00408	-0 00432	0 01617
5	22633180	AX-87136347	0 0000028	0 00015	0 69260	0 87480	0 76338	1 00000	0 00595	-0 00090	-0 00128
7	53828728	AX-87433940	0 0000028	0 00016	0 25726	0 80590	0 08404	1 00000	0 00560	-0 00243	-0 00694
15	47684169	AX-87306086	0 0000028	0 00016	0 05404	0 41632	0 64471	1 00000	0 00554	0 00409	-0 00183
20	45127671	AX-87694466	0 0000028	0 00016	0 10482	0 57994	0 34692	1 00000	0 00584	-0 00363	-0 00393
1	140584995	AX-87633983	0 0000028	0 00016	0 04407	0 37247	0 44584	1 00000	0 00555	-0 00429	0 00303
22	2072208	AX-87565122	0 0000028	0 00016	0 00085	0 02573	0 76946	1 00000	0 00547	0 00699	-0 00115
16	78049474	AX-87246714	0 0000028	0 00016	0 38640	0 87480	0 79411	1 00000	-0 00583	0 00193	0 00109
26	12404554	AX-87382447	0 0000028	0 00016	0 41547	0 87480	0 97424	1 00000	0 00568	0 00177	-0 00013

26	22716917	AX-87507484	0 0000029	0 00016	0 70846	0 87480	0 77125	1 00000	0 00562	-0 00081	-0 00118
16	56211127	AX-87483944	0 0000029	0 00016	0 01105	0 16128	0 24568	1 00000	0 00562	-0 00548	0 00468
10	29535898	AX-87130928	0 0000029	0 00016	0 90672	0 87515	0 82925	1 00000	0 00566	0 00025	-0 00088
6	25821234	AX-87007142	0 0000029	0 00016	0 74410	0 87480	0 00133	0 10278	0 00755	-0 00093	-0 00558
18	52672131	AX-86978868	0 0000029	0 00016	0 16339	0 69828	0 01459	0 45798	-0 00756	-0 00395	0 00424
7	8574242	AX-87647602	0 0000029	0 00016	0 05735	0 42848	0 03867	0 76075	0 00500	-0 00366	-0 00743
23	42485630	AX-87748008	0 0000029	0 00016	0 71833	0 87480	0 47006	1 00000	-0 00591	-0 00082	0 00306
18	4140283	AX-86950918	0 0000029	0 00016	0 05472	0 41858	0 42627	1 00000	0 00523	-0 00386	-0 00299
13	91021559	AX-87834351	0 0000029	0 00016	0 02466	0 26675	0 47930	1 00000	0 00525	-0 00453	-0 00267
25	42134663	AX-86968983	0 0000029	0 00016	0 87712	0 87480	0 68887	1 00000	0 00601	-0 00036	0 00173
7	26993751	AX-87687319	0 0000029	0 00016	0 02316	0 25673	0 00127	0 09997	0 00742	-0 00633	0 00551
21	11725585	AX-87274898	0 0000030	0 00016	0 02692	0 28124	0 50976	1 00000	0 00551	0 00468	0 00260
11	9965058	AX-87760846	0 0000030	0 00016	0 56557	0 87480	0 82528	1 00000	-0 00575	-0 00127	0 00091
11	9970330	AX-87141219	0 0000030	0 00016	0 56557	0 87480	0 82528	1 00000	-0 00575	-0 00127	0 00091
19	17774875	AX-87315962	0 0000030	0 00016	0 81941	0 87480	0 96753	1 00000	0 00569	-0 00050	0 00017
28	3062505	AX-87432495	0 0000030	0 00016	0 50042	0 87480	0 26023	1 00000	0 00567	-0 00147	-0 00458
3	32455685	AX-87258250	0 0000030	0 00016	0 05425	0 41725	0 78685	1 00000	0 00567	-0 00420	-0 00110
4	80510365	AX-87654629	0 0000030	0 00016	0 82121	0 87480	0 84416	1 00000	-0 00556	-0 00048	0 00079
4	39342189	AX-86929451	0 0000030	0 00016	0 65741	0 87480	0 98051	1 00000	-0 00534	-0 00091	-0 00009
23	3121577	AX-87730744	0 0000030	0 00016	0 00279	0 06264	0 00000	0 00000	0 00404	-0 00465	0 01690
21	41106012	AX-86988605	0 0000030	0 00017	0 62748	0 87480	0 78553	1 00000	-0 00518	0 00097	0 00101
16	1835444	AX-87534927	0 0000030	0 00017	0 35926	0 86674	0 31911	1 00000	0 00561	-0 00197	0 00399
1	165420742	AX-87301286	0 0000030	0 00017	0 67993	0 87480	0 40008	1 00000	0 01056	0 00054	-0 00209
6	39235923	AX-87363359	0 0000030	0 00017	0 64798	0 87480	0 26156	1 00000	0 00554	0 00097	-0 00447
22	15801691	AX-86911036	0 0000031	0 00017	0 39324	0 87480	0 50825	1 00000	0 00568	0 00187	-0 00270
14	30615695	AX-87586951	0 0000031	0 00017	0 14457	0 66635	0 64960	1 00000	0 00552	-0 00310	0 00180
14	30636232	AX-87511926	0 0000031	0 00017	0 14457	0 66635	0 64960	1 00000	0 00552	-0 00310	0 00180

11	40751345	AX-87850223	0 0000031	0 00017	0 02452	0 26600	0 74714	1 00000	0 00558	-0 00483	0 00129
25	7410212	AX-87862069	0 0000031	0 00017	0 09652	0 55945	0 84675	1 00000	0 00584	-0 00373	-0 00081
21	50479140	AX-87756399	0 0000031	0 00017	0 55803	0 87480	0 79447	1 00000	-0 00578	-0 00130	0 00107
15	79463808	AX-87108941	0 0000031	0 00017	0 46281	0 87480	0 87961	1 00000	0 00556	-0 00157	0 00061
19	30916748	AX-87789807	0 0000031	0 00017	0 92480	0 87616	0 92888	1 00000	-0 00612	-0 00022	0 00039
18	21833683	AX-87190666	0 0000031	0 00017	0 03011	0 30072	0 51174	1 00000	0 00546	0 00456	0 00258
6	44862205	AX-87675629	0 0000031	0 00017	0 62663	0 87480	0 85562	1 00000	-0 00562	-0 00105	0 00073
25	13218488	AX-87337605	0 0000031	0 00017	0 05711	0 42787	0 90285	1 00000	0 00540	-0 00396	0 00047
27	34478382	AX-87696534	0 0000031	0 00017	0 64914	0 87480	0 10503	1 00000	0 00563	0 00099	-0 00656
15	79488066	AX-87485922	0 0000031	0 00017	0 76366	0 87480	0 42411	1 00000	0 00558	-0 00065	-0 00321
3	31203970	AX-87513142	0 0000031	0 00017	0 17594	0 71671	0 57757	1 00000	0 00540	-0 00282	-0 00216
4	60982375	AX-87335465	0 0000031	0 00017	0 69246	0 87480	0 96180	1 00000	0 00567	-0 00086	0 00020
1	132129571	AX-87642499	0 0000031	0 00017	0 36431	0 86917	0 50982	1 00000	-0 00572	-0 00200	0 00271
16	23649625	AX-87128392	0 0000032	0 00017	0 29956	0 83565	0 40127	1 00000	-0 00547	0 00219	0 00332
11	97887181	AX-87400379	0 0000032	0 00017	0 45662	0 87480	0 90811	1 00000	-0 00571	0 00164	0 00047
7	27209023	AX-87647410	0 0000032	0 00017	0 02026	0 23681	0 50330	1 00000	0 00553	-0 00495	-0 00267
2	19965087	AX-87477835	0 0000032	0 00017	0 77024	0 87480	0 76638	1 00000	-0 00618	-0 00069	0 00132
10	110263346	AX-87761268	0 0000032	0 00017	0 65813	0 87480	0 98545	1 00000	-0 00604	-0 00103	-0 00008
20	2800360	AX-87746387	0 0000032	0 00017	0 28184	0 82621	0 90750	1 00000	0 00574	0 00238	-0 00048
13	58051209	AX-87735107	0 0000032	0 00017	0 00777	0 12663	0 63288	1 00000	0 00562	-0 00577	-0 00193
22	35389424	AX-86986344	0 0000032	0 00017	0 05422	0 41718	0 69115	1 00000	0 00576	-0 00427	-0 00165
18	58045627	AX-87730840	0 0000032	0 00017	0 71959	0 87480	0 91156	1 00000	-0 00610	-0 00084	0 00049
5	7061148	AX-87547559	0 0000032	0 00017	0 17291	0 71253	0 92060	1 00000	0 00564	-0 00298	-0 00041
1	132743497	AX-87431883	0 0000032	0 00017	0 05176	0 40620	0 40970	1 00000	0 00553	-0 00413	-0 00329
14	19377834	AX-87512062	0 0000032	0 00017	0 88134	0 87480	0 61082	1 00000	-0 00575	-0 00033	0 00211
26	22018807	AX-87116841	0 0000033	0 00017	0 30262	0 83843	0 49230	1 00000	0 00541	-0 00215	0 00268
10	15546787	AX-86987337	0 0000033	0 00017	0 03781	0 34255	0 31987	1 00000	0 00572	-0 00458	0 00410

24	29735363	AX-86990318	0 0000033	0 00017	0 54640	0 87480	0 75420	1 00000	0 00573	0 00133	-0 00129
9	67289220	AX-87376815	0 0000033	0 00018	0 24485	0 79677	0 02972	0 67109	0 00536	-0 00240	0 00840
28	39844980	AX-87849507	0 0000033	0 00018	0 33930	0 85863	0 36312	1 00000	0 00550	0 00203	0 00361
5	23579199	AX-87200858	0 0000033	0 00018	0 96899	0 88282	0 33482	1 00000	0 00560	-0 00008	0 00392
5	27071286	AX-87331303	0 0000033	0 00018	0 72626	0 87480	0 93239	1 00000	-0 00585	-0 00079	0 00036
11	14560564	AX-87437909	0 0000033	0 00018	0 01199	0 17067	0 90197	1 00000	0 00535	-0 00519	-0 00048
11	30577830	AX-87856007	0 0000033	0 00018	0 54000	0 87480	0 81942	1 00000	-0 00572	-0 00135	-0 00094
9	2598870	AX-87229922	0 0000033	0 00018	0 63217	0 87480	0 82836	1 00000	0 00554	0 00103	0 00087
3	78947890	AX-87871072	0 0000034	0 00018	0 97753	0 88431	0 69315	1 00000	0 00578	6.29633805062	0 00165
13	28477490	AX-86946042	0 0000034	0 00018	0 02723	0 28325	0 82606	1 00000	0 00542	0 00463	0 00086
3	69513078	AX-87046260	0 0000034	0 00018	0 43256	0 87480	0 72260	1 00000	-0 00516	-0 00158	0 00132
20	21656862	AX-86998141	0 0000034	0 00018	0 32879	0 85405	0 83583	1 00000	0 00555	-0 00210	0 00083
6	77225048	AX-87047880	0 0000034	0 00018	0 67625	0 87480	0 95369	1 00000	-0 00573	0 00093	0 00024
25	17452049	AX-87109770	0 0000034	0 00018	0 04940	0 39694	0 24568	1 00000	0 00764	-0 00567	-0 00205
10	25899638	AX-87399599	0 0000034	0 00018	0 24422	0 79634	0 36367	1 00000	-0 00548	-0 00247	0 00360
11	10552957	AX-87063892	0 0000034	0 00018	0 03434	0 32394	0 87141	1 00000	0 00568	-0 00464	0 00066
17	34457984	AX-87411496	0 0000034	0 00018	0 08214	0 51663	0 93975	1 00000	0 00766	0 00503	-0 00013
11	17332335	AX-87361027	0 0000034	0 00018	0 17230	0 71161	0 45528	1 00000	0 00540	0 00285	0 00291
27	13990697	AX-87428984	0 0000034	0 00018	0 29667	0 83410	0 86038	1 00000	0 00538	0 00217	-0 00068
20	50393476	AX-86967479	0 0000035	0 00018	0 55812	0 87480	0 50201	1 00000	0 00575	-0 00131	0 00279
17	15714612	AX-87697914	0 0000035	0 00018	0 07998	0 50940	0 77742	1 00000	0 00565	0 00383	0 00115
16	76348531	AX-87775678	0 0000035	0 00019	0 00416	0 08215	0 32934	1 00000	0 00560	-0 00621	0 00395
9	79685449	AX-87821450	0 0000035	0 00019	0 10578	0 58226	0 29881	1 00000	0 00546	0 00342	0 00410
9	67966917	AX-87680859	0 0000035	0 00019	0 22348	0 77524	0 82184	1 00000	0 00565	-0 00266	0 00092
18	15162892	AX-87298675	0 0000035	0 00019	0 22843	0 77970	0 22879	1 00000	0 00545	0 00254	-0 00474
4	16849868	AX-87098511	0 0000036	0 00019	0 55548	0 87480	0 63166	1 00000	0 00576	0 00132	0 00200
1	137676743	AX-87549886	0 0000036	0 00019	0 66204	0 87480	0 67379	1 00000	-0 00572	-0 00097	0 00174

11	99086788	AX-87149022	0 0000036	0 00019	0 89085	0 87480	0 93838	1 00000	0 00550	-0 00029	-0 00031
9	22898251	AX-87551920	0 0000036	0 00019	0 94396	0 87831	0 58460	1 00000	0 00572	-0 00016	0 00226
21	25758550	AX-87026350	0 0000036	0 00019	0 54025	0 87480	0 98261	1 00000	0 00556	0 00132	0 00009
6	25353790	AX-87514420	0 0000036	0 00019	0 16686	0 70323	0 96401	1 00000	0 00568	-0 00305	0 00018
5	64539615	AX-87015506	0 0000036	0 00019	0 09064	0 54292	0 51042	1 00000	0 00590	-0 00388	0 00279
20	27721634	AX-87585037	0 0000036	0 00019	0 48555	0 87480	0 82603	1 00000	-0 00550	-0 00149	-0 00087
15	85356442	AX-86943453	0 0000036	0 00019	0 12846	0 63490	0 88375	1 00000	0 00594	-0 00350	0 00063
20	17971430	AX-87534781	0 0000036	0 00019	0 31660	0 84662	0 29368	1 00000	0 00581	-0 00226	-0 00441
7	19142145	AX-87698398	0 0000036	0 00019	0 70301	0 87480	0 40239	1 00000	0 00550	0 00081	-0 00333
19	41756779	AX-87870881	0 0000036	0 00019	0 59611	0 87480	0 57092	1 00000	-0 00569	-0 00117	0 00233
18	4913760	AX-87497661	0 0000036	0 00019	0 20957	0 76036	0 74318	1 00000	0 00584	-0 00284	0 00139
19	22498667	AX-87376392	0 0000037	0 00019	0 18319	0 72766	0 93350	1 00000	-0 00545	-0 00282	-0 00033
6	59331540	AX-87280118	0 0000037	0 00019	0 12236	0 62194	0 41498	1 00000	0 00549	-0 00329	-0 00324
27	13872108	AX-87143015	0 0000037	0 00019	0 55940	0 87480	0 46212	1 00000	0 00585	0 00132	0 00312
17	28102473	AX-87012825	0 0000037	0 00019	0 79189	0 87480	0 37896	1 00000	0 00786	0 00079	-0 00161
1	35251390	AX-87227206	0 0000037	0 00019	0 65701	0 87480	0 82113	1 00000	-0 00564	-0 00098	0 00093
22	37422799	AX-87059637	0 0000037	0 00019	0 11870	0 61447	0 81240	1 00000	0 00594	-0 00360	-0 00102
22	37431394	AX-87255016	0 0000037	0 00019	0 11870	0 61447	0 81240	1 00000	0 00594	-0 00360	-0 00102
22	37433980	AX-87235144	0 0000037	0 00019	0 11870	0 61447	0 81240	1 00000	0 00594	-0 00360	-0 00102
3	31158917	AX-87117580	0 0000037	0 00019	0 73089	0 87480	0 91931	1 00000	0 00808	-0 00105	-0 00019
16	5489654	AX-87153533	0 0000037	0 00019	0 68356	0 87480	0 67512	1 00000	0 00593	0 00094	0 00180
20	17003252	AX-87702110	0 0000037	0 00019	0 71908	0 87480	0 59116	1 00000	0 00593	0 00083	-0 00231
10	5770986	AX-87631435	0 0000037	0 00019	0 91085	0 87515	0 63349	1 00000	-0 00543	-0 00024	0 00188
1	94135645	AX-87109201	0 0000037	0 00019	0 59920	0 87480	0 95808	1 00000	-0 00828	-0 00166	0 00010
29	261452	AX-87389991	0 0000037	0 00019	0 56424	0 87480	0 01073	0 38733	0 00752	-0 00165	-0 00446
1	94504851	AX-87033610	0 0000037	0 00019	0 55483	0 87480	0 91573	1 00000	-0 00814	-0 00183	0 00020
9	129193392	AX-87128329	0 0000038	0 00020	0 25926	0 80778	0 84031	1 00000	-0 00553	-0 00242	-0 00081

15	61380271	AX-86980883	0 0000038	0 00020	0 36935	0 87086	0 98280	1 00000	-0 00579	-0 00202	-0 00009
15	61417130	AX-87586608	0 0000038	0 00020	0 36935	0 87086	0 98280	1 00000	-0 00579	-0 00202	-0 00009
20	33437297	AX-87391261	0 0000038	0 00020	0 96598	0 88220	0 77484	1 00000	0 00757	0 00012	0 00050
20	26397266	AX-87441058	0 0000038	0 00020	0 52718	0 87480	0 56055	1 00000	0 00591	0 00145	0 00250
13	67543890	AX-87761883	0 0000038	0 00020	0 21414	0 76463	0 60607	1 00000	-0 00550	-0 00265	0 00206
20	51935539	AX-87289983	0 0000038	0 00020	0 11211	0 59934	0 14575	1 00000	0 00559	-0 00345	0 00585
14	31234717	AX-87431330	0 0000038	0 00020	0 85215	0 87480	0 05815	0 91093	0 00771	0 00055	-0 00340
21	39381561	AX-87816610	0 0000038	0 00020	0 78785	0 87480	0 73405	1 00000	0 00558	-0 00058	0 00138
20	84989481	AX-87362550	0 0000038	0 00020	0 17647	0 71745	0 66324	1 00000	0 00565	-0 00297	0 00179
23	42647304	AX-87794042	0 0000038	0 00020	0 65539	0 87480	0 71078	1 00000	-0 00573	-0 00099	0 00154
3	24228071	AX-87080699	0 0000038	0 00020	0 91100	0 87515	0 79242	1 00000	-0 00578	-0 00025	-0 00111
18	19182325	AX-87173120	0 0000038	0 00020	0 10989	0 59352	0 65936	1 00000	-0 00556	0 00346	0 00178
10	17544592	AX-86969827	0 0000038	0 00020	0 18166	0 72541	0 96266	1 00000	0 00549	-0 00285	0 00019
7	26661970	AX-87827178	0 0000038	0 00020	0 36380	0 86916	0 40917	1 00000	-0 00572	0 00202	-0 00343
7	16577261	AX-87737014	0 0000038	0 00020	0 83929	0 87480	0 22842	1 00000	0 00571	-0 00045	0 00500
27	13979078	AX-87452043	0 0000039	0 00020	0 30602	0 84053	0 92789	1 00000	0 00546	0 00217	-0 00036
13	63890661	AX-87190759	0 0000039	0 00020	0 51485	0 87480	0 80819	1 00000	-0 00548	0 00139	0 00097
14	19646492	AX-87199041	0 0000039	0 00020	0 40379	0 87480	0 47081	1 00000	-0 00566	-0 00184	0 00297
9	111965579	AX-87026671	0 0000039	0 00020	0 02332	0 25784	0 77892	1 00000	0 00569	-0 00502	-0 00116
12	52354191	AX-87514580	0 0000039	0 00020	0 76575	0 87480	0 00041	0 04386	0 00767	-0 00087	-0 00633
18	20680123	AX-87523992	0 0000039	0 00020	0 55104	0 87480	0 99084	1 00000	-0 00585	-0 00136	-0 00005
16	75615201	AX-87685732	0 0000039	0 00020	0 11089	0 59642	0 60301	1 00000	0 00541	-0 00336	0 00205
9	23365798	AX-87073164	0 0000039	0 00020	0 13095	0 64016	0 77387	1 00000	-0 00615	0 00361	0 00128
1	78470948	AX-87868432	0 0000039	0 00020	0 18562	0 73040	0 99903	1 00000	0 00577	-0 00297	0 00001
1	60264667	AX-87593035	0 0000040	0 00020	0 17836	0 72034	0 73756	1 00000	-0 00568	-0 00297	-0 00138
3	54467352	AX-87125248	0 0000040	0 00020	0 20620	0 75704	0 47740	1 00000	0 00797	-0 00383	0 00132
17	66288064	AX-87423213	0 0000040	0 00020	0 00124	0 03433	0 68270	1 00000	-0 00529	0 00665	0 00157

13	102396695	AX-87296952	0 0000040	0 00020	0 67025	0 87480	0 88637	1 00000	-0 00549	-0 00091	0 00057
19	26113201	AX-87844223	0 0000040	0 00020	0 61504	0 87480	0 74849	1 00000	0 00811	0 00155	0 00061
23	2019308	AX-87630158	0 0000040	0 00020	0 02925	0 29600	0 00000	0 00022	0 00439	-0 00372	0 01584
10	80903760	AX-87251326	0 0000040	0 00021	0 49876	0 87480	0 81686	1 00000	-0 00559	0 00147	0 00094
12	87098505	AX-87131048	0 0000040	0 00021	0 02249	0 25232	0 44243	1 00000	0 00547	-0 00486	0 00305
18	52699924	AX-87750581	0 0000041	0 00021	0 16081	0 69447	0 01152	0 40153	-0 00749	-0 00400	0 00442
6	59322000	AX-87490892	0 0000041	0 00021	0 12321	0 62345	0 41834	1 00000	0 00550	-0 00330	-0 00324
16	7467446	AX-87666882	0 0000041	0 00021	0 04422	0 37310	0 69271	1 00000	0 00543	0 00425	-0 00156
13	59816672	AX-87084712	0 0000041	0 00021	0 21680	0 76742	0 25604	1 00000	0 00565	-0 00272	-0 00467
22	39998226	AX-87649285	0 0000041	0 00021	0 90414	0 87500	0 76041	1 00000	0 00601	-0 00028	0 00133
13	70845043	AX-87602361	0 0000041	0 00021	0 66473	0 87480	0 48544	1 00000	0 00782	-0 00129	0 00127
13	70849314	AX-87175550	0 0000041	0 00021	0 66473	0 87480	0 48544	1 00000	0 00782	-0 00129	0 00127
1	139654338	AX-87182202	0 0000041	0 00021	0 79839	0 87480	0 79225	1 00000	0 00810	0 00079	0 00050
8	19731767	AX-87158396	0 0000041	0 00021	0 63344	0 87480	0 78475	1 00000	-0 00564	-0 00105	0 00112
14	31770589	AX-86911014	0 0000042	0 00021	0 02555	0 27258	0 97305	1 00000	-0 00532	0 00463	-0 00013
7	44925628	AX-87870116	0 0000042	0 00021	0 03489	0 32666	0 73932	1 00000	0 00561	-0 00462	-0 00136
13	71195326	AX-87598868	0 0000042	0 00021	0 08205	0 51638	0 48446	1 00000	0 00517	-0 00351	0 00263
6	13258092	AX-87568891	0 0000042	0 00021	0 85155	0 87480	0 41450	1 00000	0 00585	0 00043	0 00348
2	27732868	AX-87484600	0 0000042	0 00021	0 01252	0 17561	0 77011	1 00000	0 00552	0 00538	-0 00118
6	33394977	AX-87511722	0 0000042	0 00021	0 46898	0 87480	0 57531	1 00000	0 00584	-0 00165	-0 00239
28	31506740	AX-87076709	0 0000042	0 00021	0 37057	0 87110	0 60328	1 00000	0 00569	-0 00199	-0 00216
5	34750551	AX-87597337	0 0000043	0 00022	0 05049	0 40124	0 83264	1 00000	0 00563	-0 00430	0 00087
13	22524539	AX-87864487	0 0000043	0 00022	0 40154	0 87480	0 91705	1 00000	-0 00551	-0 00181	0 00042
9	27341937	AX-87153180	0 0000043	0 00022	0 94564	0 87865	0 73976	1 00000	-0 00556	0 00015	0 00135
6	51672409	AX-87742349	0 0000043	0 00022	0 00414	0 08201	0 91488	1 00000	0 00543	0 00608	-0 00042
18	31287911	AX-87858960	0 0000043	0 00022	0 63403	0 87480	0 98132	1 00000	-0 00599	-0 00111	-0 00010
7	8509411	AX-86909174	0 0000043	0 00022	0 91452	0 87532	0 07176	0 99902	0 00503	-0 00021	-0 00661

9	129064181	AX-87069414	0 0000043	0 00022	0 69944	0 87480	0 72758	1 00000	-0 00570	0 00086	-0 00145
20	21092077	AX-87150786	0 0000043	0 00022	0 16258	0 69685	0 72438	1 00000	0 00549	-0 00300	-0 00141
16	5499301	AX-87445806	0 0000043	0 00022	0 69386	0 87480	0 87400	1 00000	-0 00570	-0 00088	-0 00066
11	10612719	AX-87547763	0 0000043	0 00022	0 03475	0 32610	0 82514	1 00000	0 00574	-0 00474	0 00093
1	132114167	AX-87587039	0 0000043	0 00022	0 61237	0 87480	0 46874	1 00000	-0 00561	-0 00111	0 00297
5	43831371	AX-87235821	0 0000044	0 00022	0 09372	0 55095	0 50640	1 00000	0 00732	0 00470	0 00114
2	13047422	AX-86941049	0 0000044	0 00022	0 02392	0 26182	0 23577	1 00000	-0 00593	-0 00523	0 00514
9	148512458	AX-87668946	0 0000044	0 00022	0 04475	0 37565	0 89730	1 00000	0 00558	0 00438	0 00053
25	1977458	AX-87692772	0 0000044	0 00022	0 68013	0 87480	0 56369	1 00000	0 00572	0 00092	0 00240
14	95892981	AX-87859409	0 0000044	0 00022	0 63992	0 87480	0 46159	1 00000	0 00577	0 00106	0 00309
19	16914020	AX-87499597	0 0000044	0 00022	0 60992	0 87480	0 63020	1 00000	-0 00823	-0 00160	0 00093
14	74776682	AX-87864259	0 0000044	0 00022	0 02277	0 25439	0 86432	1 00000	0 00577	0 00514	-0 00072
1	79406142	AX-87340729	0 0000044	0 00022	0 75388	0 87480	0 12783	1 00000	0 00568	-0 00070	0 00632
22	17168551	AX-86903109	0 0000045	0 00022	0 03205	0 31136	0 80296	1 00000	0 00573	0 00481	-0 00105
9	57076837	AX-87538775	0 0000045	0 00022	0 61603	0 87480	0 80494	1 00000	-0 00547	-0 00107	0 00099
13	66477976	AX-87567999	0 0000045	0 00022	0 46472	0 87480	0 61846	1 00000	-0 00553	-0 00158	0 00201
25	16955473	AX-87346607	0 0000045	0 00022	0 05447	0 41792	0 26777	1 00000	0 00767	-0 00564	-0 00199
25	16983063	AX-87809478	0 0000045	0 00022	0 05447	0 41792	0 26777	1 00000	0 00767	-0 00564	-0 00199
18	19187769	AX-87104459	0 0000045	0 00022	0 10655	0 58447	0 58961	1 00000	-0 00564	0 00358	0 00221
5	66822737	AX-87306985	0 0000045	0 00023	0 80761	0 87480	0 62131	1 00000	-0 00583	-0 00056	0 00211
20	44690869	AX-87630951	0 0000045	0 00023	0 02129	0 24438	0 00390	0 20983	0 00760	-0 00670	-0 00515
5	48368614	AX-87604541	0 0000045	0 00023	0 14720	0 67078	0 54682	1 00000	0 00783	0 00438	0 00111
14	99695123	AX-87769342	0 0000045	0 00023	0 98754	0 88588	0 37146	1 00000	0 00563	-0 00003	0 00368
1	165259638	AX-87221326	0 0000045	0 00023	0 89605	0 87480	0 96679	1 00000	-0 00819	-0 00041	-0 00008
13	79767336	AX-87266702	0 0000045	0 00023	0 76741	0 87480	0 36084	1 00000	-0 00571	-0 00066	0 00382
15	45323556	AX-87566100	0 0000046	0 00023	0 28344	0 82687	0 09753	1 00000	0 00764	0 00314	-0 00297
15	45327879	AX-87180195	0 0000046	0 00023	0 28344	0 82687	0 09753	1 00000	0 00764	0 00314	-0 00297

28	5693998	AX-87758001	0 0000046	0 00023	0 67497	0 87480	0 86861	1 00000	-0 00585	-0 00096	0 00071
16	56342653	AX-87579020	0 0000046	0 00023	0 12781	0 63344	0 62687	1 00000	0 00558	-0 00333	0 00198
1	85021159	AX-86932768	0 0000046	0 00023	0 39043	0 87480	0 42578	1 00000	-0 00780	0 00257	0 00146
17	70891004	AX-87392604	0 0000046	0 00023	0 06222	0 44868	0 47993	1 00000	0 00529	-0 00389	-0 00275
25	13177953	AX-87583623	0 0000046	0 00023	0 06831	0 47069	0 92669	1 00000	0 00660	-0 00475	0 00045
25	32629156	AX-87569199	0 0000046	0 00023	0 11253	0 59994	0 87267	1 00000	-0 00561	-0 00349	0 00066
9	23984917	AX-87315609	0 0000046	0 00023	0 32276	0 84998	0 62272	1 00000	0 00814	-0 00309	0 00094
7	56586757	AX-87324318	0 0000046	0 00023	0 84862	0 87480	0 79317	1 00000	0 00565	-0 00042	-0 00109
1	85182396	AX-87550639	0 0000046	0 00023	0 66840	0 87480	0 29192	1 00000	-0 00792	0 00130	0 00196
18	51060797	AX-87584775	0 0000047	0 00023	0 17914	0 72174	0 01714	0 49787	-0 00750	-0 00386	0 00418
1	94461344	AX-86965027	0 0000047	0 00023	0 80441	0 87480	0 97932	1 00000	0 00800	-0 00076	-0 00005
10	110291935	AX-87099718	0 0000047	0 00023	0 66100	0 87480	0 98845	1 00000	-0 00590	-0 00102	-0 00006
21	22798175	AX-87023935	0 0000047	0 00023	0 39121	0 87480	0 33052	1 00000	0 00567	-0 00191	-0 00405
1	21318133	AX-87870813	0 0000047	0 00023	0 84391	0 87480	0 92308	1 00000	0 00554	-0 00043	-0 00039
21	37435144	AX-86916373	0 0000047	0 00023	0 72924	0 87480	0 70506	1 00000	0 00780	0 00104	0 00069
29	12482479	AX-87613182	0 0000048	0 00023	0 02364	0 25978	0 24530	1 00000	0 00526	-0 00467	-0 00448
12	82758879	AX-87616253	0 0000048	0 00023	0 65775	0 87480	0 71638	1 00000	-0 00557	-0 00097	-0 00148
18	51469331	AX-87515218	0 0000048	0 00024	0 20155	0 75200	0 04279	0 79780	-0 00761	-0 00373	0 00362
13	58813986	AX-87190367	0 0000048	0 00024	0 02080	0 24075	0 86265	1 00000	0 00575	-0 00522	0 00073
3	32894172	AX-87585263	0 0000048	0 00024	0 07568	0 49565	0 63825	1 00000	0 00556	-0 00387	-0 00192
9	56493723	AX-87837079	0 0000048	0 00024	0 61581	0 87480	0 86826	1 00000	-0 00569	-0 00112	-0 00069
9	56497174	AX-87439397	0 0000048	0 00024	0 61581	0 87480	0 86826	1 00000	-0 00569	-0 00112	-0 00069
15	44227566	AX-87537342	0 0000048	0 00024	0 70281	0 87480	0 83152	1 00000	0 00562	0 00084	0 00088
15	44277976	AX-87125354	0 0000048	0 00024	0 70281	0 87480	0 83152	1 00000	0 00562	0 00084	0 00088
20	27571417	AX-86954391	0 0000048	0 00024	0 21043	0 76078	0 42455	1 00000	0 00556	-0 00274	-0 00327
20	92191894	AX-87184861	0 0000048	0 00024	0 78811	0 87480	0 91645	1 00000	0 00539	-0 00057	-0 00041
25	8543330	AX-87534637	0 0000048	0 00024	0 47987	0 87480	0 84641	1 00000	0 00571	0 00158	0 00081

5	69255606	AX-86995210	0 0000048	0 00024	0 59294	0 87480	0 84150	1 00000	-0 00601	-0 00126	-0 00088
13	90244272	AX-87674568	0 0000048	0 00024	0 01096	0 16045	0 84428	1 00000	0 00548	0 00547	0 00079
17	8426655	AX-86944610	0 0000049	0 00024	0 08293	0 51888	0 69812	1 00000	0 00560	-0 00381	0 00159
18	54554652	AX-87059707	0 0000049	0 00024	0 50077	0 87480	0 50176	1 00000	-0 00557	-0 00147	0 00274
4	44387485	AX-87413852	0 0000049	0 00024	0 62611	0 87480	0 90574	1 00000	-0 00565	-0 00108	0 00049
20	28399645	AX-87095634	0 0000049	0 00024	0 15806	0 69059	0 63674	1 00000	0 00554	-0 00305	0 00192
7	49488250	AX-87588420	0 0000049	0 00024	0 45174	0 87480	0 28301	1 00000	0 00551	-0 00163	-0 00434
4	78388625	AX-87402777	0 0000049	0 00024	0 08080	0 51189	0 52106	1 00000	0 00529	-0 00363	0 00249
1	78668612	AX-87449995	0 0000049	0 00024	0 15855	0 69164	0 71316	1 00000	0 00580	-0 00322	-0 00157
20	42902950	AX-87541195	0 0000049	0 00024	0 01574	0 20295	4.640488940499	0 00793	0 00729	-0 00676	-0 00699
21	17412490	AX-87008914	0 0000049	0 00024	0 62685	0 87480	0 61843	1 00000	-0 00774	-0 00145	0 00091
2	37926892	AX-87854507	0 0000049	0 00024	0 16878	0 70599	0 50835	1 00000	-0 00578	-0 00312	0 00281
1	161757586	AX-87711986	0 0000050	0 00024	0 05633	0 42496	0 64168	1 00000	0 00534	0 00401	0 00181
9	147506524	AX-87325548	0 0000050	0 00024	0 14991	0 67570	0 64410	1 00000	0 00574	-0 00325	-0 00195
7	21426634	AX-87725716	0 0000050	0 00024	0 04999	0 39943	0 10655	1 00000	0 00541	-0 00417	-0 00641
10	80810671	AX-87165383	0 0000050	0 00024	0 30431	0 83941	0 75353	1 00000	-0 00565	0 00228	0 00130
1	51186812	AX-87708945	0 0000050	0 00024	0 03419	0 32318	1.644592340353	0 00046	0 00423	-0 00353	0 01491
11	10571301	AX-87166731	0 0000051	0 00025	0 51917	0 87480	0 79501	1 00000	-0 00554	-0 00141	0 00106
4	31765670	AX-87538818	0 0000051	0 00025	0 68812	0 87480	0 81871	1 00000	-0 00585	-0 00086	0 00099
18	58358951	AX-87246957	0 0000051	0 00025	0 65200	0 87480	0 90715	1 00000	-0 00598	-0 00107	0 00051
3	4976851	AX-87681758	0 0000051	0 00025	0 92184	0 87602	0 75128	1 00000	-0 00506	-0 00020	0 00118
20	21495483	AX-87120426	0 0000051	0 00025	0 51992	0 87480	0 81644	1 00000	0 00571	-0 00145	-0 00098
3	68178761	AX-87172735	0 0000051	0 00025	0 29243	0 83167	0 22535	1 00000	0 00554	-0 00230	-0 00494
11	21548068	AX-87018774	0 0000051	0 00025	0 33451	0 85682	0 46533	1 00000	-0 00547	-0 00210	0 00297
15	26432117	AX-87383400	0 0000052	0 00025	0 22092	0 77261	0 99663	1 00000	0 00560	-0 00270	-0 00002
18	51914586	AX-87672063	0 0000052	0 00025	0 17233	0 71161	0 01267	0 42365	-0 00747	-0 00394	0 00441
17	66257334	AX-87118110	0 0000052	0 00025	0 00310	0 06733	0 69753	1 00000	-0 00526	0 00613	0 00151

4	39766114	AX-87682317	0 0000052	0 00025	0 15965	0 69302	0 46599	1 00000	0 00539	-0 00298	-0 00289
18	58310942	AX-86965612	0 0000052	0 00025	0 63720	0 87480	0 90998	1 00000	-0 00596	-0 00111	0 00050
1	91869404	AX-87540571	0 0000052	0 00025	0 53659	0 87480	0 86089	1 00000	-0 00814	-0 00194	-0 00034
21	49621948	AX-86922506	0 0000052	0 00025	0 21647	0 76730	0 30051	1 00000	0 00587	0 00286	-0 00448
20	85670152	AX-87225158	0 0000052	0 00025	0 11769	0 61206	0 41330	1 00000	0 00564	-0 00348	0 00340
9	23905281	AX-87005183	0 0000053	0 00025	0 00092	0 02746	0 54785	1 00000	0 01043	-0 00437	-0 00151
28	5717644	AX-87614618	0 0000053	0 00025	0 94154	0 87802	0 94215	1 00000	-0 00580	-0 00017	-0 00031
12	73562202	AX-87395944	0 0000053	0 00025	0 67165	0 87480	0 96683	1 00000	0 00571	-0 00095	-0 00018
20	2304251	AX-87028213	0 0000053	0 00025	0 50151	0 87480	0 77645	1 00000	-0 00563	-0 00149	-0 00118
5	55772055	AX-87511458	0 0000053	0 00025	0 23830	0 79089	0 08363	1 00000	0 00543	-0 00252	-0 00692
1	25033465	AX-87233392	0 0000053	0 00026	0 30316	0 83872	0 73215	1 00000	0 00532	0 00207	0 00133
18	58326467	AX-87661496	0 0000053	0 00026	0 63031	0 87480	0 91506	1 00000	-0 00591	-0 00112	0 00046
14	89724850	AX-87240021	0 0000053	0 00026	0 02923	0 29582	0 96529	1 00000	0 00568	-0 00488	-0 00018
21	22720535	AX-87227939	0 0000053	0 00026	0 00712	0 11961	0 08789	1 00000	0 00684	-0 00710	-0 00276
11	21471189	AX-87213508	0 0000054	0 00026	0 32720	0 85320	0 50384	1 00000	-0 00559	-0 00216	0 00276
27	13699057	AX-87464803	0 0000054	0 00026	0 21109	0 76142	0 34984	1 00000	0 00537	0 00265	-0 00370
9	67892290	AX-87234117	0 0000054	0 00026	0 23363	0 78559	0 77322	1 00000	0 00555	-0 00261	0 00118
3	55833191	AX-87392329	0 0000054	0 00026	0 02519	0 27035	0 09520	1 00000	0 00542	-0 00479	-0 00667
18	15159277	AX-87400912	0 0000054	0 00026	0 20752	0 75822	0 23640	1 00000	0 00533	0 00265	-0 00466
3	56959920	AX-87503853	0 0000054	0 00026	0 22062	0 77221	0 71286	1 00000	0 00530	-0 00256	-0 00144
3	32458324	AX-86932530	0 0000054	0 00026	0 86257	0 87480	0 95011	1 00000	-0 00548	0 00037	0 00025
15	19245804	AX-87202643	0 0000054	0 00026	0 33751	0 85806	0 71836	1 00000	0 00530	0 00201	0 00141
29	18289227	AX-87146833	0 0000054	0 00026	0 46630	0 87480	0 71622	1 00000	-0 00556	-0 00160	0 00149
1	79373255	AX-86944321	0 0000055	0 00026	0 72901	0 87480	0 13149	1 00000	0 00565	-0 00077	0 00629
12	88687820	AX-87088994	0 0000055	0 00026	0 42155	0 87480	0 98683	1 00000	-0 00552	-0 00175	-0 00007
13	68533868	AX-86953760	0 0000055	0 00026	0 65035	0 87480	0 26852	1 00000	-0 00536	-0 00096	0 00438
1	92850383	AX-87771461	0 0000055	0 00026	0 08155	0 51447	0 70037	1 00000	-0 00994	-0 00219	0 00093

6	33268491	AX-87259931	0 0000055	0 00026	0 84404	0 87480	0 48026	1 00000	0 00577	-0 00045	0 00301
21	40789042	AX-86923893	0 0000055	0 00026	0 26900	0 81517	0 44448	1 00000	0 00779	-0 00333	0 00141
15	47383153	AX-87236263	0 0000055	0 00026	0 82600	0 87480	0 58223	1 00000	0 00558	0 00048	-0 00226
16	78008155	AX-87047630	0 0000055	0 00026	0 72820	0 87480	0 58683	1 00000	-0 00585	0 00080	0 00235
20	65325404	AX-87273589	0 0000055	0 00026	0 12909	0 63634	0 57609	1 00000	0 00572	-0 00343	0 00236
22	26739435	AX-87703694	0 0000055	0 00026	0 55950	0 87480	0 38833	1 00000	0 00560	0 00129	-0 00357
13	80125522	AX-87296541	0 0000056	0 00026	0 75670	0 87480	0 40221	1 00000	0 00577	-0 00071	-0 00357
29	30269781	AX-87788914	0 0000056	0 00026	0 20891	0 75958	0 97787	1 00000	0 00551	-0 00274	-0 00011
7	12442069	AX-86903444	0 0000056	0 00027	0 20177	0 75228	0 34942	1 00000	0 00586	-0 00296	0 00406
7	44974422	AX-87557867	0 0000056	0 00027	0 59724	0 87480	0 82625	1 00000	0 00586	-0 00122	-0 00095
18	4906083	AX-87676901	0 0000056	0 00027	0 21077	0 76103	0 74210	1 00000	0 00574	-0 00284	0 00139
20	88480495	AX-86910572	0 0000056	0 00027	0 87037	0 87480	0 95365	1 00000	0 00562	-0 00036	-0 00024
14	46763571	AX-87159483	0 0000056	0 00027	0 22100	0 77269	0 43304	1 00000	-0 00550	0 00266	0 00319
6	25296555	AX-87467912	0 0000057	0 00027	0 15507	0 68536	0 81121	1 00000	0 00567	-0 00319	0 00100
29	3191317	AX-87660567	0 0000057	0 00027	0 55912	0 87480	0 05709	0 90529	0 00751	0 00170	-0 00340
9	57067083	AX-86908742	0 0000057	0 00027	0 69183	0 87480	0 80153	1 00000	-0 00552	-0 00087	0 00103
9	68761216	AX-87016023	0 0000057	0 00027	0 08211	0 51650	0 26535	1 00000	0 00532	0 00366	-0 00438
9	58285368	AX-87481178	0 0000057	0 00027	0 70887	0 87480	0 72999	1 00000	-0 00539	-0 00080	0 00138
12	78193648	AX-87035729	0 0000057	0 00027	0 22797	0 77946	0 58906	1 00000	0 00564	0 00269	0 00225
7	26778036	AX-87387528	0 0000057	0 00027	0 28080	0 82568	0 94883	1 00000	0 00591	-0 00255	-0 00028
5	67856622	AX-87471276	0 0000057	0 00027	0 74619	0 87480	0 07978	1 00000	0 00785	-0 00098	-0 00326
12	78401325	AX-87821939	0 0000058	0 00027	0 34177	0 85913	0 00573	0 26755	0 00748	-0 00275	0 00490
12	72565667	AX-87550977	0 0000058	0 00027	0 91561	0 87544	0 21688	1 00000	-0 00804	-0 00033	0 00236
1	21106869	AX-87311477	0 0000058	0 00027	0 05078	0 40238	0 39374	1 00000	0 00566	-0 00438	0 00357
2	42570706	AX-87206234	0 0000058	0 00027	0 00094	0 02781	0 18499	1 00000	0 00982	0 00412	-0 00316
1	85048487	AX-87041818	0 0000059	0 00028	0 16142	0 69529	0 02391	0 60074	0 00763	-0 00414	-0 00409
8	10456647	AX-87565634	0 0000059	0 00028	0 22476	0 77644	0 82617	1 00000	-0 00559	-0 00269	-0 00091

28	3128551	AX-87226074	0 0000059	0 00028	0 34794	0 86107	0 36044	1 00000	0 00584	-0 00217	-0 00395
10	114296550	AX-87257906	0 0000059	0 00028	0 14019	0 65766	0 57294	1 00000	-0 00534	-0 00312	-0 00223
18	52342472	AX-87364330	0 0000059	0 00028	0 17472	0 71493	0 01338	0 43674	-0 00745	-0 00392	0 00438
18	52524300	AX-87582544	0 0000059	0 00028	0 16498	0 70056	0 01343	0 43742	-0 00743	-0 00400	0 00436
21	37477971	AX-87412534	0 0000059	0 00028	0 97330	0 88353	0 45106	1 00000	0 00787	-0 00010	0 00141
13	72070575	AX-87067870	0 0000060	0 00028	0 09100	0 54384	0 65601	1 00000	0 00521	-0 00349	0 00172
3	14403924	AX-87319160	0 0000060	0 00028	0 96436	0 88187	0 85830	1 00000	0 00556	9.85761015357	0 00074
3	14404880	AX-87536685	0 0000060	0 00028	0 96436	0 88187	0 85830	1 00000	0 00556	9.85761015357	0 00074
26	23296607	AX-87502147	0 0000060	0 00028	0 79721	0 87480	0 93518	1 00000	-0 00524	-0 00053	0 00032
19	30970104	AX-87610201	0 0000060	0 00028	0 59675	0 87480	0 95917	1 00000	-0 00588	-0 00123	0 00022
1	95627019	AX-87300751	0 0000061	0 00029	0 69373	0 87480	0 95824	1 00000	-0 00807	-0 00124	0 00010
1	21780163	AX-87702200	0 0000061	0 00029	0 02757	0 28560	0 36119	1 00000	0 00545	0 00481	0 00372
20	32727341	AX-87584159	0 0000061	0 00029	0 10082	0 57017	0 32610	1 00000	0 00540	-0 00351	-0 00393
13	57430882	AX-87069358	0 0000062	0 00029	0 03775	0 34213	0 67995	1 00000	0 00551	-0 00456	0 00168
11	37820081	AX-86955232	0 0000062	0 00029	0 12671	0 63127	0 12731	1 00000	-0 00544	-0 00332	-0 00619
9	35580625	AX-87445524	0 0000062	0 00029	0 25039	0 80057	0 88251	1 00000	0 00575	0 00263	0 00063
3	34074160	AX-87358863	0 0000063	0 00029	0 07240	0 48483	0 32283	1 00000	0 00533	-0 00381	0 00391
20	28222132	AX-87084866	0 0000063	0 00029	0 02471	0 26713	0 83375	1 00000	0 00535	-0 00478	0 00083
17	9569292	AX-87475659	0 0000063	0 00029	0 04080	0 35743	0 65964	1 00000	0 00554	-0 00450	0 00181
13	24729587	AX-87648982	0 0000063	0 00029	0 98063	0 88480	0 84732	1 00000	0 00584	-0 00006	-0 00083
26	4712992	AX-87535779	0 0000063	0 00029	0 00842	0 13414	0 02002	0 54713	0 00481	0 00507	0 00836
3	37368941	AX-87830599	0 0000063	0 00030	0 85741	0 87480	0 84317	1 00000	-0 00755	-0 00053	-0 00036
9	56599711	AX-87497142	0 0000063	0 00030	0 67444	0 87480	0 97382	1 00000	-0 00558	-0 00093	-0 00014
18	14759101	AX-87577649	0 0000063	0 00030	0 67429	0 87480	0 55364	1 00000	0 00562	0 00094	0 00248
1	56458813	AX-87683227	0 0000064	0 00030	0 42780	0 87480	0 35500	1 00000	0 00601	-0 00189	0 00413
22	26316123	AX-87067964	0 0000064	0 00030	0 64139	0 87480	0 31103	1 00000	0 00550	-0 00102	-0 00414
15	46766671	AX-86907696	0 0000065	0 00030	0 89953	0 87487	0 76751	1 00000	0 00790	-0 00039	-0 00055

16	46738422	AX-87002861	0 0000065	0 00030	0 53802	0 87480	0 73679	1 00000	-0 00553	-0 00136	0 00138
13	70641703	AX-87697058	0 0000065	0 00030	0 12021	0 61798	0 50451	1 00000	0 00563	-0 00350	0 00281
22	39585378	AX-87210398	0 0000065	0 00030	0 43229	0 87480	0 10825	1 00000	0 00750	-0 00229	0 00287
9	36193556	AX-87076909	0 0000065	0 00030	0 38443	0 87480	0 16730	1 00000	0 00761	-0 00258	-0 00251
1	94262396	AX-87286341	0 0000065	0 00030	0 54493	0 87480	0 90995	1 00000	-0 00794	-0 00187	0 00021
7	51950858	AX-87312796	0 0000065	0 00030	0 46252	0 87480	0 82422	1 00000	-0 00560	-0 00165	0 00093
7	48091259	AX-87261819	0 0000065	0 00030	0 13015	0 63875	0 89260	1 00000	0 00555	0 00334	0 00056
4	15484096	AX-87223806	0 0000065	0 00030	0 43455	0 87480	0 86755	1 00000	-0 00545	-0 00169	-0 00068
18	51722835	AX-87189522	0 0000066	0 00030	0 16156	0 69529	0 01373	0 44245	-0 00741	-0 00404	0 00436
9	71692684	AX-86933779	0 0000066	0 00030	0 27594	0 82066	0 13270	1 00000	0 00754	-0 00320	-0 00271
19	56851534	AX-87563349	0 0000066	0 00030	0 68821	0 87480	0 78420	1 00000	-0 00577	-0 00092	0 00118
17	11281738	AX-86972408	0 0000066	0 00030	0 03068	0 30389	0 20666	1 00000	0 00520	-0 00449	-0 00490
1	23709775	AX-87507487	0 0000066	0 00030	0 05664	0 42595	0 91702	1 00000	0 00540	-0 00410	-0 00042
20	28656413	AX-87401884	0 0000066	0 00030	0 99382	0 88666	0 89300	1 00000	0 00555	1.71130685535	0 00055
26	4819513	AX-87834932	0 0000066	0 00031	0 02051	0 23856	0 00072	0 06628	0 00470	-0 00433	0 01183
3	14583549	AX-87371406	0 0000066	0 00031	0 14591	0 66848	0 08110	1 00000	0 00515	0 00298	-0 00669

Table S3 The top 1% of associations with early run timing in 11 populations of North American Atlantic salmon, identified using a partial redundancy analysis (pRDA) that accounts for population structure by using the first three PC axes that describe population structure. Distance from the closest gene is reported alongside where in the genome the gene starts and ends.

Chromosome	Position	gene	loadings	SNP	start_gene	end_gene	distance
ssa01	7392462	LOC106605409	0 065215317	AX-87571622	7396282	7420337	3820
ssa01	12619549	LOC106561950	0 061667991	AX-87561060	12345532	12629141	0
ssa01	12629814	LOC106561950	0 061667991	AX-87417211	12345532	12629141	-674
ssa01	12630040	LOC106561950	0 054838643	AX-87107156	12345532	12629141	-900
ssa01	12653352	LOC106561950	0 057550256	AX-87695928	12345532	12629141	-24212
ssa01	12657963	LOC106561950	0 057550256	AX-87264958	12345532	12629141	-28823
ssa01	12985948	tmem30b	0 055512263	AX-87347495	12972983	12984775	-1174
ssa01	13250743	LOC106562977	0 054066874	AX-86999271	13285804	13295369	35061
ssa01	13509081	stac	0 055248301	AX-87613507	13468235	13513539	0
ssa01	19420048	LOC106571863	0 053130878	AX-86905179	19404170	19509883	0
ssa01	19463143	LOC106571863	0 054093653	AX-87492610	19404170	19509883	0
ssa01	19878846	zgc:113142	0 05351069	AX-87805385	19873115	19878943	0
ssa01	19887377	zgc:113142	0 05351069	AX-87366988	19873115	19878943	-8435
ssa01	20743821	LOC106574021	0 057910045	AX-87035012	20733824	20760265	0
ssa01	22460693	LOC123741685	0 052718943	AX-87870249	22460543	22463120	0
ssa01	22714381	LOC106577674	0 059132408	AX-87043500	22662540	22697858	-16524
ssa01	27512965	LOC106590872	0 055844981	AX-87401327	27527950	27547378	14985
ssa01	27527834	LOC106590872	0 055844981	AX-87383789	27527950	27547378	116
ssa01	33937044	spred1	0 056096915	AX-87460816	33936131	34010454	0
ssa01	33938272	spred1	0 056096915	AX-87187413	33936131	34010454	0
ssa01	38538817	LOC123742703	0 063218595	AX-87742520	38546221	38548096	7404
ssa01	38543801	LOC123742703	0 058699136	AX-87867270	38546221	38548096	2420
ssa01	43509604	dtnbb	0 061462147	AX-87177802	43509715	43565816	111
ssa01	43509899	dtnbb	0 061462147	AX-87106905	43509715	43565816	0
ssa01	44555063	fuca2	0 067975628	AX-87828169	44545409	44558425	0
ssa01	44555922	fuca2	0 067975628	AX-87365002	44545409	44558425	0
ssa01	45293507	efcab11	0 071362273	AX-87655415	45233969	45328543	0
ssa01	45311830	efcab11	0 071362273	AX-87719425	45233969	45328543	0
ssa01	45313323	efcab11	0 071362273	AX-87579225	45233969	45328543	0
ssa01	45735929	dio2	0 05726237	AX-87614099	45721217	45730704	-5226
ssa01	45750914	nrxn3a	0 057193141	AX-86932838	45762605	46196313	11691
ssa01	45752432	nrxn3a	0 05726237	AX-87243685	45762605	46196313	10173
ssa01	45800633	nrxn3a	0 058300086	AX-87525646	45762605	46196313	0
ssa01	45801203	nrxn3a	0 058300086	AX-87583580	45762605	46196313	0
ssa01	45898389	nrxn3a	0 074090069	AX-87709550	45762605	46196313	0
ssa01	53825747	LOC106604940	0 056001457	AX-86906364	53658244	53944416	0

ssa01	53825810	LOC106604940	0	056001457	AX-86958248	53658244	53944416	0
ssa01	53826044	LOC106604940	0	056001457	AX-87584676	53658244	53944416	0
ssa01	55129279	LOC106605143	0	059948293	AX-87311282	55129796	55133916	517
ssa01	55132984	LOC106605143	0	065085941	AX-87201266	55129796	55133916	0
ssa01	55179010	LOC106605150	0	059948293	AX-87337249	55140010	55189274	0
ssa01	55766044	LOC106605212	0	062975251	AX-87809440	55796340	55806181	30296
ssa01	55817235	LOC106605225	0	078759945	AX-86938232	55812677	55815852	-1384
ssa01	55818834	LOC106605225	0	078759945	AX-87047447	55812677	55815852	-2983
ssa01	55827335	LOC106605225	0	078759945	AX-87710487	55812677	55815852	-11484
ssa01	55829659	LOC106605225	0	078759945	AX-87093474	55812677	55815852	-13808
ssa01	56060116	LOC106605231	0	055920986	AX-87753889	56007732	56101031	0
ssa01	56076757	LOC106605231	0	053095793	AX-87717494	56007732	56101031	0
ssa01	56132900	LOC106608624	0	065986029	AX-87812594	56126327	56157437	0
ssa01	56140259	LOC106608624	0	056229644	AX-87058000	56126327	56157437	0
ssa01	56140401	LOC106608624	0	056229644	AX-87046299	56126327	56157437	0
ssa01	56232347	LOC106605748	0	053475942	AX-87004842	56242354	56283696	10007
ssa01	56415376	LOC106608641	0	063229422	AX-87553729	56420077	56422819	4701
ssa01	56417602	LOC106608641	0	054251895	AX-87723539	56420077	56422819	2475
ssa01	56427797	LOC106605756	0	054251895	AX-86914220	56424520	56430414	0
ssa01	56430321	LOC106605756	0	054251895	AX-87049880	56424520	56430414	0
ssa01	56445407	LOC106605759	0	054251895	AX-87094174	56433342	56477749	0
ssa01	56479398	LOC106605759	0	063229422	AX-87713517	56433342	56477749	-1650
ssa01	56548763	LOC123726801	0	054334702	AX-87410332	56525963	56526017	-22747
ssa01	56713858	LOC106605787	0	06476955	AX-87062541	56709114	56813838	0
ssa01	56726585	LOC106605787	0	054875075	AX-87831708	56709114	56813838	0
ssa01	56726769	LOC106605787	0	054875075	AX-87291013	56709114	56813838	0
ssa01	56825302	LOC106605805	0	052725478	AX-87617228	56824018	56829112	0
ssa01	56846265	LOC100306816	0	059044187	AX-87777355	56841915	56850221	0
ssa01	56938811	LOC106605561	0	059014349	AX-87445628	56899661	57047655	0
ssa01	56943700	LOC106605561	0	05879258	AX-87854209	56899661	57047655	0
ssa01	56984460	LOC106605561	0	060600175	AX-87685006	56899661	57047655	0
ssa01	56990553	LOC106605561	0	060600175	AX-87620573	56899661	57047655	0
ssa01	57011445	LOC106605561	0	063415231	AX-87055610	56899661	57047655	0
ssa01	57095050	LOC106605853	0	056860593	AX-87121174	57069917	57099192	0
ssa01	57095050	LOC106605882	0	056860593	AX-87121174	57088483	57103902	0
ssa01	57098339	LOC106605853	0	056860593	AX-87564028	57069917	57099192	0
ssa01	57098339	LOC106605882	0	056860593	AX-87564028	57088483	57103902	0
ssa01	57161946	LOC106605890	0	057125142	AX-87724192	57120776	57177520	0
ssa01	57312271	LOC106605956	0	064118856	AX-87678436	57270770	57313327	0
ssa01	57320494	LOC106605956	0	055165759	AX-87627492	57270770	57313327	-7168
ssa01	57377804	LOC106605977	0	059986482	AX-86997426	57355499	57358883	-18922
ssa01	57481515	LOC106605994	0	056732358	AX-87023677	57479783	57524575	0

ssa01	57568796	LOC106606050	0 052799976	AX-87359293	57568148	57632192	0
ssa01	57815419	LOC106606127	0 057552785	AX-87578615	57778096	57850402	0
ssa01	58231201	zgc:153169	0 056136701	AX-87576819	58230800	58248919	0
ssa01	58231980	zgc:153169	0 056136701	AX-87466849	58230800	58248919	0
ssa01	58232592	zgc:153169	0 056136701	AX-86914041	58230800	58248919	0
ssa01	58289551	LOC106606259	0 057513867	AX-87392538	58283394	58289425	-127
ssa01	58290773	LOC106606259	0 063423936	AX-87303834	58283394	58289425	-1349
ssa01	58293528	LOC106606259	0 071780788	AX-87824709	58283394	58289425	-4104
ssa01	58743759	LOC106606370	0 059996253	AX-87554172	58712915	58833730	0
ssa01	58747482	LOC106606370	0 059996253	AX-87430759	58712915	58833730	0
ssa01	58748015	LOC106606370	0 059996253	AX-87522706	58712915	58833730	0
ssa01	59266674	eno4	0 0543356	AX-86948384	59212009	59255398	-11277
ssa01	59362967	LOC100136392	0 0543356	AX-87796103	59347692	59401735	0
ssa01	59380697	LOC100136392	0 066979687	AX-86919058	59347692	59401735	0
ssa01	59441328	LOC106606525	0 053918904	AX-87457711	59423621	59434368	-6961
ssa01	59448716	LOC106608681	0 053918904	AX-87531906	59460010	59470565	11294
ssa01	60292674	LOC106606765	0 053381744	AX-87787225	60288215	60308698	0
ssa01	60295080	LOC106606765	0 053381744	AX-87040778	60288215	60308698	0
ssa01	60297613	LOC106606765	0 061368052	AX-87859889	60288215	60308698	0
ssa01	60374618	LOC106606783	0 06816407	AX-86963575	60377396	60395902	2778
ssa01	60422079	LOC106606791	0 063984524	AX-87223438	60405813	60453482	0
ssa01	60442290	LOC106606791	0 059222372	AX-87575493	60405813	60453482	0
ssa01	60469321	LOC106606834	0 065859249	AX-87592828	60457346	60657835	0
ssa01	60549815	LOC106606834	0 063768348	AX-86979520	60457346	60657835	0
ssa01	60581673	LOC106606834	0 054831218	AX-87401840	60457346	60657835	0
ssa01	60877667	LOC106606915	0 067781371	AX-87410458	60829260	60976575	0
ssa01	60971990	LOC106606915	0 058208344	AX-87153585	60829260	60976575	0
ssa01	61860634	LOC123743478	0 059862451	AX-87317657	61816150	61872935	0
ssa01	67147780	LOC106607602	0 064653443	AX-87024848	66971640	67451859	0
ssa01	67175920	LOC106607602	0 064653443	AX-87360858	66971640	67451859	0
ssa01	67194059	LOC106607602	0 064653443	AX-87551727	66971640	67451859	0
ssa01	67389694	LOC106607602	0 060383466	AX-87718815	66971640	67451859	0
ssa01	67875532	LOC106607666	0 05314758	AX-87126816	67822731	67838522	-37011
ssa01	68717138	LOC100380754	0 063056926	AX-87454143	68706002	68728126	0
ssa01	71776868	LOC106608059	0 070078203	AX-87287156	71714359	71832730	0
ssa01	130210306	LOC106562660	0 053590596	AX-87746568	130193678	130218093	0
ssa01	147537345	LOC106567541	0 066251386	AX-87742457	147227423	147581276	0
ssa01	149031977	LOC106568217	0 058391761	AX-87820190	149030729	149034180	0
ssa01	149165708	aggf1	0 066422182	AX-86927077	149156812	149168544	0
ssa01	149235247	LOC106568179	0 058225661	AX-87869422	149168895	149266389	0
ssa01	149366450	LOC106568153	0 054647125	AX-87214867	149356576	149443024	0
ssa01	156838592	si:dkey-34e4.1	0 055756307	AX-87203692	156724613	156902767	0

ssa01	165153907	diras2	0 071440122	AX-87592229	165143811	165148300	-5608
ssa01	165171265	syk	0 066104239	AX-87549834	165168482	165199624	0
ssa01	165180392	syk	0 064748702	AX-87422292	165168482	165199624	0
ssa01	165194420	syk	0 069305484	AX-87815712	165168482	165199624	0
ssa01	165197623	syk	0 06890953	AX-87187831	165168482	165199624	0
ssa01	165259638	auh	0 066368043	AX-87221326	165255039	165313509	0
ssa01	165532610	cdc42se2	0 053987084	AX-87110671	165525623	165603887	0
ssa02	11195722	LOC106605968	0 063278112	AX-87480965	11188585	11216852	0
ssa02	11860808	LOC106606099	0 054511216	AX-87032270	11854260	11877912	0
ssa02	17929154	LOC123728151	0 058547814	AX-87640947	17919745	17932420	0
ssa02	18330332	LOC106594007	0 069823291	AX-87240026	18323674	18326467	-3866
ssa02	18594512	LOC106605884	0 0874853	AX-87333933	18596816	18725685	2304
ssa02	19153123	LOC100196392	0 086548313	AX-87133131	19141689	19151734	-1390
ssa02	19810010	LOC106605802	0 089215596	AX-87137437	19801156	19815345	0
ssa02	19860236	LOC106605785	0 075716363	AX-87465288	19864012	19893098	3776
ssa02	19965087	LOC106573792	0 077116464	AX-87477835	19960849	19963701	-1387
ssa02	25358521	hspb6	0 054231736	AX-87654477	25355595	25358217	-305
ssa02	29205611	LOC106579301	0 055216843	AX-87630079	29209884	29216957	4273
ssa02	29595923	LOC106579226	0 053166073	AX-87081737	29585512	29595040	-884
ssa02	30934010	LOC106579633	0 055092051	AX-87483071	30904627	30964602	0
ssa02	32406791	LOC106580193	0 05393649	AX-86983593	32405426	32408940	0
ssa02	38477666	LOC106584585	0 055271705	AX-87307895	38468743	38481749	0
ssa02	42020053	LOC106583316	0 05640762	AX-87663432	41993383	42019476	-578
ssa02	42023849	LOC106583328	0 054869512	AX-87069185	42021084	42030055	0
ssa02	52694295	LOC106585904	0 053397143	AX-87096340	52685244	52696535	0
ssa02	53340259	LOC106585713	0 053061022	AX-87339176	53339034	53417855	0
ssa02	53340990	LOC106585713	0 0554571	AX-87131943	53339034	53417855	0
ssa02	71089340	LOC123730896	0 052806856	AX-87055604	71078010	71089272	-69
ssa03	25344688	LOC106599368	0 057225201	AX-87460700	25332478	25335368	-9321
ssa03	25349108	LOC106599368	0 054719043	AX-87807405	25332478	25335368	-13741
ssa03	39141770	LOC106600038	0 055680746	AX-87655536	39042986	39140361	-1410
ssa03	40433343	LOC106599971	0 07044363	AX-87526168	40345175	40422560	-10784
ssa03	52342729	LOC106600762	0 05957523	AX-87509228	52341756	52344651	0
ssa03	52815644	LOC106600786	0 061722492	AX-87666211	52814764	52815566	-79
ssa03	57278920	LOC106600850	0 061442513	AX-87021999	57273182	57281180	0
ssa03	89048645	LOC106601908	0 054009509	AX-87525125	89042410	89048735	0
ssa04	7534344	LOC106602223	0 056211205	AX-87720679	7532562	7538110	0
ssa04	15142325	LOC106602365	0 059659398	AX-87298834	15012815	15347042	0
ssa04	21750503	LOC106602526	0 057087055	AX-86950545	21745919	21772302	0
ssa04	21844824	LOC106602567	0 052838707	AX-87764578	21843361	21847234	0
ssa04	21917389	LOC106602566	0 056508738	AX-87013625	21916725	21925744	0
ssa04	21931098	fgg	0 053507268	AX-87786263	21928815	21934830	0

ssa04	22024044	LOC106602561	0 053900228	AX-87555077	22023896	22105725	0
ssa04	22025485	LOC106602561	0 057674163	AX-87139939	22023896	22105725	0
ssa04	22112449	LOC106602561	0 057876899	AX-87754785	22023896	22105725	-6725
ssa04	27763179	LOC106602710	0 065927055	AX-87780373	27740599	27777924	0
ssa04	27886441	dchs2	0 066605867	AX-87816170	27846242	27903429	0
ssa04	27927847	sfrp2	0 06163224	AX-87386483	27927198	27930326	0
ssa04	28034168	LOC106602747	0 060429955	AX-87053220	27956830	28036483	0
ssa04	28042441	mnd1	0 056114204	AX-87258112	28038707	28066000	0
ssa04	28087913	trim2a	0 053691696	AX-87325882	28067178	28129608	0
ssa04	28102133	trim2a	0 065679468	AX-87300785	28067178	28129608	0
ssa04	28115551	trim2a	0 097228193	AX-87169195	28067178	28129608	0
ssa04	28122619	trim2a	0 064962688	AX-87375068	28067178	28129608	0
ssa04	28129131	trim2a	0 061534203	AX-87820843	28067178	28129608	0
ssa04	28129803	trim2a	0 077535178	AX-87610300	28067178	28129608	-196
ssa04	28135851	trim2a	0 07802213	AX-87141324	28067178	28129608	-6244
ssa04	28260727	arfip1	0 066083798	AX-87593836	28224813	28262787	0
ssa04	28373625	LOC106602739	0 080328609	AX-87059812	28366083	28385879	0
ssa04	28380566	LOC106602739	0 084634798	AX-87577886	28366083	28385879	0
ssa04	28389841	LOC106602741	0 091516285	AX-87309553	28386468	28394657	0
ssa04	28395055	LOC106602741	0 085271169	AX-87528243	28386468	28394657	-399
ssa04	28472093	LOC123742573	0 077540708	AX-86937126	28458698	28460027	-12067
ssa04	28549984	LOC123742437	0 077665096	AX-87738679	28558683	28561408	8699
ssa04	28563413	LOC123742438	0 060258628	AX-86919556	28562244	28562719	-695
ssa04	28655198	LOC106602736	0 083775727	AX-87740197	28677753	28690256	22555
ssa04	28704950	myoz2b	0 082944349	AX-87819245	28695417	28704827	-124
ssa04	28758303	usp53b	0 084119655	AX-87741778	28709862	28759440	0
ssa04	28771307	fabp2	0 078967398	AX-87117540	28770141	28772267	0
ssa04	28823088	gucy1b1	0 092162559	AX-87012837	28786905	28829263	0
ssa04	28837682	LOC106602731	0 097655608	AX-86915880	28835688	28849445	0
ssa04	28876531	map9	0 097655608	AX-87167049	28899848	28924098	23317
ssa04	28972198	LOC106602729	0 088164321	AX-87665528	28971473	28974892	0
ssa04	28994723	ufsp2	0 089763016	AX-87309970	28988902	29001122	0
ssa04	29002718	ankrd37	0 097336597	AX-87047159	29001107	29004798	0
ssa04	29033703	LOC106602727	0 097336597	AX-87629126	29032522	29039486	0
ssa04	29076424	cfap97	0 089213009	AX-87545454	29051877	29076340	-85
ssa04	29122960	si:ch211-197n1.2	0 087873381	AX-87848897	29080321	29167552	0
ssa04	29161414	si:ch211-197n1.2	0 087158249	AX-87856948	29080321	29167552	0
ssa04	29426609	sod3	0 09590969	AX-87275853	29423700	29426519	-91
ssa04	29794018	LOC106602803	0 09245913	AX-87807698	29782766	29786507	-7512
ssa04	29951676	cntrob	0 072918844	AX-87150219	29941903	29972928	0
ssa04	29963902	cntrob	0 072918844	AX-87809974	29941903	29972928	0
ssa04	29964073	cntrob	0 072918844	AX-87547531	29941903	29972928	0

ssa04	29964119	cntrob	0	072918844	AX-87292033	29941903	29972928	0
ssa04	29967885	cntrob	0	072918844	AX-87777807	29941903	29972928	0
ssa04	30361112	LOC106602818	0	070523305	AX-87410507	30336999	30371460	0
ssa04	30443456	LOC106602819	0	060498974	AX-87116052	30412984	30500786	0
ssa04	30444473	LOC106602819	0	070523305	AX-87042623	30412984	30500786	0
ssa04	30582707	klf5l	0	070523305	AX-87466823	30568266	30587609	0
ssa04	30796751	LOC106602825	0	05537997	AX-87848751	30797433	30812654	682
ssa04	31030348	LOC106602994	0	071751637	AX-87721906	31025074	31038179	0
ssa04	31059327	cd166	0	073448953	AX-87756194	31059050	31128291	0
ssa04	31379556	cblb	0	074977912	AX-87175283	31162251	31384357	0
ssa04	31382967	cblb	0	077594774	AX-87342988	31162251	31384357	0
ssa04	31735623	LOC106602832	0	073084275	AX-87035355	31706111	31768359	0
ssa04	31765670	LOC106602832	0	071691797	AX-87538818	31706111	31768359	0
ssa04	31925134	lis1b	0	078929904	AX-87816235	31886796	31940451	0
ssa04	31952291	lis1b	0	077616676	AX-87860464	31886796	31940451	-11841
ssa04	31971481	zpld1a	0	078929904	AX-87028459	31994118	32083690	22637
ssa04	32616295	alg8	0	054708412	AX-87805035	32555827	32599271	-17025
ssa04	32682670	LOC106602845	0	081475864	AX-87211620	32677604	32762655	0
ssa04	33063621	vtnb	0	062980873	AX-87497684	33072327	33119364	8706
ssa04	33667091	spns2	0	070434496	AX-87485555	33530483	33666182	-910
ssa04	33670610	spns2	0	070434496	AX-87108520	33530483	33666182	-4429
ssa04	33683578	ruvbl2	0	058159373	AX-87829995	33685033	33699947	1455
ssa04	33692035	ruvbl2	0	059600941	AX-86978571	33685033	33699947	0
ssa04	33741920	ftr83	0	067382649	AX-87172569	33729481	33741105	-816
ssa04	33749287	ftr82	0	067742941	AX-87159092	33748118	33753821	0
ssa04	33749477	ftr82	0	067742941	AX-87428133	33748118	33753821	0
ssa04	33765298	ift22	0	055882981	AX-86972675	33764128	33766580	0
ssa04	33801245	abcg4a	0	076121599	AX-87638858	33820384	33857673	19139
ssa04	33828409	abcg4a	0	064760584	AX-87439942	33820384	33857673	0
ssa04	33829044	abcg4a	0	06882226	AX-87750410	33820384	33857673	0
ssa04	33836173	abcg4a	0	064760584	AX-87374030	33820384	33857673	0
ssa04	36476077	LOC106602907	0	05595231	AX-87221831	36421468	36491618	0
ssa04	37639796	LOC100380851	0	054721191	AX-87558115	37627176	37653326	0
ssa04	37807876	LOC106602935	0	06282141	AX-87330163	37734839	37812304	0
ssa04	37821835	LOC106602935	0	060027106	AX-87026692	37734839	37812304	-9532
ssa04	37823873	LOC106602935	0	057130847	AX-87464468	37734839	37812304	-11570
ssa04	38011531	LOC106602941	0	07063184	AX-87206136	37987820	38012371	0
ssa04	38012590	LOC106602813	0	063328647	AX-87180040	38012365	38029870	0
ssa04	38030410	LOC106602813	0	07063184	AX-87747198	38012365	38029870	-541
ssa04	38031619	chm	0	07063184	AX-87257335	38030991	38064936	0
ssa04	38036670	chm	0	07063184	AX-87324854	38030991	38064936	0
ssa04	38111146	dacha	0	063208166	AX-86985263	38073181	38203116	0

ssa04	38112598	dacha	0 063208166	AX-87676734	38073181	38203116	0
ssa04	38113957	dacha	0 063208166	AX-87169479	38073181	38203116	0
ssa04	38144996	dacha	0 064507423	AX-87398350	38073181	38203116	0
ssa04	38145002	dacha	0 066477022	AX-87105904	38073181	38203116	0
ssa04	38146003	dacha	0 064353562	AX-87168157	38073181	38203116	0
ssa04	38597340	fhl1b	0 058256656	AX-87824211	38588018	38607969	0
ssa04	38669204	LOC106602967	0 055132039	AX-87066288	38674961	38696331	5757
ssa04	38799015	LOC106602974	0 053349921	AX-87067824	38798705	38802485	0
ssa04	38951289	LOC106602982	0 052721851	AX-87574166	38947155	38954021	0
ssa04	39552776	LOC106602814	0 05439964	AX-87322434	39388584	39552616	-161
ssa04	41858843	LOC106603103	0 06045212	AX-87781064	41843696	41857578	-1266
ssa04	47594051	LOC106603292	0 064425369	AX-87359853	47403212	47706787	0
ssa04	66298894	ntm	0 052816989	AX-87169334	65848577	66322048	0
ssa04	79640144	fam53c	0 059787253	AX-87273793	79629353	79641578	0
ssa04	80542991	LOC123742502	0 059023292	AX-87820873	80467764	80503684	-39308
ssa04	80730932	gria1b	0 053776257	AX-87511924	80730380	80830828	0
ssa05	9929413	LOC106592285	0 054696827	AX-87248277	9934265	10044520	4852
ssa05	10044268	LOC106592285	0 0684949	AX-87720181	9934265	10044520	0
ssa05	22894644	enox2	0 05646069	AX-87288792	22637541	22963523	0
ssa05	27324831	LOC106604533	0 054468932	AX-87366187	27260963	27413929	0
ssa05	33401529	LOC106604681	0 053165165	AX-87612531	33349563	33420416	0
ssa05	37155034	LOC106604765	0 053300897	AX-87117495	37153888	37156672	0
ssa05	37155349	LOC106604765	0 053300897	AX-87413112	37153888	37156672	0
ssa05	37156695	LOC106604765	0 053300897	AX-87475467	37153888	37156672	-24
ssa05	41245696	LOC106604922	0 072770041	AX-87178075	41236823	41254653	0
ssa05	41381975	LOC106604925	0 056239106	AX-87062377	41303029	41349340	-32636
ssa05	43928453	fmr1	0 053159763	AX-87516192	43930326	43940543	1873
ssa05	57672791	LOC106605266	0 061339345	AX-87596960	57611218	57644670	-28122
ssa05	57705088	LOC106605268	0 061339345	AX-87303305	57706219	57745185	1131
ssa05	57786032	LOC106605249	0 062263194	AX-87335842	57761716	57772560	-13473
ssa05	72124029	LOC106576962	0 055411881	AX-87810646	72117250	72125685	0
ssa05	72157585	LOC106605733	0 062219367	AX-87121339	72155245	72169426	0
ssa05	72162637	LOC106605733	0 062219367	AX-86993980	72155245	72169426	0
ssa05	72239702	LOC106576313	0 059124269	AX-87176024	72206569	72232719	-6984
ssa05	72252129	LOC106576679	0 066629762	AX-87500314	72247765	72294814	0
ssa05	72427382	LOC106605834	0 068093389	AX-87457114	72427232	72436124	0
ssa05	72443591	mprd	0 06797609	AX-87408084	72443359	72450360	0
ssa05	72466028	LOC106605835	0 0723225	AX-87655292	72457189	72471622	0
ssa05	72475608	LOC106605836	0 057979374	AX-87679489	72475141	72483640	0
ssa05	72475615	LOC106605836	0 06181784	AX-87342364	72475141	72483640	0
ssa05	72502666	LOC106605837	0 06181784	AX-86939144	72492932	72510872	0
ssa05	72562706	LOC106605839	0 076047042	AX-87782702	72530295	72595675	0

ssa05	72562706	LOC106605840	0 076047042	AX-87782702	72562095	72565337	0
ssa05	82848676	LOC123743190	0 062818114	AX-86980666	82855896	82857582	7220
ssa05	86137908	LOC106574054	0 062819861	AX-87697856	85987377	86168860	0
ssa06	13140513	LOC106606415	0 055028451	AX-87135076	13140808	13165244	295
ssa06	15505221	LOC106606440	0 053930871	AX-87385425	15489728	15525427	0
ssa06	15509377	LOC106606440	0 059449796	AX-87311245	15489728	15525427	0
ssa06	15526065	LOC106606440	0 059449796	AX-87518559	15489728	15525427	-639
ssa06	59473992	LOC123743469	0 05446305	AX-86943403	59462792	59468586	-5407
ssa06	59500221	LOC123743469	0 054898706	AX-87553009	59462792	59468586	-31636
ssa06	59500857	LOC123743469	0 065713138	AX-87745509	59462792	59468586	-32272
ssa06	72948359	LOC106607971	0 056125204	AX-87340608	72932356	72943574	-4786
ssa06	72965488	ostm1	0 056125204	AX-87299159	72972259	72975953	6771
ssa06	82068760	LOC106608201	0 054854822	AX-87558497	82051610	82084555	0
ssa07	14663264	LOC106608688	0 054161469	AX-87390119	13861866	14968763	0
ssa07	22945670	LOC106608895	0 056538826	AX-87544891	22946309	22960273	639
ssa07	22950064	LOC106608895	0 066708538	AX-87053593	22946309	22960273	0
ssa07	22962361	LOC106608818	0 07643452	AX-87086126	22961485	22963410	0
ssa07	22962866	LOC106608818	0 07643452	AX-87352395	22961485	22963410	0
ssa07	22963332	LOC106608818	0 072992574	AX-87421987	22961485	22963410	0
ssa07	22964213	LOC106608817	0 074168083	AX-87471258	22963600	23009267	0
ssa07	22964519	LOC106608817	0 052711184	AX-87274445	22963600	23009267	0
ssa07	22964632	LOC106608817	0 052711184	AX-87821305	22963600	23009267	0
ssa07	23000893	LOC106608817	0 075258005	AX-87729558	22963600	23009267	0
ssa07	23011095	LOC106608817	0 05580894	AX-87440648	22963600	23009267	-1829
ssa07	23022156	LOC106608819	0 069793048	AX-87732109	23020948	23034140	0
ssa07	38554071	LOC106609224	0 075578274	AX-87543412	38498553	38554444	0
ssa07	46666019	LOC106609430	0 057428185	AX-87137403	46656416	46665303	-717
ssa07	54506852	LOC106609762	0 054239037	AX-87824599	54366701	54860055	0
ssa08	1194422	LOC106610035	0 086870414	AX-87522445	1189134	1194707	0
ssa08	8664525	LOC106610073	0 054088677	AX-87353624	8347797	9191850	0
ssa08	8664525	LOC106610072	0 054088677	AX-87353624	8375505	8812961	0
ssa08	8664525	LOC106610156	0 054088677	AX-87353624	8653907	8668068	0
ssa09	6858388	rcan3	0 0540856	AX-87527958	6766169	6817677	-40712
ssa09	11390062	LOC106610720	0 056973892	AX-86973401	11399012	11424820	8950
ssa09	13406673	kctd3	0 055991338	AX-87406506	13396108	13417424	0
ssa09	23303377	LOC123744603	0 0561555	AX-87790634	23252105	23314426	0
ssa09	23304141	LOC123744603	0 056577632	AX-87059227	23252105	23314426	0
ssa09	23305258	LOC123744603	0 057137993	AX-87065031	23252105	23314426	0
ssa09	23489588	LOC106610987	0 069623072	AX-87316072	23478223	23548629	0
ssa09	23914069	six4a	0 064837596	AX-87799966	23909119	23916471	0
ssa09	23992833	slc38a6	0 053393341	AX-87655906	23981707	23994710	0
ssa09	24102226	LOC106610969	0 061158505	AX-87357627	24085299	24139342	0

ssa09	24102268	LOC106610969	0 061762498	AX-87458123	24085299	24139342	0
ssa09	24102470	LOC106610969	0 058985658	AX-87083371	24085299	24139342	0
ssa09	24105899	LOC106610969	0 064273275	AX-86959853	24085299	24139342	0
ssa09	24508977	LOC106610918	0 078108182	AX-87609372	24421752	24514264	0
ssa09	24720914	LOC106610964	0 060984748	AX-87438780	24733770	24770954	12856
ssa09	24746794	LOC106610964	0 067393358	AX-87452777	24733770	24770954	0
ssa09	28121035	LOC106611108	0 055556895	AX-87500037	27962639	28083037	-37999
ssa09	28328255	LOC106611155	0 053318762	AX-87772374	28361549	28466058	33294
ssa09	28331243	LOC106611155	0 053318762	AX-87639391	28361549	28466058	30306
ssa09	28364206	LOC106611155	0 053318762	AX-87239594	28361549	28466058	0
ssa09	30543457	LOC106611182	0 054549472	AX-87783557	30543083	30584002	0
ssa09	30895454	trnaa-ugc	0 059835267	AX-87867546	30882875	30882950	-12505
ssa09	31101552	ccdc175	0 059463805	AX-87119577	31116854	31130016	15302
ssa09	31210807	daam1a	0 065222804	AX-87549314	31152151	31232364	0
ssa09	31469251	LOC100194743	0 053972681	AX-87290282	31459330	31496118	0
ssa09	31547024	LOC123724122	0 057121542	AX-87155042	31539383	31542867	-4158
ssa09	31547054	LOC123724122	0 057121542	AX-87712949	31539383	31542867	-4188
ssa09	31555583	LOC106611222	0 057121542	AX-87253902	31555030	31557522	0
ssa09	31590040	LOC106611199	0 057121542	AX-87839175	31579196	31581589	-8452
ssa09	32965019	LOC106611226	0 056362189	AX-87763779	32956287	32970793	0
ssa09	33039293	LOC106611229	0 055516361	AX-86946145	33010133	33048838	0
ssa09	33046524	LOC106611229	0 053895011	AX-86918060	33010133	33048838	0
ssa09	40325483	oct2	0 056331764	AX-87732396	40312965	40332927	0
ssa09	40332760	oct2	0 055612946	AX-87747335	40312965	40332927	0
ssa09	40348966	dusp23b	0 056060453	AX-87590443	40348098	40349977	0
ssa09	40469135	LOC106611355	0 053294792	AX-87681331	40408742	40472674	0
ssa09	40471689	LOC106611355	0 053294792	AX-87420149	40408742	40472674	0
ssa09	55444988	LOC106611494	0 055244719	AX-87360289	55412500	55457484	0
ssa09	56083070	LOC106611507	0 052802349	AX-87160228	56119487	56807249	36417
ssa09	56083837	LOC106611507	0 052802349	AX-87149150	56119487	56807249	35650
ssa09	56099161	LOC106611507	0 052802349	AX-87499564	56119487	56807249	20326
ssa09	56100278	LOC106611507	0 052802349	AX-87124738	56119487	56807249	19209
ssa09	56141798	LOC106611507	0 052802349	AX-87625276	56119487	56807249	0
ssa09	56144323	LOC106611507	0 052802349	AX-87514534	56119487	56807249	0
ssa09	56161528	LOC106611507	0 052802349	AX-87556776	56119487	56807249	0
ssa09	56162268	LOC106611507	0 052802349	AX-87852026	56119487	56807249	0
ssa09	56167247	LOC106611507	0 052802349	AX-87419220	56119487	56807249	0
ssa09	56231601	LOC106611507	0 052741154	AX-87314323	56119487	56807249	0
ssa09	56234935	LOC106611507	0 057912228	AX-87535416	56119487	56807249	0
ssa09	56294156	LOC106611507	0 052741154	AX-87427747	56119487	56807249	0
ssa09	56296270	LOC106611507	0 052741154	AX-87062035	56119487	56807249	0
ssa09	56322060	LOC106611507	0 057912228	AX-87154991	56119487	56807249	0

ssa09	56363496	LOC106611507	0 053933628	AX-87332120	56119487	56807249	0
ssa09	56375806	LOC106611507	0 066239697	AX-87003468	56119487	56807249	0
ssa09	56382679	LOC106611507	0 056931583	AX-87191460	56119487	56807249	0
ssa09	56603909	LOC106611507	0 05340009	AX-87422271	56119487	56807249	0
ssa09	56606207	LOC106611507	0 05340009	AX-87016481	56119487	56807249	0
ssa09	56738923	LOC106611507	0 055929776	AX-87762885	56119487	56807249	0
ssa09	57231649	LOC106611509	0 060013907	AX-87179587	57225288	57315572	0
ssa09	57232384	LOC106611509	0 059661551	AX-87805508	57225288	57315572	0
ssa09	57773251	LOC106611510	0 057304632	AX-87536263	57724684	57850862	0
ssa09	57922165	LOC106611497	0 063476294	AX-86990597	57858432	57963514	0
ssa09	57932005	LOC106611497	0 063476294	AX-87281689	57858432	57963514	0
ssa09	57972105	LOC106611512	0 063476294	AX-87032610	57977621	57981717	5516
ssa09	58120291	atp1b4	0 062310396	AX-87729425	58120208	58127192	0
ssa09	65471667	LOC106611628	0 054274616	AX-87867689	65464049	65565018	0
ssa09	83427618	gpc4	0 058889196	AX-87158700	83412167	83486735	0
ssa09	83673349	LOC106612114	0 069791126	AX-87856016	83661182	83676885	0
ssa09	84086818	LOC106612098	0 062486573	AX-87865565	84051188	84091099	0
ssa09	84091855	LOC106612097	0 05752343	AX-87729375	84092295	84096230	440
ssa09	84092951	LOC106612097	0 056136216	AX-87871023	84092295	84096230	0
ssa09	84101220	LOC106612096	0 054729157	AX-87831286	84096364	84101499	0
ssa09	84170176	LOC106612090	0 053140215	AX-87614568	84162126	84170260	0
ssa09	84187035	LOC106612088	0 054721076	AX-87496771	84185834	84196774	0
ssa09	86577734	LOC100380730	0 059531895	AX-87634282	86553905	86587548	0
ssa09	88673627	LOC123724131	0 054602432	AX-87759450	88667018	88673649	0
ssa09	88946877	LOC106612144	0 055599538	AX-87614203	88890086	88953935	0
ssa09	89028412	LOC106612141	0 060222978	AX-87635388	88972519	89096422	0
ssa09	89071734	LOC106612141	0 055599538	AX-87348015	88972519	89096422	0
ssa09	89072686	LOC106612141	0 055599538	AX-87361974	88972519	89096422	0
ssa09	90794534	hmmr	0 058754566	AX-87634332	90788584	90805590	0
ssa09	91304126	LOC106612218	0 07321226	AX-87638466	91303062	91339103	0
ssa09	122494741	LOC106612600	0 053768449	AX-87016566	122494029	122500340	0
ssa09	122936688	LOC106612575	0 060131886	AX-87402675	122921773	122951657	0
ssa09	122966094	LOC106612580	0 058905664	AX-87564261	122966391	122969936	297
ssa09	128012919	LOC106612862	0 060467179	AX-87789883	127849185	128192219	0
ssa09	128017110	LOC106612862	0 058984658	AX-87376473	127849185	128192219	0
ssa09	134411643	LOC106612976	0 053644246	AX-87681095	134411623	134414170	0
ssa09	134411885	LOC106612976	0 053644246	AX-87385639	134411623	134414170	0
ssa09	134420582	LOC106612976	0 053644246	AX-86911933	134411623	134414170	-6413
ssa09	141711560	LOC106613173	0 066246102	AX-87350427	141745552	141747855	33992
ssa09	141740678	LOC106613173	0 061001797	AX-86939204	141745552	141747855	4874
ssa10	10596157	actl6a	0 056522033	AX-87379385	10580252	10598090	0
ssa10	24084357	LOC106613832	0 056411816	AX-87685243	24081926	24085828	0

ssa10	26234691	LOC106613881	0 053804451	AX-87066418	26167154	26343009	0
ssa10	26243590	LOC106613881	0 053804451	AX-87393350	26167154	26343009	0
ssa10	26245955	LOC106613881	0 053804451	AX-86924571	26167154	26343009	0
ssa10	26271625	LOC106613881	0 053804451	AX-87138917	26167154	26343009	0
ssa10	26272141	LOC106613881	0 053804451	AX-86986123	26167154	26343009	0
ssa10	31687745	mdp1	0 070413292	AX-87523367	31683269	31686987	-759
ssa10	31697578	LOC106560237	0 072751341	AX-87631579	31688664	31707805	0
ssa10	31697745	LOC106560237	0 072751341	AX-86990461	31688664	31707805	0
ssa10	31730820	LOC100194696	0 075903124	AX-87450305	31730416	31734575	0
ssa10	31764184	LOC106560238	0 068213917	AX-87832958	31751825	31765121	0
ssa10	31766949	LOC106560239	0 069348742	AX-87077642	31766606	31777696	0
ssa10	74416804	LOC106561060	0 055564597	AX-87415315	74294538	74441471	0
ssa10	74439621	LOC106561060	0 053670633	AX-87466006	74294538	74441471	0
ssa10	77305121	LOC106561032	0 059763589	AX-86928183	77099384	77362422	0
ssa10	83402360	LOC106560932	0 058079809	AX-87508099	83402359	83422381	0
ssa10	83406808	LOC106560932	0 073729592	AX-87249920	83402359	83422381	0
ssa10	88922109	LOC106560807	0 057845432	AX-87442601	88908072	88921429	-681
ssa10	89380084	LOC106560801	0 072372663	AX-87813108	89425283	89435526	45199
ssa10	89473116	LOC106560802	0 054159945	AX-87241114	89434347	89453872	-19245
ssa10	94173215	LOC123724563	0 059265802	AX-87086172	94158334	94196067	0
ssa10	94210384	LOC123723687	0 058255718	AX-87576121	94200074	94227204	0
ssa10	94227205	LOC123723687	0 058255718	AX-86979974	94200074	94227204	-2
ssa10	94349659	LOC123724472	0 053357606	AX-87227205	94328543	94380101	0
ssa10	94425450	LOC123724476	0 070641489	AX-87312237	94422409	94428503	0
ssa10	94506432	LOC123724477	0 053620062	AX-87494883	94506584	94545969	152
ssa10	96087862	LOC106561335	0 060493223	AX-87258041	95817815	96104031	0
ssa10	96097481	LOC106561335	0 056774485	AX-87081342	95817815	96104031	0
ssa10	96099953	LOC106561335	0 059221326	AX-86929680	95817815	96104031	0
ssa10	96119760	LOC106561332	0 062434166	AX-87622929	96114132	96137812	0
ssa10	96142705	LOC106561333	0 063159797	AX-87848891	96137873	96151970	0
ssa10	96309528	LOC106561328	0 05809179	AX-86952897	96301253	96324987	0
ssa10	97858161	LOC106561308	0 054322306	AX-87684677	97817563	97857514	-648
ssa10	102755849	LOC123724507	0 057500172	AX-87010713	102691957	102797174	0
ssa10	102777071	LOC123724507	0 068832544	AX-87191336	102691957	102797174	0
ssa10	102790142	LOC123724507	0 071780007	AX-87343208	102691957	102797174	0
ssa10	102790197	LOC123724507	0 06802019	AX-87490647	102691957	102797174	0
ssa10	102818567	LOC123724507	0 069444311	AX-87765699	102691957	102797174	-21394
ssa10	104171421	LOC106561406	0 056468614	AX-87007124	104131354	104249960	0
ssa10	104207517	LOC106561406	0 054250185	AX-87666842	104131354	104249960	0
ssa10	104225496	LOC106561406	0 054250185	AX-87212894	104131354	104249960	0
ssa10	104281204	LOC106561405	0 053968275	AX-86972906	104279329	104295187	0
ssa10	104291722	LOC106561405	0 058131157	AX-87304026	104279329	104295187	0

ssa10	104321325	LOC106561402	0 058682664	AX-87286039	104318087	104330428	0
ssa10	104321403	LOC106561402	0 059108232	AX-87053612	104318087	104330428	0
ssa10	104342114	LOC106561401	0 061633576	AX-86963174	104347407	104383543	5293
ssa10	104356261	LOC106561401	0 057143629	AX-87231661	104347407	104383543	0
ssa10	104367168	LOC106561401	0 057143629	AX-87135362	104347407	104383543	0
ssa10	104428988	LOC106561400	0 057205254	AX-86910681	104425458	104523205	0
ssa10	109361507	LOC106561490	0 055222526	AX-87122316	109356818	109376207	0
ssa10	110987783	LOC106561587	0 057600905	AX-87048174	111018576	111020320	30793
ssa10	111184487	lsm14aa	0 054092615	AX-87491062	111166667	111184806	0
ssa10	111186212	LOC106561532	0 054092615	AX-87669590	111185770	111201796	0
ssa10	112296172	cep41	0 056783398	AX-86972579	112295695	112303624	0
ssa10	112392229	poc1b	0 05674429	AX-87743654	112303690	112373550	-18680
ssa10	113836604	kif21a	0 061335587	AX-87559368	113761112	113838086	0
ssa10	114555258	LOC106561611	0 071562984	AX-87671750	114554837	114560652	0
ssa10	114706753	LOC106561615	0 067805882	AX-87729737	114586600	114749624	0
ssa10	114760790	LOC106561616	0 066959697	AX-87322421	114760347	114861603	0
ssa10	114774857	LOC106561616	0 061919126	AX-87344901	114760347	114861603	0
ssa10	117842208	LOC106561692	0 055561856	AX-86908906	117829395	117876354	0
ssa10	122166075	LOC106561787	0 061504662	AX-87777256	122127778	122167466	0
ssa11	10387218	LOC106561978	0 056764951	AX-87686326	10358392	10364411	-22808
ssa11	10397862	si:dkey-56m19.5	0 056764951	AX-87000028	10417117	10423479	19255
ssa11	10417940	si:dkey-56m19.5	0 058331832	AX-87800156	10417117	10423479	0
ssa11	10435835	LOC106561980	0 059167143	AX-87645133	10439943	10457514	4108
ssa11	10451889	LOC106561980	0 053351973	AX-87423848	10439943	10457514	0
ssa11	10454152	LOC106561980	0 055938817	AX-87470809	10439943	10457514	0
ssa11	10471650	LOC106561981	0 064627732	AX-87677219	10458340	10477094	0
ssa11	10473667	LOC106561981	0 06047124	AX-87448397	10458340	10477094	0
ssa11	19523825	zfhx3b	0 09153121	AX-87849470	19367414	19628443	0
ssa11	27188852	LOC106562373	0 057985863	AX-87168112	27124046	27323382	0
ssa11	27205339	LOC106562373	0 052831931	AX-87071596	27124046	27323382	0
ssa11	27735781	LOC106562384	0 07044363	AX-87829306	27655509	27737240	0
ssa11	30221808	LOC106562421	0 053179922	AX-87499038	30224092	30316917	2284
ssa11	30224658	LOC106562421	0 057046236	AX-87439068	30224092	30316917	0
ssa11	30406167	LOC106562419	0 062793472	AX-87352038	30392192	30414023	0
ssa11	30688868	LOC106562413	0 053520436	AX-86942176	30569553	30690923	0
ssa11	68521122	LOC106562972	0 052874321	AX-87317924	68220625	68480415	-40708
ssa11	68526938	LOC106562972	0 052874321	AX-87489563	68220625	68480415	-46524
ssa11	78742628	LOC106563327	0 053379609	AX-87230002	78741089	78754068	0
ssa11	78742628	LOC123725189	0 053379609	AX-87230002	78741900	78743168	0
ssa11	78805487	LOC106563371	0 057493387	AX-87651664	78817070	78819009	11583
ssa11	78870872	LOC106563326	0 05799153	AX-87029641	78899811	78958586	28939
ssa11	80266247	LOC106563286	0 059000804	AX-87357559	80253693	80274223	0

ssa11	80267726	LOC106563286	0 055415211	AX-86905999	80253693	80274223	0
ssa11	80272700	LOC106563286	0 059000804	AX-87471802	80253693	80274223	0
ssa11	80273475	LOC106563286	0 059000804	AX-86906759	80253693	80274223	0
ssa11	80274778	LOC106563286	0 059000804	AX-87297888	80253693	80274223	-556
ssa11	80277385	LOC106563286	0 059000804	AX-87342531	80253693	80274223	-3163
ssa11	80277800	LOC106563286	0 055547755	AX-87084889	80253693	80274223	-3578
ssa11	80278627	LOC106563286	0 059000804	AX-87026892	80253693	80274223	-4405
ssa11	80285377	LOC106563286	0 055547755	AX-87437765	80253693	80274223	-11155
ssa11	80319166	LOC106563364	0 054974145	AX-87174575	80326873	80327766	7707
ssa11	80346563	ddx54	0 055853675	AX-87833843	80336689	80346817	0
ssa11	80353598	ddx54	0 069440375	AX-86941576	80336689	80346817	-6782
ssa11	80366279	LOC106563285	0 062278954	AX-87227297	80370846	80393937	4567
ssa11	80366507	LOC106563285	0 062278954	AX-87105592	80370846	80393937	4339
ssa11	80377330	LOC106563285	0 053311906	AX-87178624	80370846	80393937	0
ssa11	80382152	LOC106563285	0 052957203	AX-87782426	80370846	80393937	0
ssa11	80394518	LOC106563284	0 052957203	AX-87667966	80394406	80449252	0
ssa11	80396186	LOC106563284	0 052957203	AX-87109486	80394406	80449252	0
ssa11	80434716	LOC106563284	0 053311906	AX-87028609	80394406	80449252	0
ssa11	80444390	LOC106563284	0 068518558	AX-87714365	80394406	80449252	0
ssa11	80444613	LOC106563284	0 068518558	AX-87166441	80394406	80449252	0
ssa11	80445719	LOC106563284	0 068518558	AX-87806177	80394406	80449252	0
ssa11	80472627	LOC106563283	0 072778442	AX-86944012	80465178	80470006	-2622
ssa11	80554994	ass1	0 059700738	AX-87690606	80526547	80560455	0
ssa11	80567198	surf4	0 058479892	AX-87168566	80563673	80570962	0
ssa11	80674991	LOC106563274	0 081841071	AX-87366026	80601934	80685624	0
ssa11	80678897	LOC106563274	0 054512102	AX-87748741	80601934	80685624	0
ssa11	80683640	LOC106563274	0 057164929	AX-87577797	80601934	80685624	0
ssa11	80887540	LOC106563272	0 056584051	AX-87307668	80792474	80991740	0
ssa11	80967044	LOC106563272	0 058825067	AX-87219300	80792474	80991740	0
ssa11	80967118	LOC106563272	0 058825067	AX-87148864	80792474	80991740	0
ssa11	80967806	LOC106563272	0 058825067	AX-87692877	80792474	80991740	0
ssa11	81513076	LOC106563270	0 053277966	AX-87254262	81433308	81558089	0
ssa11	81957595	LOC106563268	0 061181692	AX-86961934	81562761	82096555	0
ssa11	81963438	LOC106563268	0 056964868	AX-86928863	81562761	82096555	0
ssa11	83772050	LOC106563250	0 053323985	AX-87456479	83762608	83770952	-1099
ssa11	84145823	arrdc1b	0 05495466	AX-87056189	84104577	84161073	0
ssa11	84243607	enp2	0 058321976	AX-87339150	84198640	84246491	0
ssa11	84635836	LOC106563231	0 053312786	AX-87808609	84613259	84641296	0
ssa11	84641042	LOC106563231	0 053312786	AX-87564650	84613259	84641296	0
ssa11	85408198	LOC106563222	0 065484726	AX-87699325	85348057	85412825	0
ssa11	85412642	LOC106563222	0 062045009	AX-87562842	85348057	85412825	0
ssa11	85415083	LOC106563222	0 062045009	AX-87771662	85348057	85412825	-2259

ssa11	85415919	LOC106563222	0	062045009	AX-87858079	85348057	85412825	-3095
ssa11	85422531	LOC106563222	0	062045009	AX-87419702	85348057	85412825	-9707
ssa11	85461460	LOC106563222	0	068303669	AX-87736302	85348057	85412825	-48636
ssa11	85475914	LOC106563221	0	073769763	AX-87548620	85525875	85568414	49961
ssa11	85523295	LOC106563221	0	063079497	AX-87443769	85525875	85568414	2580
ssa11	85587363	LOC106563221	0	06293028	AX-87528634	85525875	85568414	-18950
ssa11	85588106	LOC106563221	0	06293028	AX-87227421	85525875	85568414	-19693
ssa11	85623194	LOC106563220	0	060430771	AX-86941869	85628000	85653823	4806
ssa11	85647757	LOC106563220	0	071521427	AX-87119781	85628000	85653823	0
ssa11	85650668	LOC106563220	0	073286683	AX-87811472	85628000	85653823	0
ssa11	85650685	LOC106563220	0	073286683	AX-87154009	85628000	85653823	0
ssa11	85651885	LOC106563220	0	076177813	AX-87638449	85628000	85653823	0
ssa11	85651885	LOC123725192	0	076177813	AX-87638449	85650955	85652332	0
ssa11	85664472	bnip3l	0	073286683	AX-87006871	85662609	85677002	0
ssa11	85679606	LOC106563219	0	059743852	AX-87019397	85677341	85695470	0
ssa11	85694856	LOC106563219	0	073286683	AX-87009326	85677341	85695470	0
ssa11	85712648	LOC106563219	0	073286683	AX-86979805	85677341	85695470	-17179
ssa11	85712802	LOC106563219	0	073286683	AX-87165237	85677341	85695470	-17333
ssa11	85713159	LOC106563219	0	073286683	AX-86966649	85677341	85695470	-17690
ssa11	87084373	LOC106563207	0	053736948	AX-87663772	87039689	87094603	0
ssa11	87185854	5ntc	0	057846361	AX-87817332	87191221	87202077	5367
ssa11	89584318	LOC106563513	0	055252117	AX-87857958	89542949	89590965	0
ssa11	93822415	LOC123725259	0	056466109	AX-87021203	93807005	93807705	-14711
ssa11	93839650	LOC123725259	0	05725657	AX-87815877	93807005	93807705	-31946
ssa11	93849693	LOC123725259	0	054384679	AX-87400054	93807005	93807705	-41989
ssa11	94272592	pygma	0	064674393	AX-86993080	94257994	94274844	0
ssa11	94917156	dpp3	0	053181481	AX-87595807	94916908	94929608	0
ssa11	95596035	LOC106563717	0	052791696	AX-87578072	95590321	95598059	0
ssa12	9746109	LOC106564128	0	052776239	AX-87317638	9739676	9746605	0
ssa12	9774428	LOC106564127	0	06164514	AX-87494623	9761256	9772584	-1845
ssa12	9908758	LOC106594003	0	066085942	AX-87102335	9893300	9898862	-9897
ssa12	9956566	LOC106593629	0	060099626	AX-87799423	9951177	9957162	0
ssa12	9956680	LOC106593629	0	05875316	AX-87139417	9951177	9957162	0
ssa12	27925677	LOC106564712	0	054093049	AX-87556246	27898818	28056374	0
ssa12	27925958	LOC106564712	0	060219646	AX-87783816	27898818	28056374	0
ssa12	27959622	LOC106564712	0	064753346	AX-87279388	27898818	28056374	0
ssa12	27959964	LOC106564712	0	061738386	AX-87024754	27898818	28056374	0
ssa12	29094502	LOC106595778	0	053241979	AX-87414293	29077354	29094978	0
ssa12	43320351	LOC106565072	0	054730729	AX-87362099	43319269	43364949	0
ssa12	43393689	LOC106565073	0	05848783	AX-87725117	43381068	43395339	0
ssa12	44430903	LOC106565320	0	054288906	AX-87844995	44473604	44475577	42701
ssa12	46402564	LOC106565271	0	064148379	AX-87364421	46389098	46498088	0

ssa12	49895736	LOC106565213	0	067931183	AX-87448079	49761833	50021816	0
ssa12	49897360	LOC106565213	0	067931183	AX-87151595	49761833	50021816	0
ssa12	49899431	LOC106565213	0	067931183	AX-87632609	49761833	50021816	0
ssa12	49983112	LOC106565213	0	053300359	AX-87833068	49761833	50021816	0
ssa12	57059251	pbrm1l	0	053228015	AX-87022153	57030980	57068043	0
ssa12	57934300	LOC106565353	0	057278071	AX-87793091	57924523	57939314	0
ssa12	57942346	zgc:172271	0	057278071	AX-87061476	57942059	57949759	0
ssa12	57945046	zgc:172271	0	057988994	AX-87597312	57942059	57949759	0
ssa12	57945428	zgc:172271	0	058314945	AX-87626195	57942059	57949759	0
ssa12	57966789	LOC106565356	0	057887347	AX-87560733	57965412	57967306	0
ssa12	58027597	frs3	0	053661977	AX-87413935	57981953	57994421	-33177
ssa12	58058015	slc6a11a	0	076561758	AX-87732604	58087233	58108997	29218
ssa12	58089985	slc6a11a	0	05564705	AX-87309921	58087233	58108997	0
ssa12	58090100	slc6a11a	0	05564705	AX-86963766	58087233	58108997	0
ssa12	58139626	eno1b	0	055507167	AX-87025413	58139280	58149399	0
ssa12	58164282	LOC106565362	0	06980862	AX-86911035	58163625	58171597	0
ssa12	58212531	iho1	0	078985746	AX-87538498	58210234	58214293	0
ssa12	58222264	kbtbd12	0	064403555	AX-87802130	58214679	58223617	0
ssa12	58228021	slc26a6l	0	059901488	AX-87346407	58228235	58237817	214
ssa12	58234999	slc26a6l	0	059492373	AX-86955067	58228235	58237817	0
ssa12	58238746	LOC106565486	0	064403555	AX-87212705	58237958	58255748	0
ssa12	58349200	kiaa1328	0	059229347	AX-87272871	58336351	58391510	0
ssa12	58398412	LOC106565371	0	067434349	AX-87655383	58391830	58495592	0
ssa12	58448478	LOC106565371	0	074148854	AX-87206573	58391830	58495592	0
ssa12	59267727	LOC106565372	0	053856667	AX-87228098	59261936	59288375	0
ssa12	59302914	cita	0	05280502	AX-87844366	59297545	59349281	0
ssa12	59514258	LOC106565381	0	057426302	AX-87567541	59496809	59537572	0
ssa12	59521183	LOC106565381	0	057426302	AX-87398092	59496809	59537572	0
ssa12	64110520	LOC106565583	0	053934634	AX-87207869	64023045	64146522	0
ssa12	67445508	LOC106565661	0	060765005	AX-87455876	67421299	67491578	0
ssa12	67557168	tmcc2	0	056275505	AX-87296251	67559073	67565975	1905
ssa12	68082173	LOC106565639	0	058352547	AX-87169530	68081364	68095141	0
ssa12	68099625	ap5b1	0	058399495	AX-86923993	68103302	68107106	3677
ssa12	91749975	LOC106566150	0	062012592	AX-86942774	91701749	91831889	0
ssa12	99107785	LOC106566244	0	052852021	AX-87542540	99117040	99146964	9255
ssa13	14432131	LOC106566493	0	055358938	AX-87258339	14297795	14463043	0
ssa13	14517091	slc6a8	0	072910508	AX-87803733	14517058	14543691	0
ssa13	23630893	LOC106566750	0	055630727	AX-87484312	23634282	23642704	3389
ssa13	23647728	LOC106566749	0	052921411	AX-87631577	23647982	23655581	254
ssa13	23647955	LOC106566749	0	052921411	AX-87275911	23647982	23655581	27
ssa13	25175148	LOC106566728	0	053880836	AX-87063417	25157766	25265045	0
ssa13	28207009	LOC106566803	0	05290485	AX-87078214	28210944	28401882	3935

ssa13	30534182	LOC106566880	0 055271628	AX-87526630	30524207	30595234	0
ssa13	30628652	cfap74	0 054402303	AX-87530037	30610986	30660681	0
ssa13	38285785	LOC106567150	0 055220125	AX-87447910	38210947	38403929	0
ssa13	41539557	LOC106567065	0 054115436	AX-87316453	41466681	41538568	-990
ssa13	49971979	LOC106567372	0 054937608	AX-87242531	49948487	49969201	-2779
ssa13	50155615	LOC106567375	0 055286369	AX-87615884	50089938	50196928	0
ssa13	50241470	LOC106567377	0 053133959	AX-87050846	50277057	50391223	35587
ssa13	50244488	LOC106567377	0 053133959	AX-87588461	50277057	50391223	32569
ssa13	50286483	LOC106567377	0 053133959	AX-87416285	50277057	50391223	0
ssa13	50399042	LOC106567378	0 053774441	AX-87107265	50397154	50407244	0
ssa13	50840074	LOC106567382	0 068401272	AX-87413230	50825647	50841164	0
ssa13	54653265	egr1	0 077572505	AX-87443823	54657254	54658185	3989
ssa13	54656725	egr1	0 057525406	AX-87296843	54657254	54658185	529
ssa13	54670399	egr1	0 056030734	AX-87564712	54657254	54658185	-12215
ssa13	54911249	LOC106567433	0 056053399	AX-87207959	54920000	55055115	8751
ssa13	55281223	LOC106567439	0 06363963	AX-87168144	55260520	55289296	0
ssa13	75233608	LOC106568014	0 067521728	AX-87575107	75252261	75257406	18653
ssa13	75252912	LOC106568014	0 070377546	AX-87486922	75252261	75257406	0
ssa13	75305968	LOC106568009	0 067521728	AX-86933711	75305466	75324663	0
ssa13	75339111	LOC106568008	0 060837867	AX-86996802	75332330	75345898	0
ssa13	75366681	LOC106568007	0 065066765	AX-87751251	75354940	75456306	0
ssa13	75370134	LOC106568007	0 056191418	AX-87133830	75354940	75456306	0
ssa13	75392242	LOC106568007	0 053755939	AX-87073797	75354940	75456306	0
ssa13	75489146	LOC106568070	0 05744568	AX-87286509	75460894	75488231	-916
ssa13	75497725	LOC106568006	0 056040143	AX-87611091	75504855	75561443	7130
ssa13	78343717	LOC106567969	0 053274752	AX-87005151	78279049	78347650	0
ssa13	78497027	LOC106567964	0 071364352	AX-87625260	78499721	78510500	2694
ssa13	78605835	med13a	0 066185429	AX-87633189	78601952	78721308	0
ssa13	78615035	med13a	0 066273528	AX-87388990	78601952	78721308	0
ssa13	78643781	med13a	0 060967876	AX-87500446	78601952	78721308	0
ssa13	78649523	med13a	0 063295951	AX-87739952	78601952	78721308	0
ssa13	78692273	med13a	0 06364526	AX-87218719	78601952	78721308	0
ssa13	78692356	med13a	0 06364526	AX-87698721	78601952	78721308	0
ssa13	78692801	med13a	0 062447397	AX-87149536	78601952	78721308	0
ssa13	78738555	ints2	0 066093403	AX-86937959	78722419	78744926	0
ssa13	78740070	ints2	0 066093403	AX-87480017	78722419	78744926	0
ssa13	78744112	ints2	0 066690712	AX-87230867	78722419	78744926	0
ssa13	79527746	cxadr	0 056125452	AX-87745803	79535068	79606884	7322
ssa13	79535629	cxadr	0 056125452	AX-87418013	79535068	79606884	0
ssa13	79540168	cxadr	0 056125452	AX-87412889	79535068	79606884	0
ssa13	79550373	cxadr	0 056125452	AX-87700758	79535068	79606884	0
ssa13	106531058	stbd1	0 057245521	AX-87194612	106530692	106541427	0

ssa13	106531232	stbd1	0 057245521	AX-87301538	106530692	106541427	0
ssa13	106531278	stbd1	0 057245521	AX-87729440	106530692	106541427	0
ssa13	106795863	ccni	0 053030693	AX-87290074	106774412	106797348	0
ssa13	108191873	LOC106568540	0 053906407	AX-87045825	107903783	108331854	0
ssa14	12429301	LOC106568966	0 054901125	AX-87719459	12432035	12454655	2734
ssa14	12437267	LOC106568966	0 055570648	AX-87858650	12432035	12454655	0
ssa14	12437926	LOC106568966	0 055570648	AX-87599428	12432035	12454655	0
ssa14	22765252	LOC106569075	0 069732493	AX-87765987	22683290	22810692	0
ssa14	22768182	LOC106569075	0 066164958	AX-87036874	22683290	22810692	0
ssa14	22955401	LOC106569080	0 053740617	AX-87810484	22925659	22927660	-27742
ssa14	23906420	tmem53	0 057501389	AX-87257888	23945216	23946881	38796
ssa14	23910338	tmem53	0 057501389	AX-87000239	23945216	23946881	34878
ssa14	24608815	cdh12a	0 064882515	AX-87463327	24654857	24683989	46042
ssa14	24609400	cdh12a	0 064882515	AX-87036015	24654857	24683989	45457
ssa14	25211028	LOC106569231	0 060262101	AX-87796929	25199052	25221637	0
ssa14	32069222	LOC106569331	0 055363362	AX-87666227	32050339	32070479	0
ssa14	32069552	LOC106569331	0 053605784	AX-86962209	32050339	32070479	0
ssa14	43696597	LOC106569597	0 078450544	AX-87796896	43657594	43699102	0
ssa14	47776338	LOC106569492	0 06574278	AX-87735164	47764781	47941831	0
ssa14	67205160	LOC106569877	0 052765311	AX-87265836	67211664	67213837	6504
ssa14	67205494	LOC106569877	0 052765311	AX-87387084	67211664	67213837	6170
ssa14	67286949	LOC106569874	0 055122811	AX-87492430	67279851	67286725	-225
ssa14	67294159	LOC106569874	0 054678405	AX-87565669	67279851	67286725	-7435
ssa14	72579312	LOC106570268	0 053867387	AX-87585590	72561986	72611188	0
ssa14	72607201	LOC106570268	0 053867387	AX-87548401	72561986	72611188	0
ssa14	72683407	LOC106570255	0 071388478	AX-87184224	72678925	72724513	0
ssa14	89340676	tp4a2	0 053610637	AX-87355694	89326517	89339711	-966
ssa14	89828790	LOC106570560	0 054460172	AX-87003340	89712174	89808318	-20473
ssa14	89847615	si:dkeyp-120h9.1	0 054822462	AX-87057282	89863107	89896254	15492
ssa14	89858786	si:dkeyp-120h9.1	0 064589424	AX-86982487	89863107	89896254	4321
ssa14	89864078	si:dkeyp-120h9.1	0 065612775	AX-87008675	89863107	89896254	0
ssa14	89875485	si:dkeyp-120h9.1	0 055338129	AX-87848215	89863107	89896254	0
ssa14	89898749	LOC106570558	0 05411635	AX-87702414	89898662	89913094	0
ssa14	89962352	LOC106570561	0 059454139	AX-87102763	89998149	90036004	35797
ssa14	90080117	LOC106590894	0 052964938	AX-87473450	90040233	90083112	0
ssa14	90080621	LOC106590894	0 054838708	AX-87046882	90040233	90083112	0
ssa14	90202258	LOC106570566	0 057498068	AX-87513711	90202142	90314172	0
ssa14	90366705	LOC106570572	0 052981117	AX-87615998	90361364	90367313	0
ssa15	3841768	LOC106570861	0 058582731	AX-87280400	3841695	3845736	0
ssa15	3841771	LOC106570861	0 055746837	AX-87841606	3841695	3845736	0
ssa15	3913129	LOC106570887	0 066788503	AX-87707494	3925222	3927161	12093
ssa15	4536780	LOC106570894	0 06217352	AX-86923107	4287663	4652106	0

ssa15	25581644	kcrb	0 068034256	AX-87325050	25570959	25577487	-4158
ssa15	25605700	kcrb	0 068034256	AX-87220232	25570959	25577487	-28214
ssa15	26172581	zbtb1	0 062566685	AX-87584870	26171824	26179042	0
ssa15	26180241	zbtb1	0 062566685	AX-87549364	26171824	26179042	-1200
ssa15	26182076	LOC106571132	0 062566685	AX-87803518	26181507	26195525	0
ssa15	48115493	LOC106571666	0 053745509	AX-87660596	48012965	48118169	0
ssa15	48118738	LOC106571666	0 053745509	AX-87173614	48012965	48118169	-570
ssa15	48127435	LOC106571666	0 053198347	AX-87523471	48012965	48118169	-9267
ssa15	48430440	LOC106571665	0 058765523	AX-87065271	48243360	48393937	-36504
ssa15	48439290	LOC106571665	0 061710187	AX-86948856	48243360	48393937	-45354
ssa15	48651253	LOC106571662	0 053818685	AX-87635840	48621295	48702205	0
ssa15	63171913	LOC106571843	0 060373548	AX-87774748	63179262	63206913	7349
ssa15	68694633	LOC106572256	0 058407312	AX-87142701	68630044	68700055	0
ssa15	68695166	LOC106572256	0 058407312	AX-87216650	68630044	68700055	0
ssa15	68718840	LOC106572256	0 05734849	AX-87671215	68630044	68700055	-18786
ssa15	76710572	etbr2	0 052753303	AX-87568402	76707213	76710632	0
ssa15	88545715	LOC106572513	0 056594571	AX-87249242	88521586	88567235	0
ssa15	100566691	pex10	0 055736947	AX-86948231	100565951	100570028	0
ssa16	16618786	btbd11b	0 057215444	AX-87431694	16553310	16695791	0
ssa16	18111464	LOC123727758	0 061185351	AX-87124849	18159137	18160412	47673
ssa16	23360774	LOC106573509	0 053351856	AX-87061062	23357373	23596164	0
ssa16	29467896	nr2f2	0 053664415	AX-87779640	29507484	29517571	39588
ssa16	30793287	nrn1la	0 057974621	AX-86916001	30806381	30851433	13094
ssa16	31151928	ripor1	0 057837179	AX-87000549	31040127	31174818	0
ssa16	32442845	LOC106573707	0 073036081	AX-87065292	32461155	32468380	18310
ssa16	32452840	LOC106573707	0 073036081	AX-87394098	32461155	32468380	8315
ssa16	32678141	LOC106573705	0 077919066	AX-87357891	32558830	32696591	0
ssa16	32695471	LOC106573705	0 073022913	AX-87378371	32558830	32696591	0
ssa16	32696114	LOC106573705	0 07044959	AX-87203706	32558830	32696591	0
ssa16	32696114	LOC106573705	0 071060927	AX-87668100	32558830	32696591	0
ssa16	32711947	LOC106573704	0 07044959	AX-86948352	32708679	32711723	-225
ssa16	32724459	LOC106573703	0 06467682	AX-87327554	32714194	32732233	0
ssa16	32736121	LOC106573703	0 060983698	AX-87650094	32714194	32732233	-3889
ssa16	32759292	LOC106573703	0 063931534	AX-87586387	32714194	32732233	-27060
ssa16	32780917	LOC106573703	0 063931534	AX-87103639	32714194	32732233	-48685
ssa16	32881086	LOC106573804	0 053353113	AX-87344883	32851590	32890848	0
ssa16	32900416	LOC106573804	0 064675604	AX-87598581	32851590	32890848	-9569
ssa16	32904528	LOC106573804	0 069409275	AX-87436269	32851590	32890848	-13681
ssa16	32905226	LOC106573804	0 063120716	AX-87666620	32851590	32890848	-14379
ssa16	32943017	LOC106573702	0 069409275	AX-87032727	32942484	33649087	0
ssa16	33095586	LOC106573702	0 062607241	AX-87108320	32942484	33649087	0
ssa16	33163117	LOC106573702	0 069470247	AX-87214521	32942484	33649087	0

ssa16	33205099	LOC106573702	0 067667652	AX-87224116	32942484	33649087	0
ssa16	33214592	LOC106573702	0 067667652	AX-87826181	32942484	33649087	0
ssa16	33241963	LOC106573702	0 062799806	AX-87011361	32942484	33649087	0
ssa16	33264824	LOC106573702	0 062799806	AX-87724858	32942484	33649087	0
ssa16	33284764	LOC106573702	0 056077603	AX-87755922	32942484	33649087	0
ssa16	33354558	LOC106573702	0 067667652	AX-86977495	32942484	33649087	0
ssa16	33419924	LOC106573702	0 054835189	AX-87288929	32942484	33649087	0
ssa16	33497617	LOC106573702	0 064257159	AX-86947645	32942484	33649087	0
ssa16	33551448	LOC106573702	0 06834601	AX-87071979	32942484	33649087	0
ssa16	33554026	LOC106573702	0 06834601	AX-87336875	32942484	33649087	0
ssa16	33629752	LOC106573702	0 062565796	AX-87642767	32942484	33649087	0
ssa16	33772311	LOC106573701	0 068323978	AX-87402017	33752488	33768083	-4229
ssa16	33816645	LOC106573700	0 067938679	AX-87864027	33832433	33857676	15788
ssa16	33832076	LOC106573700	0 067938679	AX-87165234	33832433	33857676	357
ssa16	33856372	LOC106573700	0 067264532	AX-87221937	33832433	33857676	0
ssa16	33856525	LOC106573700	0 067938679	AX-87733756	33832433	33857676	0
ssa16	33869907	LOC106573699	0 075438308	AX-87531636	33858341	33951458	0
ssa16	33945399	LOC106573699	0 075438308	AX-87151269	33858341	33951458	0
ssa16	33955376	LOC106573699	0 061961094	AX-87811389	33858341	33951458	-3919
ssa16	33956089	LOC106573699	0 075438308	AX-87023051	33858341	33951458	-4632
ssa16	34057826	LOC106573696	0 061961094	AX-87091576	34032733	34060675	0
ssa16	34063414	LOC106573696	0 061961094	AX-87254128	34032733	34060675	-2740
ssa16	34067653	LOC106573696	0 075438308	AX-87753307	34032733	34060675	-6979
ssa16	34091529	LOC106573696	0 075438308	AX-87872877	34032733	34060675	-30855
ssa16	34094769	LOC106573696	0 061961094	AX-87370287	34032733	34060675	-34095
ssa16	34109676	LOC106573697	0 0617189	AX-87843327	34131365	34147058	21689
ssa16	34126311	LOC106573697	0 066411276	AX-87630574	34131365	34147058	5054
ssa16	34146487	LOC106573697	0 053446002	AX-87021680	34131365	34147058	0
ssa16	34153235	LOC106573694	0 075438308	AX-87154456	34147187	34156126	0
ssa16	34157745	LOC106573698	0 075438308	AX-87259725	34156088	34157742	-4
ssa16	34170508	LOC106573695	0 075438308	AX-87696932	34160229	34165320	-5189
ssa16	34206427	LOC106573803	0 075420823	AX-87810190	34217983	34219688	11556
ssa16	34346685	bt2a2	0 073596181	AX-87524940	34346941	34347144	256
ssa16	34381472	bt2a2	0 054975614	AX-87560596	34346941	34347144	-34329
ssa16	44994490	LOC106573951	0 069069588	AX-87344364	44993991	45098916	0
ssa16	45095640	LOC106573951	0 069069588	AX-87693152	44993991	45098916	0
ssa16	45095723	LOC106573951	0 069069588	AX-87568859	44993991	45098916	0
ssa16	45696669	LOC106574059	0 074344913	AX-87348248	45663624	45664582	-32088
ssa16	45971339	LOC106574055	0 055713484	AX-87568852	45823768	45981559	0
ssa16	46022924	LOC106574053	0 081450458	AX-86957574	45982916	46016437	-6488
ssa16	46529997	LOC106574075	0 07234659	AX-87458368	46246260	46530210	0
ssa16	49151157	LOC106574090	0 072882468	AX-87630717	49081425	49156478	0

ssa16	49987673	ptprsa	0	058486936	AX-87659373	49975507	50397393	0
ssa16	50017862	ptprsa	0	053366863	AX-87375234	49975507	50397393	0
ssa16	50071953	ptprsa	0	053366863	AX-87005935	49975507	50397393	0
ssa16	50328859	ptprsa	0	054067517	AX-87519794	49975507	50397393	0
ssa16	50439849	LOC106574152	0	08456553	AX-86938461	50445341	50456524	5492
ssa16	50464890	gipc3	0	075317298	AX-87411182	50463084	50499349	0
ssa16	50485642	gipc3	0	084985452	AX-87060626	50463084	50499349	0
ssa16	50499855	LOC106574150	0	083311321	AX-87450408	50499492	50527743	0
ssa16	50526040	LOC106574150	0	080574985	AX-87344335	50499492	50527743	0
ssa16	50536758	asb3	0	064848827	AX-86914996	50535637	50540884	0
ssa16	50574664	LOC106574144	0	084631359	AX-87111600	50558563	50586267	0
ssa16	50593306	LOC106574143	0	080647852	AX-87825094	50592448	50635205	0
ssa16	50612898	LOC106574143	0	074454865	AX-87532653	50592448	50635205	0
ssa16	50675647	LOC106574140	0	059293312	AX-87855323	50678485	50681621	2838
ssa16	50680783	LOC106574140	0	053415203	AX-87411952	50678485	50681621	0
ssa16	50684526	ndufb5	0	073944161	AX-87284199	50681754	50684447	-80
ssa16	50723431	usp13	0	087142562	AX-87648759	50696607	50734745	0
ssa16	50786361	LOC106574138	0	063423856	AX-87225637	50740355	50920531	0
ssa16	50923585	LOC106574138	0	059065993	AX-87057607	50740355	50920531	-3055
ssa16	50950275	LOC123727713	0	078910185	AX-87777567	50968888	50994595	18613
ssa16	50950446	LOC123727713	0	058311873	AX-87672890	50968888	50994595	18442
ssa16	51006940	LOC106574136	0	05281426	AX-87172482	51003281	51015816	0
ssa16	51064157	LOC106574134	0	078818078	AX-87385622	51063813	51067244	0
ssa16	51105587	psmd1	0	083894709	AX-87363258	51084097	51143138	0
ssa16	51105587	htr2b	0	083894709	AX-87363258	51101644	51116973	0
ssa16	51232173	ppp1r2	0	099724427	AX-87605530	51232289	51265404	116
ssa16	51273897	LOC106574127	0	06208409	AX-87353221	51274173	51289118	276
ssa16	51387606	rnf13	0	069267949	AX-87435138	51317541	51404299	0
ssa16	51388974	rnf13	0	053581798	AX-87316601	51317541	51404299	0
ssa16	51538657	LOC106574174	0	055909168	AX-86940816	51536111	51585623	0
ssa16	51625386	rubcn	0	071274942	AX-87227193	51607057	51674153	0
ssa16	51637528	rubcn	0	071274942	AX-87085413	51607057	51674153	0
ssa16	51704488	rubcn	0	084223322	AX-87408508	51607057	51674153	-30336
ssa16	51772073	LOC123727715	0	08590992	AX-87238025	51771166	51773201	0
ssa16	51772931	LOC123727715	0	086249388	AX-87676449	51771166	51773201	0
ssa16	51796663	LOC106574175	0	086249388	AX-87498970	51794778	51796961	0
ssa16	51838955	LOC106574172	0	078281225	AX-87553416	51842980	51854506	4025
ssa16	51870530	dynlt2b	0	080543986	AX-87853696	51863135	51871149	0
ssa16	51918522	nmur1a	0	085541628	AX-87418290	51913734	51951026	0
ssa16	51999672	LOC123727937	0	081487836	AX-87689125	51961871	51961927	-37746
ssa16	52086184	LOC106574168	0	082273323	AX-87627137	52073205	52097302	0
ssa16	52086305	LOC106574168	0	081487836	AX-87838616	52073205	52097302	0

ssa16	52106349	LOC106574165	0	081093661	AX-87821617	52098122	52195287	0
ssa16	52150151	LOC106574165	0	089454695	AX-87743114	52098122	52195287	0
ssa16	52160587	LOC106574165	0	080560147	AX-87486934	52098122	52195287	0
ssa16	52244686	LOC106574164	0	084099084	AX-87346090	52206691	52237153	-7534
ssa16	52479168	LOC106574163	0	060860271	AX-87333547	52461007	52573904	0
ssa16	52570645	LOC106574163	0	058454278	AX-87857604	52461007	52573904	0
ssa16	52583098	LOC106574163	0	058310477	AX-87573554	52461007	52573904	-9195
ssa16	52609498	LOC106574163	0	073187586	AX-87248823	52461007	52573904	-35595
ssa16	71737618	LOC106574536	0	054502265	AX-87047034	71701206	71736827	-792
ssa16	71951707	LOC106574571	0	055704074	AX-87309859	71947764	71995759	0
ssa16	71965603	LOC106574571	0	055520746	AX-87110804	71947764	71995759	0
ssa16	71968084	LOC106574571	0	055591919	AX-87136096	71947764	71995759	0
ssa16	71981121	LOC106574571	0	056788191	AX-87409912	71947764	71995759	0
ssa16	72126197	LOC106574518	0	053468476	AX-87144074	72125561	72132770	0
ssa16	72188614	LOC106574516	0	053165085	AX-87304441	72188234	72209533	0
ssa16	72319184	acvr1l	0	054253999	AX-87015634	72314917	72323300	0
ssa16	72442210	LOC106574508	0	061711415	AX-87099411	72435457	72454040	0
ssa16	72447668	LOC106574508	0	062177554	AX-87786878	72435457	72454040	0
ssa16	72449334	LOC106574508	0	062177554	AX-86904585	72435457	72454040	0
ssa16	72575890	LOC106574495	0	068902586	AX-87560727	72566841	72602233	0
ssa16	76550749	LOC106591349	0	08166831	AX-87126489	76450713	76644121	0
ssa16	76946707	LOC106574735	0	06956491	AX-87627238	76898773	76952329	0
ssa16	77234767	LOC106574938	0	067604351	AX-87130772	77132810	77465492	0
ssa16	77480464	LOC106574938	0	062426521	AX-87695559	77132810	77465492	-14973
ssa16	78011360	LOC106574775	0	059285528	AX-86995822	77966476	77999280	-12081
ssa16	87025462	LOC106598090	0	061042109	AX-87047272	86922932	87062823	0
ssa17	3884024	LOC106561251	0	058615392	AX-87666621	3863478	3916793	0
ssa17	3924388	LOC123728136	0	065049614	AX-87373720	3920803	3924794	0
ssa17	3957366	LOC123728313	0	067323579	AX-87063110	3929966	3966878	0
ssa17	3963655	LOC123728313	0	061069968	AX-87367683	3929966	3966878	0
ssa17	3984247	LOC106561259	0	057496062	AX-87676568	3974708	3990192	0
ssa17	3989838	LOC106561259	0	057496062	AX-86965102	3974708	3990192	0
ssa17	12738739	LOC106575240	0	05475968	AX-87723903	12718918	12735985	-2755
ssa17	12758559	LOC106575200	0	056460343	AX-87053953	12758053	12778905	0
ssa17	12774254	LOC106575200	0	063653244	AX-87000371	12758053	12778905	0
ssa17	12808822	LOC123728339	0	068555721	AX-87762408	12780668	12780722	-28101
ssa17	12809239	LOC123728339	0	068555721	AX-87210447	12780668	12780722	-28518
ssa17	12831185	pudp	0	054884344	AX-87533512	12848457	12864201	17272
ssa17	12854512	pudp	0	068386107	AX-87359523	12848457	12864201	0
ssa17	12863813	pudp	0	059466229	AX-87775856	12848457	12864201	0
ssa17	12925541	LOC106575199	0	055609455	AX-87554868	12905997	12981474	0
ssa17	15385412	LOC106575271	0	052713099	AX-87750618	15336582	15384442	-971

ssa17	17122216	LOC106575286	0 066672747	AX-87488166	17124258	17127064	2042
ssa17	17141018	LOC106575289	0 058349788	AX-86969483	17136218	17171383	0
ssa17	17144692	LOC106575289	0 058349788	AX-87127042	17136218	17171383	0
ssa17	17163874	LOC106575289	0 067690884	AX-87221102	17136218	17171383	0
ssa17	17200886	LOC106575293	0 054760053	AX-87349469	17198654	17202635	0
ssa17	17201358	LOC106575293	0 054760053	AX-87559927	17198654	17202635	0
ssa17	17201893	LOC106575293	0 066644135	AX-87237220	17198654	17202635	0
ssa17	17215949	LOC106575295	0 06406959	AX-87837466	17216195	17254332	246
ssa17	17215955	LOC106575295	0 06406959	AX-87032501	17216195	17254332	240
ssa17	17218243	LOC106575295	0 058473128	AX-87657000	17216195	17254332	0
ssa17	17260231	LOC106575294	0 055234384	AX-87124481	17254239	17267231	0
ssa17	17286247	LOC106575298	0 065052476	AX-86972846	17286033	17292960	0
ssa17	17290599	LOC106575298	0 060109139	AX-87440631	17286033	17292960	0
ssa17	64200990	LOC106576364	0 058935801	AX-87302182	64011231	64267469	0
ssa17	64227841	LOC106576364	0 054119089	AX-87274081	64011231	64267469	0
ssa17	64232063	LOC106576364	0 052972823	AX-87387301	64011231	64267469	0
ssa18	13970482	LOC106576704	0 062262438	AX-87388323	13946580	14052706	0
ssa18	14107293	LOC106576702	0 055953252	AX-87272901	14097074	14111071	0
ssa18	14395799	nt5c2a	0 056630654	AX-87238784	14373176	14400852	0
ssa18	14591441	col13a1	0 056469417	AX-87123387	14563137	14679767	0
ssa18	22498093	LOC106577051	0 052916284	AX-86909782	22273088	22626848	0
ssa18	55954585	LOC106577513	0 054401123	AX-87760832	55934850	55938914	-15672
ssa18	56072776	LOC106577518	0 061924419	AX-86935780	56074379	56095622	1603
ssa18	63336675	LOC106577627	0 058979962	AX-87306747	63225917	63456617	0
ssa18	63673699	LOC106577632	0 05713383	AX-87825456	63667586	63678750	0
ssa18	63691655	LOC106577633	0 065635504	AX-87126823	63682115	63747030	0
ssa18	64609412	LOC106577672	0 062896198	AX-87646758	64607528	64609713	0
ssa18	65127145	LOC106577745	0 059164559	AX-87334216	65119045	65121713	-5433
ssa18	65143807	LOC106577747	0 06149931	AX-87318975	65142667	65144505	0
ssa18	65170737	LOC106577748	0 054577389	AX-86916827	65173119	65174856	2382
ssa18	65220270	LOC106577685	0 061749051	AX-87776650	65218275	65222897	0
ssa18	65343929	LOC106577727	0 054302587	AX-87445598	65344415	65346342	486
ssa18	66539472	LOC123728664	0 05402985	AX-87367593	66465748	66654942	0
ssa18	66673732	LOC106577772	0 057817513	AX-87854865	66687221	66705642	13489
ssa18	66683969	LOC106577772	0 057392106	AX-87543414	66687221	66705642	3252
ssa18	66688034	LOC106577772	0 057392106	AX-87203295	66687221	66705642	0
ssa18	66711936	LOC106577772	0 05750841	AX-87575313	66687221	66705642	-6295
ssa18	66730251	LOC106577772	0 06174501	AX-87646237	66687221	66705642	-24610
ssa18	66854705	LOC106577804	0 055436206	AX-87864961	66830709	66844005	-10701
ssa18	67049915	kdm6ba	0 053935262	AX-87128509	66974077	67079690	0
ssa18	69367434	LOC106577864	0 055817647	AX-86911004	69321572	69374966	0
ssa18	72980956	LOC106577974	0 05459127	AX-87729861	72958020	72962412	-18545

ssa19	12982391	prmt6	0 054982657	AX-86940715	12972466	12973855	-8537
ssa19	13504410	olfm3a	0 064991034	AX-87047943	13474563	13506346	0
ssa19	13718066	arhgap29a	0 067041453	AX-87775065	13644019	13721632	0
ssa19	13736027	LOC106578486	0 068272322	AX-86983129	13732671	13746056	0
ssa19	15028486	pde1ca	0 063282376	AX-87117619	15023579	15193773	0
ssa19	15030930	pde1ca	0 062634539	AX-87771475	15023579	15193773	0
ssa19	15031214	pde1ca	0 059772513	AX-87704305	15023579	15193773	0
ssa19	15032951	pde1ca	0 076678982	AX-87405900	15023579	15193773	0
ssa19	16590199	pfkpa	0 054880119	AX-87441150	16543338	16580029	-10171
ssa19	17455102	LOC106578541	0 060491907	AX-87732896	17245761	17812146	0
ssa19	17993242	LOC123729050	0 058423117	AX-87543784	17959752	17960561	-32682
ssa19	30798468	LOC106578663	0 060066991	AX-87116110	30820565	30828131	22097
ssa19	30798946	LOC106578663	0 063980401	AX-87496004	30820565	30828131	21619
ssa19	30803174	LOC106578663	0 057375622	AX-87544694	30820565	30828131	17391
ssa19	30978377	LOC106578660	0 054158406	AX-87481000	30951222	31017878	0
ssa19	31090986	LOC106578659	0 053180453	AX-87291152	31019807	31091808	0
ssa19	31106590	LOC106578709	0 053180453	AX-87152306	31102228	31103718	-2873
ssa19	31214060	LOC106578655	0 054060317	AX-87476485	31161233	31357940	0
ssa19	31224213	LOC106578655	0 055948974	AX-87460078	31161233	31357940	0
ssa19	31235661	LOC106578655	0 054060317	AX-87731674	31161233	31357940	0
ssa19	31258557	LOC106578655	0 055549599	AX-87160047	31161233	31357940	0
ssa19	31287901	LOC106578655	0 058687143	AX-87556996	31161233	31357940	0
ssa19	31295293	LOC106578655	0 057290359	AX-87635674	31161233	31357940	0
ssa19	31311050	LOC106578655	0 053877883	AX-87151754	31161233	31357940	0
ssa19	31426320	LOC106578728	0 054499674	AX-87522811	31412893	31428954	0
ssa19	31479971	LOC106578729	0 061067228	AX-87546705	31475188	31494509	0
ssa19	31627101	LOC106578734	0 067254425	AX-87636527	31610494	31725698	0
ssa19	31635371	LOC106578734	0 066454704	AX-87077931	31610494	31725698	0
ssa19	31658260	LOC106578734	0 067039493	AX-87297292	31610494	31725698	0
ssa19	31675590	LOC106578734	0 058960402	AX-87215047	31610494	31725698	0
ssa19	31733597	LOC106578734	0 068404974	AX-87003584	31610494	31725698	-7900
ssa19	31887100	LOC106578731	0 065784098	AX-87522636	31882762	31887183	0
ssa19	31888066	LOC106578731	0 073181285	AX-87291236	31882762	31887183	-884
ssa19	31900551	LOC106578731	0 062494282	AX-87542010	31882762	31887183	-13369
ssa19	31900976	LOC106578731	0 070459239	AX-87052630	31882762	31887183	-13794
ssa19	32535245	LOC106578738	0 065419829	AX-87332703	32389092	32596478	0
ssa19	32575619	LOC106578738	0 066325432	AX-86966985	32389092	32596478	0
ssa19	33076748	LOC106578765	0 06230883	AX-87301661	32896140	33460660	0
ssa19	33100956	LOC106578765	0 06230883	AX-87133755	32896140	33460660	0
ssa19	33337986	LOC106578765	0 058990233	AX-86992183	32896140	33460660	0
ssa19	34803713	nid1a	0 061355118	AX-87362384	34782512	34922094	0
ssa19	34804917	nid1a	0 058421289	AX-87632854	34782512	34922094	0

ssa19	34969885	lyst	0 058601004	AX-87371140	34927184	35147808	0
ssa19	35230045	ggps1	0 063999938	AX-87786460	35209889	35248000	0
ssa19	35483775	arid4b	0 063934438	AX-87821340	35248419	35491478	0
ssa19	36858978	LOC106578783	0 057524295	AX-87604174	36734163	36814366	-44613
ssa19	36862034	LOC106578783	0 056698314	AX-87223453	36734163	36814366	-47669
ssa19	39426250	LOC106578757	0 05893694	AX-87676914	39132134	39521385	0
ssa19	39586042	LOC106578803	0 052977419	AX-87196788	39544164	39586340	0
ssa19	39598698	trnaa-cgc	0 052977419	AX-87019097	39608547	39608616	9849
ssa19	39599280	trnaa-cgc	0 052977419	AX-87866813	39608547	39608616	9267
ssa19	39601166	trnaa-cgc	0 052977419	AX-87465718	39608547	39608616	7381
ssa19	40030767	LOC106578764	0 054312045	AX-87742565	40026902	40037369	0
ssa19	40040582	LOC106578821	0 055330643	AX-87673751	40037634	40044110	0
ssa19	40226622	tacc2	0 06285633	AX-87756073	40200544	40277407	0
ssa19	41928151	LOC106578830	0 057237366	AX-87018810	41903650	41947269	0
ssa19	48291569	LOC106578945	0 059984554	AX-87223205	48286556	48307067	0
ssa19	48300460	LOC106578945	0 065088997	AX-87387979	48286556	48307067	0
ssa19	48321685	LOC106578945	0 066183225	AX-86965559	48286556	48307067	-14619
ssa19	56119860	raraa	0 055978834	AX-87598906	55995919	56209733	0
ssa19	56160306	raraa	0 053170289	AX-87157333	55995919	56209733	0
ssa19	56205891	raraa	0 066947889	AX-87402848	55995919	56209733	0
ssa19	56294313	top2a	0 064461931	AX-87415895	56269812	56294589	0
ssa19	56294313	lrrc3ca	0 064461931	AX-87415895	56284158	56313299	0
ssa19	56314203	lrrc3ca	0 064461931	AX-87365294	56284158	56313299	-905
ssa19	56734067	LOC106579241	0 064227357	AX-87589014	56717803	56732605	-1463
ssa19	56736250	LOC106579241	0 068771748	AX-87499571	56717803	56732605	-3646
ssa19	56751747	LOC106579239	0 064227357	AX-87748116	56763075	56838419	11328
ssa19	56782741	LOC106579239	0 068868812	AX-87453833	56763075	56838419	0
ssa19	56802586	LOC106579239	0 068868812	AX-87430184	56763075	56838419	0
ssa19	56809756	LOC106579239	0 068868812	AX-87810344	56763075	56838419	0
ssa19	56821317	LOC106579239	0 068868812	AX-87629859	56763075	56838419	0
ssa19	56828644	LOC106579239	0 068868812	AX-87075721	56763075	56838419	0
ssa19	56836815	LOC106579239	0 068868812	AX-87425852	56763075	56838419	0
ssa19	56837194	LOC106579239	0 068868812	AX-87804065	56763075	56838419	0
ssa19	56839526	LOC106579238	0 064438415	AX-87511608	56839045	56854867	0
ssa19	56846232	LOC106579238	0 064438415	AX-87668630	56839045	56854867	0
ssa19	56854036	LOC106579238	0 064438415	AX-87680343	56839045	56854867	0
ssa19	56868731	LOC106579237	0 064438415	AX-87801795	56863010	56874567	0
ssa19	56870971	LOC106579237	0 064438415	AX-87548800	56863010	56874567	0
ssa19	56889289	znf646	0 064213605	AX-87540925	56882744	56892890	0
ssa19	56890317	znf646	0 064213605	AX-87198966	56882744	56892890	0
ssa19	56894961	LOC106579236	0 055566922	AX-87833402	56893824	56905399	0
ssa19	56902251	LOC106579236	0 065837122	AX-87823784	56893824	56905399	0

ssa19	56911746	rnf40	0 064438415	AX-87786098	56906542	56919433	0
ssa19	56919452	rnf40	0 065531975	AX-87471259	56906542	56919433	-20
ssa19	56922686	phkg2	0 064438415	AX-87201623	56919594	56942502	0
ssa19	56942637	ccdc189	0 064438415	AX-87439108	56942453	56944669	0
ssa19	57014768	kctd5b	0 068571441	AX-87664174	56989790	57022723	0
ssa19	57019289	kctd5b	0 054382976	AX-87141590	56989790	57022723	0
ssa19	57057396	cssa19h7orf50	0 068571441	AX-87262573	57049957	57066649	0
ssa19	57057396	LOC106579223	0 068571441	AX-87262573	57052898	57058541	0
ssa19	60932815	LOC106579138	0 054050035	AX-87073349	60865813	61015107	0
ssa19	61153989	naglu	0 054214579	AX-87250961	61133138	61154781	0
ssa19	61212807	coasy	0 054214579	AX-87136458	61197946	61218430	0
ssa19	61213353	coasy	0 058996396	AX-86929657	61197946	61218430	0
ssa19	61221592	LOC106579130	0 053864735	AX-87516762	61221501	61241399	0
ssa20	6582497	LOC106580191	0 065324258	AX-87660717	6551112	6587273	0
ssa20	6605124	LOC106580191	0 071048062	AX-87218347	6551112	6587273	-17852
ssa20	6621595	LOC123729345	0 07231627	AX-87048956	6623282	6636324	1687
ssa20	6753862	LOC106580185	0 067333809	AX-87197272	6753192	6762900	0
ssa20	6760434	LOC106580185	0 066252323	AX-87759950	6753192	6762900	0
ssa20	6797404	LOC106580242	0 064859422	AX-86937239	6790118	6804593	0
ssa20	6797530	LOC106580242	0 064859422	AX-87640696	6790118	6804593	0
ssa20	6819321	LOC106580241	0 065831203	AX-86977743	6818050	6870757	0
ssa20	6826775	LOC106580241	0 055745525	AX-87159695	6818050	6870757	0
ssa20	6826883	LOC106580241	0 0620996	AX-87546889	6818050	6870757	0
ssa20	6895163	LOC106580184	0 055059463	AX-86969774	6852855	6956675	0
ssa20	6911342	LOC106580184	0 056304451	AX-87668371	6852855	6956675	0
ssa20	8924832	LOC123729318	0 063588147	AX-87456006	8912315	8922059	-2774
ssa20	8951146	LOC123729318	0 072494719	AX-87680906	8912315	8922059	-29088
ssa20	14967040	LOC100380695	0 056632199	AX-87183899	14951923	14972599	0
ssa20	15012043	LOC106580092	0 064989933	AX-86928814	15002207	15087514	0
ssa20	19332265	LOC106579985	0 061697055	AX-87070471	19307908	19334705	0
ssa20	21522624	LOC106579918	0 053532467	AX-87471614	21430698	21573018	0
ssa20	21526230	LOC106579918	0 05346995	AX-87055304	21430698	21573018	0
ssa20	21528351	LOC106579918	0 053339148	AX-87068559	21430698	21573018	0
ssa20	21530961	LOC106579918	0 053339148	AX-87432826	21430698	21573018	0
ssa20	21533000	LOC106579918	0 053339148	AX-87178242	21430698	21573018	0
ssa20	38325276	LOC106580556	0 056386141	AX-87002176	38249424	38370128	0
ssa20	38381606	LOC106580558	0 059836166	AX-86995134	38367649	38389184	0
ssa20	39167265	LOC106580577	0 061165525	AX-87533514	39125998	39170913	0
ssa20	57112936	LOC106580911	0 060416742	AX-87713625	57086723	57182096	0
ssa20	57227127	LOC106580945	0 059974059	AX-87733567	57195460	57294594	0
ssa20	57303759	LOC106580920	0 067720766	AX-87683693	57302196	57488481	0
ssa20	57314713	LOC106580920	0 066433595	AX-87064082	57302196	57488481	0

ssa20	74822135	LOC106581305	0 062724953	AX-87594981	74814136	74841466	0
ssa21	10623545	LOC106581870	0 053677976	AX-87513220	10653191	10667861	29646
ssa21	18580376	efnb2a	0 060411574	AX-87171112	18552400	18578791	-1586
ssa21	18602026	arglu1a	0 056378749	AX-87086765	18582959	18589801	-12226
ssa21	18607158	arglu1a	0 059510871	AX-87860454	18582959	18589801	-17358
ssa21	18618789	arglu1a	0 061856123	AX-87265928	18582959	18589801	-28989
ssa21	18634479	arglu1a	0 062526686	AX-87032951	18582959	18589801	-44679
ssa21	18670705	LOC106581720	0 072360642	AX-87465139	18718377	18729024	47672
ssa21	18728585	LOC106581720	0 079804967	AX-87343623	18718377	18729024	0
ssa21	18741161	LOC106581739	0 072128143	AX-87748639	18742458	18766765	1297
ssa21	18741692	LOC106581739	0 072128143	AX-87143008	18742458	18766765	766
ssa21	48290473	LOC106582440	0 077358611	AX-87069770	48300647	48461234	10174
ssa21	48296441	LOC106582440	0 077358611	AX-87089042	48300647	48461234	4206
ssa21	48310465	LOC106582440	0 079173303	AX-87415021	48300647	48461234	0
ssa21	57990361	LOC106582571	0 06123144	AX-86931814	58003846	58019546	13485
ssa22	22729465	LOC106582998	0 059933718	AX-87143337	22729561	22740533	96
ssa22	25690071	tfeb	0 062050754	AX-87483035	25710544	25847066	20473
ssa22	29040791	LOC106583226	0 065805691	AX-87689850	29024673	29074490	0
ssa22	29057995	LOC106583226	0 061764231	AX-87834777	29024673	29074490	0
ssa22	30694762	LOC106583191	0 05707373	AX-87853608	30694441	30700401	0
ssa22	30705206	LOC106583189	0 05707373	AX-87705896	30705017	30708898	0
ssa22	31266477	LOC106583181	0 053878003	AX-87639280	31205966	31303598	0
ssa22	31286693	LOC106583181	0 066900138	AX-87281798	31205966	31303598	0
ssa22	31324456	LOC106583181	0 058308376	AX-87189974	31205966	31303598	-20859
ssa22	31416552	LOC106583180	0 055179495	AX-87114059	31364752	31467375	0
ssa22	31418236	LOC106583180	0 062701324	AX-87001027	31364752	31467375	0
ssa22	31610989	LOC106583179	0 053956272	AX-87026936	31601982	31614591	0
ssa22	40498160	LOC106583501	0 061245723	AX-87066164	40464018	40665918	0
ssa22	40971457	LOC106583499	0 05319532	AX-87665113	40919742	41076416	0
ssa22	41059978	LOC106583499	0 054968838	AX-87848785	40919742	41076416	0
ssa22	42270530	LOC106583381	0 055593049	AX-86954832	42271086	42274267	556
ssa22	42324060	LOC106583452	0 052675708	AX-87352477	42278642	42361104	0
ssa22	54408464	grip2b	0 060843074	AX-87417897	54242269	54476677	0
ssa22	56125393	dag1	0 054094675	AX-87518599	56023673	56095144	-30250
ssa22	56211790	nicn1	0 057449921	AX-87548260	56211826	56229521	36
ssa22	56228788	nicn1	0 057037932	AX-87146666	56211826	56229521	0
ssa22	56239451	nicn1	0 07174288	AX-87812636	56211826	56229521	-9931
ssa22	56244587	nicn1	0 063523589	AX-87135136	56211826	56229521	-15067
ssa22	56293564	LOC106583761	0 065311808	AX-87175384	56310032	56313094	16468
ssa22	56304687	LOC106583761	0 062668442	AX-87413980	56310032	56313094	5345
ssa22	56617556	LOC106583731	0 069675856	AX-87400126	56643110	57002209	25554
ssa23	1754915	LOC106583926	0 067707827	AX-87003597	1751580	1754108	-808

ssa23	1863210	LOC106583924	0 067707827	AX-87745867	1826370	1862879	-332
ssa23	1863702	LOC106583924	0 067707827	AX-87660855	1826370	1862879	-824
ssa23	2071567	LOC106583920	0 052702451	AX-87265651	2033411	2116005	0
ssa23	2309217	LOC106583913	0 063567265	AX-87270664	2308500	2314580	0
ssa23	2631177	mk03	0 06412017	AX-87229488	2629161	2643711	0
ssa23	2702106	LOC106583902	0 058894638	AX-87605253	2711525	2746192	9419
ssa23	3242338	LOC106583946	0 058941292	AX-86989758	3240330	3286119	0
ssa23	3276452	LOC106583946	0 055548835	AX-87725891	3240330	3286119	0
ssa23	3313356	LOC106583946	0 058941292	AX-87442741	3240330	3286119	-27238
ssa23	4854901	LOC106583966	0 067539923	AX-87053941	4825116	4891178	0
ssa23	4951821	LOC106583933	0 059079708	AX-87312883	4961451	5164659	9630
ssa23	4951871	LOC106583933	0 059079708	AX-87530343	4961451	5164659	9580
ssa23	4953252	LOC106583933	0 059079708	AX-87569440	4961451	5164659	8199
ssa23	7140552	LOC106584010	0 053333216	AX-87167901	7098199	7258913	0
ssa23	10405630	LOC106583991	0 05530663	AX-87520618	10321368	10425211	0
ssa23	15754506	LOC106584181	0 054922645	AX-87023260	15424229	15847640	0
ssa23	15779640	LOC106584181	0 054238759	AX-86933077	15424229	15847640	0
ssa23	21143631	LOC106584113	0 062374155	AX-86937077	21011162	21174645	0
ssa23	21381461	LOC106584104	0 059910165	AX-87443260	21369679	21440062	0
ssa23	22137938	cac3	0 058074316	AX-87362093	22133498	22136412	-1527
ssa23	22182876	LOC106584215	0 062515347	AX-87497833	22199665	22207771	16789
ssa23	23311904	LOC106584277	0 055110248	AX-87122251	23308190	23316410	0
ssa23	23332324	LOC106584278	0 058782395	AX-87244925	23327638	23333141	0
ssa23	23348380	LOC106584278	0 058782395	AX-87055346	23327638	23333141	-15240
ssa23	23430356	LOC106584279	0 060113499	AX-87005213	23464674	23521091	34318
ssa23	23430419	LOC106584279	0 061559113	AX-87348328	23464674	23521091	34255
ssa23	23442224	LOC106584279	0 070751151	AX-87776395	23464674	23521091	22450
ssa23	23516359	LOC106584279	0 086100461	AX-87069671	23464674	23521091	0
ssa23	23571119	tnfl6	0 082356909	AX-87848796	23570711	23573503	0
ssa23	23571699	tnfl6	0 090434639	AX-87771762	23570711	23573503	0
ssa23	23571888	tnfl6	0 08175645	AX-87315479	23570711	23573503	0
ssa23	23582390	tnfl6	0 063099667	AX-87566578	23570711	23573503	-8888
ssa23	23614978	LOC106584281	0 057210712	AX-87167505	23648168	23672534	33190
ssa23	23616082	LOC106584281	0 057210712	AX-87525762	23648168	23672534	32086
ssa23	23658833	LOC106584281	0 063835358	AX-87692315	23648168	23672534	0
ssa23	23828224	LOC106584283	0 055620578	AX-87673010	23676233	23834657	0
ssa23	23834477	LOC106584283	0 055620578	AX-87024798	23676233	23834657	0
ssa23	23863687	LOC106584286	0 054392238	AX-87505001	23864126	23881407	439
ssa23	24062565	LOC106584288	0 058969616	AX-87229374	24033194	24084498	0
ssa23	25163035	LOC106584250	0 074203646	AX-87571057	25199638	25201977	36603
ssa23	25263084	LOC123729878	0 074203646	AX-87186656	25303029	25312041	39945
ssa23	25637953	LOC106584306	0 055294643	AX-87341624	25495951	25853832	0

ssa23	25641375	LOC106584306	0 055294643	AX-87266819	25495951	25853832	0
ssa23	25816515	LOC106584306	0 05759281	AX-87546624	25495951	25853832	0
ssa23	25816656	LOC106584306	0 05759281	AX-87003103	25495951	25853832	0
ssa23	25846472	LOC106584306	0 058457981	AX-87667552	25495951	25853832	0
ssa23	26091142	LOC106584308	0 053075254	AX-87663220	26024137	26114925	0
ssa23	26291216	LOC106584314	0 053184113	AX-87746167	26285872	26322194	0
ssa23	29257430	LOC106584354	0 058540033	AX-87441549	29256631	29270177	0
ssa23	30539843	LOC106584383	0 052876456	AX-87399103	30530423	30547299	0
ssa23	30763961	m6pbp	0 066350261	AX-86999589	30738713	30753426	-10536
ssa23	30765742	m6pbp	0 064793931	AX-87356535	30738713	30753426	-12317
ssa23	30826666	LOC106584391	0 053702538	AX-87865831	30789490	30923995	0
ssa23	32459139	oaz1s	0 059753146	AX-87861038	32449423	32461144	0
ssa23	32711088	LOC106584429	0 055805854	AX-87088675	32710265	32721898	0
ssa23	33031253	LOC106584442	0 058787455	AX-87745685	33065376	33077406	34123
ssa23	42256676	LOC106584658	0 054791435	AX-87201485	42261492	42263646	4816
ssa24	3234032	LOC106584969	0 053319484	AX-87142281	3199408	3207222	-26811
ssa24	5459812	rgs7bpa	0 053696094	AX-87296013	5439190	5463425	0
ssa24	11990787	tango2	0 073481255	AX-87468406	11954546	12011870	0
ssa24	12004452	tango2	0 069140084	AX-87426098	11954546	12011870	0
ssa24	12010587	tango2	0 053945144	AX-87530867	11954546	12011870	0
ssa24	12014506	LOC106585092	0 06687269	AX-87610566	12012324	12023293	0
ssa24	12014572	LOC106585092	0 06687269	AX-87044217	12012324	12023293	0
ssa24	12344753	LOC106585083	0 062954676	AX-87015272	12273333	12654145	0
ssa24	12349881	LOC106585083	0 064996048	AX-87183796	12273333	12654145	0
ssa24	12351542	LOC106585083	0 067134097	AX-87315991	12273333	12654145	0
ssa24	12351771	LOC106585083	0 054376462	AX-87781107	12273333	12654145	0
ssa24	20342343	LOC106585590	0 071800234	AX-87239924	20329014	20343617	0
ssa24	20949635	LOC106585560	0 054053811	AX-87204252	20892956	20968187	0
ssa24	20957723	LOC106585560	0 072453404	AX-87193966	20892956	20968187	0
ssa24	21004098	LOC106585561	0 057582178	AX-87515370	20981522	21037148	0
ssa24	21012929	LOC106585561	0 064103673	AX-87023605	20981522	21037148	0
ssa24	21259311	LOC106585582	0 060270298	AX-87624319	21256609	21260985	0
ssa24	21407951	LOC106585552	0 067731377	AX-86949904	21391968	21460801	0
ssa24	21446451	LOC106585552	0 067731377	AX-87364803	21391968	21460801	0
ssa24	21455572	LOC106585552	0 067731377	AX-87789153	21391968	21460801	0
ssa24	21467210	LOC106585551	0 067731377	AX-87849517	21465804	21469038	0
ssa24	21536849	LOC106585547	0 061171183	AX-87361673	21519504	21526314	-10536
ssa24	21553029	LOC106585546	0 07698173	AX-87764483	21552505	21577605	0
ssa24	21570742	LOC106585546	0 061171183	AX-87596089	21552505	21577605	0
ssa24	21572814	LOC106585546	0 056567508	AX-87682886	21552505	21577605	0
ssa24	21808103	pbx3b	0 068812182	AX-87042055	21815083	21905438	6980
ssa24	21832472	pbx3b	0 064215618	AX-87447590	21815083	21905438	0

ssa24	21840203	pbx3b	0 072291588	AX-87090632	21815083	21905438	0
ssa24	21840688	pbx3b	0 072291588	AX-87429837	21815083	21905438	0
ssa24	21843061	pbx3b	0 064215618	AX-87268384	21815083	21905438	0
ssa24	21846138	pbx3b	0 061037	AX-87712800	21815083	21905438	0
ssa24	21855907	pbx3b	0 072291588	AX-87302501	21815083	21905438	0
ssa24	21855990	pbx3b	0 067457318	AX-87793024	21815083	21905438	0
ssa24	21912377	pbx3b	0 069391309	AX-87339210	21815083	21905438	-6940
ssa24	21917677	pbx3b	0 069391309	AX-87101228	21815083	21905438	-12240
ssa24	21931750	pbx3b	0 059674748	AX-86958553	21815083	21905438	-26313
ssa24	22086926	lmx1bb	0 064897982	AX-87653988	22120898	22190798	33972
ssa24	22159283	lmx1bb	0 068057492	AX-86912916	22120898	22190798	0
ssa24	22433522	ncs1b	0 055513971	AX-86985671	22424658	22459357	0
ssa24	22438529	ncs1b	0 053313704	AX-87354061	22424658	22459357	0
ssa24	22478570	LOC106585535	0 05393923	AX-87057270	22475153	22483150	0
ssa24	22578976	LOC106585532	0 07585896	AX-87271999	22544288	22758640	0
ssa24	22584704	LOC106585532	0 070701239	AX-87140820	22544288	22758640	0
ssa24	22586664	LOC106585532	0 061436181	AX-87311045	22544288	22758640	0
ssa24	22627567	LOC106585532	0 061587183	AX-87079543	22544288	22758640	0
ssa24	22638818	LOC106585532	0 078579903	AX-87202584	22544288	22758640	0
ssa24	22646179	LOC106585532	0 067126821	AX-86959975	22544288	22758640	0
ssa24	22672453	LOC106585532	0 0789	AX-87748091	22544288	22758640	0
ssa24	22684666	LOC106585532	0 087675009	AX-87037117	22544288	22758640	0
ssa24	22684711	LOC106585532	0 087675009	AX-87680316	22544288	22758640	0
ssa24	22736657	LOC106585532	0 07998825	AX-87310049	22544288	22758640	0
ssa24	22737347	LOC106585532	0 07998825	AX-87429650	22544288	22758640	0
ssa24	22752883	LOC106585532	0 078763467	AX-87796906	22544288	22758640	0
ssa24	22783116	LOC106585531	0 052958898	AX-87038535	22769387	22805543	0
ssa24	22805383	LOC106585531	0 057625409	AX-87111495	22769387	22805543	0
ssa24	25039103	LOC106585466	0 053342754	AX-87397113	25024115	25047690	0
ssa24	25073185	LOC106585464	0 059814295	AX-87333217	25061002	25071794	-1392
ssa24	25094074	LOC106585462	0 059814295	AX-87429418	25103326	25107327	9252
ssa24	25107810	LOC106585463	0 059814295	AX-87266017	25107609	25109307	0
ssa24	25132389	gstt1a	0 055856205	AX-87733279	25131510	25134715	0
ssa24	25428657	LOC106585447	0 055626764	AX-87403457	25428904	25479093	247
ssa24	25431157	LOC106585447	0 055626764	AX-87231023	25428904	25479093	0
ssa24	25444120	LOC106585447	0 055626764	AX-87609027	25428904	25479093	0
ssa24	25444377	LOC106585447	0 055626764	AX-87241392	25428904	25479093	0
ssa24	25470398	LOC106585447	0 056133353	AX-87155687	25428904	25479093	0
ssa24	25476308	LOC106585447	0 061170274	AX-87240201	25428904	25479093	0
ssa24	25479399	LOC106585447	0 055626764	AX-87745965	25428904	25479093	-307
ssa24	25637309	nr6a1a	0 05404696	AX-87122136	25584559	25725848	0
ssa24	25637309	LOC123730376	0 05404696	AX-87122136	25635836	25647748	0

ssa24	25985433	LOC106585441	0	054623048	AX-87863081	25984824	25992162	0
ssa24	26185722	LOC100136483	0	056717104	AX-87405851	26182857	26185589	-134
ssa24	35508226	aqp3a	0	057968778	AX-87249308	35503131	35510359	0
ssa24	35508506	aqp3a	0	057968778	AX-87160388	35503131	35510359	0
ssa24	35717205	si:dkey-40c11.2	0	062681492	AX-87461294	35678272	35740030	0
ssa24	48286529	dmrt1	0	053079111	AX-87831049	48210596	48275304	-11226
ssa25	4169326	LOC106586029	0	0563184	AX-87499098	4101079	4191483	0
ssa25	10271158	hibch	0	054517654	AX-87821737	10245288	10292902	0
ssa25	10273010	hibch	0	05394362	AX-87530767	10245288	10292902	0
ssa25	19664645	LOC106586305	0	053190294	AX-87146255	19662114	19671025	0
ssa25	26616444	LOC106586456	0	057013012	AX-87477968	26468193	26649698	0
ssa25	26617776	LOC106586456	0	057013012	AX-87698371	26468193	26649698	0
ssa25	26693061	LOC106586458	0	05580941	AX-87859283	26656784	26704634	0
ssa25	26718848	LOC106586459	0	053786686	AX-87663271	26722178	26725972	3330
ssa25	26734611	LOC106586460	0	056012241	AX-87637343	26738076	26773873	3465
ssa25	26767642	LOC106586460	0	064946059	AX-87759239	26738076	26773873	0
ssa25	26768072	LOC106586460	0	059884979	AX-87620442	26738076	26773873	0
ssa25	26811078	LOC106586462	0	066084581	AX-87250558	26789501	26960201	0
ssa25	26963025	LOC106586462	0	05997013	AX-87461013	26789501	26960201	-2825
ssa25	27018098	LOC106586461	0	059922401	AX-87679095	26970957	27031776	0
ssa25	28873826	LOC106586512	0	052861803	AX-87546557	28865764	28879599	0
ssa25	28878139	LOC106586512	0	054748407	AX-87772435	28865764	28879599	0
ssa25	28884043	LOC106586409	0	055685708	AX-87299245	28882712	28884964	0
ssa25	28996054	vgll3	0	057649176	AX-87309414	28995238	28999269	0
ssa25	29007451	vgll3	0	056842105	AX-87420691	28995238	28999269	-8183
ssa25	29007652	vgll3	0	056842105	AX-86940584	28995238	28999269	-8384
ssa25	29080742	akap11	0	060000813	AX-87042309	29055826	29085687	0
ssa25	29110453	tnfsf11	0	056722358	AX-87121759	29105583	29115762	0
ssa25	29119898	tnfsf11	0	053890287	AX-87751693	29105583	29115762	-4137
ssa25	29753987	ranbp2	0	07702888	AX-86988021	29734423	29756244	0
ssa25	29822025	LOC106586531	0	05920853	AX-87783219	29800340	29830444	0
ssa25	29830159	LOC106586531	0	05920853	AX-87268522	29800340	29830444	0
ssa25	29893178	LOC106586545	0	05920853	AX-87118662	29886543	29892451	-728
ssa25	31244615	LOC106586592	0	05677109	AX-87328956	31196994	31241997	-2619
ssa25	31250539	gja3	0	052976343	AX-87752350	31251779	31256641	1240
ssa25	31259733	gjb8	0	052976343	AX-87657384	31257065	31260551	0
ssa25	31279701	cryl1	0	064920756	AX-87026644	31261651	31295460	0
ssa25	31293209	cryl1	0	064920756	AX-87497379	31261651	31295460	0
ssa25	31540848	wars2	0	062270198	AX-87728093	31529387	31550248	0
ssa25	31541553	wars2	0	062270198	AX-87122421	31529387	31550248	0
ssa25	31694416	LOC106586553	0	055733947	AX-87209817	31676473	31678840	-15577
ssa25	31696297	LOC106586553	0	055733947	AX-87819526	31676473	31678840	-17458

ssa25	34183991	LOC106586649	0 078265651	AX-87293462	34183280	34214911	0
ssa25	36220311	asb11	0 054083063	AX-87115760	36219298	36224816	0
ssa25	36221113	asb11	0 054083063	AX-86951297	36219298	36224816	0
ssa25	36223234	asb11	0 054083063	AX-86940804	36219298	36224816	0
ssa25	36296289	LOC106586687	0 061670486	AX-87214745	36283148	36328187	0
ssa25	36718221	LOC106586712	0 057880897	AX-86911738	36745801	36846505	27580
ssa25	36728931	LOC106586712	0 057880897	AX-87751077	36745801	36846505	16870
ssa25	36738563	LOC106586712	0 054104413	AX-87538082	36745801	36846505	7238
ssa25	36791125	LOC106586712	0 060700752	AX-87306962	36745801	36846505	0
ssa25	38262450	rnaseh2b	0 071464935	AX-87199807	38256228	38266908	0
ssa25	39333184	LOC106586740	0 054402825	AX-86909386	39329647	39356881	0
ssa25	39333184	LOC123730499	0 054402825	AX-86909386	39332314	39333192	0
ssa25	48766580	cnga4	0 052924277	AX-87153668	48761811	48767166	0
ssa25	48860338	LOC106586898	0 055781035	AX-87766256	48864286	48869641	3948
ssa25	50593315	LOC106586925	0 056186928	AX-87446734	50587467	50597681	0
ssa25	50593425	LOC106586925	0 056186928	AX-87152239	50587467	50597681	0
ssa25	50604988	LOC106586925	0 055234367	AX-87113777	50587467	50597681	-7308
ssa25	52562383	LOC106592342	0 057922461	AX-87082599	52562203	52683677	0
ssa26	1674806	cntrl	0 06196042	AX-87526237	1560419	1675163	0
ssa26	1674806	cfap77	0 06196042	AX-87526237	1672344	1801205	0
ssa26	7510087	LOC106587065	0 056037916	AX-87577023	7439617	7726942	0
ssa26	9325096	LOC106587091	0 054263576	AX-87811688	9322426	9351795	0
ssa26	9325484	LOC106587091	0 053523593	AX-87681691	9322426	9351795	0
ssa26	9344496	LOC106587091	0 052775521	AX-87051596	9322426	9351795	0
ssa26	9346972	LOC106587091	0 055294097	AX-87635509	9322426	9351795	0
ssa26	9351488	LOC106587091	0 052775521	AX-87035393	9322426	9351795	0
ssa26	9566534	LOC106587094	0 056398126	AX-86954066	9523696	9693485	0
ssa26	10500162	mtch2	0 06176631	AX-87447070	10498121	10514104	0
ssa26	10500249	mtch2	0 052673657	AX-87181881	10498121	10514104	0
ssa26	19701606	LOC106587320	0 064236821	AX-87034873	19573824	19703242	0
ssa26	19716004	LOC106587320	0 055454855	AX-87400937	19573824	19703242	-12763
ssa26	19729540	LOC106587324	0 063243188	AX-87092549	19734281	20146244	4741
ssa26	19737657	LOC106587324	0 060218865	AX-87097240	19734281	20146244	0
ssa26	19815959	LOC106587324	0 070057344	AX-87324760	19734281	20146244	0
ssa26	19915752	LOC106587324	0 109594737	AX-87037910	19734281	20146244	0
ssa26	19971690	LOC106587324	0 090680597	AX-87057338	19734281	20146244	0
ssa26	20048314	LOC106587324	0 077304782	AX-87145605	19734281	20146244	0
ssa26	25686608	plekha7b	0 059566728	AX-87467198	25554991	25729174	0
ssa26	25757322	LOC106587493	0 054835469	AX-87256907	25736997	25940966	0
ssa26	25802423	LOC106587493	0 060599376	AX-87408238	25736997	25940966	0
ssa26	26260150	LOC106587530	0 071749915	AX-87254452	26179060	26272820	0
ssa26	26264388	LOC106587530	0 071749915	AX-87536231	26179060	26272820	0

ssa26	26264849	LOC106587530	0	071749915	AX-87217518	26179060	26272820	0
ssa26	26313375	rras2	0	058057248	AX-87726684	26289215	26334519	0
ssa26	28129380	brd7	0	060369624	AX-87330843	28127980	28137681	0
ssa26	28133535	brd7	0	053523032	AX-87236470	28127980	28137681	0
ssa26	28165325	brd7	0	052878471	AX-87431447	28127980	28137681	-27645
ssa26	28191045	LOC106587577	0	058070481	AX-87645394	28234942	28274430	43897
ssa26	29559336	LOC106587595	0	076785802	AX-87487759	29394912	29588799	0
ssa26	49116778	LOC106572911	0	063783273	AX-87149778	49088424	49131631	0
ssa27	21537131	LOC106588718	0	079814734	AX-87288832	21549270	21590684	12139
ssa27	21537244	LOC106588718	0	079814734	AX-87148251	21549270	21590684	12026
ssa27	25744639	LOC106588819	0	053510433	AX-87278612	25730282	25746667	0
ssa27	29456763	LOC106588886	0	055858945	AX-87745810	29454923	29461376	0
ssa27	33998757	LOC106588992	0	056214022	AX-87695302	33998354	34251134	0
ssa27	33998774	LOC106588992	0	056214022	AX-87291204	33998354	34251134	0
ssa27	33999933	LOC106588992	0	053976472	AX-87652618	33998354	34251134	0
ssa27	34046351	LOC106588992	0	056865843	AX-87235482	33998354	34251134	0
ssa28	2086675	LOC106589304	0	057650531	AX-87135232	2021495	2214959	0
ssa28	10201545	ca10a	0	057351225	AX-87826102	9922977	10247231	0
ssa28	10395334	LOC106589448	0	052704832	AX-87171672	10390582	10410663	0
ssa28	10397029	LOC106589448	0	058058537	AX-87558167	10390582	10410663	0
ssa28	10410971	LOC106589448	0	055489047	AX-87048205	10390582	10410663	-309
ssa28	10439199	LOC106589450	0	06112372	AX-86974907	10431990	10448408	0
ssa28	10520672	LOC106589452	0	055125121	AX-87816215	10519890	10554705	0
ssa28	12146823	LOC106589486	0	057530541	AX-87393834	12144714	12154344	0
ssa28	12146823	LOC106589487	0	057530541	AX-87393834	12144714	12147200	0
ssa28	12154013	LOC106589486	0	072597005	AX-87016139	12144714	12154344	0
ssa28	12184612	myocd	0	069168711	AX-86911992	12183786	12331195	0
ssa28	15694262	LOC106589576	0	058981613	AX-87870437	15676863	15766045	0
ssa28	15733489	LOC106589576	0	064238978	AX-87055308	15676863	15766045	0
ssa28	16899192	LOC106589211	0	061343005	AX-87078350	16842469	16928399	0
ssa28	16899418	LOC106589211	0	061343005	AX-87549288	16842469	16928399	0
ssa28	16936298	dnai2b	0	053975868	AX-87448695	16934693	16945700	0
ssa28	16955469	ttyh2	0	059147532	AX-87787978	16952092	17067553	0
ssa28	28763716	LOC106589820	0	056516291	AX-87088773	28758532	28802561	0
ssa28	31205535	LOC106589866	0	054061096	AX-87502719	30973752	31865071	0
ssa28	32088122	LOC106589900	0	061887306	AX-87166069	31973775	32111726	0
ssa28	32127070	LOC106589899	0	074024037	AX-87244477	32119621	32228073	0
ssa28	32127758	LOC106589899	0	069928485	AX-87668111	32119621	32228073	0
ssa29	16077866	gpr158a	0	068117744	AX-87571750	16008824	16111329	0
ssa29	16078326	gpr158a	0	07060853	AX-87657387	16008824	16111329	0
ssa29	21815523	ptprma	0	058232238	AX-87419449	21717733	22017446	0
ssa29	21833801	ptprma	0	058849438	AX-87468960	21717733	22017446	0

ssa29	21993614	ptprma	0	054392865	AX-87537566	21717733	22017446	0
ssa29	21993632	ptprma	0	055114344	AX-86990817	21717733	22017446	0
ssa29	41337436	LOC106590692	0	055984524	AX-87244438	41336261	41419514	0
ssa29	41415927	LOC106590692	0	054178943	AX-87017818	41336261	41419514	0
ssa29	41418427	LOC106590692	0	062850487	AX-86976645	41336261	41419514	0

Table S4 The top 1% of associations with late run timing in 11 populations of North American Atlantic salmon, identified using a partial redundancy analysis (pRDA) that accounts for population structure by using the first three PC axes that describe population structure. Distance from the closest gene is reported alongside where in the genome the gene starts and ends.

Chromosome	Position	gene	loadings	SNP	start_gene	end_gene	distance
ssa01	2825709	LOC106560212	0 061258166	AX-87460327	2692404	2842935	0
ssa01	10176181	mdga2a	0 06829637	AX-87163508	10046531	10253446	0
ssa01	10177806	mdga2a	0 065779858	AX-87562724	10046531	10253446	0
ssa01	10198409	mdga2a	0 058187345	AX-87423249	10046531	10253446	0
ssa01	17972762	LOC106570236	0 063526991	AX-87455069	17885012	17981917	0
ssa01	25910468	LOC106587081	0 060820813	AX-87871144	25872092	26041001	0
ssa01	42527075	mideasb	0 058229651	AX-87754227	42515390	42547432	0
ssa01	42565830	mideasb	0 059752063	AX-87736120	42515390	42547432	-18399
ssa01	42600987	crip1	0 059752063	AX-87765255	42600764	42611797	0
ssa01	42839684	LOC106602782	0 058227865	AX-87546078	42859252	42865900	19568
ssa01	47078323	LOC106603011	0 058040768	AX-87399098	47011661	47076385	-1939
ssa01	47094833	LOC106603001	0 056938906	AX-87278454	47092791	47109941	0
ssa01	47430423	LOC106602955	0 05795131	AX-87061268	47380865	47473260	0
ssa01	47435997	LOC106602955	0 05795131	AX-87703405	47380865	47473260	0
ssa01	49285065	LOC106604231	0 073410562	AX-87588916	49317050	49346590	31985
ssa01	49315241	LOC106604231	0 073410562	AX-86998955	49317050	49346590	1809
ssa01	49562757	wdfy4	0 071129061	AX-86958517	49354636	49565797	0
ssa01	49615561	LOC106604271	0 071211463	AX-87611269	49590410	49613777	-1785
ssa01	49780423	LOC106604144	0 071129061	AX-87051061	49772292	49835031	0
ssa01	49878569	LOC106604144	0 071129061	AX-87249213	49772292	49835031	-43539
ssa01	50052694	LOC123726755	0 071129061	AX-87378411	50054643	50055197	1949
ssa01	50336182	pprc1	0 058012284	AX-87711620	50313200	50345216	0
ssa01	50439077	LOC106604404	0 05719735	AX-86907574	50428820	50463502	0
ssa01	50730606	LOC106604449	0 062046931	AX-87852407	50730803	50760977	197
ssa01	50841941	LOC106604452	0 062046931	AX-86992511	50839506	50840535	-1407
ssa01	50872543	LOC106604421	0 065789976	AX-87270787	50880855	50999611	8312
ssa01	50881811	LOC106604421	0 068424672	AX-87191078	50880855	50999611	0
ssa01	50989350	LOC106604421	0 068424672	AX-87466867	50880855	50999611	0
ssa01	51028548	cutc	0 062001982	AX-87566793	51019791	51029983	0
ssa01	51122918	LOC106604487	0 062736956	AX-87841790	51134010	51167912	11092
ssa01	51123929	LOC106604487	0 062736956	AX-87859879	51134010	51167912	10081
ssa01	51124219	LOC106604487	0 063588643	AX-86955577	51134010	51167912	9791
ssa01	51124457	LOC106604487	0 062736956	AX-87780910	51134010	51167912	9553
ssa01	51163279	LOC106604487	0 062736956	AX-87586634	51134010	51167912	0
ssa01	51166835	LOC106604487	0 062736956	AX-87482156	51134010	51167912	0
ssa01	51167304	LOC106604487	0 062736956	AX-87742618	51134010	51167912	0

ssa01	51185089	LOC106604506	0 062736956	AX-87454829	51185069	51188474	0
ssa01	51186812	LOC106604506	0 062736956	AX-87708945	51185069	51188474	0
ssa01	51187265	LOC106604506	0 062736956	AX-86970997	51185069	51188474	0
ssa01	51236127	LOC106604510	0 060116331	AX-87105803	51229427	51237682	0
ssa01	51254278	LOC106604523	0 061049983	AX-87192184	51257696	51270396	3418
ssa01	51255755	LOC106604523	0 061049983	AX-87073730	51257696	51270396	1941
ssa01	51257031	LOC106604523	0 061049983	AX-86913002	51257696	51270396	665
ssa01	51270509	LOC106604523	0 059770682	AX-87155100	51257696	51270396	-114
ssa01	51311235	LOC106604517	0 065098043	AX-87734876	51270984	51319224	0
ssa01	51324855	LOC106604517	0 060996787	AX-87179595	51270984	51319224	-5632
ssa01	51420632	LOC123743198	0 071951899	AX-87753209	51420317	51428601	0
ssa01	51453704	LOC106604550	0 061745611	AX-87484496	51444504	51456371	0
ssa01	51456424	LOC106604550	0 062098595	AX-87382269	51444504	51456371	-54
ssa01	51466716	LOC106604566	0 063714275	AX-87504051	51459504	51487254	0
ssa01	51469074	LOC106604566	0 061291283	AX-87437394	51459504	51487254	0
ssa01	51478764	LOC106604566	0 061291283	AX-87216261	51459504	51487254	0
ssa01	51480105	LOC106604566	0 063714275	AX-87137509	51459504	51487254	0
ssa01	51480811	LOC106604566	0 061291283	AX-87354044	51459504	51487254	0
ssa01	51516806	LOC106605279	0 061291283	AX-87520867	51508206	51509052	-7755
ssa01	51518484	LOC106605279	0 061291283	AX-87019853	51508206	51509052	-9433
ssa01	51518629	LOC106605279	0 061291283	AX-87695934	51508206	51509052	-9578
ssa01	51641388	LOC106604587	0 063165438	AX-87070309	51632992	51649703	0
ssa01	51694219	LOC106604599	0 057300038	AX-87621055	51654443	51733945	0
ssa01	51699501	LOC106604599	0 066314506	AX-87479232	51654443	51733945	0
ssa01	51754505	LOC106604619	0 063424578	AX-87323552	51737049	51768201	0
ssa01	51809143	LOC106604631	0 057331396	AX-86959643	51806009	51823188	0
ssa01	51818733	LOC106604631	0 063359568	AX-86984367	51806009	51823188	0
ssa01	51851760	LOC106604631	0 059164241	AX-87747643	51806009	51823188	-28573
ssa01	51877248	LOC106604674	0 069271438	AX-87458069	51897363	51931457	20115
ssa01	52065162	LOC123743248	0 060117321	AX-87239262	51964142	52018132	-47031
ssa01	52065468	LOC123743248	0 060117321	AX-87618258	51964142	52018132	-47337
ssa01	52067075	LOC123743248	0 060117321	AX-87131034	51964142	52018132	-48944
ssa01	52067276	LOC123743248	0 058929112	AX-86978019	51964142	52018132	-49145
ssa01	52099584	emx2	0 058543186	AX-87695798	52134743	52139781	35159
ssa01	52102816	emx2	0 07020907	AX-87276333	52134743	52139781	31927
ssa01	52176076	dennd10	0 056583677	AX-87262905	52139856	52178118	0
ssa01	52215395	tm9sf3	0 05922788	AX-87231287	52205253	52224374	0
ssa01	52359001	spata48	0 071275419	AX-87625202	52345138	52366033	0
ssa01	52412150	ikzf1	0 063449145	AX-87369499	52390911	52414160	0
ssa01	52414175	ikzf1	0 061940903	AX-87655639	52390911	52414160	-16
ssa01	52415146	ikzf1	0 062909501	AX-87395630	52390911	52414160	-987
ssa01	52430263	fignl1	0 06696619	AX-87818304	52423701	52433273	0

ssa01	52495465	LOC106604799	0	060765639	AX-87362966	52480142	52481863	-13603
ssa01	52495794	LOC106604799	0	061940903	AX-87709697	52480142	52481863	-13932
ssa01	52495954	LOC106604799	0	059277087	AX-87351532	52480142	52481863	-14092
ssa01	52503294	LOC106604807	0	069130285	AX-87317995	52521945	52538494	18651
ssa01	52510006	LOC106604807	0	069130285	AX-87857866	52521945	52538494	11939
ssa01	52510021	LOC106604807	0	069130285	AX-86955176	52521945	52538494	11924
ssa01	52547626	LOC106604820	0	060657059	AX-87301679	52548399	52642565	773
ssa01	52548066	LOC106604820	0	060657059	AX-87682394	52548399	52642565	333
ssa01	52548499	LOC106604820	0	060657059	AX-86957775	52548399	52642565	0
ssa01	52564210	LOC106604820	0	066727216	AX-87576202	52548399	52642565	0
ssa01	52564735	LOC106604820	0	066727216	AX-87042393	52548399	52642565	0
ssa01	52582540	LOC106604820	0	068763071	AX-87042191	52548399	52642565	0
ssa01	52589078	LOC106604820	0	068763071	AX-87305258	52548399	52642565	0
ssa01	52658300	LOC106604820	0	062044226	AX-87258286	52548399	52642565	-15736
ssa01	52679974	LOC106604835	0	067647339	AX-87716015	52677815	52686592	0
ssa01	52689217	LOC106604835	0	056922011	AX-86985188	52677815	52686592	-2626
ssa01	52768927	LOC106605317	0	067201106	AX-87506376	52793083	52808290	24156
ssa01	52769649	LOC106605317	0	067909025	AX-87479837	52793083	52808290	23434
ssa01	52770255	LOC106605317	0	066594538	AX-87481281	52793083	52808290	22828
ssa01	52770305	LOC106605317	0	064897894	AX-87147755	52793083	52808290	22778
ssa01	52770518	LOC106605317	0	066594538	AX-86965447	52793083	52808290	22565
ssa01	52824614	LOC106604861	0	057900134	AX-87495673	52810540	52840073	0
ssa01	52832002	LOC106604861	0	056819071	AX-87754508	52810540	52840073	0
ssa01	52851178	LOC106604847	0	056964897	AX-87617726	52845756	52889136	0
ssa01	52852074	LOC106604847	0	056819071	AX-87764200	52845756	52889136	0
ssa01	52872193	LOC106604847	0	062698346	AX-87198100	52845756	52889136	0
ssa01	52888806	LOC106604847	0	068246443	AX-87240289	52845756	52889136	0
ssa01	52904416	march5	0	068922789	AX-87129395	52891566	52944057	0
ssa01	52930699	march5	0	069048164	AX-87600824	52891566	52944057	0
ssa01	52935163	march5	0	061596541	AX-87609167	52891566	52944057	0
ssa01	52989449	cpeb3	0	069551127	AX-87014301	52944137	53022249	0
ssa01	53048851	btaf1	0	065888132	AX-87075595	53024072	53091032	0
ssa01	53075876	btaf1	0	063332676	AX-87179048	53024072	53091032	0
ssa01	53252299	LOC106605322	0	05806696	AX-87159923	53170508	53314314	0
ssa01	53382845	LOC106604920	0	058225571	AX-87866920	53428737	53497162	45892
ssa01	53382855	LOC106604920	0	058225571	AX-87306066	53428737	53497162	45882
ssa01	53394659	LOC106604920	0	058225571	AX-87011129	53428737	53497162	34078
ssa01	53394677	LOC106604920	0	058933332	AX-87501653	53428737	53497162	34060
ssa01	53405729	LOC106604920	0	058225571	AX-86997915	53428737	53497162	23008
ssa01	53442736	LOC106604920	0	058900044	AX-87204831	53428737	53497162	0
ssa01	53660941	LOC106604940	0	059458647	AX-87179029	53658244	53944416	0
ssa01	53695786	LOC106604940	0	073332662	AX-87543264	53658244	53944416	0

ssa01	53697416	LOC106604940	0	068447215	AX-87233148	53658244	53944416	0
ssa01	53699724	LOC106604940	0	068447215	AX-87271720	53658244	53944416	0
ssa01	53719994	LOC106604940	0	073148443	AX-87058951	53658244	53944416	0
ssa01	53745091	LOC106604940	0	060413604	AX-87399031	53658244	53944416	0
ssa01	53749151	LOC106604940	0	05989088	AX-86936361	53658244	53944416	0
ssa01	53765552	LOC106604940	0	067411613	AX-87629790	53658244	53944416	0
ssa01	53940826	LOC106604940	0	061753728	AX-87367709	53658244	53944416	0
ssa01	53941035	LOC106604940	0	077856449	AX-87786884	53658244	53944416	0
ssa01	53945895	LOC106604940	0	060879818	AX-87682162	53658244	53944416	-1480
ssa01	53949139	LOC106604940	0	056800583	AX-87479277	53658244	53944416	-4724
ssa01	53949178	LOC106604940	0	058248454	AX-87355720	53658244	53944416	-4763
ssa01	54005277	LOC106604947	0	074481313	AX-87870169	54003646	54097251	0
ssa01	54010840	LOC106604947	0	064326392	AX-87682443	54003646	54097251	0
ssa01	54042018	LOC106604947	0	064211046	AX-87433791	54003646	54097251	0
ssa01	54097113	LOC106604947	0	066472968	AX-87284797	54003646	54097251	0
ssa01	54176106	LOC106604960	0	066472968	AX-87310681	54186246	54281257	10140
ssa01	54186771	LOC106604960	0	069435141	AX-86976975	54186246	54281257	0
ssa01	54199137	LOC106604960	0	073495074	AX-87449071	54186246	54281257	0
ssa01	54297612	LOC106604960	0	060949413	AX-87065402	54186246	54281257	-16356
ssa01	54300684	LOC106604960	0	058241853	AX-87372258	54186246	54281257	-19428
ssa01	54384440	valopa	0	05965952	AX-87544721	54356849	54380327	-4114
ssa01	54435957	LOC106605012	0	057751449	AX-87720141	54435824	54634715	0
ssa01	54597937	LOC106605012	0	060047882	AX-87153723	54435824	54634715	0
ssa01	54941072	LOC100306744	0	059218242	AX-87269122	54937737	54953195	0
ssa01	55336914	LOC106605192	0	060066005	AX-87457406	55336375	55339745	0
ssa01	55337493	LOC106605192	0	060066005	AX-87215184	55336375	55339745	0
ssa01	55354985	LOC106605192	0	064122147	AX-87648379	55336375	55339745	-15241
ssa01	55608371	trnai-uau	0	062828386	AX-87830893	55647198	55647291	38827
ssa01	55864398	LOC106605225	0	058416381	AX-87250539	55812677	55815852	-48547
ssa01	55865222	LOC106605225	0	058416381	AX-87426543	55812677	55815852	-49371
ssa01	56019498	LOC106605231	0	057974334	AX-87047896	56007732	56101031	0
ssa01	56100384	LOC106605231	0	066802075	AX-87833469	56007732	56101031	0
ssa01	56108517	LOC106605231	0	076434868	AX-87580627	56007732	56101031	-7487
ssa01	56108560	LOC106605231	0	078873539	AX-87041303	56007732	56101031	-7530
ssa01	56138693	LOC106608624	0	075211009	AX-87333357	56126327	56157437	0
ssa01	56156049	LOC106608624	0	073375343	AX-87486911	56126327	56157437	0
ssa01	56459252	LOC106605759	0	056608953	AX-86952454	56433342	56477749	0
ssa01	56459645	LOC106605759	0	056608953	AX-87106564	56433342	56477749	0
ssa01	56464082	LOC106605759	0	056608953	AX-87600286	56433342	56477749	0
ssa01	56467068	LOC106605759	0	056608953	AX-87513674	56433342	56477749	0
ssa01	56467442	LOC106605759	0	056608953	AX-87202749	56433342	56477749	0
ssa01	56478860	LOC106605759	0	06116776	AX-87412094	56433342	56477749	-1112

ssa01	56490469	LOC106605759	0 066995034	AX-87305275	56433342	56477749	-12721
ssa01	56522084	LOC123726801	0 058930778	AX-87155720	56525963	56526017	3879
ssa01	56637553	LOC106605778	0 05900099	AX-87410434	56624390	56645834	0
ssa01	56640846	LOC106605778	0 057932282	AX-87409400	56624390	56645834	0
ssa01	56644677	LOC106605778	0 064758354	AX-86982104	56624390	56645834	0
ssa01	56680798	LOC106605787	0 058831327	AX-87239730	56709114	56813838	28316
ssa01	68460970	rgs7a	0 073625602	AX-87410226	68331699	68457716	-3255
ssa01	71422147	LOC106607988	0 060201197	AX-87744532	71400554	71497889	0
ssa01	73873516	trmt2b	0 062943071	AX-86951098	73851128	73873936	0
ssa01	74837046	LOC123743785	0 057629977	AX-87781043	74533903	74928610	0
ssa01	74837273	LOC123743785	0 057629977	AX-87537752	74533903	74928610	0
ssa01	79345787	LOC106612554	0 061812278	AX-87272545	79295077	79346251	0
ssa01	79403064	mcph1	0 061812278	AX-87590317	79350777	79404242	0
ssa01	79574023	LOC106612532	0 061542281	AX-87160485	79513461	80070143	0
ssa01	94721594	LOC106610511	0 05662073	AX-87133346	94688413	94719523	-2072
ssa01	95602127	LOC106610657	0 061678229	AX-87564480	95584797	95618324	0
ssa01	95602398	LOC106610657	0 062589427	AX-86961260	95584797	95618324	0
ssa01	126847348	LOC106561858	0 058688216	AX-87769680	126809710	126847480	0
ssa01	127991503	LOC106561913	0 058306878	AX-87717612	127902309	128000954	0
ssa01	128020661	LOC106561919	0 058325605	AX-87743562	128015698	128026675	0
ssa01	128020997	LOC106561919	0 057402263	AX-87171082	128015698	128026675	0
ssa01	128091474	LOC106561946	0 057790035	AX-86951654	128106302	128110583	14828
ssa01	138101917	LOC106565454	0 060168299	AX-87310087	138107684	138141393	5767
ssa01	148525207	LOC106567744	0 060712687	AX-87422648	148566509	148589531	41302
ssa01	148666228	LOC106567759	0 064395411	AX-87226102	148660678	148666279	0
ssa01	153663690	LOC106568729	0 067223341	AX-87421123	153658055	153666819	0
ssa01	153663739	LOC106568729	0 067223341	AX-87089014	153658055	153666819	0
ssa01	153672130	LOC106568724	0 068273581	AX-87195851	153668535	153674601	0
ssa01	153740138	LOC106568736	0 06266009	AX-87498689	153705067	153820189	0
ssa01	159355410	si:dkey-220k22.1	0 057574957	AX-87784151	159349721	159433585	0
ssa01	159361932	si:dkey-220k22.1	0 057574957	AX-87618420	159349721	159433585	0
ssa01	159413300	si:dkey-220k22.1	0 060713945	AX-87175990	159349721	159433585	0
ssa01	159427949	si:dkey-220k22.1	0 057451153	AX-87477639	159349721	159433585	0
ssa01	159501499	LOC106570355	0 058429728	AX-87303977	159494184	159506018	0
ssa01	159504440	LOC106570355	0 063941654	AX-87385824	159494184	159506018	0
ssa01	159505153	LOC106570355	0 058429728	AX-87334379	159494184	159506018	0
ssa01	159537525	zgc:55461	0 06464566	AX-87096372	159536608	159542448	0
ssa01	168570390	LOC106572199	0 057030588	AX-87564645	168540210	168554335	-16056
ssa02	8980388	LOC106573289	0 06340708	AX-87549682	8961671	8975329	-5060
ssa02	26578594	ppox	0 060316309	AX-87531473	26574922	26582555	0
ssa02	27590172	LOC106579462	0 063079148	AX-87842279	27581308	27602523	0
ssa02	27593377	LOC106579462	0 063079148	AX-87539666	27581308	27602523	0

ssa02	27603517	LOC106579601	0	058252151	AX-87113766	27602369	27604937	0
ssa02	27671632	LOC106579451	0	057808607	AX-87504473	27664752	27702693	0
ssa02	35261390	LOC106580741	0	057002911	AX-87544685	35242744	35264123	0
ssa02	35261896	LOC106580741	0	060219496	AX-87450076	35242744	35264123	0
ssa02	35262689	LOC106580741	0	057002911	AX-87495567	35242744	35264123	0
ssa02	35263074	LOC106580741	0	060606718	AX-87746335	35242744	35264123	0
ssa02	35276402	LOC106580741	0	062623982	AX-87388915	35242744	35264123	-12280
ssa02	35446632	LOC106580670	0	064326189	AX-87727273	35417459	35451331	0
ssa02	35456356	LOC106580679	0	062482398	AX-87208379	35451439	35473934	0
ssa02	35457080	LOC106580679	0	068543131	AX-87851369	35451439	35473934	0
ssa02	35665002	LOC106581382	0	057116342	AX-87130691	35666599	35671325	1597
ssa02	35747319	LOC106581364	0	057329314	AX-87372735	35723519	35725418	-21902
ssa02	35775286	LOC106581785	0	071355273	AX-87376215	35782884	35804804	7598
ssa02	35801991	LOC106581785	0	060357921	AX-87216912	35782884	35804804	0
ssa02	35803303	LOC106581785	0	063600855	AX-87798713	35782884	35804804	0
ssa02	37194037	LOC106582184	0	060378774	AX-86995274	37185001	37286935	0
ssa02	37209226	LOC106582184	0	061243163	AX-87370757	37185001	37286935	0
ssa02	40240353	LOC106581708	0	061747284	AX-87483424	40245776	40250515	5423
ssa02	52053092	LOC106586159	0	058059384	AX-87445694	52054265	52059222	1173
ssa02	56336928	LOC106587857	0	059360228	AX-87360246	56334196	56348274	0
ssa02	61364139	LOC106586472	0	062335539	AX-87530997	61347569	61404409	0
ssa03	4969097	LOC106596732	0	072163525	AX-87553410	4954544	5054290	0
ssa03	5045066	LOC106596732	0	057120441	AX-87714328	4954544	5054290	0
ssa03	5045288	LOC106596732	0	057120441	AX-87491320	4954544	5054290	0
ssa03	5077203	LOC106596709	0	059923736	AX-87637784	5062494	5077682	0
ssa03	14126724	LOC106598927	0	066227275	AX-87790115	14111135	14130508	0
ssa03	14194848	LOC106598932	0	064028992	AX-87708345	14157904	14200321	0
ssa03	14195665	LOC106598932	0	064028992	AX-87711838	14157904	14200321	0
ssa03	14269371	LOC106598933	0	074647281	AX-87348006	14234279	14277868	0
ssa03	14328709	LOC106598935	0	079415405	AX-87198235	14365750	14376465	37041
ssa03	14365043	LOC106598935	0	060600049	AX-87099264	14365750	14376465	707
ssa03	14409541	pif1	0	062561912	AX-87194509	14399350	14410425	0
ssa03	14456346	ptger4c	0	062893711	AX-87125103	14478640	14480929	22294
ssa03	14481555	LOC106598943	0	062963479	AX-87475923	14480950	14508960	0
ssa03	14504511	LOC106598943	0	060108254	AX-87009739	14480950	14508960	0
ssa03	14507998	LOC106598943	0	059700227	AX-87432219	14480950	14508960	0
ssa03	14545629	LOC106598945	0	060542228	AX-87846122	14525574	14607755	0
ssa03	14555388	LOC106598945	0	073787279	AX-87155397	14525574	14607755	0
ssa03	14555560	LOC106598945	0	073384336	AX-87640147	14525574	14607755	0
ssa03	14557702	LOC106598945	0	073787279	AX-87330006	14525574	14607755	0
ssa03	14561179	LOC106598945	0	073787279	AX-87049174	14525574	14607755	0
ssa03	14561274	LOC106598945	0	073787279	AX-87090229	14525574	14607755	0

ssa03	14568930	LOC106598945	0	073787279	AX-87466668	14525574	14607755	0
ssa03	14569857	LOC106598945	0	072296809	AX-87722656	14525574	14607755	0
ssa03	14569937	LOC106598945	0	073787279	AX-87427434	14525574	14607755	0
ssa03	14574278	LOC106598945	0	073273918	AX-87091230	14525574	14607755	0
ssa03	14583549	LOC106598945	0	057427698	AX-87371406	14525574	14607755	0
ssa03	14604538	LOC106598945	0	062696084	AX-87455958	14525574	14607755	0
ssa03	20956194	LOC106599513	0	06481007	AX-87408966	20854124	20966766	0
ssa03	36446027	LOC106600370	0	060451749	AX-87495678	36409611	36449247	0
ssa03	39615784	LOC106600004	0	062446201	AX-87610340	39557074	39651942	0
ssa03	44506798	LOC106600570	0	060328252	AX-87603560	44500404	44526392	0
ssa03	65768258	LOC106601251	0	064945161	AX-87568521	65769417	65776076	1159
ssa03	65776136	LOC106601251	0	070714516	AX-86941125	65769417	65776076	-61
ssa03	65776239	LOC106601251	0	070714516	AX-87767768	65769417	65776076	-164
ssa03	65776674	LOC106601251	0	070714516	AX-87084651	65769417	65776076	-599
ssa03	79616321	LOC106601829	0	059433098	AX-87857896	79600671	79670416	0
ssa03	80628700	LOC106606620	0	058083411	AX-87656886	80623336	80631793	0
ssa03	80679361	LOC106601740	0	063003422	AX-86906269	80678799	80684937	0
ssa03	80815414	LOC106606607	0	061003854	AX-87174388	80814499	80826156	0
ssa03	80831460	LOC106606606	0	059670181	AX-87767997	80826873	80832356	0
ssa03	80846315	LOC106606605	0	07048894	AX-87183269	80832968	80846870	0
ssa03	86275567	LOC106601932	0	05855048	AX-87546138	86251083	86318974	0
ssa03	100250016	LOC123741859	0	067799709	AX-87393463	100280366	100355672	30350
ssa04	15329073	LOC106602365	0	063677512	AX-87417368	15012815	15347042	0
ssa04	17949219	LOC106602441	0	062988663	AX-86942160	17956542	17998479	7323
ssa04	17949562	LOC106602441	0	060957776	AX-86933048	17956542	17998479	6980
ssa04	17951374	LOC106602441	0	059988264	AX-87365857	17956542	17998479	5168
ssa04	21288192	LOC106602576	0	069826045	AX-87502121	21281973	21287964	-229
ssa04	21684022	LOC106602574	0	060521016	AX-87013691	21681729	21691315	0
ssa04	21852844	LOC106602568	0	074812167	AX-87781458	21847845	21852700	-145
ssa04	21871591	LOC106602571	0	070602999	AX-87230615	21868162	21871230	-362
ssa04	21897745	LOC106602565	0	066346478	AX-87748135	21892407	21913042	0
ssa04	27091283	LOC106602757	0	059184942	AX-87065866	26452345	27122650	0
ssa04	27099486	LOC106602757	0	059239796	AX-87734396	26452345	27122650	0
ssa04	27867830	dchs2	0	057532785	AX-87366367	27846242	27903429	0
ssa04	46826550	LOC106603277	0	06910829	AX-87203240	46642057	46896686	0
ssa04	46827089	LOC106603277	0	068777803	AX-87130526	46642057	46896686	0
ssa04	47158034	LOC106603282	0	068992371	AX-87085834	47096774	47159494	0
ssa04	47160121	LOC106603282	0	062345873	AX-87287334	47096774	47159494	-628
ssa04	47168761	LOC106603284	0	06998231	AX-87185622	47160829	47176518	0
ssa04	47315854	nf1b	0	070747112	AX-87672031	47300222	47373907	0
ssa04	49118196	fdx1b	0	056633228	AX-87861235	49116939	49119919	0
ssa04	49118628	fdx1b	0	056633228	AX-87824167	49116939	49119919	0

ssa04	51601691	rb1	0 063521807	AX-87274429	51574114	51611281	0
ssa04	51602237	rb1	0 05853593	AX-87147102	51574114	51611281	0
ssa04	51602499	rb1	0 05853593	AX-87329823	51574114	51611281	0
ssa04	51605368	rb1	0 058459811	AX-87140411	51574114	51611281	0
ssa04	51619788	slc6a7	0 05729612	AX-87807909	51611910	51618946	-843
ssa04	51759447	LOC123742580	0 063475647	AX-86961381	51769676	51771201	10229
ssa04	51778902	rn167	0 064414152	AX-87594507	51782727	51799502	3825
ssa04	51781388	rn167	0 064164525	AX-87610977	51782727	51799502	1339
ssa04	51843483	LOC106603400	0 057800123	AX-87812050	51843388	51889573	0
ssa04	52383245	LOC106603592	0 064367702	AX-87759619	52366789	52369853	-13393
ssa04	52403831	LOC106603592	0 057959571	AX-87685813	52366789	52369853	-33979
ssa04	52502725	LOC106603590	0 058386926	AX-87243597	52495303	52511725	0
ssa04	52504027	LOC106603590	0 058386926	AX-87714068	52495303	52511725	0
ssa04	52539964	LOC106603589	0 065745381	AX-87451619	52550290	52599611	10326
ssa04	52551950	LOC106603589	0 065745381	AX-87293345	52550290	52599611	0
ssa04	52555111	LOC106603589	0 065745381	AX-87602325	52550290	52599611	0
ssa04	53094784	LOC106603565	0 059624338	AX-87398765	53088826	53109756	0
ssa04	58167911	LOC106603609	0 06528441	AX-87587087	58143073	58144324	-23588
ssa04	58181814	LOC106603609	0 064462175	AX-87010256	58143073	58144324	-37491
ssa04	58184364	LOC106603609	0 064462175	AX-87326788	58143073	58144324	-40041
ssa04	88897224	kpna7	0 061549513	AX-86996473	88891925	88899305	0
ssa04	89741675	LOC106604063	0 060704838	AX-87177438	89662451	89749830	0
ssa05	4646447	LOC100196664	0 060405974	AX-87625258	4663575	4737626	17128
ssa05	35999426	LOC100194849	0 06458179	AX-86951465	35951294	36141393	0
ssa05	35999979	LOC100194849	0 06458179	AX-87147341	35951294	36141393	0
ssa05	36390769	LOC106604745	0 05921346	AX-86999079	36418015	36423773	27246
ssa05	36402135	LOC106604745	0 05921346	AX-87669806	36418015	36423773	15880
ssa05	38332508	LOC106604876	0 066126311	AX-86973759	38305488	38496354	0
ssa05	38332508	LOC106604822	0 066126311	AX-86973759	38331687	38334248	0
ssa05	38332919	LOC106604876	0 068276798	AX-86969062	38305488	38496354	0
ssa05	38332919	LOC106604822	0 068276798	AX-86969062	38331687	38334248	0
ssa05	39881930	LOC106604841	0 057072508	AX-87085387	39879751	39886571	0
ssa05	53624621	LOC106605177	0 056798755	AX-87567565	53637726	53639029	13105
ssa05	53637519	LOC106605177	0 056798755	AX-87195098	53637726	53639029	207
ssa05	53686667	LOC106605120	0 056798755	AX-86918458	53682193	53687134	0
ssa05	59226625	LOC106605316	0 064840587	AX-87344569	59174863	59247509	0
ssa05	64365410	LOC100194674	0 063437577	AX-87046267	64211975	64370554	0
ssa05	64373953	LOC106605546	0 064627202	AX-87082810	64371247	64379223	0
ssa05	64920921	LOC106605539	0 057905432	AX-87375293	64917234	64923323	0
ssa05	67552418	LOC106605640	0 065089912	AX-87179286	67551240	67575698	0
ssa05	67611211	LOC106605668	0 074203091	AX-86918449	67610015	67618493	0
ssa05	67658095	LOC106605587	0 061349345	AX-87134081	67656494	67693733	0

ssa05	67668528	LOC106605587	0 064398841	AX-87363144	67656494	67693733	0
ssa05	67686551	LOC106605587	0 064447717	AX-87308517	67656494	67693733	0
ssa05	68360795	nphs1	0 072606206	AX-87124089	68260599	68323414	-37382
ssa05	68363974	nphs1	0 073586263	AX-87531652	68260599	68323414	-40561
ssa05	86137908	LOC106574054	0 072001765	AX-87697856	85987377	86168860	0
ssa06	7815543	LOC106592621	0 062084198	AX-87129756	7803988	7822188	0
ssa06	13553112	LOC106606358	0 068738144	AX-87523043	13546569	13552878	-235
ssa06	14839774	LOC106606423	0 076326771	AX-87346703	14768033	14875584	0
ssa06	15142074	roa2	0 060460512	AX-87857109	15127122	15129695	-12380
ssa06	15149368	LOC106606518	0 060460512	AX-86909047	15156461	15158039	7093
ssa06	15618378	LOC106606438	0 057064375	AX-87007718	15539642	15617594	-785
ssa06	15816299	LOC106606445	0 075269635	AX-87244760	15823719	15825059	7420
ssa06	16682428	LOC106606408	0 068121744	AX-87372637	16730060	16744503	47632
ssa06	16908522	LOC106606458	0 068750369	AX-87140148	16897462	16899071	-9452
ssa06	17004602	LOC106606457	0 073453157	AX-86920138	16980848	16982453	-22150
ssa06	17392881	LOC106606471	0 065528048	AX-87048363	17379342	17477159	0
ssa06	17398626	LOC106606471	0 059908708	AX-87517920	17379342	17477159	0
ssa06	17401343	LOC106606471	0 065528048	AX-87500761	17379342	17477159	0
ssa06	22411786	LOC106606650	0 059338489	AX-87327894	22400896	22419798	0
ssa06	32439651	LOC106606999	0 05825058	AX-87546664	32455840	32457590	16189
ssa06	32517010	LOC106606982	0 059848306	AX-87474096	32511023	32522564	0
ssa06	33369920	rbp	0 072582864	AX-87496283	33373814	33383336	3894
ssa06	33375180	rbp	0 07417808	AX-87285861	33373814	33383336	0
ssa06	33383639	LOC123743536	0 074189285	AX-87524529	33383112	33391785	0
ssa06	33468039	LOC106607035	0 057577672	AX-87434787	33467743	33469795	0
ssa06	33468322	LOC106607035	0 057577672	AX-86960545	33467743	33469795	0
ssa06	33526087	LOC100380669	0 0587027	AX-87370301	33510298	33542762	0
ssa06	34660425	LOC106601199	0 069431318	AX-87358382	34601492	34663762	0
ssa06	34674614	bglap	0 068025301	AX-87767050	34674738	34676510	124
ssa06	38617331	LOC106607349	0 057381348	AX-87029590	38601535	38692190	0
ssa06	44016451	hoxb3ab	0 057573359	AX-87153930	43961239	44018971	0
ssa06	44292601	LOC106607485	0 06221263	AX-87808336	44336130	44369665	43529
ssa06	44344131	LOC106607485	0 061934768	AX-87794685	44336130	44369665	0
ssa06	44357589	LOC106607485	0 061934768	AX-87419420	44336130	44369665	0
ssa06	44373565	LOC106607482	0 061934768	AX-87567338	44376718	44390816	3153
ssa06	44388016	LOC106607482	0 058765908	AX-87130159	44376718	44390816	0
ssa06	46591315	LOC106607452	0 061245763	AX-87839174	46591513	46611626	198
ssa06	46598978	LOC106607452	0 061091279	AX-87320260	46591513	46611626	0
ssa06	47277317	LOC106607426	0 062197123	AX-87167634	47261238	47289315	0
ssa06	58875559	LOC106607720	0 062123763	AX-87733676	58878061	58950577	2502
ssa06	60007610	dcam	0 058660479	AX-87152072	59991794	60015688	0
ssa06	60009271	dcam	0 07092251	AX-87677819	59991794	60015688	0

ssa06	60014912	dcam	0 059765005	AX-87486829	59991794	60015688	0
ssa06	60015278	dcam	0 066864568	AX-87458834	59991794	60015688	0
ssa06	60017046	cd2l6	0 062203366	AX-87357102	60016502	60027814	0
ssa06	60075570	LOC106607744	0 06180697	AX-86979445	60093556	60166633	17986
ssa06	60468632	LOC106607909	0 058252391	AX-87667288	60487636	60489355	19004
ssa06	60600635	ptprk	0 058788318	AX-87137443	60588002	60744411	0
ssa06	60603744	ptprk	0 057765305	AX-87104257	60588002	60744411	0
ssa06	60606762	ptprk	0 057765305	AX-87277549	60588002	60744411	0
ssa06	62735950	slc35f1	0 060561329	AX-87848645	62686482	62809498	0
ssa06	62737131	slc35f1	0 060561329	AX-87096284	62686482	62809498	0
ssa06	62743817	slc35f1	0 075077443	AX-87402776	62686482	62809498	0
ssa06	67020235	LOC106607880	0 068444377	AX-86973082	67004648	67007232	-13004
ssa06	67047707	LOC123743558	0 058688729	AX-87395736	67036787	67037734	-9974
ssa06	69696089	fam110c	0 060861175	AX-86974599	69695965	69698298	0
ssa06	69708136	fam110c	0 058358204	AX-87553427	69695965	69698298	-9839
ssa06	71810156	LOC106607985	0 062334848	AX-87570908	71606360	71923218	0
ssa06	84694868	LOC106608234	0 069790129	AX-87107950	84694805	84728758	0
ssa06	84910284	LOC106608241	0 074356739	AX-87067476	84903964	84925983	0
ssa06	84930748	LOC106608241	0 079069061	AX-87337741	84903964	84925983	-4766
ssa06	85019999	LOC106608246	0 082705538	AX-87238979	84954909	85022803	0
ssa06	85048680	LOC106608248	0 08308275	AX-87764995	85048437	85049659	0
ssa06	85123049	trnae-uuc	0 077023148	AX-87626252	85152883	85152957	29834
ssa06	85123688	trnae-uuc	0 079098906	AX-87521825	85152883	85152957	29195
ssa06	85136072	trnae-uuc	0 083129762	AX-87150392	85152883	85152957	16811
ssa06	85136175	trnae-uuc	0 083139523	AX-86982270	85152883	85152957	16708
ssa06	85138495	trnae-uuc	0 078372426	AX-86934667	85152883	85152957	14388
ssa06	85184018	trnae-uuc	0 085306591	AX-87121609	85152883	85152957	-31062
ssa06	85446858	LOC106608231	0 071222094	AX-87114111	85415453	85447366	0
ssa06	85446858	LOC106608232	0 071222094	AX-87114111	85446234	85460189	0
ssa06	85459498	LOC106608232	0 080229416	AX-87619936	85446234	85460189	0
ssa06	85460247	LOC106608232	0 080229416	AX-87626550	85446234	85460189	-59
ssa06	85492809	actc1a	0 077428688	AX-87645832	85492375	85500250	0
ssa06	85495496	actc1a	0 083110224	AX-87710566	85492375	85500250	0
ssa06	85506189	LOC106608260	0 080229416	AX-87172230	85500813	85516729	0
ssa06	85555954	LOC106608256	0 078421291	AX-87101438	85518278	85565762	0
ssa06	85562033	LOC106608256	0 078421291	AX-87358742	85518278	85565762	0
ssa06	85564157	LOC106608256	0 07190515	AX-87171805	85518278	85565762	0
ssa06	85614937	LOC106608253	0 070832431	AX-86995143	85589751	85878495	0
ssa06	85621344	LOC106608253	0 06883407	AX-86910906	85589751	85878495	0
ssa06	93831650	sec63	0 058963312	AX-86934315	93839187	93878942	7537
ssa06	94578709	LOC106608381	0 068219706	AX-86950881	94561926	94596713	0
ssa06	94798135	LOC106591735	0 058203932	AX-87008684	94802916	94848679	4781

ssa07	5672454	4f2	0 059100467	AX-87090450	5650885	5672798	0
ssa07	6252534	LOC106608499	0 065489775	AX-87085943	6251894	6253417	0
ssa07	6253134	LOC106608499	0 06039723	AX-87178124	6251894	6253417	0
ssa07	6264417	LOC106608492	0 060489007	AX-86965136	6263792	6266961	0
ssa07	11170685	LOC106608549	0 071640654	AX-87722405	11118872	11188889	0
ssa07	11363109	LOC106608564	0 063284961	AX-87216043	11318593	11819182	0
ssa07	13097423	rab1ba	0 063361926	AX-87184704	13096444	13113326	0
ssa07	18240395	LOC106608611	0 065971382	AX-87685154	18183383	18242838	0
ssa07	18700130	LOC106608606	0 067883964	AX-87649707	18593920	19250220	0
ssa07	19002548	LOC106608606	0 058330152	AX-87563644	18593920	19250220	0
ssa07	23271008	serpinh2	0 073186866	AX-87729219	23298844	23320301	27836
ssa07	25786522	LOC106608915	0 0782051	AX-87218346	25780263	25785800	-723
ssa07	25847483	LOC106608914	0 065801071	AX-86991630	25847475	25854268	0
ssa07	25861752	LOC123723867	0 069643864	AX-87691998	25853810	25878210	0
ssa07	25897170	LOC106609008	0 074382001	AX-87034538	25897829	25900333	659
ssa07	25899200	LOC106609008	0 061528398	AX-87761595	25897829	25900333	0
ssa07	25900373	LOC106609008	0 06159115	AX-87636148	25897829	25900333	-41
ssa07	26049668	LOC106609004	0 065826332	AX-87356681	26048351	26075966	0
ssa07	29250142	col4a5	0 065624275	AX-87284009	29213666	29301247	0
ssa07	38920941	LOC106609220	0 072167844	AX-87014123	38889439	39030835	0
ssa07	41656240	LOC106609322	0 066501564	AX-87583886	41652892	41698206	0
ssa07	46383749	LOC106609461	0 057490233	AX-87010212	46372961	46400889	0
ssa07	46398931	LOC106609461	0 058907709	AX-87164189	46372961	46400889	0
ssa07	56520352	LOC106609740	0 064679069	AX-87199947	56148514	56537451	0
ssa07	56780134	LOC106609713	0 06904667	AX-87526334	56759160	56834010	0
ssa08	1332925	LOC106610019	0 062512966	AX-87794559	1325433	1371085	0
ssa08	1884307	LOC106610026	0 057410857	AX-86934781	1804110	1844225	-40083
ssa08	7382808	LOC106609886	0 069793827	AX-87177687	7381162	7420160	0
ssa08	20480267	LOC106610417	0 05880345	AX-87865405	20481643	20488996	1376
ssa09	4082127	LOC106610601	0 059213336	AX-87792560	3900633	4128102	0
ssa09	5760269	LOC106610595	0 061186172	AX-87307861	5564353	5965997	0
ssa09	14996184	LOC123744595	0 06378174	AX-87573938	14991357	14997678	0
ssa09	15138888	LOC106610776	0 066783308	AX-87026438	15128571	15316214	0
ssa09	15150337	LOC106610776	0 057268903	AX-87492022	15128571	15316214	0
ssa09	15269169	LOC106610776	0 061760951	AX-87024614	15128571	15316214	0
ssa09	15689731	LOC106610785	0 065637938	AX-87831648	15561820	15791063	0
ssa09	15721760	LOC106610785	0 066120964	AX-87593227	15561820	15791063	0
ssa09	15733161	LOC106610785	0 067481639	AX-87277051	15561820	15791063	0
ssa09	15756866	LOC106610785	0 065596581	AX-87424630	15561820	15791063	0
ssa09	21754121	LOC106610924	0 05799764	AX-87812245	21750823	21755625	0
ssa09	21793314	coq6	0 060294366	AX-87425662	21783300	21793975	0
ssa09	22242179	LOC106611017	0 088732507	AX-87704181	22222406	22224582	-17598

ssa09	22269835	LOC106611016	0 084291006	AX-87354752	22273227	22300308	3392
ssa09	22291985	LOC106611016	0 077553419	AX-87569125	22273227	22300308	0
ssa09	22296211	LOC106611016	0 075096069	AX-87529693	22273227	22300308	0
ssa09	22301310	LOC106611016	0 072399966	AX-87015664	22273227	22300308	-1003
ssa09	22310284	LOC106611015	0 078780736	AX-87393995	22307016	22350772	0
ssa09	22350955	msh4	0 088353927	AX-87160155	22350867	22357315	0
ssa09	22353122	msh4	0 089769604	AX-87002255	22350867	22357315	0
ssa09	22353853	msh4	0 089375892	AX-87712774	22350867	22357315	0
ssa09	22393873	LOC106611014	0 08884258	AX-86972967	22400544	22403654	6671
ssa09	22393917	LOC106611014	0 088318076	AX-87768534	22400544	22403654	6627
ssa09	22603335	LOC106610923	0 059213629	AX-87155494	22536118	22633603	0
ssa09	22901882	LOC106611090	0 062431662	AX-87553789	22891360	22903392	0
ssa09	22901993	LOC106611090	0 062431662	AX-87341718	22891360	22903392	0
ssa09	22902298	LOC106611090	0 078366756	AX-87780747	22891360	22903392	0
ssa09	23012391	LOC106611010	0 063844228	AX-86960367	23009243	23014493	0
ssa09	23012391	LOC123744602	0 063844228	AX-86960367	23011756	23015520	0
ssa09	23015868	serpina10b	0 066046878	AX-87869952	23014616	23016869	0
ssa09	23018260	ddx24	0 066159116	AX-87298117	23017047	23021538	0
ssa09	23019366	ddx24	0 060262248	AX-87381501	23017047	23021538	0
ssa09	23019563	ddx24	0 06090399	AX-87700068	23017047	23021538	0
ssa09	23024588	ddx24	0 060868481	AX-87073403	23017047	23021538	-3051
ssa09	23024924	si:ch1073-416d2.4	0 059174393	AX-87386372	23027828	23040640	2904
ssa09	23028663	si:ch1073-416d2.4	0 058557644	AX-86966106	23027828	23040640	0
ssa09	23044741	LOC106611003	0 059617204	AX-87458884	23040706	23052280	0
ssa09	23050842	LOC106611003	0 06150747	AX-87360820	23040706	23052280	0
ssa09	23075418	prima1	0 061418785	AX-87704113	23062998	23081064	0
ssa09	23329474	LOC106610995	0 059105165	AX-87079662	23328226	23339098	0
ssa09	24102226	LOC106610969	0 057476246	AX-87357627	24085299	24139342	0
ssa09	24102268	LOC106610969	0 057357266	AX-87458123	24085299	24139342	0
ssa09	24298864	wdr89	0 067409102	AX-87269348	24298076	24302128	0
ssa09	24298864	2a5e	0 067409102	AX-87269348	24298106	24378183	0
ssa09	24430995	LOC106610918	0 061009356	AX-87778387	24421752	24514264	0
ssa09	24443500	LOC106610918	0 059446669	AX-87525393	24421752	24514264	0
ssa09	24450955	LOC106610918	0 062573002	AX-87501289	24421752	24514264	0
ssa09	29202759	LOC106611167	0 063042919	AX-86935308	29180373	29365397	0
ssa09	29372733	LOC106611167	0 067973876	AX-87312935	29180373	29365397	-7337
ssa09	29388310	LOC106611167	0 065532047	AX-87001877	29180373	29365397	-22914
ssa09	29402082	LOC106611167	0 06893445	AX-87787636	29180373	29365397	-36686
ssa09	29498631	LOC106611216	0 057203231	AX-87634104	29511103	29511744	12472
ssa09	29542715	LOC123724121	0 057203231	AX-87856735	29546386	29547908	3671
ssa09	29566892	LOC123724121	0 057203231	AX-87147003	29546386	29547908	-18985
ssa09	29589717	LOC123724121	0 059927835	AX-87659501	29546386	29547908	-41810

ssa09	29590290	LOC123724121	0 063609862	AX-87510072	29546386	29547908	-42383
ssa09	29590985	LOC123724121	0 063609862	AX-87083965	29546386	29547908	-43078
ssa09	29591695	LOC123724121	0 062508259	AX-87793274	29546386	29547908	-43788
ssa09	29701773	calm1a	0 071533098	AX-87511604	29691049	29709198	0
ssa09	29717996	calm1a	0 070270199	AX-87832704	29691049	29709198	-8799
ssa09	29839254	brms1la	0 074630526	AX-87014633	29838835	29842669	0
ssa09	29868201	LOC106611173	0 074605442	AX-87184646	29843915	29967519	0
ssa09	29956838	LOC106611173	0 061661821	AX-87802137	29843915	29967519	0
ssa09	58286840	ocrl	0 065754863	AX-87445431	58252421	58288375	0
ssa09	60432412	LOC106611555	0 071858478	AX-87365243	60369916	60525639	0
ssa09	60488298	LOC106611555	0 07311612	AX-87545991	60369916	60525639	0
ssa09	64584568	LOC106611617	0 064029533	AX-87256953	64580690	64583019	-1550
ssa09	79234833	LOC106612010	0 067675046	AX-87842147	79274215	79277587	39382
ssa09	79661425	LOC106611959	0 057490892	AX-86982344	79482206	79818139	0
ssa09	79663128	LOC106611959	0 061248911	AX-87328182	79482206	79818139	0
ssa09	79685449	LOC106611959	0 061248911	AX-87821450	79482206	79818139	0
ssa09	79685700	LOC106611959	0 061248911	AX-87048540	79482206	79818139	0
ssa09	93730407	smad5	0 061432704	AX-87339922	93716010	93730257	-151
ssa09	114802716	LOC106612494	0 056891027	AX-87838050	114783431	114889387	0
ssa09	114834851	LOC106612494	0 05721395	AX-87636493	114783431	114889387	0
ssa09	114864053	LOC106612494	0 057356214	AX-87152106	114783431	114889387	0
ssa09	114865609	LOC106612494	0 074153088	AX-87522984	114783431	114889387	0
ssa09	140407518	LOC106613135	0 058573021	AX-87374457	140378799	140474914	0
ssa09	152855283	LOC106613290	0 061596643	AX-87536213	152855290	152859103	7
ssa09	152857111	LOC106613290	0 057975349	AX-87866616	152855290	152859103	0
ssa09	152857228	LOC106613290	0 056628019	AX-87120953	152855290	152859103	0
ssa09	153883932	LOC106613308	0 057812415	AX-87869473	153852613	153878971	-4962
ssa10	15032476	LOC106613576	0 06294967	AX-87854340	15025200	15051123	0
ssa10	15032617	LOC106613576	0 065150099	AX-87037736	15025200	15051123	0
ssa10	15034691	LOC106613576	0 06294967	AX-87085071	15025200	15051123	0
ssa10	23672820	sema6bb	0 064326591	AX-87762605	23613856	23749590	0
ssa10	24453500	LOC106613840	0 070799137	AX-86948855	24443451	24515571	0
ssa10	24484532	LOC106613840	0 070799137	AX-87116720	24443451	24515571	0
ssa10	24536748	LOC106613839	0 077363163	AX-87774495	24525832	24554669	0
ssa10	24544258	LOC106613839	0 070957319	AX-87563403	24525832	24554669	0
ssa10	24559468	LOC106613842	0 079030721	AX-87304576	24556865	24587848	0
ssa10	24569986	LOC106613842	0 071798306	AX-87724151	24556865	24587848	0
ssa10	24692327	LOC106613847	0 057761606	AX-87067008	24688813	24694665	0
ssa10	24828284	LOC106613850	0 058444348	AX-87430636	24821009	24828494	0
ssa10	26426021	bcar3	0 059264209	AX-87099243	26418021	26507295	0
ssa10	26474655	bcar3	0 056864443	AX-86957156	26418021	26507295	0
ssa10	36155439	LOC106560309	0 057241564	AX-87867447	36033314	36195922	0

ssa10	37610456	ptprfa	0	057986055	AX-87247115	37551748	37922740	0
ssa10	61408210	LOC106560735	0	072635419	AX-86908574	61304916	61425977	0
ssa10	65219035	si:ch211-236l14.4	0	056720068	AX-87365471	65218298	65308163	0
ssa10	81021328	LOC123724974	0	0608372	AX-87131029	80989155	80989207	-32122
ssa10	81061415	LOC123724775	0	0608372	AX-87829061	81092496	81092636	31081
ssa10	81092276	LOC123724775	0	063069164	AX-87032202	81092496	81092636	220
ssa10	81092326	LOC123724775	0	066601722	AX-86942769	81092496	81092636	170
ssa10	81092455	LOC123724775	0	066601722	AX-87353577	81092496	81092636	41
ssa10	81667322	LOC106560972	0	071956588	AX-86961322	81603503	81712011	0
ssa10	81672533	LOC106560972	0	067320609	AX-87485257	81603503	81712011	0
ssa10	81673980	LOC106560972	0	067486518	AX-87630680	81603503	81712011	0
ssa10	81706144	LOC106560972	0	067390052	AX-87276626	81603503	81712011	0
ssa10	84297593	vps9d1	0	057785311	AX-87242346	84277865	84308104	0
ssa10	86143490	LOC106560943	0	05809552	AX-87025303	86174389	86185353	30899
ssa10	86145074	LOC106560943	0	05809552	AX-87666725	86174389	86185353	29315
ssa10	86170708	LOC106560943	0	062213957	AX-87838709	86174389	86185353	3681
ssa10	86183701	LOC106560943	0	059284277	AX-86947241	86174389	86185353	0
ssa10	86187872	LOC106560868	0	061119199	AX-87427676	86187953	86193284	81
ssa10	86189418	LOC106560868	0	069916639	AX-86976728	86187953	86193284	0
ssa10	86190922	LOC106560868	0	061119199	AX-87491660	86187953	86193284	0
ssa10	86190941	LOC106560868	0	086162496	AX-87325300	86187953	86193284	0
ssa10	86191483	LOC106560868	0	061407404	AX-87826075	86187953	86193284	0
ssa10	86198087	LOC106560867	0	061506943	AX-87514808	86193160	86332516	0
ssa10	95149791	LOC106561231	0	064886915	AX-87445868	95160650	95577958	10859
ssa10	111464932	LOC106561543	0	064044312	AX-87395566	111461477	111497076	0
ssa10	111471210	LOC106561543	0	072116064	AX-87135199	111461477	111497076	0
ssa10	111490445	LOC106561543	0	071584735	AX-87072796	111461477	111497076	0
ssa10	111505105	LOC100380677	0	065197059	AX-87471951	111505442	111554069	337
ssa10	111507762	LOC100380677	0	065372242	AX-87177833	111505442	111554069	0
ssa10	111507845	LOC100380677	0	061019575	AX-87559904	111505442	111554069	0
ssa10	111508816	LOC100380677	0	0629864	AX-87209839	111505442	111554069	0
ssa10	111509253	LOC100380677	0	065372242	AX-87241273	111505442	111554069	0
ssa10	111608262	LOC106561546	0	06587705	AX-87551072	111575032	111608909	0
ssa10	122954625	LOC106593086	0	068846871	AX-87476067	122928125	123008826	0
ssa10	123015604	sergef	0	063268468	AX-87520909	123015071	123086061	0
ssa11	11926156	phkb	0	05675646	AX-87501867	11914865	11973733	0
ssa11	11959712	phkb	0	06090782	AX-86964376	11914865	11973733	0
ssa11	11976667	LOC106561994	0	065620775	AX-87363906	11973913	12104656	0
ssa11	16314102	LOC106562146	0	057550995	AX-87746439	16204859	16332215	0
ssa11	22970532	LOC123725241	0	065256293	AX-87110627	22955009	22969307	-1226
ssa11	22970886	LOC123725241	0	065256293	AX-87323360	22955009	22969307	-1580
ssa11	22977239	LOC123725241	0	065256293	AX-87855991	22955009	22969307	-7933

ssa11	23141908	LOC106562299	0 069553228	AX-87454525	23172164	23319420	30256
ssa11	23157887	LOC106562299	0 069553228	AX-87264190	23172164	23319420	14277
ssa11	23162900	LOC106562299	0 069553228	AX-87518187	23172164	23319420	9264
ssa11	23166666	LOC106562299	0 065544626	AX-87174650	23172164	23319420	5498
ssa11	24324008	LOC106563532	0 065638489	AX-87857923	24322715	24343506	0
ssa11	24326124	LOC106563532	0 065638489	AX-87244560	24322715	24343506	0
ssa11	24366123	LOC106563530	0 070383572	AX-87294777	24349661	24376766	0
ssa11	28182244	LOC123725251	0 059092695	AX-87411131	28162306	28162847	-19398
ssa11	28652552	LOC106562393	0 061224277	AX-87234319	28692635	28753589	40083
ssa11	72548873	LOC106563040	0 068119812	AX-87357553	72548400	72558986	0
ssa11	72653191	LOC106563036	0 057016244	AX-87719990	72598837	72679765	0
ssa11	74093403	LOC106563143	0 058355063	AX-87534094	74066868	74204117	0
ssa11	74464777	LOC106563138	0 059283052	AX-87087725	74394480	74733846	0
ssa11	75085815	trnap-ugg	0 060806719	AX-87102372	75130025	75130102	44210
ssa11	79240678	slc30a5	0 06886633	AX-87466267	79235400	79241717	0
ssa11	79243465	ccnb1	0 06886633	AX-87755997	79242466	79245798	0
ssa11	79264843	ccnb1	0 068805673	AX-87565829	79242466	79245798	-19046
ssa11	79277743	LOC106563365	0 059316092	AX-87236203	79288610	79289250	10867
ssa11	79550018	LOC106563300	0 057360804	AX-87617462	79543813	79550604	0
ssa11	83948184	LOC106563245	0 061332957	AX-86985827	83902292	84024245	0
ssa11	88076430	npm	0 068091067	AX-87696971	88072680	88077073	0
ssa11	88076495	npm	0 067040746	AX-87578991	88072680	88077073	0
ssa11	88089268	LOC106563176	0 07080549	AX-87229897	88081469	88089254	-15
ssa11	88179912	cldx	0 061233386	AX-87534318	88175829	88183032	0
ssa11	88297185	LOC106563170	0 062332071	AX-87261963	88220293	88298702	0
ssa11	88298276	LOC106563170	0 063416232	AX-87602467	88220293	88298702	0
ssa11	89058014	LOC106563393	0 056992246	AX-87674533	89056978	89072402	0
ssa11	89246483	LOC106563399	0 061712987	AX-87047324	89263237	89396380	16754
ssa11	89285145	LOC106563399	0 059429907	AX-87163563	89263237	89396380	0
ssa11	89298845	LOC106563399	0 05692872	AX-87097609	89263237	89396380	0
ssa11	89300862	LOC106563399	0 05666522	AX-86998921	89263237	89396380	0
ssa11	89325874	LOC106563399	0 056732884	AX-87274238	89263237	89396380	0
ssa11	89381778	LOC106563399	0 05692872	AX-87575899	89263237	89396380	0
ssa11	96434042	LOC106563746	0 061202577	AX-87031274	96414963	96434423	0
ssa11	96698005	LOC106563742	0 056787193	AX-87357775	96685694	96691370	-6636
ssa11	96850111	LOC106563735	0 060438542	AX-86977410	96849770	96857336	0
ssa11	97004243	LOC106563731	0 066407337	AX-87797361	97000506	97005554	0
ssa11	97005647	LOC106563730	0 066407337	AX-87623752	97005550	97008786	0
ssa11	97071430	LOC106563726	0 065884784	AX-87055505	97046687	97183093	0
ssa11	97102483	LOC106563726	0 072920725	AX-87616310	97046687	97183093	0
ssa11	97102514	LOC106563726	0 070919495	AX-87181131	97046687	97183093	0
ssa11	97169501	LOC106563726	0 068663854	AX-87548119	97046687	97183093	0

ssa11	97170403	LOC106563726	0 07205066	AX-87290389	97046687	97183093	0
ssa11	97171343	LOC106563726	0 07514977	AX-87691754	97046687	97183093	0
ssa11	97358864	LOC106563753	0 058150435	AX-87622272	97378976	97380998	20112
ssa11	97417229	cnot6	0 061671486	AX-87689794	97403813	97417613	0
ssa11	97654695	LOC106563761	0 057165081	AX-87601652	97668807	97775634	14112
ssa11	97658368	LOC106563761	0 057165081	AX-87454917	97668807	97775634	10439
ssa11	97665296	LOC106563761	0 061530417	AX-87733013	97668807	97775634	3511
ssa11	98240538	LOC106563813	0 057215079	AX-87861085	98239412	98245731	0
ssa11	99356209	LOC106563798	0 070270815	AX-87202420	99350710	99358275	0
ssa11	100162598	LOC106563656	0 058240125	AX-87464483	100173889	100176881	11291
ssa11	100176023	LOC106563656	0 058240125	AX-87252717	100173889	100176881	0
ssa11	100186472	LOC106563656	0 058815295	AX-87044348	100173889	100176881	-9592
ssa11	100303141	LOC106563645	0 068928151	AX-87559020	100300446	100301871	-1271
ssa12	14105076	LOC106564260	0 0688367	AX-87469333	14084897	14110506	0
ssa12	14782804	LOC123725421	0 066386174	AX-87306604	14748548	14774451	-8354
ssa12	26295202	LOC106564696	0 05676872	AX-87500731	26275873	26308665	0
ssa12	26308835	LOC106564696	0 05676872	AX-87250865	26275873	26308665	-171
ssa12	59970111	LOC106565395	0 058728189	AX-87592777	59966706	59973429	0
ssa12	61556778	LOC106565447	0 059211603	AX-87688161	61519331	61551523	-5256
ssa12	68822106	LOC106565623	0 064545354	AX-87258406	68813588	68830291	0
ssa12	73372975	LOC106565865	0 065030202	AX-87121476	73288466	73393292	0
ssa12	81483486	LOC106565730	0 07468037	AX-87642676	81513463	81600753	29977
ssa12	81497747	LOC106565730	0 057852095	AX-87006472	81513463	81600753	15716
ssa12	84237165	hemk1	0 061526711	AX-87155396	84234536	84238035	0
ssa12	88162739	igfn1.1	0 071549823	AX-87383763	88155742	88189473	0
ssa13	7582717	lama5	0 059422949	AX-87635366	7549275	7789344	0
ssa13	7683458	lama5	0 056886336	AX-87397483	7549275	7789344	0
ssa13	7732466	lama5	0 060570621	AX-87102855	7549275	7789344	0
ssa13	16732452	cacna2d3a	0 059290701	AX-87214242	16456358	16784920	0
ssa13	22516059	LOC106566616	0 071627676	AX-87245003	22473228	22569452	0
ssa13	22569084	LOC106566616	0 063893664	AX-87645561	22473228	22569452	0
ssa13	22679663	LOC106566610	0 063531587	AX-87664832	22668818	22793607	0
ssa13	32470023	LOC106566898	0 059737932	AX-87036862	32472679	32492636	2656
ssa13	32771358	LOC106566907	0 065523697	AX-86928085	32734757	32888803	0
ssa13	32937606	si:ch73-217n20.1	0 060635543	AX-87512212	32936274	32944328	0
ssa13	36270469	LOC106567213	0 066959726	AX-87009611	36270973	36275717	504
ssa13	37305118	slc48a1a	0 057261151	AX-87114560	37301032	37305465	0
ssa13	37307217	LOC106567160	0 06611849	AX-87424177	37305689	37313413	0
ssa13	38355999	LOC106567150	0 059526123	AX-87078107	38210947	38403929	0
ssa13	38362185	LOC106567150	0 061482817	AX-87465159	38210947	38403929	0
ssa13	41723705	LOC106567057	0 063362462	AX-87024176	41681819	41821211	0
ssa13	48933928	plch2a	0 059156492	AX-87601961	48842615	49026542	0

ssa13	48944579	plch2a	0	063136468	AX-87082880	48842615	49026542	0
ssa13	48959938	plch2a	0	060323189	AX-87345098	48842615	49026542	0
ssa13	49054825	LOC106567362	0	064531214	AX-87583192	49045588	49133031	0
ssa13	49121674	LOC106567362	0	065704786	AX-87784564	49045588	49133031	0
ssa13	49280416	LOC106567368	0	067340024	AX-87473305	49257942	49259136	-21281
ssa13	49288942	LOC106567368	0	068445194	AX-87326662	49257942	49259136	-29807
ssa13	49347413	LOC106567369	0	061365641	AX-87349660	49338493	49361318	0
ssa13	49369741	megf6	0	063952112	AX-87544239	49361355	49456500	0
ssa13	49806877	LOC106567371	0	057284825	AX-87836166	49801956	49803866	-3012
ssa13	50519822	LOC106567379	0	074159609	AX-87220199	50468830	50526062	0
ssa13	51002974	LOC106567384	0	061538958	AX-87720060	50938965	50982150	-20825
ssa13	52135234	LOC106567400	0	058423279	AX-87424962	52085228	52269317	0
ssa13	54223071	LOC106567420	0	066108178	AX-87418572	54206641	54275518	0
ssa13	54472713	LOC106567426	0	068831931	AX-87301931	54461941	54476930	0
ssa13	54476629	LOC106567426	0	068831931	AX-87775610	54461941	54476930	0
ssa13	55287479	LOC106567439	0	058654117	AX-87001699	55260520	55289296	0
ssa13	55626934	LOC106567442	0	06397245	AX-87818949	55592857	55625612	-1323
ssa13	55636409	LOC106567484	0	06397245	AX-87573644	55636372	55648484	0
ssa13	55643211	LOC106567484	0	06397245	AX-87426248	55636372	55648484	0
ssa13	55647207	LOC106567484	0	06397245	AX-87751706	55636372	55648484	0
ssa13	55649163	LOC106567443	0	06397245	AX-87105924	55648840	55675825	0
ssa13	55649342	LOC106567443	0	06397245	AX-87710707	55648840	55675825	0
ssa13	55661837	LOC106567443	0	06397245	AX-87636265	55648840	55675825	0
ssa13	55665062	LOC106567443	0	06397245	AX-87233806	55648840	55675825	0
ssa13	55670587	LOC106567443	0	062363334	AX-87598483	55648840	55675825	0
ssa13	55670714	LOC106567443	0	06397245	AX-87723004	55648840	55675825	0
ssa13	55798893	LOC106567447	0	058912163	AX-87852627	55792441	55820204	0
ssa13	61293861	LOC106567543	0	080590581	AX-87540751	61264338	61307186	0
ssa13	61365509	LOC106567544	0	0787726	AX-87495465	61336892	61366074	0
ssa13	61442305	LOC106567545	0	057411295	AX-87157764	61379836	61507594	0
ssa13	61468559	LOC106567545	0	074431347	AX-87071940	61379836	61507594	0
ssa13	61508524	LOC106567545	0	060024173	AX-87130589	61379836	61507594	-931
ssa13	65753065	LOC106567580	0	062359102	AX-87257406	65717674	65898836	0
ssa13	65816576	LOC106567580	0	059650394	AX-87280245	65717674	65898836	0
ssa13	67498449	vps11	0	064837925	AX-87045741	67496814	67522070	0
ssa13	67501097	vps11	0	064837925	AX-87166151	67496814	67522070	0
ssa13	67501185	vps11	0	064837925	AX-87231898	67496814	67522070	0
ssa13	67507678	vps11	0	063869504	AX-86977843	67496814	67522070	0
ssa13	67509522	vps11	0	064837925	AX-87654218	67496814	67522070	0
ssa13	67510311	vps11	0	064837925	AX-87514819	67496814	67522070	0
ssa13	67522082	LOC106567637	0	064837925	AX-87017749	67521266	67544325	0
ssa13	67522214	LOC106567637	0	064837925	AX-87605099	67521266	67544325	0

ssa13	67561611	zpr1	0 062311952	AX-87704136	67561562	67580439	0
ssa13	67758634	bsx	0 065388281	AX-87686541	67755120	67757184	-1451
ssa13	67760119	lim2.1	0 067493882	AX-86954288	67760223	67770516	104
ssa13	68840650	LOC106567659	0 061132338	AX-87767390	68534727	69009172	0
ssa13	68861694	LOC106567659	0 058302738	AX-87519035	68534727	69009172	0
ssa13	68881943	LOC106567659	0 059660592	AX-87263059	68534727	69009172	0
ssa13	68936805	LOC106567659	0 059660592	AX-87668244	68534727	69009172	0
ssa13	69335985	LOC106567647	0 072023466	AX-87526129	69251251	69355725	0
ssa13	69362264	tmem218	0 074282748	AX-87193106	69357017	69364386	0
ssa13	69482947	LOC106567652	0 064302614	AX-87065217	69414035	69714914	0
ssa13	69569394	LOC106567652	0 057020653	AX-87719118	69414035	69714914	0
ssa13	70454335	arhgap32b	0 060110272	AX-87272818	70308830	70551602	0
ssa13	70455467	arhgap32b	0 066130352	AX-87112376	70308830	70551602	0
ssa13	90374046	LOC106568105	0 062375848	AX-87506186	90365844	90372723	-1324
ssa13	90464228	bmp1	0 062375848	AX-87658113	90420852	90479257	0
ssa13	90726678	LOC100136462	0 056614434	AX-86961771	90712254	90722950	-3729
ssa13	91038470	LOC106568226	0 073035615	AX-86989814	91035567	91084754	0
ssa13	91080432	LOC106568226	0 086390774	AX-87710069	91035567	91084754	0
ssa13	91300558	LOC106568207	0 058449821	AX-87845610	91294644	91302473	0
ssa13	91310606	LOC106568220	0 06463452	AX-87626138	91318416	91320792	7810
ssa13	91312348	LOC106568220	0 06463452	AX-87760644	91318416	91320792	6068
ssa13	91360441	foxred1	0 062171929	AX-87747911	91360057	91374183	0
ssa13	91803011	LOC106568196	0 077078894	AX-87501882	91771514	91788577	-14435
ssa13	92537209	LOC106568185	0 057026272	AX-87246177	92508418	92621005	0
ssa13	92614033	LOC106568185	0 061073174	AX-87771976	92508418	92621005	0
ssa13	107672307	LOC106568532	0 065142029	AX-87823160	107619707	107672136	-172
ssa13	108353329	LOC106568538	0 059538051	AX-87036336	108352988	108420408	0
ssa14	492225	klhl18	0 059352929	AX-87715147	423348	521590	0
ssa14	22757659	LOC106569075	0 064029231	AX-87599525	22683290	22810692	0
ssa14	43003922	kirrel1b	0 058107872	AX-87275144	43003902	43065872	0
ssa14	74776682	LOC106570301	0 066656006	AX-87864259	74775348	74778214	0
ssa14	74776687	LOC106570301	0 066656006	AX-86908437	74775348	74778214	0
ssa14	74808367	LOC106570299	0 059358872	AX-87058040	74792884	74824715	0
ssa14	74834232	LOC106570298	0 07661012	AX-87737615	74833644	74846510	0
ssa14	74834590	LOC106570298	0 076466114	AX-86919138	74833644	74846510	0
ssa14	74834805	LOC106570298	0 078103668	AX-87018845	74833644	74846510	0
ssa14	75013737	LOC106570290	0 058975788	AX-87569113	74999002	75018058	0
ssa14	76045123	LOC106570311	0 059208925	AX-87576291	75973641	76023603	-21521
ssa15	2484370	LOC106570834	0 061519691	AX-87841888	2479986	2493585	0
ssa15	24883010	LOC106571107	0 057972834	AX-87027947	24815091	24906953	0
ssa15	25368557	LOC123727248	0 068239874	AX-87615060	25370223	25382541	1666
ssa15	25368934	LOC123727248	0 068239874	AX-87075321	25370223	25382541	1289

ssa15	25371493	LOC123727248	0 066623348	AX-87510071	25370223	25382541	0
ssa15	25400042	LOC106571120	0 072953047	AX-87274646	25400284	25423863	242
ssa15	25417838	LOC106571120	0 064789648	AX-87296065	25400284	25423863	0
ssa15	25462232	LOC106571124	0 06056346	AX-86922948	25461426	25568434	0
ssa15	25465420	LOC106571124	0 064734201	AX-87113043	25461426	25568434	0
ssa15	34597694	LOC106571327	0 057794384	AX-86934367	34596959	34609019	0
ssa15	34599314	LOC106571327	0 057794384	AX-86915454	34596959	34609019	0
ssa15	34608753	LOC106571327	0 057794384	AX-86913385	34596959	34609019	0
ssa15	34636817	LOC106571323	0 057794384	AX-87812933	34618583	34645965	0
ssa15	34646730	nt5d1	0 057794384	AX-87667107	34646137	34725712	0
ssa15	34861367	LOC106571321	0 064343501	AX-87741700	34862479	34962148	1112
ssa15	34996299	marcs	0 059431892	AX-87117050	34993961	34997382	0
ssa15	34996824	marcs	0 067248277	AX-87422119	34993961	34997382	0
ssa15	34997854	marcs	0 064343501	AX-87784159	34993961	34997382	-473
ssa15	34998133	LOC106571347	0 059431892	AX-87666017	34998342	34999833	209
ssa15	35027724	LOC106571347	0 059976442	AX-87765788	34998342	34999833	-27892
ssa15	35589835	LOC106571312	0 058255377	AX-86997571	35583691	35602247	0
ssa15	35603158	cdk19	0 060672376	AX-87366339	35603450	35658016	292
ssa15	35606629	cdk19	0 059471332	AX-87231604	35603450	35658016	0
ssa15	44663925	LOC106571714	0 056978931	AX-87306518	44584296	44835223	0
ssa15	44668219	LOC106571714	0 059425086	AX-87761426	44584296	44835223	0
ssa15	44670219	LOC106571714	0 056978931	AX-87393708	44584296	44835223	0
ssa15	65070920	LOC106571924	0 075826183	AX-87719451	65017053	65414757	0
ssa15	79510067	kif1b	0 071191988	AX-87688144	79408727	79509698	-370
ssa15	79510075	kif1b	0 071191988	AX-87318452	79408727	79509698	-378
ssa15	79550665	ube4b	0 074565916	AX-87505490	79510790	79562363	0
ssa15	86111948	LOC106572392	0 065936566	AX-87466460	85539831	86140509	0
ssa15	91485663	LOC106572632	0 066084539	AX-86962677	91487408	91566076	1745
ssa15	91554323	LOC106572632	0 057881789	AX-87047065	91487408	91566076	0
ssa15	91554442	LOC106572632	0 057881789	AX-87401560	91487408	91566076	0
ssa15	91554634	LOC106572632	0 05941462	AX-87490164	91487408	91566076	0
ssa15	94385142	LOC106572671	0 057864855	AX-87833209	94376426	94384015	-1128
ssa15	94395451	LOC106572669	0 061184303	AX-87403650	94388480	94452733	0
ssa16	4219921	LOC106573061	0 061420771	AX-87045319	4174162	4245099	0
ssa16	4984787	LOC106573083	0 059632922	AX-86970330	4993159	5115349	8372
ssa16	7419912	LOC106573156	0 067090443	AX-87690213	7409719	7415456	-4457
ssa16	13162762	LOC106573206	0 059239516	AX-87096634	13154862	13182909	0
ssa16	13177543	LOC106573206	0 056849399	AX-86985693	13154862	13182909	0
ssa16	20415252	LOC106573348	0 056591679	AX-87774721	20216026	20396774	-18479
ssa16	40351705	LOC106573547	0 059057648	AX-87413058	40349596	40379215	0
ssa16	46356147	LOC106574075	0 062273479	AX-86917174	46246260	46530210	0
ssa16	51999672	LOC123727937	0 059614483	AX-87689125	51961871	51961927	-37746

ssa16	52086305	LOC106574168	0	059614483	AX-87838616	52073205	52097302	0
ssa16	52106349	LOC106574165	0	059949587	AX-87821617	52098122	52195287	0
ssa16	52150151	LOC106574165	0	059656088	AX-87743114	52098122	52195287	0
ssa16	52160587	LOC106574165	0	057018	AX-87486934	52098122	52195287	0
ssa16	52244686	LOC106574164	0	058454265	AX-87346090	52206691	52237153	-7534
ssa16	63933424	LOC106574307	0	058250186	AX-86945487	63930917	63940612	0
ssa16	63935858	LOC106574307	0	058250186	AX-87076595	63930917	63940612	0
ssa16	64626503	LOC106574300	0	058173177	AX-86946254	64623696	64699452	0
ssa16	65032265	LOC106574298	0	068865825	AX-87279775	65036226	65166732	3961
ssa16	65178527	si:ch211-246m6.5	0	068865825	AX-86926389	65178453	65224073	0
ssa16	65266504	aox5	0	06752318	AX-87501903	65244819	65270907	0
ssa16	65565944	ahr2d	0	066677306	AX-87712440	65535234	65611355	0
ssa16	65762254	LOC106574287	0	059292422	AX-87113086	65762331	65769581	77
ssa16	65822145	LOC106574285	0	065198361	AX-87761181	65813746	65833193	0
ssa16	65826406	LOC106574285	0	057835578	AX-87742103	65813746	65833193	0
ssa16	65831535	LOC106574285	0	065198361	AX-86925954	65813746	65833193	0
ssa16	65871346	ftcd	0	071677188	AX-87826549	65863928	65870868	-479
ssa16	65876876	LOC106574281	0	071278868	AX-87091554	65877092	65901559	216
ssa16	65885290	LOC106574281	0	070813601	AX-87614576	65877092	65901559	0
ssa16	65891393	LOC106574281	0	071278868	AX-87240514	65877092	65901559	0
ssa16	65896103	LOC106574281	0	071278868	AX-86950153	65877092	65901559	0
ssa16	65896337	LOC106574281	0	071278868	AX-87462493	65877092	65901559	0
ssa16	65900216	LOC106574281	0	068092809	AX-87336370	65877092	65901559	0
ssa16	65900855	LOC106574281	0	071278868	AX-87441610	65877092	65901559	0
ssa16	65928099	LOC106574280	0	068107862	AX-87156461	65901784	65926326	-1774
ssa16	65960153	LOC106574279	0	069988201	AX-87726717	65932315	65993032	0
ssa16	87873124	LOC106574953	0	056608021	AX-87067255	87802118	87955350	0
ssa16	88373713	LOC106575349	0	064323547	AX-87490539	88362100	88390415	0
ssa17	8549848	LOC106575119	0	057348868	AX-87064534	8552239	8606546	2391
ssa17	8636052	LOC106575116	0	065179492	AX-87125368	8621123	8646848	0
ssa17	9807444	LOC106575089	0	058873861	AX-87442283	9720642	9861927	0
ssa17	9982396	plcc	0	058454467	AX-87813081	9981673	10013998	0
ssa17	10215232	LOC106575079	0	072768067	AX-87304749	10201242	10310678	0
ssa17	10778841	LOC106575234	0	081358382	AX-87363755	10780722	10823513	1881
ssa17	10780332	LOC106575234	0	081358382	AX-87491994	10780722	10823513	390
ssa17	10781685	LOC106575234	0	080423086	AX-87843981	10780722	10823513	0
ssa17	10785736	LOC106575234	0	060817196	AX-87448449	10780722	10823513	0
ssa17	10791365	LOC106575234	0	065959586	AX-87329762	10780722	10823513	0
ssa17	18808545	LOC106575374	0	056849741	AX-87733536	18787667	18809144	0
ssa17	18852934	LOC106575378	0	06203281	AX-87073346	18845860	18849456	-3479
ssa17	18894385	LOC106575379	0	064915685	AX-87000404	18893695	18926486	0
ssa17	60014968	LOC106576215	0	065626113	AX-86945011	59842442	60105157	0

ssa17	60118661	LOC106576214	0 064456747	AX-87121640	60121701	60212849	3040
ssa17	60119242	LOC106576214	0 072234848	AX-87356062	60121701	60212849	2459
ssa17	60121525	LOC106576214	0 070994807	AX-87267892	60121701	60212849	176
ssa17	60144564	LOC106576214	0 071412863	AX-87449724	60121701	60212849	0
ssa17	60170677	LOC106576214	0 065348317	AX-87348807	60121701	60212849	0
ssa17	60185480	LOC106576214	0 071267657	AX-87245223	60121701	60212849	0
ssa17	62663885	LOC106576312	0 061121599	AX-87575469	62635312	62703267	0
ssa17	62665299	LOC106576312	0 061640822	AX-87721847	62635312	62703267	0
ssa17	62671216	LOC106576312	0 059553467	AX-87166092	62635312	62703267	0
ssa17	62673229	LOC106576312	0 061121599	AX-87684149	62635312	62703267	0
ssa17	68307839	LOC106609571	0 057346985	AX-87659655	68025794	68367584	0
ssa17	68609173	LOC106576572	0 058354199	AX-87027121	68472631	68661915	0
ssa17	74492064	LOC106576583	0 058556392	AX-86911418	74486667	74506108	0
ssa17	74525808	LOC106576584	0 05939368	AX-87821631	74511400	74529562	0
ssa17	74534828	LOC106576584	0 059957139	AX-87456008	74511400	74529562	-5267
ssa18	10953815	LOC106576768	0 061774272	AX-87093509	10944396	10962834	0
ssa18	10964836	LOC106576768	0 057304684	AX-86982602	10944396	10962834	-2003
ssa18	10970474	LOC106576768	0 058653232	AX-87227901	10944396	10962834	-7641
ssa18	11384984	tisd	0 066910065	AX-87678468	11381520	11384795	-190
ssa18	11385870	tisd	0 07020708	AX-87681049	11381520	11384795	-1076
ssa18	16094467	LOC106576956	0 060995621	AX-87739109	16083564	16094811	0
ssa18	16096096	LOC106576957	0 056996509	AX-87740766	16096162	16112276	66
ssa18	16106302	LOC106576957	0 060995621	AX-87705974	16096162	16112276	0
ssa18	16110439	LOC106576957	0 060995621	AX-87310367	16096162	16112276	0
ssa18	16128140	LOC106576958	0 06319873	AX-87401670	16126092	16132255	0
ssa18	16128567	LOC106576958	0 076391866	AX-87554877	16126092	16132255	0
ssa18	18420654	LOC106576990	0 059260447	AX-87228053	18371071	18425130	0
ssa18	18624432	LOC106576995	0 059800231	AX-87625360	18623461	18650453	0
ssa18	18717207	pcsk2	0 070645169	AX-87716919	18733200	18817352	15993
ssa18	18724813	pcsk2	0 076047167	AX-87262264	18733200	18817352	8387
ssa18	18730682	pcsk2	0 057339398	AX-87061605	18733200	18817352	2518
ssa18	23674126	LOC106577144	0 068987478	AX-87742236	23634548	23800213	0
ssa18	23723911	LOC106577144	0 067416929	AX-87650155	23634548	23800213	0
ssa18	25420281	LOC106577124	0 063340133	AX-87399152	25259347	25614581	0
ssa18	35021071	LOC106577388	0 064660347	AX-86948044	35013119	35021717	0
ssa18	56635058	LOC106577538	0 058820063	AX-87646331	56614869	56633382	-1677
ssa18	57187884	LOC106577539	0 057359012	AX-87206260	56842211	57778326	0
ssa18	59835489	LOC106577599	0 058697513	AX-87238961	59833698	59890206	0
ssa18	66817165	LOC106577804	0 058180228	AX-87463742	66830709	66844005	13544
ssa18	81420855	LOC106578155	0 068183786	AX-87371535	81452662	81466739	31807
ssa19	1363856	LOC123729044	0 059011677	AX-87640870	1309139	1353503	-10354
ssa19	1380055	LOC106578273	0 072884916	AX-87266058	1378334	1411053	0

ssa19	16559785	pfkpa	0 061637878	AX-87007108	16543338	16580029	0
ssa19	16561088	pfkpa	0 061637878	AX-86919337	16543338	16580029	0
ssa19	16577339	pfkpa	0 061012594	AX-86929760	16543338	16580029	0
ssa19	25249279	fars2	0 061425675	AX-87218099	25111919	25262506	0
ssa19	46748786	trnat-ugu	0 060768695	AX-87553948	46792165	46792239	43379
ssa19	46748901	trnat-ugu	0 063238019	AX-86927412	46792165	46792239	43264
ssa19	46771154	trnat-ugu	0 061825479	AX-86965808	46792165	46792239	21011
ssa19	46786936	trnat-ugu	0 060768695	AX-87103047	46792165	46792239	5229
ssa19	80700395	LOC106579687	0 057714426	AX-87050527	80588157	80723148	0
ssa19	85223474	trnav-uac	0 077624768	AX-87147472	85242470	85242545	18996
ssa20	9738342	kiaa0825	0 058947906	AX-87101180	9615104	9770385	0
ssa20	9788470	slf1	0 061977765	AX-87086540	9770594	9807603	0
ssa20	9806884	slf1	0 067530779	AX-87200259	9770594	9807603	0
ssa20	9827137	LOC106580145	0 065727045	AX-87555864	9808600	10036686	0
ssa20	9828986	LOC106580145	0 069855978	AX-87186881	9808600	10036686	0
ssa20	9829670	LOC106580145	0 069855978	AX-87412632	9808600	10036686	0
ssa20	9851761	LOC106580145	0 06956059	AX-86990367	9808600	10036686	0
ssa20	9857886	LOC106580145	0 070437219	AX-87422866	9808600	10036686	0
ssa20	21942864	LOC106579912	0 059017988	AX-86993420	21945189	21950397	2325
ssa20	45755157	LOC123729170	0 061014272	AX-87414387	45752090	45931775	0
ssa20	47312917	cbl	0 056904658	AX-87447972	47325598	47380899	12681
ssa20	47960914	LOC123729175	0 06580859	AX-86940162	47958021	47980048	0
ssa20	47961176	LOC123729175	0 061183315	AX-87720806	47958021	47980048	0
ssa20	48079259	LOC123729233	0 062345454	AX-87538355	48079994	48115904	735
ssa20	48079265	LOC123729233	0 058827953	AX-87054716	48079994	48115904	729
ssa20	48218624	LOC123729176	0 059237656	AX-87166872	48123449	48223094	0
ssa20	48409017	LOC123729177	0 060195297	AX-87790547	48402070	48409073	0
ssa20	48414965	LOC123729244	0 059095649	AX-87058778	48412250	48461632	0
ssa20	48427319	LOC123729244	0 060195297	AX-87323690	48412250	48461632	0
ssa20	48437919	LOC123729244	0 060195297	AX-87037893	48412250	48461632	0
ssa20	48480116	LOC123729244	0 067439383	AX-87286014	48412250	48461632	-18485
ssa20	48543641	LOC123729245	0 065072559	AX-87869455	48550279	48551584	6638
ssa20	48543934	LOC123729245	0 06247414	AX-87776113	48550279	48551584	6345
ssa20	48545192	LOC123729245	0 065072559	AX-87181522	48550279	48551584	5087
ssa20	48550817	LOC123729245	0 066483976	AX-87622413	48550279	48551584	0
ssa20	48709459	calm3a	0 061498647	AX-87306778	48695048	48712077	0
ssa20	48711696	calm3a	0 061498647	AX-87735387	48695048	48712077	0
ssa20	48734416	LOC123729248	0 063924771	AX-87724110	48724033	48775291	0
ssa20	48783461	LOC123729248	0 061498647	AX-87168273	48724033	48775291	-8171
ssa20	52274170	LOC106580770	0 057571988	AX-87620506	52194242	52270448	-3723
ssa20	52284673	spit2	0 056763448	AX-87733902	52288854	52303802	4181
ssa20	68649252	LOC106581099	0 056763163	AX-87561848	68506721	68818462	0

ssa20	68649939	LOC106581099	0 056763163	AX-87093818	68506721	68818462	0
ssa20	68661752	LOC106581099	0 056763163	AX-87262709	68506721	68818462	0
ssa20	68667661	LOC106581099	0 056763163	AX-87085031	68506721	68818462	0
ssa20	68668429	LOC106581099	0 056763163	AX-87192348	68506721	68818462	0
ssa20	68668957	LOC106581099	0 056763163	AX-87573355	68506721	68818462	0
ssa20	69156998	LOC100136491	0 057835743	AX-87354985	69145785	69158502	0
ssa20	69161107	LOC100136491	0 073717568	AX-86999447	69145785	69158502	-2606
ssa20	69169980	LOC100136491	0 073053088	AX-87545352	69145785	69158502	-11479
ssa20	69170262	LOC100136491	0 073053088	AX-87550069	69145785	69158502	-11761
ssa20	69175366	LOC100136491	0 073053088	AX-87113871	69145785	69158502	-16865
ssa20	69178434	LOC100136491	0 07328167	AX-87385350	69145785	69158502	-19933
ssa20	69183206	LOC100136491	0 073053088	AX-87566068	69145785	69158502	-24705
ssa20	69198558	LOC106581098	0 057335291	AX-86960482	69214638	69262327	16080
ssa20	69290863	LOC106581097	0 058209655	AX-87517677	69287951	69318905	0
ssa20	69413262	kcnj5	0 077142207	AX-86948377	69362105	69399894	-13369
ssa20	69555081	LOC106581092	0 071225625	AX-87809356	69556762	69566323	1681
ssa20	69659548	b3gat1a	0 071225625	AX-87047724	69631302	69740874	0
ssa20	69702531	b3gat1a	0 071225625	AX-87272990	69631302	69740874	0
ssa20	69718783	b3gat1a	0 072153233	AX-87194579	69631302	69740874	0
ssa20	69725017	b3gat1a	0 057533659	AX-87085557	69631302	69740874	0
ssa20	69773395	b3gat1a	0 057533659	AX-87773996	69631302	69740874	-32522
ssa20	69973164	siae	0 071225625	AX-87163551	69963978	69988797	0
ssa20	70070513	LOC106581222	0 071225625	AX-87162137	70071243	70081325	730
ssa20	70258869	LOC106581220	0 061677198	AX-87241253	70306322	70316153	47453
ssa20	70259720	LOC106581220	0 057623202	AX-87749825	70306322	70316153	46602
ssa20	70286911	LOC106581220	0 072672747	AX-87680350	70306322	70316153	19411
ssa20	70720380	LOC106581238	0 059892447	AX-87205711	70700810	70719725	-656
ssa20	70737007	LOC106581239	0 06029451	AX-87648224	70721941	70931715	0
ssa20	70755479	LOC106581239	0 074009159	AX-87848122	70721941	70931715	0
ssa20	70935950	LOC106581239	0 06766537	AX-87614753	70721941	70931715	-4236
ssa20	70937096	LOC106581239	0 072894779	AX-87522298	70721941	70931715	-5382
ssa20	70938922	LOC106581239	0 06766537	AX-87475601	70721941	70931715	-7208
ssa20	70972732	LOC106581247	0 060639562	AX-86941210	70969996	71152770	0
ssa20	77492684	slc36a4	0 058328197	AX-87660901	77294256	77556468	0
ssa20	77546786	slc36a4	0 05866336	AX-87268664	77294256	77556468	0
ssa21	1301137	tbc1d4	0 058719215	AX-87812432	1200802	1361383	0
ssa21	10228709	LOC106581700	0 068287567	AX-87273313	9919164	10237320	0
ssa21	10454206	LOC123729587	0 065673803	AX-87684891	10248998	10456423	0
ssa21	10487743	LOC106581718	0 067132756	AX-87385938	10492660	10493964	4917
ssa21	10504618	LOC106581718	0 062215401	AX-87660886	10492660	10493964	-10655
ssa21	10608045	LOC106581870	0 06402382	AX-87709041	10653191	10667861	45146
ssa21	10608447	LOC106581870	0 067245763	AX-87597843	10653191	10667861	44744

ssa21	10654575	LOC106581870	0 057708731	AX-87546677	10653191	10667861	0
ssa21	10773281	hdac4	0 057654177	AX-87531370	10772364	11018212	0
ssa21	14320582	LOC106581814	0 059472323	AX-87043398	14317309	14325277	0
ssa21	14813593	LOC106581809	0 059041262	AX-87452622	14721985	14814931	0
ssa21	14856276	LOC106581710	0 066321255	AX-87404780	14839148	14911507	0
ssa21	15612097	csrnp3	0 071617964	AX-86979781	15604341	15677006	0
ssa21	16510812	mettl8	0 060532579	AX-87456621	16492023	16508737	-2076
ssa21	22295129	kdm6a	0 067583121	AX-87045698	22254818	22359126	0
ssa21	22295550	kdm6a	0 062497694	AX-87201723	22254818	22359126	0
ssa21	22800026	ddx3xa	0 060782769	AX-87085224	22791685	22816082	0
ssa21	36690650	LOC106582286	0 062711511	AX-87462272	36562616	36744637	0
ssa21	36708103	LOC106582286	0 062125867	AX-87311838	36562616	36744637	0
ssa21	36734753	LOC106582286	0 061308223	AX-87615571	36562616	36744637	0
ssa21	36759105	LOC100194642	0 061308223	AX-87307222	36744173	36754768	-4338
ssa21	36759430	LOC100194642	0 061308223	AX-87225563	36744173	36754768	-4663
ssa21	36763235	LOC100194642	0 061308223	AX-87615994	36744173	36754768	-8468
ssa21	40724276	slc49a4	0 059799814	AX-87691553	40680673	40751648	0
ssa21	42087212	LOC106582334	0 05695864	AX-87576115	41884111	42065630	-21583
ssa21	45421954	LOC106582401	0 057283343	AX-86984252	45405808	45447622	0
ssa21	51042376	atp5g2	0 066767858	AX-87849852	51041210	51043589	0
ssa21	51047666	LOC106582491	0 079512764	AX-87704495	51044546	51079803	0
ssa21	51049046	LOC106582491	0 096862176	AX-86922730	51044546	51079803	0
ssa21	53680991	tmem198a	0 063620003	AX-87552101	53675738	53766525	0
ssa22	7746529	LOC106582705	0 062611401	AX-87574104	7743331	7776676	0
ssa22	7785924	LOC106582705	0 061944699	AX-87168820	7743331	7776676	-9249
ssa22	12660305	fam83e	0 059507411	AX-87016849	12657565	12665303	0
ssa22	12660545	fam83e	0 059507411	AX-87481622	12657565	12665303	0
ssa22	12661513	fam83e	0 059507411	AX-87691934	12657565	12665303	0
ssa22	12661840	fam83e	0 059507411	AX-87696094	12657565	12665303	0
ssa22	29447612	LOC106583215	0 063136681	AX-87190073	29399666	29452194	0
ssa22	29477144	LOC106583214	0 060446114	AX-87198726	29462572	29487464	0
ssa22	29485174	LOC106583214	0 060446114	AX-86994231	29462572	29487464	0
ssa22	29575233	eefsec	0 058450409	AX-87200481	29542125	29562829	-12405
ssa22	49450821	LOC106583657	0 059936288	AX-87076603	49444316	49677213	0
ssa22	50171153	eya2	0 059210028	AX-87834162	50098533	50152053	-19101
ssa22	51833621	LOC106583670	0 066297711	AX-87180977	51807154	51850142	0
ssa22	52037175	LOC106583688	0 062719362	AX-87751954	52041064	52093602	3889
ssa22	57892753	gmppb	0 066755762	AX-87590613	57878385	57893148	0
ssa23	714108	LOC106583871	0 058481275	AX-87539788	720369	747342	6261
ssa23	873411	LOC106583881	0 06513879	AX-87219041	872612	878394	0
ssa23	902804	LOC106583882	0 066714072	AX-87168852	884247	911520	0
ssa23	1061687	LOC106583889	0 063729346	AX-87402415	1108328	1111603	46641

ssa23	1099666	LOC106583889	0 069714711	AX-87707887	1108328	1111603	8662
ssa23	1412114	LOC106583883	0 071255464	AX-87649060	1364867	1443837	0
ssa23	1475864	LOC106583886	0 067350552	AX-87728904	1463766	1476835	0
ssa23	1582073	LOC106583929	0 065759472	AX-87035916	1550733	1627658	0
ssa23	1647682	hn1l	0 071287506	AX-87165759	1626320	1662297	0
ssa23	1647802	hn1l	0 056960312	AX-87279488	1626320	1662297	0
ssa23	1687342	LOC106583928	0 064953484	AX-87405554	1662488	1716962	0
ssa23	1695933	LOC106583928	0 074898165	AX-87477347	1662488	1716962	0
ssa23	1716774	LOC106583928	0 05947212	AX-87392189	1662488	1716962	0
ssa23	1719042	LOC106583928	0 071287506	AX-87540092	1662488	1716962	-2081
ssa23	1721434	LOC106583928	0 071287506	AX-87128349	1662488	1716962	-4473
ssa23	1753432	LOC106583926	0 065630618	AX-87363946	1751580	1754108	0
ssa23	1756795	LOC106583926	0 05947212	AX-87661903	1751580	1754108	-2688
ssa23	1823697	LOC106583924	0 070027524	AX-87282336	1826370	1862879	2673
ssa23	1824669	LOC106583924	0 074494213	AX-87449636	1826370	1862879	1701
ssa23	1828586	LOC106583924	0 065759472	AX-87396853	1826370	1862879	0
ssa23	1829153	LOC106583924	0 071252438	AX-87843876	1826370	1862879	0
ssa23	1830440	LOC106583924	0 068020052	AX-87149676	1826370	1862879	0
ssa23	1830509	LOC106583924	0 068020052	AX-87167834	1826370	1862879	0
ssa23	1834373	LOC106583924	0 073451814	AX-87315947	1826370	1862879	0
ssa23	1834454	LOC106583924	0 073451814	AX-87667920	1826370	1862879	0
ssa23	1834865	LOC106583924	0 073451814	AX-87690031	1826370	1862879	0
ssa23	1896305	LOC106583924	0 068963007	AX-87172839	1826370	1862879	-33427
ssa23	1913685	LOC106583923	0 068020052	AX-87141856	1958254	1976518	44569
ssa23	1960859	LOC106583923	0 068020052	AX-87141910	1958254	1976518	0
ssa23	1967630	LOC106583923	0 064670059	AX-87573377	1958254	1976518	0
ssa23	2018161	LOC106583922	0 073575899	AX-86946958	2012005	2019338	0
ssa23	2019308	LOC106583922	0 064390625	AX-87630158	2012005	2019338	0
ssa23	2071908	LOC106583920	0 064390625	AX-87257484	2033411	2116005	0
ssa23	2115484	LOC106583920	0 070027524	AX-87021802	2033411	2116005	0
ssa23	2144014	LOC106583918	0 06562275	AX-87250774	2127474	2145593	0
ssa23	2165481	LOC106583898	0 065545379	AX-87803273	2158809	2165316	-166
ssa23	2217217	LOC106583897	0 062749411	AX-87791752	2216613	2218848	0
ssa23	2218699	LOC106583897	0 067267643	AX-87082790	2216613	2218848	0
ssa23	2270511	LOC106583916	0 063029559	AX-87854671	2252506	2268200	-2312
ssa23	2283309	LOC106583915	0 068213071	AX-87810107	2279272	2286562	0
ssa23	2308857	LOC106583913	0 060738098	AX-87434124	2308500	2314580	0
ssa23	2327951	LOC123729882	0 059687355	AX-87761720	2340603	2343143	12652
ssa23	2344056	LOC106583914	0 057263581	AX-87094060	2343403	2354555	0
ssa23	2360731	LOC106583912	0 061294206	AX-87247549	2355421	2362108	0
ssa23	2368140	LOC106583912	0 062303763	AX-87058348	2355421	2362108	-6033
ssa23	2393019	LOC106583911	0 076686639	AX-87530118	2389132	2408482	0

ssa23	2436066	LOC106583908	0 066143602	AX-87236129	2428787	2443376	0
ssa23	2505134	rnf25	0 067332448	AX-87788904	2504743	2516698	0
ssa23	2529404	LOC123723737	0 061706436	AX-87826390	2520180	2530197	0
ssa23	2600986	LOC106583904	0 067995422	AX-87103270	2600359	2629041	0
ssa23	2629734	mk03	0 07217839	AX-87561113	2629161	2643711	0
ssa23	2630008	mk03	0 070799884	AX-86983773	2629161	2643711	0
ssa23	2631670	mk03	0 062873983	AX-87291109	2629161	2643711	0
ssa23	2640388	mk03	0 060596463	AX-87421127	2629161	2643711	0
ssa23	2681975	LOC106583903	0 06699729	AX-87197291	2668186	2690940	0
ssa23	2947816	LOC106583936	0 064626971	AX-87873005	2942347	2967459	0
ssa23	2982441	LOC106583937	0 064354271	AX-87394917	2968364	3019993	0
ssa23	3005468	LOC106583937	0 065337209	AX-87162229	2968364	3019993	0
ssa23	3019841	LOC106583937	0 059033189	AX-86988739	2968364	3019993	0
ssa23	3036436	LOC106583938	0 059848723	AX-87840770	3031589	3042910	0
ssa23	3043436	LOC106583938	0 060049706	AX-87856359	3031589	3042910	-527
ssa23	3115353	LOC106583942	0 060596463	AX-87681765	3110714	3127379	0
ssa23	3115646	LOC106583942	0 074849127	AX-86997998	3110714	3127379	0
ssa23	3115729	LOC106583942	0 070027524	AX-87734331	3110714	3127379	0
ssa23	3121577	LOC106583942	0 064025207	AX-87730744	3110714	3127379	0
ssa23	3154592	LOC100380384	0 066621115	AX-86983703	3128365	3159242	0
ssa23	3259004	LOC106583946	0 068996771	AX-87588621	3240330	3286119	0
ssa23	3260408	LOC106583946	0 068996771	AX-87424133	3240330	3286119	0
ssa23	3276464	LOC106583946	0 065759472	AX-86929741	3240330	3286119	0
ssa23	3380913	LOC106583948	0 065759472	AX-87715672	3373044	3499951	0
ssa23	3381417	LOC106583948	0 065759472	AX-87829317	3373044	3499951	0
ssa23	3382675	LOC106583948	0 062899713	AX-86938612	3373044	3499951	0
ssa23	3382784	LOC106583948	0 059559763	AX-87565281	3373044	3499951	0
ssa23	3391279	LOC106583948	0 067905176	AX-87345844	3373044	3499951	0
ssa23	3457986	LOC106583948	0 066937698	AX-87687901	3373044	3499951	0
ssa23	3466773	LOC106583948	0 066937698	AX-87453570	3373044	3499951	0
ssa23	3488009	LOC106583948	0 05832073	AX-87411652	3373044	3499951	0
ssa23	3496508	LOC106583948	0 059502277	AX-86979919	3373044	3499951	0
ssa23	3501782	LOC106583949	0 06434922	AX-87832206	3502605	3508828	823
ssa23	3537621	LOC106583950	0 07209625	AX-87448518	3543987	3696146	6366
ssa23	3679439	LOC106583950	0 061436991	AX-87482527	3543987	3696146	0
ssa23	3682828	LOC106583950	0 061436991	AX-87203511	3543987	3696146	0
ssa23	3713677	LOC123729850	0 061352182	AX-86980819	3706151	3710694	-2984
ssa23	3725681	LOC123729850	0 061352182	AX-87291944	3706151	3710694	-14988
ssa23	3745156	LOC123729850	0 061352182	AX-87253127	3706151	3710694	-34463
ssa23	3856177	LOC106583931	0 063399543	AX-86943695	3819125	3849831	-6347
ssa23	3894868	adgrl4	0 060518635	AX-87709750	3865593	3899600	0
ssa23	3926458	zranb2	0 059902232	AX-86944514	3928033	3943335	1575

ssa23	3929225	zranb2	0 059902232	AX-87391909	3928033	3943335	0
ssa23	3973152	LOC106583932	0 061221702	AX-87620961	3972553	4004015	0
ssa23	4171518	LOC106583955	0 057961703	AX-87385859	4168439	4175456	0
ssa23	4174832	LOC106583955	0 060539032	AX-87528941	4168439	4175456	0
ssa23	4174975	LOC106583955	0 060539032	AX-87311526	4168439	4175456	0
ssa23	4216040	LOC106583956	0 05919672	AX-87032514	4186695	4216974	0
ssa23	4271729	LOC106583958	0 058804032	AX-87816187	4262137	4288685	0
ssa23	4368224	LOC106583961	0 063139033	AX-87705887	4370887	4594513	2663
ssa23	4568952	LOC106583961	0 056708245	AX-87626935	4370887	4594513	0
ssa23	4742911	phb2	0 067776709	AX-87707383	4760709	4772183	17798
ssa23	4756099	phb2	0 064775306	AX-86948789	4760709	4772183	4610
ssa23	4763361	phb2	0 067776709	AX-87486464	4760709	4772183	0
ssa23	4848431	LOC106583966	0 068943819	AX-87721185	4825116	4891178	0
ssa23	4848513	LOC106583966	0 068943819	AX-87219159	4825116	4891178	0
ssa23	4848544	LOC106583966	0 068943819	AX-87099596	4825116	4891178	0
ssa23	4851423	LOC106583966	0 062633387	AX-87194948	4825116	4891178	0
ssa23	4852527	LOC106583966	0 067776709	AX-86950404	4825116	4891178	0
ssa23	4887570	LOC106583966	0 063223947	AX-87650128	4825116	4891178	0
ssa23	4888229	LOC106583966	0 062254793	AX-87500873	4825116	4891178	0
ssa23	4996120	LOC106583933	0 062824622	AX-87269535	4961451	5164659	0
ssa23	4998659	LOC106583933	0 062824622	AX-87292208	4961451	5164659	0
ssa23	5001735	LOC106583933	0 062824622	AX-87715906	4961451	5164659	0
ssa23	5034335	LOC106583933	0 068187739	AX-87672630	4961451	5164659	0
ssa23	5066890	LOC106583933	0 072554792	AX-87831158	4961451	5164659	0
ssa23	5198565	LOC106583981	0 058419772	AX-87181719	5218547	5231471	19982
ssa23	5227091	LOC106583981	0 060303326	AX-87228764	5218547	5231471	0
ssa23	5291033	nek7	0 058397016	AX-87180902	5270663	5407173	0
ssa23	5307770	nek7	0 059181487	AX-87033678	5270663	5407173	0
ssa23	5375800	nek7	0 068169392	AX-87030070	5270663	5407173	0
ssa23	5409671	nek7	0 058897328	AX-87405186	5270663	5407173	-2499
ssa23	5410352	nek7	0 058897328	AX-86950577	5270663	5407173	-3180
ssa23	5484285	LOC106583979	0 057698501	AX-87061095	5451191	5478986	-5300
ssa23	5484582	LOC106583979	0 060732931	AX-87322481	5451191	5478986	-5597
ssa23	5597167	LOC106583977	0 062386502	AX-87689968	5537220	5549031	-48137
ssa23	5597784	LOC106583977	0 062386502	AX-87641924	5537220	5549031	-48754
ssa23	6056017	LOC106583974	0 062280993	AX-86904160	6092942	6125674	36925
ssa23	6149959	LOC106583973	0 058470106	AX-87098467	6159826	6161179	9867
ssa23	6294651	LOC106583972	0 065313785	AX-87046911	6294340	6363393	0
ssa23	6391337	LOC123729872	0 065722224	AX-87229426	6371816	6376443	-14895
ssa23	6391902	LOC123729872	0 065722224	AX-87815151	6371816	6376443	-15460
ssa23	6392154	LOC123729872	0 065722224	AX-87510291	6371816	6376443	-15712
ssa23	6449250	LOC106584002	0 0575259	AX-87577142	6482989	6536932	33739

ssa23	6489215	LOC106584002	0 063679113	AX-87378314	6482989	6536932	0
ssa23	6552869	LOC106584004	0 056725859	AX-87355526	6551200	6558024	0
ssa23	6557013	LOC106584004	0 056725859	AX-87563205	6551200	6558024	0
ssa23	6654565	LOC106584006	0 067606233	AX-87358530	6651721	6696791	0
ssa23	6672861	LOC106584006	0 061801826	AX-87020095	6651721	6696791	0
ssa23	6726689	LOC106584007	0 067199279	AX-87355589	6698260	6715765	-10925
ssa23	6767944	LOC106584008	0 065054564	AX-87351005	6804212	6813838	36268
ssa23	6794897	LOC106584008	0 059249799	AX-87275938	6804212	6813838	9315
ssa23	6797545	LOC106584008	0 060121532	AX-86935810	6804212	6813838	6667
ssa23	6840943	LOC106584009	0 063341006	AX-87202934	6833243	6949691	0
ssa23	7202990	LOC106584010	0 060512959	AX-87341158	7098199	7258913	0
ssa23	7229313	LOC106584010	0 060276159	AX-87121416	7098199	7258913	0
ssa23	7239025	LOC106584010	0 061785526	AX-87858967	7098199	7258913	0
ssa23	7454609	LOC106584012	0 060400425	AX-87446529	7451573	7778705	0
ssa23	7454654	LOC106584012	0 066233955	AX-87290518	7451573	7778705	0
ssa23	7454787	LOC106584012	0 066233955	AX-87382864	7451573	7778705	0
ssa23	7460899	LOC106584012	0 058524259	AX-87246006	7451573	7778705	0
ssa23	7497196	LOC106584012	0 061502199	AX-87567044	7451573	7778705	0
ssa23	7509423	LOC106584012	0 0620599	AX-87718578	7451573	7778705	0
ssa23	7526545	LOC106584012	0 058303527	AX-87611249	7451573	7778705	0
ssa23	7561850	LOC106584012	0 058303527	AX-87851675	7451573	7778705	0
ssa23	7586659	LOC106584012	0 058303527	AX-87478820	7451573	7778705	0
ssa23	7632886	LOC106584012	0 061552986	AX-87610776	7451573	7778705	0
ssa23	7691571	LOC106584012	0 058303527	AX-87246317	7451573	7778705	0
ssa23	7700829	LOC106584012	0 058303527	AX-87503817	7451573	7778705	0
ssa23	7701082	LOC106584012	0 058359333	AX-87671451	7451573	7778705	0
ssa23	7701408	LOC106584012	0 058359333	AX-87683881	7451573	7778705	0
ssa23	7701634	LOC106584012	0 058303527	AX-87490150	7451573	7778705	0
ssa23	7703699	LOC106584012	0 058303527	AX-87833044	7451573	7778705	0
ssa23	7704096	LOC106584012	0 058303527	AX-87221652	7451573	7778705	0
ssa23	7928700	LOC106584015	0 058123816	AX-87275779	7956528	7983485	27828
ssa23	7957105	LOC106584015	0 057751931	AX-87406591	7956528	7983485	0
ssa23	7980830	LOC106584015	0 063466815	AX-86994688	7956528	7983485	0
ssa23	8318769	LOC106584023	0 060266494	AX-87176721	8322011	8585498	3242
ssa23	8475301	LOC106584023	0 062433928	AX-87497659	8322011	8585498	0
ssa23	8608611	LOC106584025	0 068722391	AX-87159858	8603186	8703439	0
ssa23	8913380	LOC106583988	0 05687901	AX-87726988	8884203	8916806	0
ssa23	8960832	LOC106583988	0 057299721	AX-87601259	8884203	8916806	-44027
ssa23	8962453	LOC106583988	0 05788129	AX-87820411	8884203	8916806	-45648
ssa23	8963187	LOC106583988	0 057299721	AX-87746782	8884203	8916806	-46382
ssa23	9159517	LOC106583989	0 057080044	AX-87364900	9156682	9247957	0
ssa23	9164685	LOC106583989	0 059892742	AX-87104073	9156682	9247957	0

ssa23	9172152	LOC106583989	0 065222901	AX-87048770	9156682	9247957	0
ssa23	9221352	LOC106583989	0 057402779	AX-87380133	9156682	9247957	0
ssa23	9221498	LOC106583989	0 057451414	AX-87765392	9156682	9247957	0
ssa23	9266457	LOC100194572	0 057623881	AX-86965558	9258041	9273562	0
ssa23	10263192	LOC106584037	0 057847057	AX-87790109	9980198	10290040	0
ssa23	15819928	LOC106584181	0 070612485	AX-86947590	15424229	15847640	0
ssa23	17496928	LOC106584157	0 066878856	AX-87073824	17459004	17515773	0
ssa23	17515057	LOC106584157	0 067632946	AX-87536029	17459004	17515773	0
ssa23	17554653	LOC106584157	0 062324337	AX-87098084	17459004	17515773	-38881
ssa23	17554833	LOC106584157	0 06142118	AX-86923420	17459004	17515773	-39061
ssa23	29098119	LOC106584350	0 059954467	AX-87340435	29041123	29197206	0
ssa23	29115978	LOC106584350	0 060158995	AX-86962867	29041123	29197206	0
ssa23	29189753	LOC106584350	0 059954467	AX-87738144	29041123	29197206	0
ssa23	29232255	trnav-aac	0 059954467	AX-87860490	29230950	29231022	-1234
ssa23	51616290	tbc1d2b	0 056594474	AX-87578768	51613383	51693230	0
ssa23	51786798	dnaja	0 058974726	AX-87527075	51764587	51782503	-4296
ssa23	51810936	gcnt3	0 06655241	AX-87043140	51819442	51821354	8506
ssa23	52028902	LOC106584844	0 061621088	AX-87781373	52027230	52029994	0
ssa24	16427594	LOC106584982	0 063178066	AX-87114539	16430363	16494727	2769
ssa24	16430433	LOC106584982	0 065022616	AX-87342053	16430363	16494727	0
ssa24	16502817	LOC106584981	0 08001852	AX-86941401	16500109	16530477	0
ssa24	17456914	LOC106585264	0 06360571	AX-87204492	17448819	17453345	-3570
ssa24	17500594	LOC106585266	0 064292831	AX-87418116	17486836	17532960	0
ssa24	23974310	LOC106585508	0 06484859	AX-87348512	23973370	23978570	0
ssa24	27944391	LOC106585720	0 077542837	AX-87050840	27978263	28038880	33872
ssa24	28032491	LOC106585720	0 066398925	AX-87048475	27978263	28038880	0
ssa24	28035724	LOC106585720	0 058387441	AX-87714096	27978263	28038880	0
ssa24	28150560	LOC106585718	0 065614426	AX-87358294	28103564	28165737	0
ssa24	28370513	LOC106585358	0 062013836	AX-87337008	28239415	28405198	0
ssa24	31129758	dab2ipa	0 06790755	AX-87391163	31123218	31253378	0
ssa24	31129830	dab2ipa	0 06790755	AX-87514551	31123218	31253378	0
ssa24	31132114	dab2ipa	0 06790755	AX-87308829	31123218	31253378	0
ssa24	31147984	dab2ipa	0 064133161	AX-87561176	31123218	31253378	0
ssa24	31174622	dab2ipa	0 06790755	AX-87102798	31123218	31253378	0
ssa24	31176761	dab2ipa	0 06790755	AX-87011284	31123218	31253378	0
ssa24	31182918	dab2ipa	0 06790755	AX-87826989	31123218	31253378	0
ssa24	36536621	LOC106585853	0 062218212	AX-87554585	36513786	36537307	0
ssa25	10274927	hibch	0 061171116	AX-87300071	10245288	10292902	0
ssa25	14012660	LOC106586201	0 061503494	AX-87522879	13898095	14018002	0
ssa25	14036625	LOC106586202	0 06455945	AX-87110721	14019782	14054022	0
ssa25	14037261	LOC106586202	0 06455945	AX-86955560	14019782	14054022	0
ssa25	14107662	LOC106586079	0 058946438	AX-87122161	14101114	14109698	0

ssa25	14108365	LOC106586079	0 058946438	AX-87784302	14101114	14109698	0
ssa25	14120357	LOC106586205	0 058946438	AX-87339547	14111795	14117500	-2858
ssa25	14123090	LOC106586206	0 058946438	AX-87641402	14124097	14187869	1007
ssa25	14125222	LOC106586206	0 060098188	AX-87769916	14124097	14187869	0
ssa25	17748881	LOC106586282	0 05750681	AX-87821599	17710947	17790466	0
ssa25	17748902	LOC106586282	0 05750681	AX-86997634	17710947	17790466	0
ssa25	17847718	LOC106586083	0 069844815	AX-87814775	17847211	17847843	0
ssa25	17856704	LOC106586083	0 067051234	AX-87317057	17847211	17847843	-8862
ssa25	17882938	sh3b4	0 05903569	AX-87396976	17869345	17914298	0
ssa25	17888154	sh3b4	0 065354078	AX-87242412	17869345	17914298	0
ssa25	17888211	sh3b4	0 06203628	AX-87520640	17869345	17914298	0
ssa25	17963020	arl4c	0 065984391	AX-87202109	17961325	17963307	0
ssa25	17965241	arl4c	0 057113437	AX-87721004	17961325	17963307	-1935
ssa25	17986362	arl4c	0 06770909	AX-87507993	17961325	17963307	-23056
ssa25	18141500	arhgap15	0 067424165	AX-87538223	18110300	18161470	0
ssa25	20028179	ube2f	0 063559761	AX-87155262	20010747	20099185	0
ssa25	25544428	LOC106586446	0 069321406	AX-87245689	25282279	25760645	0
ssa25	25562807	LOC106586446	0 057811661	AX-87831847	25282279	25760645	0
ssa25	25742684	LOC106586446	0 069769908	AX-87404072	25282279	25760645	0
ssa25	25997532	myl1	0 071689715	AX-87433649	26001428	26010964	3896
ssa25	26048743	map2	0 059955225	AX-87652411	26015454	26151901	0
ssa25	33239789	LOC106586568	0 062056876	AX-86946444	33240109	33242059	320
ssa25	33240280	LOC106586568	0 062056876	AX-87155801	33240109	33242059	0
ssa25	33241363	LOC106586568	0 067844634	AX-87814834	33240109	33242059	0
ssa25	33241956	LOC106586568	0 062056876	AX-87394047	33240109	33242059	0
ssa25	33242361	LOC106586568	0 06126466	AX-87073848	33240109	33242059	-303
ssa25	33242478	LOC106586568	0 061268002	AX-87785218	33240109	33242059	-420
ssa25	33244466	LOC106586568	0 057414999	AX-87375545	33240109	33242059	-2408
ssa25	49377729	LOC106586909	0 060531994	AX-87340340	49372980	49538994	0
ssa25	49436824	LOC106586909	0 062559878	AX-87516692	49372980	49538994	0
ssa26	1918617	ak8	0 058927569	AX-87558505	1899746	1980751	0
ssa26	8712205	zdh13	0 064037365	AX-87274748	8709218	8737034	0
ssa26	8714314	zdh13	0 064037365	AX-87236029	8709218	8737034	0
ssa26	8755439	LOC106587079	0 065301708	AX-87390935	8754030	8778106	0
ssa26	8755724	LOC106587079	0 065301708	AX-87417458	8754030	8778106	0
ssa26	8755753	LOC106587079	0 067522343	AX-87604136	8754030	8778106	0
ssa26	8757865	LOC106587079	0 067522343	AX-87076826	8754030	8778106	0
ssa26	8758108	LOC106587079	0 066793708	AX-87522189	8754030	8778106	0
ssa26	8759052	LOC106587079	0 065340389	AX-87614208	8754030	8778106	0
ssa26	8759056	LOC106587079	0 067697684	AX-86930152	8754030	8778106	0
ssa26	13486521	LOC106587295	0 062389611	AX-87609245	13472691	13488894	0
ssa26	17694334	LOC106587216	0 06637726	AX-87381973	17643452	17739400	0

ssa26	17926756	LOC106587212	0 065613503	AX-86942344	17867910	18026423	0
ssa26	22145677	LOC106587383	0 061215732	AX-87229068	22118440	22205683	0
ssa26	22246546	LOC106587382	0 062294982	AX-87500567	22235275	22268582	0
ssa26	22287889	tbb3	0 078811942	AX-87085674	22277957	22292532	0
ssa26	22287895	tbb3	0 078811942	AX-87480492	22277957	22292532	0
ssa26	30543864	LOC106587604	0 065230691	AX-87049905	30545085	30589010	1221
ssa27	1383447	LOC106588141	0 060953603	AX-87241872	1373424	1375576	-7872
ssa27	6500688	ext1b	0 078595177	AX-87756254	6441840	6551990	0
ssa27	6526446	ext1b	0 059188448	AX-87374243	6441840	6551990	0
ssa27	7872398	LOC106588469	0 077051811	AX-87544026	7761122	7936941	0
ssa27	11263132	coea1	0 060367706	AX-87813274	11243823	11268454	0
ssa27	12613388	LOC106588322	0 06072116	AX-87829273	12569778	12687016	0
ssa27	12629840	LOC106588322	0 059740891	AX-87499284	12569778	12687016	0
ssa27	12636110	LOC106588322	0 061135559	AX-87815654	12569778	12687016	0
ssa27	12641303	LOC106588322	0 05987389	AX-87428090	12569778	12687016	0
ssa27	25626620	tpd52	0 059775467	AX-87602293	25625582	25652513	0
ssa27	25698590	LOC106588817	0 061124563	AX-87417289	25654386	25672821	-25770
ssa27	28668683	LOC106588858	0 070249791	AX-87659314	28655085	28695581	0
ssa27	33421397	LOC123730919	0 060745097	AX-87407260	33384395	33411012	-10386
ssa28	964322	LOC106589273	0 065107035	AX-87043461	840313	974988	0
ssa28	2502514	LOC106589308	0 059444575	AX-87110398	2521093	2574951	18579
ssa28	8716500	LOC106589429	0 058071076	AX-87389254	8679743	8717262	0
ssa28	8719801	LOC106589429	0 05792056	AX-87085245	8679743	8717262	-2540
ssa28	28098492	LOC106589815	0 060618724	AX-87509562	28066497	28584298	0
ssa28	28117426	LOC106589815	0 065878402	AX-87225230	28066497	28584298	0
ssa28	28117527	LOC106589815	0 065878402	AX-86958221	28066497	28584298	0
ssa28	28128748	LOC106589815	0 065878402	AX-87809706	28066497	28584298	0
ssa28	28157501	LOC106589815	0 063541604	AX-86903102	28066497	28584298	0
ssa29	3223209	LOC106590042	0 073547531	AX-87440697	3211756	3315068	0
ssa29	3229712	LOC106590042	0 069732579	AX-87411065	3211756	3315068	0
ssa29	3233068	LOC106590042	0 074053409	AX-87031572	3211756	3315068	0
ssa29	3282337	LOC106590042	0 066961023	AX-87740636	3211756	3315068	0
ssa29	3497548	LOC106590040	0 073076077	AX-87347650	3497502	3550930	0
ssa29	3498015	LOC106590040	0 073076077	AX-86908105	3497502	3550930	0
ssa29	3498874	LOC106590040	0 076102824	AX-87845498	3497502	3550930	0
ssa29	3513667	LOC106590040	0 069448876	AX-86935590	3497502	3550930	0
ssa29	3549592	LOC106590040	0 065574448	AX-87247721	3497502	3550930	0
ssa29	9999745	LOC106590110	0 059172642	AX-87629496	9950047	9972777	-26969
ssa29	11417281	cacnb2a	0 059059224	AX-87748826	11397927	11509607	0
ssa29	11417798	cacnb2a	0 058823809	AX-87295514	11397927	11509607	0
ssa29	11428013	cacnb2a	0 059995184	AX-87084013	11397927	11509607	0
ssa29	11445281	cacnb2a	0 057029967	AX-87225782	11397927	11509607	0

ssa29	12444994	LOC106590155	0 069874364	AX-87419119	12454878	12469801	9884
ssa29	12464909	LOC106590155	0 070863739	AX-87262153	12454878	12469801	0
ssa29	12468067	LOC106590155	0 070863739	AX-87679592	12454878	12469801	0
ssa29	12469743	LOC106590155	0 070863739	AX-87322728	12454878	12469801	0
ssa29	12476537	LOC106590156	0 074517033	AX-87363350	12473318	12483890	0
ssa29	12477260	LOC106590156	0 078122149	AX-87200500	12473318	12483890	0
ssa29	14458750	LOC106590196	0 057394599	AX-87224856	14454960	14575810	0
ssa29	18307362	LOC106590287	0 074864357	AX-86979597	18288607	18329934	0
ssa29	26679129	LOC106590478	0 061140607	AX-87174705	26659278	26681809	0
ssa29	39717942	LOC106590681	0 057318204	AX-87628603	39741355	39869183	23413
ssa29	39855501	LOC106590681	0 080150393	AX-87329759	39741355	39869183	0
ssa29	39855933	LOC106590681	0 080150393	AX-87790854	39741355	39869183	0
ssa29	39902282	LOC106590681	0 071254847	AX-87689817	39741355	39869183	-33100
ssa29	41499388	LOC106590690	0 06080515	AX-87835028	41490884	41526596	0
ssa29	41513406	LOC106590690	0 059071884	AX-87785657	41490884	41526596	0
ssa29	41840523	vill	0 068530092	AX-87586553	41814615	41886156	0

Table S5 The top 1% of associations with modality (single versus multiple run timing peaks) in 11 populations of North American Atlantic salmon, identified using a partial redundancy analysis (pRDA) with population structure correction. Distance from the closest gene is reported alongside where in the genome the gene starts and ends.

Chrom.	Position	gene	loadings	SNP	start_gene	end_gene	distance SNP gene
ssa01	5996888	sos2	0 07717720	AX-87155661	5994111	6091819	0
ssa01	5996937	sos2	0 07717720	AX-87533038	5994111	6091819	0
ssa01	6291131	map4k5	0 06722054	AX-87422319	6157708	6305720	0
ssa01	14958952	LOC106565738	0 06392320	AX-87696214	14936801	14939638	-19315
ssa01	15217271	LOC106565829	0 07196583	AX-87181983	15230423	15237269	13152
ssa01	15270542	LOC106565829	0 07196583	AX-87300776	15230423	15237269	-33274
ssa01	15285312	LOC106565787	0 07594521	AX-87625890	15317636	15318765	32324
ssa01	16333805	LOC106566627	0 06315398	AX-87820081	16298094	16414823	0
ssa01	16358330	LOC106566627	0 05965113	AX-87685400	16298094	16414823	0
ssa01	16945230	LOC106568847	0 05694192	AX-87370989	16945215	16945776	0
ssa01	16946919	hnrnpua	0 05694192	AX-87762141	16947108	16960607	189
ssa01	16947458	hnrnpua	0 05694192	AX-87444796	16947108	16960607	0
ssa01	16948280	hnrnpua	0 05694192	AX-87039041	16947108	16960607	0
ssa01	20496932	LOC106573748	0 05557907	AX-87489330	20439438	20529977	0
ssa01	20508374	LOC106573748	0 05557907	AX-87051223	20439438	20529977	0
ssa01	20510486	LOC106573748	0 05557907	AX-86932382	20439438	20529977	0
ssa01	20541388	LOC106573748	0 05557907	AX-87535835	20439438	20529977	-11412
ssa01	20591103	LOC106576201	0 05557907	AX-87047575	20557389	20561884	-29220
ssa01	24412295	LOC106583710	0 07666985	AX-87148692	24403141	24412878	0
ssa01	24537269	LOC106581578	0 05748860	AX-87642669	24531808	24541294	0
ssa01	26515389	LOC106588600	0 05769949	AX-87726207	26494902	26569238	0
ssa01	28322991	LOC106593878	0 06761759	AX-87391880	28323686	28330013	695
ssa01	28348856	LOC106593970	0 05774846	AX-87542233	28354573	28389002	5717
ssa01	41276678	syne3	0 06097515	AX-87331790	41273188	41322193	0
ssa01	41277549	syne3	0 06104633	AX-87213067	41273188	41322193	0
ssa01	45772047	nrxn3a	0 06118510	AX-87677807	45762605	46196313	0
ssa01	52099584	emx2	0 05792792	AX-87695798	52134743	52139781	35159
ssa01	52729110	LOC106604835	0 06465017	AX-87055449	52677815	52686592	-42519
ssa01	61580774	LOC106605623	0 05831349	AX-87137034	61599245	61621154	18471
ssa01	61594476	LOC106605623	0 05831349	AX-87686944	61599245	61621154	4769
ssa01	62583060	LOC106607083	0 06174436	AX-87229844	62536940	62586540	0
ssa01	62589472	LOC106607083	0 06399692	AX-87358195	62536940	62586540	-2933
ssa01	62657283	LOC106607091	0 06421209	AX-87220657	62595243	62662977	0
ssa01	62658040	LOC106607091	0 06424021	AX-87660697	62595243	62662977	0
ssa01	62658153	LOC106607091	0 06421209	AX-87154506	62595243	62662977	0
ssa01	62728736	ogfrl1	0 06822546	AX-87557231	62729181	62734422	445

ssa01	62733747	ogfrl1	0 06875761	AX-87461687	62729181	62734422	0
ssa01	62737099	ogfrl1	0 08452832	AX-87797006	62729181	62734422	-2678
ssa01	62749857	LOC106607144	0 08452832	AX-87856964	62752829	62788697	2972
ssa01	62753766	LOC106607144	0 08870250	AX-87199456	62752829	62788697	0
ssa01	63309022	LOC100195610	0 07170836	AX-86944192	63294255	63305190	-3833
ssa01	63439950	LOC106607182	0 06530455	AX-86973505	63398435	63769511	0
ssa01	71411078	LOC106607988	0 07206078	AX-87042972	71400554	71497889	0
ssa01	73170521	mrps5	0 06815601	AX-87501190	73173074	73200101	2553
ssa01	73178593	mrps5	0 06000725	AX-86990628	73173074	73200101	0
ssa01	73195682	mrps5	0 05599806	AX-87063329	73173074	73200101	0
ssa01	74434115	LOC106605742	0 06316864	AX-87658902	74346023	74397698	-36418
ssa01	74472270	znf503	0 06341079	AX-87452068	74510121	74513018	37851
ssa01	74487771	znf503	0 06715622	AX-87735625	74510121	74513018	22350
ssa01	78825644	LOC106612672	0 06861786	AX-86986919	78810853	78826239	0
ssa01	86782825	macrod2	0 06282576	AX-87777393	86203733	87348365	0
ssa01	87657732	LOC106609120	0 05636143	AX-87598972	87657597	87669276	0
ssa01	87828078	LOC106609027	0 05809661	AX-87318459	87822595	87830694	0
ssa01	93250173	LOC106610476	0 07454661	AX-87752690	93288849	93473620	38676
ssa01	93462015	LOC106610476	0 07454661	AX-87401167	93288849	93473620	0
ssa01	93471868	LOC106610476	0 07454661	AX-87258731	93288849	93473620	0
ssa01	126666880	ppip2	0 05549023	AX-87319016	126650276	126671066	0
ssa01	126742201	LOC100380870	0 06498927	AX-87294080	126697527	126751174	0
ssa01	128106688	LOC106561946	0 06140163	AX-86979162	128106302	128110583	0
ssa01	129344496	LOC106562280	0 06424496	AX-87191631	129331367	129355683	0
ssa01	129346977	LOC106562280	0 06626954	AX-86917788	129331367	129355683	0
ssa01	129349354	LOC106562280	0 06173602	AX-87522668	129331367	129355683	0
ssa01	130921812	LOC123725137	0 06285800	AX-87501181	130942250	130950809	20438
ssa01	131330757	rai14	0 05626629	AX-87021426	131330980	131375780	223
ssa01	134062757	LOC106564485	0 05548008	AX-87755226	134065421	134070382	2664
ssa01	134111957	rnf185	0 06000349	AX-87647854	134107495	134112771	0
ssa01	134111957	LOC106564493	0 06000349	AX-87647854	134111824	134114837	0
ssa01	134281952	LOC106564592	0 05546894	AX-87589590	134237916	134287794	0
ssa01	147539354	LOC106567541	0 07292815	AX-87829433	147227423	147581276	0
ssa01	147561622	LOC106567541	0 05623971	AX-87232492	147227423	147581276	0
ssa01	153842534	LOC123726834	0 06098466	AX-87473798	153827442	153827569	-14966
ssa01	153843436	LOC123726834	0 06098466	AX-87463782	153827442	153827569	-15868
ssa01	162610360	LOC106571259	0 06993248	AX-87324612	162610281	162618554	0
ssa02	12214449	pgcp	0 05776007	AX-87621139	12184888	12218175	0
ssa02	12987393	ctnnb1	0 05779715	AX-87176439	12972758	13005222	0
ssa02	13919230	LOC106593045	0 07143021	AX-87230577	13921207	13927726	1977
ssa02	31434640	LOC106579919	0 06341801	AX-87381406	31413268	31435333	0
ssa02	31445973	LOC106579923	0 06341801	AX-87780133	31449385	31468074	3412

ssa02	31490512	LOC106579931	0	07293220	AX-87761599	31468969	31491780	0
ssa02	32277668	LOC106580132	0	05954407	AX-87780831	32274992	32278971	0
ssa02	33627183	LOC106581260	0	05547042	AX-87228144	33622422	33661268	0
ssa02	34379376	LOC106581105	0	06540827	AX-87153387	34375397	34397366	0
ssa02	35747319	LOC106581364	0	05551393	AX-87372735	35723519	35725418	-21902
ssa02	35775286	LOC106581785	0	06995769	AX-87376215	35782884	35804804	7598
ssa02	35827945	LOC106581796	0	06600935	AX-87549180	35817565	35829115	0
ssa02	38446057	LOC123729284	0	07349133	AX-87299891	38447052	38451090	995
ssa02	38506919	LOC106582654	0	06899772	AX-87817410	38501462	38507163	0
ssa02	38519656	LOC106582677	0	08064079	AX-87603981	38516080	38520321	0
ssa02	38526778	LOC106582686	0	08064079	AX-87388979	38527185	38557444	407
ssa02	38611573	LOC106582734	0	05805622	AX-87591436	38637471	38793408	25898
ssa02	38707990	LOC106582734	0	06128260	AX-86908245	38637471	38793408	0
ssa02	38733481	LOC106582734	0	06043347	AX-87297557	38637471	38793408	0
ssa02	45919520	LOC106584098	0	06000096	AX-87789539	45902368	45922048	0
ssa02	54638792	LOC106588327	0	05817362	AX-87679042	54627571	54697226	0
ssa02	57423310	LOC106587368	0	06213600	AX-87645026	57420600	57462328	0
ssa02	62686109	LOC106589173	0	05712631	AX-87216526	62681192	62686760	0
ssa02	66064995	LOC106590570	0	06483031	AX-87455474	66055043	66065800	0
ssa02	66066282	LOC106590570	0	06331668	AX-87421870	66055043	66065800	-483
ssa02	71455332	LOC123723933	0	05824692	AX-87807714	71448081	71455430	0
ssa03	4879989	LOC106596671	0	05863445	AX-87863526	4843550	4877449	-2541
ssa03	11559569	LOC106598456	0	05602558	AX-87169006	11561290	11631993	1721
ssa03	12421554	fytd1	0	05738110	AX-86961127	12417762	12427538	0
ssa03	13356935	LOC106598862	0	08340534	AX-87258833	13308412	13349183	-7753
ssa03	13360393	LOC106598864	0	08340534	AX-87132331	13370467	13380694	10074
ssa03	13828155	septin2	0	07674235	AX-87118054	13817909	13833925	0
ssa03	13864270	LOC106598901	0	06108869	AX-87433424	13854616	13869746	0
ssa03	13985732	LOC106598907	0	07589161	AX-87807463	13978785	14009378	0
ssa03	14042908	LOC106598913	0	05553661	AX-87042261	14044527	14049036	1619
ssa03	14045386	LOC106598913	0	05553661	AX-87322529	14044527	14049036	0
ssa03	14126724	LOC106598927	0	06352503	AX-87790115	14111135	14130508	0
ssa03	14194848	LOC106598932	0	05960095	AX-87708345	14157904	14200321	0
ssa03	14195665	LOC106598932	0	05960095	AX-87711838	14157904	14200321	0
ssa03	14485382	LOC106598943	0	08494539	AX-87004624	14480950	14508960	0
ssa03	14583180	LOC106598945	0	07507255	AX-87074026	14525574	14607755	0
ssa03	14668259	LOC106598949	0	08277385	AX-87825814	14650130	14684603	0
ssa03	14711089	tm16h	0	05740510	AX-87100577	14710107	14735447	0
ssa03	14777133	nr2f6a	0	09165274	AX-87458984	14768174	14787407	0
ssa03	16332052	LOC123741889	0	08487791	AX-87725560	16330878	16333036	0
ssa03	24040409	tnr	0	07724316	AX-87610622	23972883	24137786	0
ssa03	24040488	tnr	0	07724316	AX-87796780	23972883	24137786	0

ssa03	24044497	tnr	0	07593718	AX-87065961	23972883	24137786	0
ssa03	28513278	LOC106599252	0	07209741	AX-87848226	28545154	28691819	31876
ssa03	28527322	LOC106599252	0	07232607	AX-86933215	28545154	28691819	17832
ssa03	28552684	LOC106599252	0	05613497	AX-87170247	28545154	28691819	0
ssa03	28553004	LOC106599252	0	06119890	AX-87718728	28545154	28691819	0
ssa03	29982008	LOC106599706	0	05694904	AX-87684760	29909821	29993458	0
ssa03	29982214	LOC106599706	0	05694904	AX-87441324	29909821	29993458	0
ssa03	31737078	LOC106599757	0	06456238	AX-87669082	31677880	31803961	0
ssa03	31738255	LOC106599757	0	06277799	AX-87866979	31677880	31803961	0
ssa03	31823963	LOC106599757	0	07093387	AX-87788312	31677880	31803961	-20003
ssa03	31826549	LOC106599757	0	07093387	AX-87222130	31677880	31803961	-22589
ssa03	31832404	LOC106599757	0	07368537	AX-87297238	31677880	31803961	-28444
ssa03	31869685	LOC123741891	0	06814629	AX-87031089	31918137	31920488	48452
ssa03	31869844	LOC123741891	0	06814629	AX-87681534	31918137	31920488	48293
ssa03	31871667	LOC123741891	0	06814629	AX-87462247	31918137	31920488	46470
ssa03	31900427	LOC123741891	0	06814629	AX-87351567	31918137	31920488	17710
ssa03	31932871	LOC106599764	0	07301107	AX-87622456	31932232	31957002	0
ssa03	31960234	LOC106599772	0	06609778	AX-87623449	31958672	31960986	0
ssa03	31963100	LOC106599765	0	08290520	AX-87004810	31960989	31973047	0
ssa03	31968102	LOC106599765	0	07301107	AX-87467203	31960989	31973047	0
ssa03	33363568	LOC106599848	0	07851576	AX-87734012	33355172	33375197	0
ssa03	34572883	LOC106600432	0	07788008	AX-87101633	34561007	34629875	0
ssa03	34574042	LOC106600432	0	07788008	AX-87776551	34561007	34629875	0
ssa03	34574669	LOC106600432	0	06602405	AX-87389432	34561007	34629875	0
ssa03	36446027	LOC106600370	0	06284596	AX-87495678	36409611	36449247	0
ssa03	36598163	tox4a	0	05869565	AX-87366555	36590662	36599136	0
ssa03	36598884	tox4a	0	05599049	AX-87218699	36590662	36599136	0
ssa03	36600396	tox4a	0	05784225	AX-87785281	36590662	36599136	-1261
ssa03	36600884	LOC106600304	0	05935199	AX-87681577	36602541	36626294	1657
ssa03	51745700	atxn2l	0	05610513	AX-87447805	51725168	51746653	0
ssa03	51746707	lat	0	06042247	AX-87014079	51746710	51767698	3
ssa03	51772399	spns1	0	06833124	AX-87603787	51768603	51781018	0
ssa03	51799812	sgf29	0	06881680	AX-87428830	51797824	51809772	0
ssa03	52189662	LOC106600760	0	06745409	AX-87631777	52194972	52283481	5310
ssa03	52190052	LOC106600760	0	06086959	AX-87248542	52194972	52283481	4920
ssa03	57050214	LOC106600852	0	05937732	AX-86968166	57047858	57069530	0
ssa03	57050726	LOC106600852	0	06137331	AX-86934051	57047858	57069530	0
ssa03	57052912	LOC106600852	0	05683456	AX-87216045	57047858	57069530	0
ssa03	57053439	LOC106600852	0	06137331	AX-86955531	57047858	57069530	0
ssa03	57172138	LOC106600976	0	05644573	AX-87707370	57179267	57185122	7129
ssa03	57218845	LOC106600851	0	05761922	AX-87695983	57208045	57228854	0
ssa03	57249036	LOC106600851	0	06893417	AX-86927602	57208045	57228854	-20183

ssa03	60157555	LOC106601005	0	06342511	AX-87617608	60168218	60186639	10663
ssa03	72095822	LOC106601550	0	05549525	AX-87846891	72069540	72095636	-187
ssa03	72117811	LOC106601548	0	06609297	AX-87396360	72102774	72120244	0
ssa03	72155464	nucb1	0	07254450	AX-87341078	72153448	72166566	0
ssa03	72600933	kmt5c	0	06034445	AX-87319533	72593029	72605496	0
ssa03	72603413	kmt5c	0	07226464	AX-87466599	72593029	72605496	0
ssa03	72622499	LOC106601579	0	07472527	AX-87419041	72610803	72624092	0
ssa03	72691161	LOC106601582	0	07398246	AX-87239114	72676434	72703679	0
ssa03	72940225	LOC106601520	0	05918659	AX-87589158	72898990	72939386	-840
ssa03	72947349	LOC106601521	0	07892530	AX-87809176	72949943	72962964	2594
ssa03	73028108	LOC106601605	0	08214959	AX-87498801	73027873	73035806	0
ssa03	73032319	LOC106601605	0	07830433	AX-87866693	73027873	73035806	0
ssa03	73036450	LOC106601607	0	06443119	AX-87513139	73035375	73047410	0
ssa03	73036703	LOC106601607	0	06563437	AX-87004570	73035375	73047410	0
ssa03	73052489	LOC123741702	0	06114651	AX-87089040	73050491	73065304	0
ssa03	73105634	LOC106601603	0	05875817	AX-87063724	73096502	73100051	-5584
ssa03	73110078	LOC106601603	0	06568139	AX-87470558	73096502	73100051	-10028
ssa03	73113530	LOC106601601	0	06575645	AX-87780904	73123052	73209962	9522
ssa03	73123871	LOC106601601	0	07875080	AX-87056367	73123052	73209962	0
ssa03	73424393	LOC106601645	0	07363778	AX-87112036	73442672	73487034	18279
ssa03	73439156	LOC106601645	0	07149354	AX-87559401	73442672	73487034	3516
ssa03	73478666	LOC106601645	0	08207730	AX-87155793	73442672	73487034	0
ssa03	73565868	LOC106606818	0	05617587	AX-87637530	73532673	73565808	-61
ssa03	73575403	at7l3	0	05646383	AX-87284739	73566036	73575528	0
ssa03	74848546	LOC106601642	0	05971881	AX-86996933	74847786	74863966	0
ssa03	74986294	LOC106601711	0	07221842	AX-87540983	74984053	74990299	0
ssa03	75010891	LOC106595893	0	06849964	AX-87608747	75026804	75029485	15913
ssa03	76215523	LOC106606768	0	09262843	AX-86910506	76069834	76206898	-8626
ssa03	77587148	LOC123741727	0	05915880	AX-87247615	77572424	77583559	-3590
ssa03	80173015	LOC106606596	0	05834615	AX-87642712	80120117	80193633	0
ssa03	93961542	LOC106601948	0	05665115	AX-87544854	93957427	93961671	0
ssa04	10629241	LOC106602270	0	06855452	AX-87567951	10627477	10628175	-1067
ssa04	10629570	LOC106602270	0	06324064	AX-87442969	10627477	10628175	-1396
ssa04	11034195	LOC106602290	0	06319474	AX-87028580	11030441	11049908	0
ssa04	11038333	LOC106602290	0	05853750	AX-87693402	11030441	11049908	0
ssa04	11064095	LOC106602289	0	05561477	AX-87359388	11055524	11063497	-599
ssa04	11181580	LOC106602284	0	06187716	AX-86926715	11145929	11198531	0
ssa04	11220763	LOC106602285	0	06532042	AX-87695338	11217829	11243095	0
ssa04	11345179	LOC106602268	0	06714012	AX-87758705	11324083	11349710	0
ssa04	11345287	LOC106602268	0	06111028	AX-87065717	11324083	11349710	0
ssa04	11483411	LOC106602309	0	06714839	AX-87152505	11502992	11518124	19581
ssa04	11503842	LOC106602309	0	06008878	AX-87169863	11502992	11518124	0

ssa04	11568454	LOC106610333	0	06430036	AX-87583415	11522579	11588159	0
ssa04	11591726	LOC100194708	0	06407294	AX-87504866	11588023	11605271	0
ssa04	11591911	LOC100194708	0	05933320	AX-87478263	11588023	11605271	0
ssa04	11592066	LOC100194708	0	06382591	AX-86937343	11588023	11605271	0
ssa04	11593167	LOC100194708	0	06407294	AX-87763277	11588023	11605271	0
ssa04	12057660	LOC106602306	0	05601682	AX-86905973	12044701	12058339	0
ssa04	12059179	LOC106602307	0	05601682	AX-87260249	12058778	12071636	0
ssa04	12061460	LOC106602307	0	05601682	AX-87055374	12058778	12071636	0
ssa04	15761571	LOC106602374	0	07067461	AX-86930546	15638813	15727559	-34013
ssa04	15799424	LOC106602392	0	08092694	AX-87182681	15832727	15845143	33303
ssa04	20571189	LOC106602608	0	05640819	AX-87535919	20542461	20616010	0
ssa04	20571189	LOC106602617	0	05640819	AX-87535919	20567924	20580887	0
ssa04	20577432	LOC106602608	0	06618616	AX-87382710	20542461	20616010	0
ssa04	20577432	LOC106602617	0	06618616	AX-87382710	20567924	20580887	0
ssa04	20577512	LOC106602608	0	06618616	AX-86973456	20542461	20616010	0
ssa04	20577512	LOC106602617	0	06618616	AX-86973456	20567924	20580887	0
ssa04	20577760	LOC106602608	0	06618616	AX-87866416	20542461	20616010	0
ssa04	20577760	LOC106602617	0	06618616	AX-87866416	20567924	20580887	0
ssa04	20580225	LOC106602608	0	06034148	AX-87663098	20542461	20616010	0
ssa04	20580225	LOC106602617	0	06034148	AX-87663098	20567924	20580887	0
ssa04	20678570	LOC106602602	0	05779707	AX-87287290	20638678	20678927	0
ssa04	21173848	LOC106602642	0	06168324	AX-87803081	21164773	21172976	-873
ssa04	21212487	si:dkey-28b4.8	0	05743987	AX-86931767	21209319	21226283	0
ssa04	21684022	LOC106602574	0	09400429	AX-87013691	21681729	21691315	0
ssa04	21694192	LOC106602527	0	07033638	AX-87276289	21692062	21735738	0
ssa04	21852844	LOC106602568	0	05978574	AX-87781458	21847845	21852700	-145
ssa04	22044109	LOC106602561	0	07320632	AX-87581717	22023896	22105725	0
ssa04	22049669	LOC106602561	0	08315060	AX-87865897	22023896	22105725	0
ssa04	22049669	LOC106602562	0	08315060	AX-87865897	22046403	22064898	0
ssa04	22053718	LOC106602561	0	08315060	AX-87593241	22023896	22105725	0
ssa04	22053718	LOC106602562	0	08315060	AX-87593241	22046403	22064898	0
ssa04	22063019	LOC106602561	0	06746828	AX-87034258	22023896	22105725	0
ssa04	22063019	LOC106602562	0	06746828	AX-87034258	22046403	22064898	0
ssa04	22066710	LOC106602561	0	09046999	AX-87332030	22023896	22105725	0
ssa04	22071117	LOC106602561	0	08052608	AX-87794967	22023896	22105725	0
ssa04	22071900	LOC106602561	0	08144534	AX-87223564	22023896	22105725	0
ssa04	22075095	LOC106602561	0	05732336	AX-87738024	22023896	22105725	0
ssa04	22083972	LOC106602561	0	05680154	AX-86980580	22023896	22105725	0
ssa04	22084909	LOC106602561	0	05680154	AX-86994344	22023896	22105725	0
ssa04	22140505	tlr3	0	05972121	AX-87760687	22160209	22167987	19704
ssa04	22140712	tlr3	0	05972121	AX-87237073	22160209	22167987	19497
ssa04	22234375	mtnr1aa	0	05870297	AX-87375950	22232496	22291602	0

ssa04	22234569	mtnr1aa	0	05870297	AX-87167708	22232496	22291602	0
ssa04	23120043	LOC106602634	0	05713148	AX-87627889	23067556	23076737	-43307
ssa04	24591118	LOC106602686	0	06213040	AX-87498418	24588052	24594031	0
ssa04	43351168	LOC123742578	0	05925666	AX-87663802	43350202	43360702	0
ssa04	45993843	LOC106603266	0	05578632	AX-87627622	45993242	46009897	0
ssa04	46193659	LOC123742354	0	06097057	AX-87531479	46152135	46254646	0
ssa04	46446827	LOC106603271	0	05966968	AX-87770355	46422528	46496990	0
ssa04	46447446	LOC106603271	0	06037945	AX-87269336	46422528	46496990	0
ssa04	46452961	LOC106603271	0	05858435	AX-87110486	46422528	46496990	0
ssa04	52150189	LOC106603599	0	07080917	AX-87139822	52149922	52152821	0
ssa04	52502725	LOC106603590	0	06371945	AX-87243597	52495303	52511725	0
ssa04	52504027	LOC106603590	0	06371945	AX-87714068	52495303	52511725	0
ssa04	52539964	LOC106603589	0	06317536	AX-87451619	52550290	52599611	10326
ssa04	52551950	LOC106603589	0	06317536	AX-87293345	52550290	52599611	0
ssa04	52555111	LOC106603589	0	06317536	AX-87602325	52550290	52599611	0
ssa04	53307051	LOC106603560	0	06029940	AX-87619455	53279073	53319438	0
ssa04	53307269	LOC106603560	0	06029940	AX-87092358	53279073	53319438	0
ssa04	53322559	LOC106603560	0	06029940	AX-87218027	53279073	53319438	-3122
ssa04	54126021	LOC106603548	0	06703500	AX-87300046	54026198	54127794	0
ssa04	54151438	LOC106603547	0	06416277	AX-87059144	54144894	54154219	0
ssa04	54155868	LOC106603547	0	06416277	AX-87788024	54144894	54154219	-1650
ssa04	56619742	LOC106603516	0	05583340	AX-87240071	56464282	56621225	0
ssa04	56620152	LOC106603516	0	05583340	AX-86941353	56464282	56621225	0
ssa04	61382948	LOC106603444	0	05556469	AX-87002214	61357072	61374120	-8829
ssa04	65427567	LOC106603663	0	05762645	AX-87200250	65454640	65834185	27073
ssa04	72142128	LOC106603763	0	05617589	AX-87801648	72049139	72227981	0
ssa04	79524590	afap1l1b	0	06481045	AX-87002384	79503589	79546599	0
ssa04	79581751	LOC123742499	0	05595731	AX-87031718	79576292	79579816	-1936
ssa04	79594670	foxi3a	0	06210585	AX-87797389	79594462	79597405	0
ssa04	88130774	LOC106604040	0	07161686	AX-87037374	87866244	88440348	0
ssa05	11803710	LOC106604246	0	06419133	AX-87814467	11794642	11822583	0
ssa05	11824299	LOC106604248	0	06850914	AX-87390693	11824257	11830790	0
ssa05	11946783	LOC106604255	0	06357180	AX-87744589	11940640	11942954	-3830
ssa05	21508204	slc30a9	0	06505363	AX-87533091	21497781	21512399	0
ssa05	26458317	fgfbp1a	0	05859315	AX-87713724	26466696	26468182	8379
ssa05	26468077	fgfbp1a	0	07066345	AX-87680668	26466696	26468182	0
ssa05	26471053	LOC106604540	0	07066345	AX-87251689	26469168	26472257	0
ssa05	26482278	LOC106604539	0	07066345	AX-87062954	26480862	26592407	0
ssa05	26511847	LOC106604539	0	06628460	AX-87534421	26480862	26592407	0
ssa05	26521159	LOC106604539	0	07299013	AX-87304719	26480862	26592407	0
ssa05	26525147	LOC106604539	0	06616020	AX-87220603	26480862	26592407	0
ssa05	26525263	LOC106604539	0	06616020	AX-87733243	26480862	26592407	0

ssa05	26534707	LOC106604539	0 06717682	AX-87058659	26480862	26592407	0
ssa05	31879649	LOC106604630	0 05912916	AX-87551782	31886592	31965293	6943
ssa05	31886430	LOC106604630	0 06263161	AX-87519175	31886592	31965293	162
ssa05	33764536	LOC106604685	0 05598968	AX-87840009	33742354	33759880	-4657
ssa05	34305113	LOC106604600	0 07163550	AX-87188621	34265250	34271973	-33141
ssa05	34414161	LOC106604686	0 05942416	AX-87373563	34427261	34437289	13100
ssa05	41226044	LOC106604921	0 05861193	AX-87012096	41220416	41229303	0
ssa05	41228306	LOC106604921	0 06342511	AX-87419364	41220416	41229303	0
ssa05	41237225	LOC106604922	0 06283476	AX-87109009	41236823	41254653	0
ssa05	41265198	LOC106604923	0 06760409	AX-87090023	41261883	41275785	0
ssa05	48710971	LOC106605039	0 05756601	AX-87344211	48710801	48714924	0
ssa05	49096091	LOC106605038	0 05605729	AX-87399706	49078126	49096025	-67
ssa05	49098672	LOC106605025	0 06363634	AX-87120118	49098105	49107790	0
ssa05	61359138	pnp4b	0 05571518	AX-87659166	61334109	61354872	-4267
ssa05	62837074	LOC106605418	0 05947670	AX-87250971	62847111	62869624	10037
ssa05	62852909	LOC106605418	0 07025110	AX-87323000	62847111	62869624	0
ssa05	63710960	LOC106605391	0 05647218	AX-87442940	63660646	63876422	0
ssa05	65093273	LOC106605519	0 05982638	AX-87309540	65076567	65108435	0
ssa05	65111783	LOC106605518	0 05620495	AX-87450251	65110154	65122794	0
ssa05	68284339	nphs1	0 05837826	AX-87336436	68260599	68323414	0
ssa05	69689224	LOC106605674	0 05906924	AX-86974287	69688682	69692193	0
ssa05	69689238	LOC106605674	0 06656502	AX-87823039	69688682	69692193	0
ssa05	72204443	LOC106576313	0 06305788	AX-87065563	72206569	72232719	2126
ssa05	73926775	LOC106594979	0 05703476	AX-87069086	73853621	73925531	-1245
ssa05	76774106	LOC106605932	0 05986940	AX-87113258	76773961	76780362	0
ssa05	76808226	LOC106605928	0 05758990	AX-87121631	76794967	76813918	0
ssa05	76808383	LOC106605928	0 05961472	AX-86982366	76794967	76813918	0
ssa05	76855716	LOC106575377	0 05767232	AX-87256536	76864648	76881278	8932
ssa05	76917192	LOC106605934	0 06157131	AX-86965909	76902979	76922892	0
ssa05	76917904	LOC106605934	0 05853412	AX-87541737	76902979	76922892	0
ssa05	76926062	LOC106593883	0 06345279	AX-87824877	76925275	76938592	0
ssa05	76927563	LOC106593883	0 06069306	AX-87492384	76925275	76938592	0
ssa05	77283600	LOC106575309	0 06206424	AX-87744546	77128611	77283973	0
ssa05	77310329	LOC106605988	0 06175630	AX-87622802	77288225	77402238	0
ssa05	77415198	LOC106605961	0 06175630	AX-87307535	77405797	77416175	0
ssa05	77489499	LOC106576009	0 07070356	AX-87484296	77457912	77489598	0
ssa05	77560867	LOC106606002	0 06852900	AX-87816237	77563377	77564120	2510
ssa05	78467232	LOC106574458	0 05546677	AX-87279944	78439990	78472346	0
ssa05	81266646	LOC106606032	0 05671046	AX-87602707	81257510	81286395	0
ssa05	82848676	LOC123743190	0 05787272	AX-86980666	82855896	82857582	7220
ssa05	86137908	LOC106574054	0 06816062	AX-87697856	85987377	86168860	0
ssa05	86318267	LOC106591203	0 07203441	AX-87598619	86246990	86318597	0

ssa06	4919688	coil	0 07342231	AX-87360196	4919176	4938028	0
ssa06	14926012	LOC106606431	0 06012565	AX-87217634	14917976	14938381	0
ssa06	17498371	LOC106606469	0 08244950	AX-87581412	17484092	17509927	0
ssa06	21760181	LOC106598296	0 05563500	AX-86924676	21698725	21763660	0
ssa06	22323432	LOC106606667	0 05857862	AX-87074323	22272012	22332616	0
ssa06	24054310	LOC106606734	0 06475404	AX-87551136	24054099	24065898	0
ssa06	24054310	LOC106606736	0 06475404	AX-87551136	24054099	24056479	0
ssa06	24061763	LOC106606734	0 07181950	AX-87636564	24054099	24065898	0
ssa06	24061763	LOC106606735	0 07181950	AX-87636564	24056880	24063495	0
ssa06	24073567	LOC106606733	0 07534890	AX-87025160	24065880	24076738	0
ssa06	24075161	LOC106606733	0 07675058	AX-87348432	24065880	24076738	0
ssa06	24171828	LOC106606731	0 07228641	AX-87505552	24139370	24179103	0
ssa06	24172868	LOC106606731	0 06776323	AX-87260053	24139370	24179103	0
ssa06	25499962	LOC106606803	0 08251644	AX-87701585	25490375	25510557	0
ssa06	25534241	LOC106606805	0 05687098	AX-87643691	25527622	25533682	-560
ssa06	28146779	atxn7l3a	0 05617587	AX-87120794	28137527	28146634	-146
ssa06	28242553	LOC106601618	0 07444670	AX-87745041	28205652	28249132	0
ssa06	28245605	LOC106601618	0 07583605	AX-87712734	28205652	28249132	0
ssa06	28646231	ndua4	0 06586461	AX-87830048	28645703	28649720	0
ssa06	29686808	LOC106606904	0 05765983	AX-87661747	29685848	29687975	0
ssa06	29709697	LOC106593156	0 07366192	AX-87675907	29705009	29740792	0
ssa06	29727517	LOC106593156	0 06609297	AX-87084987	29705009	29740792	0
ssa06	37431697	LOC106607221	0 05584287	AX-87578316	37430489	37441402	0
ssa06	43757050	LOC106607494	0 07275017	AX-87230688	43714778	43783860	0
ssa06	44880146	LOC100194647	0 05727507	AX-87321029	44870933	44878736	-1411
ssa06	44898435	LOC106607471	0 07258228	AX-87598111	44902237	44910411	3802
ssa06	44900244	LOC106607471	0 07518212	AX-87623527	44902237	44910411	1993
ssa06	44975248	LOC106607468	0 05712531	AX-87203981	44970619	45152888	0
ssa06	44975338	LOC106607468	0 06658503	AX-87853386	44970619	45152888	0
ssa06	57438946	LOC106607695	0 06481587	AX-86950129	57434040	57437258	-1689
ssa06	57445510	mei4	0 06958073	AX-87232886	57442437	57489455	0
ssa06	57446829	mei4	0 06056648	AX-87562983	57442437	57489455	0
ssa06	57488999	mei4	0 05721699	AX-87624466	57442437	57489455	0
ssa06	57511213	mei4	0 05576081	AX-87591920	57442437	57489455	-21759
ssa06	57528620	nt5e	0 06097718	AX-87464809	57567307	57593508	38687
ssa06	57789423	LOC106607706	0 05670129	AX-87336493	57780656	57802819	0
ssa06	57789483	LOC106607706	0 05670129	AX-87459259	57780656	57802819	0
ssa06	62118942	tbc1d32	0 05778382	AX-86975299	62060122	62099999	-18944
ssa06	62137960	tbc1d32	0 05778382	AX-87742239	62060122	62099999	-37962
ssa06	62848167	nus1	0 06844395	AX-87348667	62826345	62850254	0
ssa06	62849222	nus1	0 06844395	AX-87859925	62826345	62850254	0
ssa06	62849437	nus1	0 06844395	AX-87402043	62826345	62850254	0

ssa06	64812187	LOC106607829	0 05584018	AX-87249837	64767645	64884011	0
ssa06	66855040	LOC106607876	0 06334992	AX-87102811	66859138	66879220	4098
ssa06	72554811	fig4a	0 05784633	AX-87840652	72451970	72558153	0
ssa06	75655801	LOC106608075	0 05590531	AX-87107213	75657086	75698883	1285
ssa06	75655903	LOC106608075	0 05590531	AX-87636530	75657086	75698883	1183
ssa06	86886699	sipa1l1	0 05888463	AX-87437222	86755502	87050572	0
ssa06	87342324	LOC106608268	0 06879344	AX-87064950	87314739	87343083	0
ssa06	87363817	pigh	0 06669427	AX-87570595	87343316	87345365	-18453
ssa06	87431871	ino80	0 05819770	AX-86928549	87429721	87545590	0
ssa06	87454379	ino80	0 06216773	AX-86996688	87429721	87545590	0
ssa06	87563937	dll4	0 06331523	AX-87492739	87567582	87588409	3645
ssa06	88237869	syndig1l	0 05669664	AX-87615092	88269109	88359689	31240
ssa07	4350210	LOC106608443	0 05635422	AX-87086620	4252567	4391720	0
ssa07	17008444	LOC106608609	0 06795351	AX-87870519	17020005	17062325	11561
ssa07	19097708	LOC106608606	0 05919965	AX-87106267	18593920	19250220	0
ssa07	23322130	serpinh2	0 06458322	AX-87491514	23298844	23320301	-1830
ssa07	23324419	dnah2	0 07204918	AX-87308712	23324476	23532733	57
ssa07	45200889	LOC106609368	0 07171320	AX-87573533	45190810	45203363	0
ssa07	45868297	ccnd2	0 05736819	AX-87700557	45872944	45884485	4647
ssa07	49419232	LOC106609519	0 06198346	AX-87014489	49406931	49452359	0
ssa07	55624051	LOC106596138	0 07277512	AX-87391357	55618041	55644598	0
ssa07	56028595	LOC106609755	0 06106042	AX-87303142	56028124	56032636	0
ssa07	56037775	LOC106609755	0 05570517	AX-87338856	56028124	56032636	-5140
ssa08	1680304	LOC106610024	0 05803790	AX-87409077	1565082	1689967	0
ssa08	3084336	LOC106609934	0 05612246	AX-87831869	2509770	3266673	0
ssa08	5428308	LOC123744321	0 05736087	AX-87106718	5435948	5436003	7640
ssa08	6143992	LOC106609937	0 05587690	AX-87677767	6175476	6234380	31484
ssa08	6184011	LOC106609937	0 05568181	AX-87080487	6175476	6234380	0
ssa08	6536071	LOC106609867	0 05722232	AX-87192014	6536409	6542577	338
ssa08	13809825	LOC106610201	0 05545289	AX-87290942	13814748	13818522	4923
ssa08	13951409	LOC106610200	0 05847474	AX-87335216	13956295	13958250	4886
ssa08	17044724	LOC106610360	0 05648187	AX-87637556	17023410	17091527	0
ssa09	170976	LOC106610635	0 05758629	AX-87463933	167756	218828	0
ssa09	233059	LOC106610585	0 06395418	AX-87109287	238782	295126	5723
ssa09	5584079	LOC106610595	0 05933252	AX-87109946	5564353	5965997	0
ssa09	6117133	LOC106610594	0 06204498	AX-87844417	6149596	6183905	32463
ssa09	6183835	LOC106610594	0 06582103	AX-86939847	6149596	6183905	0
ssa09	6186559	LOC106610594	0 06582103	AX-87757070	6149596	6183905	-2655
ssa09	6194099	si:ch211-266g18.6	0 06812030	AX-87633304	6193892	6259082	0
ssa09	6194126	si:ch211-266g18.6	0 05821762	AX-87681285	6193892	6259082	0
ssa09	6311269	fut9a	0 06479332	AX-87590537	6277565	6304745	-6525
ssa09	6311279	fut9a	0 06479332	AX-87305793	6277565	6304745	-6535

ssa09	6329798	LOC106610578	0 06757061	AX-87699729	6333536	6352018	3738
ssa09	9976701	LOC123744589	0 06046116	AX-87430146	9980719	9982134	4018
ssa09	9994725	LOC123744590	0 05721597	AX-87027293	9994487	10018416	0
ssa09	11230427	asap2a	0 05667405	AX-87556481	11154408	11266116	0
ssa09	11250914	asap2a	0 05755674	AX-87078194	11154408	11266116	0
ssa09	16980343	LOC106610806	0 06290104	AX-87788784	16976431	16981815	0
ssa09	16981699	LOC106610806	0 06290104	AX-87448191	16976431	16981815	0
ssa09	16981839	LOC106610806	0 06290104	AX-87126978	16976431	16981815	-25
ssa09	18760213	LOC106610864	0 07395963	AX-87230997	18720490	18771864	0
ssa09	18767331	LOC106610864	0 06490687	AX-87754609	18720490	18771864	0
ssa09	18850711	LOC106610859	0 08123130	AX-87488060	18808033	18858961	0
ssa09	18913699	LOC106610858	0 06429389	AX-87185507	18870473	18913022	-678
ssa09	18915209	LOC106610857	0 07013914	AX-87388738	18916991	18923965	1782
ssa09	18918077	LOC106610857	0 05726476	AX-87571531	18916991	18923965	0
ssa09	18928801	LOC106610856	0 07492227	AX-87598987	18932752	18945691	3951
ssa09	21419170	LOC106611047	0 07345893	AX-86959577	21387525	21401476	-17695
ssa09	21506998	LOC106611046	0 07414415	AX-87813325	21503757	21536255	0
ssa09	21584992	mlh3	0 07197543	AX-87511746	21580839	21587762	0
ssa09	21585463	mlh3	0 06896795	AX-87228087	21580839	21587762	0
ssa09	21588445	acyp1	0 06625301	AX-87517867	21587836	21589286	0
ssa09	21597734	LOC106610925	0 06843163	AX-87707072	21595813	21605615	0
ssa09	21656870	LOC106611036	0 08681359	AX-87860158	21627655	21683481	0
ssa09	25808006	LOC106611085	0 07972297	AX-87349115	25770137	25771178	-36829
ssa09	25916004	LOC106610930	0 07385313	AX-87574498	25875902	25914662	-1343
ssa09	29518780	LOC106611216	0 07720835	AX-87073203	29511103	29511744	-7037
ssa09	29680977	nrde2	0 05789623	AX-87766538	29672344	29688723	0
ssa09	29686562	nrde2	0 05789623	AX-86997351	29672344	29688723	0
ssa09	29717996	calm1a	0 05868023	AX-87832704	29691049	29709198	-8799
ssa09	29778317	LOC106611152	0 05604379	AX-87869851	29735260	29836922	0
ssa09	29956838	LOC106611173	0 06697719	AX-87802137	29843915	29967519	0
ssa09	30127957	LOC100380709	0 05708148	AX-87810926	30098734	30105027	-22931
ssa09	33421777	LOC106611240	0 06276289	AX-87277868	33396891	33399958	-21820
ssa09	33822261	LOC106611246	0 05558386	AX-87406001	33771699	33828065	0
ssa09	38990083	LOC106611330	0 07111932	AX-87543529	38958574	39020210	0
ssa09	39561962	sash1b	0 05588985	AX-87454582	39467267	39582304	0
ssa09	72453734	LOC106611816	0 06133005	AX-87181352	72372609	72704637	0
ssa09	77557303	LOC106611956	0 06067216	AX-87294634	77554173	78013009	0
ssa09	78982013	LOC123744796	0 06768881	AX-86984491	78884069	79161090	0
ssa09	81116657	si:ch211-159i8.4	0 06439791	AX-87496027	81131910	81141836	15253
ssa09	81188956	LOC106611996	0 05790467	AX-87046104	81171128	81178064	-10893
ssa09	81549999	al3a2	0 05709803	AX-87660923	81594206	81601802	44207
ssa09	110930141	LOC106612406	0 05608090	AX-87124382	110885909	110940387	0

ssa09	110938834	LOC106612406	0 05608090	AX-87331352	110885909	110940387	0
ssa09	110986747	LOC106612343	0 05679417	AX-87104452	110980483	111069157	0
ssa09	110989751	LOC106612343	0 05679417	AX-87223597	110980483	111069157	0
ssa09	114802939	LOC106612494	0 05721800	AX-87571863	114783431	114889387	0
ssa09	119974870	LOC106612644	0 05835896	AX-87285474	119972121	119974134	-737
ssa09	119978502	LOC106612643	0 05738043	AX-87526310	119977726	120102168	0
ssa09	128315455	LOC106612854	0 07637079	AX-87046336	128313745	128324097	0
ssa09	129065211	LOC106612835	0 05833890	AX-87735511	129004458	129088072	0
ssa09	129254965	LOC106612831	0 06121669	AX-87579195	129178607	129263417	0
ssa09	129355393	nips2	0 06017680	AX-87647076	129376817	129384298	21424
ssa09	129452433	LOC106612828	0 05705067	AX-87319467	129445843	129459857	0
ssa09	134128365	LOC106612987	0 05885998	AX-87392330	134113431	134128782	0
ssa09	148208835	arhgef17	0 05683082	AX-87120961	148164177	148264422	0
ssa10	1234328	LOC106613408	0 06156023	AX-87360928	1151348	1371375	0
ssa10	10734138	LOC106613502	0 05726685	AX-87429612	10716244	10733212	-927
ssa10	11046201	LOC106613507	0 08616572	AX-86955047	10861980	11144351	0
ssa10	11048627	LOC106613507	0 06576614	AX-87370114	10861980	11144351	0
ssa10	11048718	LOC106613507	0 08118022	AX-87198531	10861980	11144351	0
ssa10	14243120	LOC106613561	0 06512649	AX-87872560	14096955	14234983	-8138
ssa10	14249746	LOC106613561	0 07552306	AX-87224901	14096955	14234983	-14764
ssa10	14670980	LOC106613570	0 07547455	AX-87829564	14505668	14674636	0
ssa10	14673579	LOC106613570	0 07701915	AX-86955470	14505668	14674636	0
ssa10	14673916	LOC106613570	0 06957632	AX-86986211	14505668	14674636	0
ssa10	14728904	LOC106613573	0 06752157	AX-87148953	14676771	14707742	-21163
ssa10	16590846	LOC106613625	0 05795242	AX-87265102	16596744	16598901	5898
ssa10	16641955	homer3b	0 08598444	AX-87344166	16602709	16662878	0
ssa10	16690640	LOC106613628	0 07637797	AX-87605250	16686386	16695175	0
ssa10	16739629	LOC106613630	0 06696461	AX-87630643	16747404	16749027	7775
ssa10	16757279	LOC106613631	0 06931935	AX-87815161	16750154	16773567	0
ssa10	16811447	LOC106613633	0 08883999	AX-86938017	16807494	16974169	0
ssa10	16811506	LOC106613633	0 05550013	AX-87542529	16807494	16974169	0
ssa10	16992121	LOC106613635	0 05852985	AX-87398789	16985055	16997235	0
ssa10	17025221	LOC106613636	0 06238242	AX-87796632	17017910	17070918	0
ssa10	27573596	soat1	0 05584473	AX-87871001	27562046	27573992	0
ssa10	29326477	LOC106613928	0 05663660	AX-86959458	29309025	29336594	0
ssa10	29330902	LOC106613928	0 05663660	AX-86983322	29309025	29336594	0
ssa10	30541841	prdx6	0 06660947	AX-87290739	30573687	30576133	31846
ssa10	30574164	prdx6	0 06660947	AX-87544630	30573687	30576133	0
ssa10	40945967	naprt	0 05645144	AX-87612876	40932507	40950207	0
ssa10	42790097	cssa10h1orf52	0 07173060	AX-87194218	42789045	42793834	0
ssa10	42809290	ddah1	0 07173060	AX-87226783	42808514	42948242	0
ssa10	42825519	ddah1	0 06535428	AX-87402823	42808514	42948242	0

ssa10	42846912	ddah1	0	06327173	AX-87587341	42808514	42948242	0
ssa10	43419968	LOC106560455	0	05564700	AX-87080415	43372651	43421589	0
ssa10	59598155	LOC106560669	0	05703337	AX-87830518	59504591	59600127	0
ssa10	72850446	LOC106560749	0	05645904	AX-86922391	72776472	72850968	0
ssa10	81711188	LOC106560972	0	05696856	AX-87235189	81603503	81712011	0
ssa10	82039996	tesmin	0	05993235	AX-87820820	82038395	82051867	0
ssa10	82120156	lrp5	0	05666112	AX-87862043	82118724	82171160	0
ssa10	86174413	LOC106560943	0	06072292	AX-87733008	86174389	86185353	0
ssa10	86244237	LOC106560867	0	06966068	AX-87865651	86193160	86332516	0
ssa10	87117893	LOC100195319	0	05791013	AX-87590188	87107915	87143610	0
ssa10	115624650	LOC106561633	0	06290117	AX-87010501	115593279	115625873	0
ssa10	115625435	LOC106561633	0	06290117	AX-87235678	115593279	115625873	0
ssa11	13410163	LOC106562037	0	05760042	AX-86996312	13371025	13389522	-20642
ssa11	13425892	LOC106562037	0	06275784	AX-87644880	13371025	13389522	-36371
ssa11	13727438	si:ch211-186j3.6	0	05547753	AX-87696313	13716361	13972076	0
ssa11	13728965	si:ch211-186j3.6	0	05547753	AX-86964816	13716361	13972076	0
ssa11	21814302	LOC106562269	0	05967355	AX-87397938	21786937	21820042	0
ssa11	21816880	LOC106562269	0	06458499	AX-87126951	21786937	21820042	0
ssa11	29408325	LOC106587580	0	05685240	AX-87590851	29293175	29455620	0
ssa11	39543394	LOC106568778	0	06378870	AX-87004849	39469405	39655153	0
ssa11	71460680	LOC106563054	0	05904152	AX-87783883	71419079	71435312	-25369
ssa11	71682674	LOC106563053	0	06387517	AX-87707576	71638672	71722037	0
ssa11	81854191	LOC106563268	0	05661356	AX-87761986	81562761	82096555	0
ssa11	81859625	LOC106563268	0	05661356	AX-87362264	81562761	82096555	0
ssa11	83429440	LOC106563260	0	05968700	AX-87803602	83429857	83481825	417
ssa11	83815713	LOC106563248	0	05581810	AX-87753155	83809646	83813223	-2491
ssa11	83853301	LOC106563247	0	05581810	AX-87686507	83839437	83874644	0
ssa11	83973597	LOC106563245	0	07907054	AX-87335239	83902292	84024245	0
ssa11	83974062	LOC106563245	0	07693948	AX-87408958	83902292	84024245	0
ssa11	84031963	LOC106563244	0	06834842	AX-87039647	84035483	84038877	3520
ssa11	84089939	LOC106563243	0	06116081	AX-87293215	84047833	84097252	0
ssa11	84129738	arrdc1b	0	06990910	AX-87127412	84104577	84161073	0
ssa11	84157692	arrdc1b	0	06961239	AX-87488798	84104577	84161073	0
ssa11	84160331	arrdc1b	0	06449151	AX-86967272	84104577	84161073	0
ssa11	84160748	arrdc1b	0	06933211	AX-87115857	84104577	84161073	0
ssa11	84190636	mamdc4	0	05815319	AX-87651222	84177193	84191138	0
ssa11	84414610	LOC106563236	0	05841524	AX-87184460	84405737	84418016	0
ssa11	87752269	prnpb	0	05703079	AX-87814146	87749428	87753295	0
ssa11	88089268	LOC106563176	0	06930866	AX-87229897	88081469	88089254	-15
ssa11	88179912	cldx	0	07388747	AX-87534318	88175829	88183032	0
ssa11	88297185	LOC106563170	0	07548401	AX-87261963	88220293	88298702	0
ssa11	88429440	LOC106563375	0	07268673	AX-87427244	88428339	88519894	0

ssa11	88456898	LOC106563375	0	07344965	AX-87858616	88428339	88519894	0
ssa11	98629415	supt6h	0	06097187	AX-87050501	98610092	98667708	0
ssa11	100421085	LOC106563808	0	05629855	AX-87843017	100412263	100422296	0
ssa11	101656005	aprin	0	05987975	AX-87049725	101626728	101666586	0
ssa11	101799030	stard13a	0	06948940	AX-87279778	101814146	101991555	15116
ssa11	101814817	stard13a	0	07691237	AX-87857897	101814146	101991555	0
ssa12	24474435	LOC106564615	0	06733395	AX-87391330	24455186	24491601	0
ssa12	24476040	LOC106564615	0	06896299	AX-87572244	24455186	24491601	0
ssa12	24481673	LOC106564615	0	07213837	AX-87370951	24455186	24491601	0
ssa12	24481680	LOC106564615	0	07213837	AX-87523210	24455186	24491601	0
ssa12	24485855	LOC106564615	0	05942202	AX-87794226	24455186	24491601	0
ssa12	32178708	LOC106564916	0	05908401	AX-87622825	32134484	32141516	-37193
ssa12	32251794	kpra	0	05707389	AX-87398136	32248050	32275892	0
ssa12	32275726	kpra	0	05562795	AX-87559322	32248050	32275892	0
ssa12	36638024	hebp1	0	06355135	AX-87084415	36627784	36640635	0
ssa12	36674010	LOC106564807	0	06434757	AX-87154930	36641121	36680565	0
ssa12	36674373	LOC106564807	0	06434757	AX-87572959	36641121	36680565	0
ssa12	36863033	LOC106564801	0	06341509	AX-87357890	36839023	36912681	0
ssa12	36934450	LOC106564799	0	05967534	AX-87342004	36928305	36943800	0
ssa12	36934510	LOC106564799	0	05596836	AX-86996746	36928305	36943800	0
ssa12	39570566	mmp24	0	06324665	AX-87767894	39593542	39699466	22976
ssa12	39626259	mmp24	0	06324665	AX-87103495	39593542	39699466	0
ssa12	39626786	mmp24	0	06626370	AX-87580417	39593542	39699466	0
ssa12	39734704	LOC106565023	0	06694795	AX-87565010	39734230	39739781	0
ssa12	39866033	LOC106565027	0	05606112	AX-87113765	39831839	39880589	0
ssa12	52032905	LOC106565168	0	05958614	AX-87437856	51955772	52061608	0
ssa12	52504869	LOC106565157	0	06278989	AX-87614652	52505487	52517053	618
ssa12	58222264	kbtbd12	0	05837990	AX-87802130	58214679	58223617	0
ssa12	58234999	slc26a6l	0	05918178	AX-86955067	58228235	58237817	0
ssa12	58238746	LOC106565486	0	05837990	AX-87212705	58237958	58255748	0
ssa12	59303517	cita	0	05701086	AX-87461102	59297545	59349281	0
ssa12	59503866	LOC106565381	0	07370377	AX-87772012	59496809	59537572	0
ssa12	59518516	LOC106565381	0	06970210	AX-86925498	59496809	59537572	0
ssa12	61699112	LOC106565451	0	06063296	AX-87836207	61688160	61702390	0
ssa12	61750656	LOC106565452	0	05740678	AX-87770402	61713245	61768652	0
ssa12	61758417	LOC106565452	0	05574263	AX-86926596	61713245	61768652	0
ssa12	69427634	LOC106565694	0	05855552	AX-87806849	69371444	69438802	0
ssa12	69448940	LOC106565694	0	05615955	AX-87494106	69371444	69438802	-10139
ssa12	72385008	LOC106565889	0	05547767	AX-87534157	72349770	72400322	0
ssa12	75384953	adamts9	0	05665275	AX-87397028	75309854	75352207	-32747
ssa12	100038377	LOC106566258	0	06007841	AX-87609474	99918182	100039563	0
ssa13	14148984	LOC106566490	0	05838876	AX-87079628	14018202	14114855	-34130

ssa13	26211689	LOC106566694	0	05928132	AX-87825478	26190519	26219001	0
ssa13	28851272	LOC106566818	0	05655945	AX-87673194	28823564	28853519	0
ssa13	47148919	LOC106566949	0	07635507	AX-87102981	46821667	47230665	0
ssa13	47155480	LOC106566949	0	08633584	AX-87579659	46821667	47230665	0
ssa13	47155731	LOC106566949	0	08633584	AX-87105955	46821667	47230665	0
ssa13	47173676	LOC106566949	0	06561567	AX-87359725	46821667	47230665	0
ssa13	47289622	LOC106566948	0	06081065	AX-87360944	47246886	47349700	0
ssa13	47361383	LOC106566946	0	08526704	AX-87872713	47352005	47361915	0
ssa13	47397546	LOC106566945	0	08526704	AX-87059354	47422949	47571753	25403
ssa13	47421995	LOC106566945	0	06241053	AX-87595249	47422949	47571753	954
ssa13	47437798	LOC106566945	0	06241053	AX-87310721	47422949	47571753	0
ssa13	48023140	LOC106566938	0	06943913	AX-87490709	47998449	48023613	0
ssa13	48384906	skia	0	07548384	AX-87102248	48372998	48451305	0
ssa13	48390331	skia	0	08184358	AX-87671188	48372998	48451305	0
ssa13	64326986	LOC106567559	0	05596940	AX-87703507	64149351	64515196	0
ssa13	78365129	ptrh2	0	06491293	AX-87863108	78355993	78357821	-7309
ssa13	81577882	LOC106567909	0	06158356	AX-87589889	81569919	81587985	0
ssa13	81602909	LOC106567908	0	06329616	AX-87332996	81592859	81614392	0
ssa13	81624824	LOC106567906	0	05958396	AX-86940085	81615136	81629513	0
ssa13	81626465	LOC106567906	0	05958396	AX-87611963	81615136	81629513	0
ssa13	81628593	LOC106567906	0	05958396	AX-87264424	81615136	81629513	0
ssa13	87236916	msi2h	0	06014372	AX-87034134	86957145	87315927	0
ssa13	88336804	LOC106568088	0	05876930	AX-86980350	88349637	88358136	12833
ssa13	88783403	LOC106568092	0	06054607	AX-87870047	88783797	88801210	394
ssa13	88867135	LOC106568094	0	06152448	AX-87292448	88873280	89136258	6145
ssa13	88878987	LOC106568094	0	09500183	AX-87287148	88873280	89136258	0
ssa13	88883866	LOC106568094	0	09500183	AX-87800238	88873280	89136258	0
ssa13	88888242	LOC106568094	0	09844961	AX-87209562	88873280	89136258	0
ssa13	88930842	LOC106568094	0	09500183	AX-86955600	88873280	89136258	0
ssa13	88931193	LOC106568094	0	09271081	AX-86967885	88873280	89136258	0
ssa13	89140463	LOC106568094	0	06758332	AX-87362059	88873280	89136258	-4206
ssa13	89173247	LOC106568109	0	07203933	AX-87475520	89150551	89394069	0
ssa13	89697591	LOC106568095	0	06261757	AX-87199099	89605090	89750602	0
ssa13	89701858	LOC106568095	0	06745145	AX-87646900	89605090	89750602	0
ssa13	90036356	LOC106568123	0	08683943	AX-87590074	90022680	90025313	-11044
ssa13	90043002	LOC123726091	0	05956044	AX-87353719	90059855	90061102	16853
ssa13	90045347	LOC123726091	0	08683943	AX-87091570	90059855	90061102	14508
ssa13	90046647	LOC123726091	0	06079524	AX-86971595	90059855	90061102	13208
ssa13	91179584	LOC106568209	0	05801122	AX-87830755	91172757	91184571	0
ssa13	91183430	LOC106568209	0	05801122	AX-87849259	91172757	91184571	0
ssa13	91186575	LOC106568209	0	05801122	AX-87707558	91172757	91184571	-2005
ssa13	91186789	LOC106568209	0	05801122	AX-87153167	91172757	91184571	-2219

ssa13	91710546	LOC106568261	0 05669139	AX-87210394	91687623	91716774	0
ssa13	92330748	LOC106568246	0 05751838	AX-87834537	92263436	92348572	0
ssa13	96989519	LOC106568328	0 05577972	AX-87807020	96978799	96993309	0
ssa13	97781434	LOC106568341	0 06718807	AX-87129988	97749916	97800488	0
ssa13	97927180	LOC106568344	0 07322144	AX-87374357	97926530	97938494	0
ssa13	97929297	LOC106568344	0 07322144	AX-87708947	97926530	97938494	0
ssa13	97989059	LOC106568347	0 07845635	AX-87439357	97984308	97996157	0
ssa13	97998917	LOC106568348	0 07696179	AX-87751270	97998526	98019956	0
ssa13	98000178	LOC106568348	0 07696179	AX-87662621	97998526	98019956	0
ssa13	98043921	LOC106568351	0 05846380	AX-87601699	98038572	98060450	0
ssa13	98068175	LOC106568351	0 05846380	AX-87245072	98038572	98060450	-7726
ssa13	98142763	LOC106568352	0 05830941	AX-87508806	98120403	98150650	0
ssa13	98249437	LOC106568353	0 05830941	AX-87194067	98203985	98277768	0
ssa13	98333294	LOC106568357	0 05830941	AX-87753416	98331972	98334413	0
ssa13	98797376	epb41l4a	0 05668096	AX-87381232	98667641	98797981	0
ssa13	98797376	apc	0 05668096	AX-87381232	98791168	98876633	0
ssa13	98808337	apc	0 05668096	AX-87366205	98791168	98876633	0
ssa13	98808530	apc	0 05668096	AX-87657086	98791168	98876633	0
ssa13	98809283	apc	0 05668096	AX-87740466	98791168	98876633	0
ssa13	98824445	apc	0 05668096	AX-86982910	98791168	98876633	0
ssa13	98824654	apc	0 05852717	AX-87376732	98791168	98876633	0
ssa13	98839194	apc	0 05668096	AX-87097899	98791168	98876633	0
ssa13	98842590	apc	0 05668096	AX-87344784	98791168	98876633	0
ssa13	98853602	apc	0 05668096	AX-87434444	98791168	98876633	0
ssa13	98864138	apc	0 05668096	AX-87837671	98791168	98876633	0
ssa13	98864527	apc	0 05809917	AX-87365045	98791168	98876633	0
ssa13	98865459	apc	0 05668096	AX-87287134	98791168	98876633	0
ssa13	98872990	apc	0 05668096	AX-87289751	98791168	98876633	0
ssa13	98874952	apc	0 06082288	AX-87412448	98791168	98876633	0
ssa13	98932842	LOC106568370	0 05680172	AX-87160870	98932519	98933579	0
ssa13	98955521	LOC106568373	0 05947300	AX-87017244	98961444	98963443	5923
ssa13	98961488	LOC106568373	0 05947300	AX-87458534	98961444	98963443	0
ssa13	99030917	LOC106568374	0 06013062	AX-87783641	99070745	99182858	39828
ssa13	99327868	cntnap3	0 05861581	AX-86967452	99224292	99411154	0
ssa13	99354663	cntnap3	0 06318556	AX-87293224	99224292	99411154	0
ssa13	99415693	cntnap3	0 07223602	AX-87142875	99224292	99411154	-4540
ssa13	99563134	LOC106568414	0 06688212	AX-87718814	99564297	99575697	1163
ssa13	99613809	tdgf1	0 07389370	AX-87560135	99633517	99636523	19708
ssa13	99644764	tdgf1	0 05582947	AX-87140692	99633517	99636523	-8242
ssa13	101606537	LOC106568423	0 06257085	AX-87024158	101595199	101609297	0
ssa13	101718979	LOC106568424	0 05825740	AX-87282872	101756306	101798611	37327
ssa13	101937891	LOC106568426	0 06906472	AX-87553511	101915644	101942223	0

ssa13	101938565	LOC106568426	0 07356249	AX-87698585	101915644	101942223	0
ssa13	101949700	LOC106568427	0 08573573	AX-87040994	101949978	101951154	278
ssa13	102033446	LOC106568448	0 09044729	AX-87044352	102030393	102034660	0
ssa13	105330593	LOC106568517	0 05565644	AX-87221758	105177077	105350600	0
ssa13	105330992	LOC106568517	0 05992901	AX-87449698	105177077	105350600	0
ssa13	105492558	LOC106568497	0 06756628	AX-87516843	105414274	105514652	0
ssa13	105492852	LOC106568497	0 06756628	AX-87201370	105414274	105514652	0
ssa13	105499704	LOC106568497	0 06460455	AX-87171994	105414274	105514652	0
ssa13	105501443	LOC106568497	0 06460455	AX-87237885	105414274	105514652	0
ssa13	105509824	LOC106568497	0 06460455	AX-87086158	105414274	105514652	0
ssa14	4838415	pex5lb	0 06055771	AX-87746042	4787305	4957377	0
ssa14	4849481	pex5lb	0 05993296	AX-87412679	4787305	4957377	0
ssa14	4999615	phb2a	0 07568368	AX-87068722	5014002	5040029	14387
ssa14	5000344	phb2a	0 07568368	AX-87510589	5014002	5040029	13658
ssa14	16646653	LOC106568870	0 05696605	AX-87111489	16618962	16655753	0
ssa14	16717702	LOC106568865	0 06402755	AX-87301231	16712361	16721790	0
ssa14	18369728	LOC106568846	0 05867450	AX-87038969	18320623	18356132	-13597
ssa14	18439463	LOC106568843	0 05710652	AX-87377789	18438362	18482438	0
ssa14	18440853	LOC106568843	0 06566156	AX-87389077	18438362	18482438	0
ssa14	18618308	LOC106568841	0 06578252	AX-87157966	18656305	18896298	37997
ssa14	18655911	LOC106568841	0 06578252	AX-87107823	18656305	18896298	394
ssa14	18751317	LOC106568841	0 06070439	AX-87802637	18656305	18896298	0
ssa14	18761296	LOC106568841	0 06242605	AX-87399139	18656305	18896298	0
ssa14	18761638	LOC106568841	0 05857367	AX-87198674	18656305	18896298	0
ssa14	19152099	LOC106568835	0 07292388	AX-86953157	19091656	19154305	0
ssa14	19152104	LOC106568835	0 09035133	AX-87650027	19091656	19154305	0
ssa14	19153832	LOC106568835	0 07292388	AX-87431049	19091656	19154305	0
ssa14	19649139	LOC106568826	0 06882941	AX-87066470	19642813	19670873	0
ssa14	27080302	LOC106569192	0 07637288	AX-87585846	27035355	27173675	0
ssa14	27251295	LOC106569189	0 05724984	AX-87775298	27226860	27276762	0
ssa14	27272368	LOC106569189	0 05621211	AX-87815263	27226860	27276762	0
ssa14	39281555	LOC106569551	0 06651577	AX-87838973	39272092	39281113	-443
ssa14	43638943	nadkb	0 06722969	AX-87517504	43629177	43644527	0
ssa14	66474480	LOC106569902	0 05701817	AX-87011009	66456406	66481672	0
ssa14	66474522	LOC106569902	0 05701817	AX-87796337	66456406	66481672	0
ssa14	66768639	LOC106569891	0 06418807	AX-87698996	66746493	66769105	0
ssa14	66772766	LOC106569893	0 06418807	AX-87306967	66772620	66783454	0
ssa14	66780832	LOC106569893	0 06418807	AX-87037974	66772620	66783454	0
ssa14	66781565	LOC106569893	0 06418807	AX-87638408	66772620	66783454	0
ssa14	66781617	LOC106569893	0 06418807	AX-87157742	66772620	66783454	0
ssa14	66783278	LOC106569893	0 06418807	AX-87153025	66772620	66783454	0
ssa14	66783950	LOC106569893	0 06418807	AX-87521233	66772620	66783454	-497

ssa14	66785286	LOC106569890	0	06418807	AX-87773118	66784744	66801969	0
ssa14	66785349	LOC106569890	0	06418807	AX-87413790	66784744	66801969	0
ssa14	67109033	LOC106569883	0	05608522	AX-87718434	67103980	67139856	0
ssa14	67253836	LOC106570157	0	05745636	AX-87439829	67252438	67257441	0
ssa14	74808367	LOC106570299	0	05617289	AX-87058040	74792884	74824715	0
ssa14	76045123	LOC106570311	0	05619785	AX-87576291	75973641	76023603	-21521
ssa14	76404682	LOC106570312	0	06446370	AX-87771698	76363863	76416769	0
ssa14	76455110	LOC106570313	0	06202202	AX-87285041	76425466	76620581	0
ssa14	81732789	slc39a4	0	06359636	AX-87643476	81743572	81785996	10783
ssa15	2484370	LOC106570834	0	05769005	AX-87841888	2479986	2493585	0
ssa15	7320541	ndufs4	0	06533685	AX-86979962	7362192	7405876	41651
ssa15	8976569	npr3	0	05582044	AX-87672107	8917664	8986105	0
ssa15	11620837	kank1a	0	07031162	AX-86967499	11468519	11673796	0
ssa15	12346253	smarca2	0	06638656	AX-87764098	12356012	12506428	9759
ssa15	14475895	tbx18	0	05572361	AX-87646080	14465921	14491494	0
ssa15	18269748	sox7	0	06380500	AX-87757628	18310381	18314650	40633
ssa15	18421381	LOC106571018	0	05856284	AX-87247937	18374735	18405197	-16185
ssa15	18772314	LOC106571014	0	06218593	AX-87511174	18763169	18785405	0
ssa15	18816472	LOC106571015	0	07130453	AX-87660800	18789981	18854330	0
ssa15	39682722	LOC106571409	0	05595203	AX-87454333	39685982	39699066	3260
ssa15	39784285	LOC100194672	0	06216560	AX-87624187	39760356	39798345	0
ssa15	40074103	LOC106571412	0	06697748	AX-87246083	40083551	40098617	9448
ssa15	40104843	LOC106571414	0	06627484	AX-87061707	40105148	40154805	305
ssa15	40126942	LOC106571414	0	06182475	AX-87257805	40105148	40154805	0
ssa15	40128504	LOC106571414	0	06627484	AX-87720779	40105148	40154805	0
ssa15	40291040	hivep2a	0	06154219	AX-87133455	40274226	40357885	0
ssa15	40845819	LOC106571433	0	05756823	AX-87777928	40830557	40889355	0
ssa15	40862149	LOC106571433	0	05756823	AX-87047769	40830557	40889355	0
ssa15	40888352	LOC106571433	0	07296570	AX-87440133	40830557	40889355	0
ssa15	40924642	LOC106571434	0	06434288	AX-87081927	40892215	40925137	0
ssa15	44029425	LOC106571498	0	06007028	AX-87413051	43918602	44055321	0
ssa15	44032327	LOC106571498	0	05733524	AX-87419415	43918602	44055321	0
ssa15	44043635	LOC106571498	0	05635451	AX-87840432	43918602	44055321	0
ssa15	44142171	kcnh5a	0	06583406	AX-87637690	44086733	44229075	0
ssa15	44442137	ppp1r13bb	0	07806581	AX-87090476	44439646	44544458	0
ssa15	44453197	ppp1r13bb	0	07034717	AX-87278595	44439646	44544458	0
ssa15	44453624	ppp1r13bb	0	07806581	AX-87218632	44439646	44544458	0
ssa15	44530526	ppp1r13bb	0	07255688	AX-87270166	44439646	44544458	0
ssa15	44531649	ppp1r13bb	0	05899654	AX-87816598	44439646	44544458	0
ssa15	44532081	ppp1r13bb	0	07255688	AX-86969923	44439646	44544458	0
ssa15	44532603	ppp1r13bb	0	07255688	AX-87695804	44439646	44544458	0
ssa15	44533329	ppp1r13bb	0	07255688	AX-87372090	44439646	44544458	0

ssa15	44533801	ppp1r13bb	0 07255688	AX-87666149	44439646	44544458	0
ssa15	44541162	ppp1r13bb	0 07255688	AX-87527516	44439646	44544458	0
ssa15	44541415	ppp1r13bb	0 05899654	AX-87852478	44439646	44544458	0
ssa15	44544881	ppp1r13bb	0 05940861	AX-87480341	44439646	44544458	-424
ssa15	44605698	LOC106571714	0 07255688	AX-87039867	44584296	44835223	0
ssa15	44618930	LOC106571714	0 05899654	AX-87521130	44584296	44835223	0
ssa15	44630898	LOC106571714	0 05641844	AX-87563720	44584296	44835223	0
ssa15	44631467	LOC106571714	0 07255688	AX-87012086	44584296	44835223	0
ssa15	44639417	LOC106571714	0 07255688	AX-87387502	44584296	44835223	0
ssa15	44663925	LOC106571714	0 05857664	AX-87306518	44584296	44835223	0
ssa15	44668219	LOC106571714	0 05905594	AX-87761426	44584296	44835223	0
ssa15	44670219	LOC106571714	0 05857664	AX-87393708	44584296	44835223	0
ssa15	46549105	LOC106571722	0 06566182	AX-87229643	46548618	46549947	0
ssa15	46604811	ahsa1a	0 07515014	AX-87577506	46595866	46606677	0
ssa15	46604811	LOC106571687	0 07515014	AX-87577506	46602662	46605839	0
ssa15	46630383	vipas39	0 07515014	AX-87369659	46606976	46632300	0
ssa15	46643203	gp132	0 07515014	AX-87648586	46643001	46667362	0
ssa15	48091639	LOC106571666	0 06077152	AX-87782151	48012965	48118169	0
ssa15	48360581	LOC106571665	0 06965716	AX-87684606	48243360	48393937	0
ssa15	48368539	LOC106571665	0 06965716	AX-87691197	48243360	48393937	0
ssa15	48368559	LOC106571665	0 06965716	AX-87031123	48243360	48393937	0
ssa15	49237533	LOC106571644	0 06515848	AX-87053889	49200431	49219517	-18017
ssa15	49237649	LOC106571644	0 06515848	AX-87510398	49200431	49219517	-18133
ssa15	49240139	LOC106571644	0 06186467	AX-87469663	49200431	49219517	-20623
ssa15	59568652	LOC106571799	0 06086994	AX-87703214	59534617	59590784	0
ssa15	65274433	LOC106571924	0 06924817	AX-87323707	65017053	65414757	0
ssa15	65294736	LOC106571924	0 06903452	AX-87158053	65017053	65414757	0
ssa15	70885315	acot7	0 07138664	AX-87407306	70838449	70907494	0
ssa15	70908802	acot7	0 07275860	AX-86966121	70838449	70907494	-1309
ssa15	70980505	her3	0 07129441	AX-87583501	70982339	70983840	1834
ssa15	71039418	tp73	0 07573508	AX-87241039	71036515	71084134	0
ssa15	71065283	tp73	0 06798177	AX-87693870	71036515	71084134	0
ssa15	71067518	tp73	0 06798177	AX-87513316	71036515	71084134	0
ssa15	76004399	LOC106572115	0 05566291	AX-87835693	76029299	76049410	24900
ssa15	77718906	LOC106572071	0 05750242	AX-87532300	77701920	77763282	0
ssa15	77763134	LOC106572071	0 05773001	AX-87034020	77701920	77763282	0
ssa15	77793289	LOC106572071	0 05773001	AX-87703539	77701920	77763282	-30008
ssa15	78429557	chl1a	0 07605395	AX-87636226	78472163	78582372	42606
ssa15	85568790	LOC106572392	0 05621176	AX-87735834	85539831	86140509	0
ssa15	105838397	LOC106572946	0 06997904	AX-87443902	105782243	105985941	0
ssa15	109733211	LOC106572983	0 06243303	AX-87756354	109694197	109732755	-457
ssa16	4962195	LOC106573137	0 05871161	AX-87064972	4941368	4974697	0

ssa16	5158080	LOC106573085	0 06841726	AX-87798775	5129241	5256388	0
ssa16	5206794	LOC106573085	0 06505910	AX-87708934	5129241	5256388	0
ssa16	5208014	LOC106573085	0 06505910	AX-87808663	5129241	5256388	0
ssa16	11949227	LOC106573227	0 06361639	AX-87324709	11916888	11960574	0
ssa16	14576772	tmtc2b	0 05695168	AX-86936762	14474132	14650154	0
ssa16	18468902	fbxo31	0 05759323	AX-87302277	18462404	18491078	0
ssa16	18538314	LOC106573322	0 05653050	AX-87581241	18533272	18550774	0
ssa16	29998608	mef2aa	0 05567798	AX-87785306	29869598	30001024	0
ssa16	29998654	mef2aa	0 05567798	AX-86951071	29869598	30001024	0
ssa16	46917959	LOC106574038	0 06244481	AX-87588653	46917464	46992364	0
ssa16	46996209	LOC106574037	0 06383755	AX-86990694	46993991	47019448	0
ssa16	47001143	LOC106574037	0 05849829	AX-87174630	46993991	47019448	0
ssa16	47111415	LOC106574032	0 05849829	AX-87599115	47111111	47118386	0
ssa16	47131413	LOC106574031	0 05849829	AX-87257202	47125656	47156809	0
ssa16	47260550	LOC106574027	0 05686827	AX-87177120	47289615	47292601	29065
ssa16	47304850	mob3c	0 05745847	AX-87815934	47304582	47319853	0
ssa16	47470169	LOC106574022	0 06202812	AX-87303563	47483208	47516302	13039
ssa16	47493784	LOC106574022	0 06906942	AX-87018068	47483208	47516302	0
ssa16	71271259	LOC106574551	0 07300510	AX-87531671	71248851	71271683	0
ssa16	77989654	LOC106574775	0 06324158	AX-87526525	77966476	77999280	0
ssa16	77991573	LOC106574775	0 06512615	AX-87238876	77966476	77999280	0
ssa16	78011032	LOC106574775	0 06505519	AX-87625609	77966476	77999280	-11753
ssa16	78023798	LOC106574795	0 06615146	AX-86931212	78027995	78062801	4197
ssa16	78144987	LOC106574692	0 06083635	AX-87329589	78142378	78205505	0
ssa16	78933231	LOC106593628	0 06385756	AX-87212018	78918235	78946723	0
ssa16	79013819	tuba8l2	0 06140354	AX-86915803	79013538	79020838	0
ssa16	79889707	LOC106574967	0 07218252	AX-87201191	79889140	79892522	0
ssa16	79893932	LOC106574967	0 07150908	AX-87722060	79889140	79892522	-1411
ssa17	7988490	LOC106575121	0 07661658	AX-87342586	8012251	8018056	23761
ssa17	8636052	LOC106575116	0 05723775	AX-87125368	8621123	8646848	0
ssa17	9471211	LOC100380703	0 06657321	AX-87415044	9468325	9479977	0
ssa17	9497351	fastkd2	0 06098609	AX-87241829	9488709	9497653	0
ssa17	22947138	LOC123728174	0 07235646	AX-86929882	22965019	22968119	17881
ssa17	23009710	si:ch211-132g1.3	0 06882960	AX-87849594	22999251	23009623	-88
ssa17	23066180	LOC106575572	0 06407061	AX-87057990	23058588	23069227	0
ssa17	23093812	LOC106575576	0 06643183	AX-86972909	23096313	23098296	2501
ssa17	25147767	LOC106575569	0 07638675	AX-87608765	24742105	25154688	0
ssa17	25154377	LOC106575569	0 06544209	AX-87649335	24742105	25154688	0
ssa17	34530782	LOC106575911	0 05774149	AX-87029458	34530683	34540172	0
ssa17	34590772	LOC106595652	0 05774149	AX-87770487	34572790	34592286	0
ssa17	35901524	LOC106592258	0 06005074	AX-87015031	35499113	36009286	0
ssa17	57550198	LOC106576203	0 05674860	AX-87317739	57544354	57551928	0

ssa17	57551586	LOC106576203	0	05674860	AX-87074990	57544354	57551928	0
ssa17	61995528	itfg2	0	06973684	AX-87354473	61995851	62007657	323
ssa17	62050179	LOC106576288	0	07207811	AX-87333973	62051163	62054766	984
ssa17	62088284	LOC106576292	0	07415821	AX-87857058	62085473	62089787	0
ssa17	62172384	LOC106576297	0	06352855	AX-87315554	62166618	62180590	0
ssa17	62213625	LOC106576298	0	07320354	AX-87217597	62214845	62272305	1220
ssa17	62249550	LOC106576298	0	07198534	AX-87106287	62214845	62272305	0
ssa17	62249550	LOC123728237	0	07198534	AX-87106287	62246760	62278788	0
ssa17	62333177	LOC106576301	0	07821037	AX-87031182	62317161	62339917	0
ssa17	62360607	LOC106576335	0	07704527	AX-87280412	62349959	62371998	0
ssa17	62374590	LOC106576302	0	08275876	AX-87645248	62372624	62383281	0
ssa17	62555362	LOC106576340	0	08688635	AX-87456991	62555128	62556966	0
ssa17	62572922	LOC106576307	0	08546023	AX-87535229	62565306	62577084	0
ssa17	62597991	LOC106576310	0	07992257	AX-87777876	62596523	62599297	0
ssa17	62635100	LOC106576312	0	07086895	AX-87247606	62635312	62703267	212
ssa17	62654253	LOC106576312	0	09001550	AX-87539274	62635312	62703267	0
ssa17	62663885	LOC106576312	0	09138436	AX-87575469	62635312	62703267	0
ssa17	62665299	LOC106576312	0	08170680	AX-87721847	62635312	62703267	0
ssa17	62671216	LOC106576312	0	08800309	AX-87166092	62635312	62703267	0
ssa17	62673229	LOC106576312	0	09138436	AX-87684149	62635312	62703267	0
ssa17	62718494	LOC106576342	0	07782788	AX-87281009	62712812	62723371	0
ssa17	62755412	LOC106576315	0	08607151	AX-87345263	62756670	62762037	1258
ssa17	62760261	LOC106576315	0	09624108	AX-87790833	62756670	62762037	0
ssa17	62767809	pawr	0	09042655	AX-87647365	62762127	62842892	0
ssa17	62769627	pawr	0	09293213	AX-87071394	62762127	62842892	0
ssa17	62780132	pawr	0	06224722	AX-87132262	62762127	62842892	0
ssa17	62788321	pawr	0	09293213	AX-87073752	62762127	62842892	0
ssa17	62804190	pawr	0	09411487	AX-87102311	62762127	62842892	0
ssa17	62823885	pawr	0	09255701	AX-86969674	62762127	62842892	0
ssa17	62838246	pawr	0	10212647	AX-87326519	62762127	62842892	0
ssa17	62875414	LOC106576317	0	05683849	AX-87457857	62869290	63085325	0
ssa17	62876176	LOC106576317	0	08205656	AX-86964385	62869290	63085325	0
ssa17	62876713	LOC106576317	0	05683849	AX-87174990	62869290	63085325	0
ssa17	62903210	LOC106576317	0	05950938	AX-87015957	62869290	63085325	0
ssa17	62913460	LOC106576317	0	05935388	AX-87192521	62869290	63085325	0
ssa17	62918300	LOC106576317	0	06891689	AX-87794847	62869290	63085325	0
ssa17	62919028	LOC106576317	0	05952814	AX-87527239	62869290	63085325	0
ssa17	62931849	LOC106576317	0	06766979	AX-87068844	62869290	63085325	0
ssa17	62936793	LOC106576317	0	06893333	AX-87777621	62869290	63085325	0
ssa17	62943816	LOC106576317	0	06893333	AX-87440666	62869290	63085325	0
ssa17	62947430	LOC106576317	0	06467817	AX-87033980	62869290	63085325	0
ssa17	62965726	LOC106576317	0	06602875	AX-86970592	62869290	63085325	0

ssa17	62974553	LOC106576317	0	08146176	AX-87304249	62869290	63085325	0
ssa17	62981822	LOC106576317	0	07049284	AX-87341308	62869290	63085325	0
ssa17	63030934	LOC106576317	0	08119538	AX-87342747	62869290	63085325	0
ssa17	63104795	LOC106576319	0	06288744	AX-87291201	63098256	63105694	0
ssa17	63105853	LOC106576319	0	06288744	AX-87697155	63098256	63105694	-160
ssa17	63113481	LOC106576320	0	05804007	AX-86939239	63107853	63142545	0
ssa17	63117921	LOC106576320	0	06741113	AX-87574576	63107853	63142545	0
ssa17	63119203	LOC106576320	0	06741113	AX-87165272	63107853	63142545	0
ssa17	63152953	LOC106576320	0	06894043	AX-87343628	63107853	63142545	-10409
ssa17	63158288	LOC106576320	0	06425250	AX-87501492	63107853	63142545	-15744
ssa17	63159412	LOC106576320	0	06711376	AX-86921406	63107853	63142545	-16868
ssa17	63173612	LOC106576349	0	08386171	AX-87491695	63203968	63206484	30356
ssa17	63176313	LOC106576349	0	07348044	AX-86932814	63203968	63206484	27655
ssa17	63181263	LOC106576349	0	07348044	AX-87002103	63203968	63206484	22705
ssa17	63244433	LOC106576322	0	09543305	AX-87201550	63248307	63249674	3874
ssa17	63280073	LOC123728101	0	09714257	AX-87469839	63276727	63277673	-2401
ssa17	63294127	myf6	0	09490739	AX-87380026	63301081	63302460	6954
ssa17	63296410	myf6	0	06194347	AX-86928553	63301081	63302460	4671
ssa17	63308533	LOC106576326	0	06014695	AX-87659118	63309003	63311670	470
ssa17	63309209	LOC106576326	0	06175684	AX-87675919	63309003	63311670	0
ssa17	63309725	LOC106576326	0	05689056	AX-87657857	63309003	63311670	0
ssa17	63310928	LOC106576326	0	06014695	AX-87230325	63309003	63311670	0
ssa17	63320556	lin7a	0	08223514	AX-87310962	63321934	63353642	1378
ssa17	63348191	lin7a	0	08887876	AX-87474429	63321934	63353642	0
ssa17	63360608	LOC106576328	0	09384827	AX-87765141	63361867	63399762	1259
ssa17	63362481	LOC106576328	0	09384827	AX-86921378	63361867	63399762	0
ssa17	63366611	LOC106576328	0	09114818	AX-87053532	63361867	63399762	0
ssa17	63369572	LOC106576328	0	07621463	AX-87535236	63361867	63399762	0
ssa17	63406265	ppfia2	0	08617038	AX-87766535	63404548	63644751	0
ssa17	63406597	ppfia2	0	09208314	AX-87210698	63404548	63644751	0
ssa17	63408223	ppfia2	0	05902127	AX-87061742	63404548	63644751	0
ssa17	63409106	ppfia2	0	05579283	AX-87812534	63404548	63644751	0
ssa17	63421838	ppfia2	0	09294469	AX-87503083	63404548	63644751	0
ssa17	63422221	ppfia2	0	09290321	AX-87855610	63404548	63644751	0
ssa17	63431661	ppfia2	0	08266297	AX-87487176	63404548	63644751	0
ssa17	63431773	ppfia2	0	09067987	AX-87801349	63404548	63644751	0
ssa17	63607991	ppfia2	0	05919620	AX-87861323	63404548	63644751	0
ssa17	64713529	LOC106576423	0	05811671	AX-87481925	64678506	64754067	0
ssa17	64770491	ptn	0	05699591	AX-87680501	64759008	64811318	0
ssa17	64857585	tulp3	0	07256353	AX-87633331	64844349	64883652	0
ssa17	64865398	tulp3	0	07424841	AX-87437625	64844349	64883652	0
ssa17	64994038	LOC106576417	0	06611064	AX-86951670	64988932	64998794	0

ssa17	64995445	LOC106576417	0 06611064	AX-87412587	64988932	64998794	0
ssa17	64998928	LOC106576417	0 06611064	AX-86937012	64988932	64998794	-135
ssa17	65037768	LOC106576413	0 06378929	AX-87512605	65030090	65057129	0
ssa17	66019566	LOC106576369	0 05559280	AX-87783366	66013018	66072573	0
ssa17	66692224	LOC106576453	0 05639736	AX-87665299	66682024	66705398	0
ssa17	66985312	LOC106576460	0 06144495	AX-87346457	66960895	66969354	-15959
ssa17	67099592	LOC106576502	0 06144495	AX-87753709	67112007	67131195	12415
ssa17	67192092	imdh1	0 05665505	AX-87206512	67198214	67247856	6122
ssa17	67194858	imdh1	0 05665505	AX-87728403	67198214	67247856	3356
ssa17	67197037	imdh1	0 05665505	AX-87176536	67198214	67247856	1177
ssa17	67199324	imdh1	0 05665505	AX-87028022	67198214	67247856	0
ssa17	73392282	LOC106576619	0 06642307	AX-87632824	73384399	73394020	0
ssa18	8291828	LOC106576807	0 05949457	AX-87469348	8310447	8336736	18619
ssa18	8295334	LOC106576807	0 06982511	AX-87348045	8310447	8336736	15113
ssa18	8370445	polr1b	0 05633583	AX-87177559	8357332	8373033	0
ssa18	8371489	polr1b	0 05906100	AX-87144745	8357332	8373033	0
ssa18	8378648	polr1b	0 05907626	AX-87513925	8357332	8373033	-5616
ssa18	8408835	polr1b	0 06583511	AX-87362511	8357332	8373033	-35803
ssa18	8409305	polr1b	0 05979910	AX-87442373	8357332	8373033	-36273
ssa18	8429166	LOC106576803	0 06614388	AX-87496745	8475300	8479568	46134
ssa18	8431373	LOC106576803	0 06643408	AX-87801468	8475300	8479568	43927
ssa18	8473861	LOC106576803	0 05708546	AX-87624609	8475300	8479568	1439
ssa18	10462970	LOC106576776	0 06720796	AX-87366934	10348011	10628892	0
ssa18	11340315	LOC106576760	0 06052709	AX-87839136	11269443	11312421	-27895
ssa18	12577395	LOC106576737	0 05652033	AX-87717971	12579916	12669836	2521
ssa18	16498260	ndst2a	0 05861173	AX-86990053	16352007	16515248	0
ssa18	54545291	LOC106577489	0 06683391	AX-87532533	54460968	54546187	0
ssa18	58277180	LOC123728729	0 05783163	AX-87409448	58275980	58278113	0
ssa18	58285997	LOC106577558	0 06239535	AX-87293805	58277213	58289373	0
ssa18	58316536	LOC106577559	0 06239535	AX-87057817	58310190	58317787	0
ssa18	58363517	LOC106577563	0 05612339	AX-86911236	58362800	58390732	0
ssa18	66817165	LOC106577804	0 05639981	AX-87463742	66830709	66844005	13544
ssa18	73845793	trnal-aag	0 06175409	AX-87715553	73845959	73846040	166
ssa18	76673880	LOC106578039	0 05971560	AX-87458909	76510823	76801315	0
ssa19	8685090	tpk1	0 06328730	AX-87553952	8652776	8827641	0
ssa19	8685417	tpk1	0 05755539	AX-87217141	8652776	8827641	0
ssa19	8717659	tpk1	0 06164533	AX-86998864	8652776	8827641	0
ssa19	8721706	tpk1	0 06177389	AX-87855028	8652776	8827641	0
ssa19	8883973	tnfrsf11a	0 06619187	AX-87055906	8881629	8902270	0
ssa19	15257328	trdmt1	0 05894919	AX-87353397	15241938	15261546	0
ssa19	17683792	LOC106578541	0 06315748	AX-87014088	17245761	17812146	0
ssa19	17683906	LOC106578541	0 06240035	AX-87424187	17245761	17812146	0

ssa19	17699739	LOC106578541	0 07359731	AX-87663608	17245761	17812146	0
ssa19	17722953	LOC106578541	0 05581232	AX-87263500	17245761	17812146	0
ssa19	17759951	LOC106578541	0 07279109	AX-86925104	17245761	17812146	0
ssa19	17776986	LOC106578541	0 05943853	AX-87067895	17245761	17812146	0
ssa19	19333307	svila	0 06088769	AX-87668386	19326439	19465435	0
ssa19	19393995	svila	0 06994819	AX-87525240	19326439	19465435	0
ssa19	19400640	svila	0 06783416	AX-87339365	19326439	19465435	0
ssa19	19403799	svila	0 07390802	AX-87021705	19326439	19465435	0
ssa19	31426316	LOC106578728	0 05663145	AX-87263596	31412893	31428954	0
ssa19	33051997	LOC106578765	0 05574932	AX-87195825	32896140	33460660	0
ssa19	53225974	LOC106579029	0 06787757	AX-87835532	53226464	53239893	490
ssa19	64452935	sult1st6	0 05677746	AX-87415702	64393521	64456640	0
ssa19	80579812	LOC106579695	0 07055517	AX-87493594	80568515	80574538	-5275
ssa19	80589702	LOC106579687	0 07020342	AX-87129066	80588157	80723148	0
ssa19	80601908	LOC106579687	0 05835638	AX-87739741	80588157	80723148	0
ssa19	80621159	LOC106579687	0 08208057	AX-87048077	80588157	80723148	0
ssa19	80850450	LOC106597323	0 05804070	AX-87614990	80832750	80850847	0
ssa20	10632534	LOC106580142	0 06308045	AX-87157881	10632200	10650098	0
ssa20	11005863	LOC106580141	0 05795637	AX-87046277	10816104	11120375	0
ssa20	11064697	LOC106580141	0 06286645	AX-87740069	10816104	11120375	0
ssa20	14487371	fam222a	0 05699828	AX-87722764	14491924	14614393	4553
ssa20	14499687	fam222a	0 05670330	AX-87016786	14491924	14614393	0
ssa20	21724088	LOC106579914	0 08251232	AX-87136235	21686355	21732667	0
ssa20	33769116	LOC106580487	0 06810493	AX-87468110	33625442	33998797	0
ssa20	33779287	LOC106580487	0 06810493	AX-87488203	33625442	33998797	0
ssa20	33794028	LOC106580487	0 06123156	AX-86933595	33625442	33998797	0
ssa20	33795135	LOC106580487	0 06586817	AX-87626085	33625442	33998797	0
ssa20	33803881	LOC106580487	0 06672301	AX-87659994	33625442	33998797	0
ssa20	33808638	LOC106580487	0 06672301	AX-87847804	33625442	33998797	0
ssa20	34078407	LOC106580542	0 06311846	AX-87573310	34025408	34065907	-12501
ssa20	34079340	LOC106580542	0 06311846	AX-87628771	34025408	34065907	-13434
ssa20	34083290	LOC106580542	0 06311846	AX-87834464	34025408	34065907	-17384
ssa20	73251077	LOC106581302	0 06826806	AX-87659310	73062302	73308451	0
ssa20	76001297	LOC106581285	0 05989059	AX-87038651	76000718	76002221	0
ssa20	86692849	LOC106581476	0 06451255	AX-86956610	86574798	86729512	0
ssa20	91752224	cog6	0 06067735	AX-87064572	91636074	91755191	0
ssa20	91832716	LOC106581552	0 06234309	AX-87685831	91760809	91921462	0
ssa21	1301137	tbc1d4	0 06303554	AX-87812432	1200802	1361383	0
ssa21	1942689	klf12b	0 05930255	AX-87373854	1714606	1946645	0
ssa21	1993513	LOC106581629	0 05930255	AX-87404852	1984898	1995244	0
ssa21	2012691	LOC106581629	0 05930255	AX-87307803	1984898	1995244	-17448
ssa21	20892516	igfbp-2b2	0 06178629	AX-87136356	20875213	20988900	0

ssa21	20892774	igfbp-2b2	0 06424725	AX-87097865	20875213	20988900	0
ssa21	21099631	LOC106581928	0 05791142	AX-87011568	21016986	21139105	0
ssa21	21328615	gulp1a	0 05924169	AX-87082008	21243411	21410784	0
ssa21	21335252	gulp1a	0 05971135	AX-87223540	21243411	21410784	0
ssa21	21432928	gulp1a	0 05852178	AX-86949286	21243411	21410784	-22145
ssa21	21488222	LOC106581889	0 05848921	AX-87029696	21487644	21489104	0
ssa21	31103491	LOC106582057	0 05868521	AX-87634297	31097001	31107225	0
ssa21	31140883	LOC106582033	0 06864008	AX-87821910	31119078	31142135	0
ssa21	31181055	LOC106582055	0 07043041	AX-87333231	31180816	31186233	0
ssa21	31182363	LOC106582055	0 06788165	AX-87732617	31180816	31186233	0
ssa21	31231069	LOC106582055	0 05735261	AX-87007802	31180816	31186233	-44837
ssa21	32233270	LOC106582185	0 05716669	AX-87465244	32212012	32237068	0
ssa21	32737670	LOC100380296	0 06299751	AX-87506301	32657840	32740910	0
ssa21	32737924	LOC100380296	0 06299751	AX-87492197	32657840	32740910	0
ssa21	32739741	LOC100380296	0 06299751	AX-87379923	32657840	32740910	0
ssa21	32742327	LOC100380296	0 06646247	AX-87132098	32657840	32740910	-1418
ssa21	32742753	LOC100380296	0 06299751	AX-87682623	32657840	32740910	-1844
ssa21	32996589	LOC106582170	0 05842964	AX-87040972	33002536	33013119	5947
ssa21	33041497	LOC106582223	0 06127851	AX-87212652	33031883	33051359	0
ssa21	37436157	LOC123729570	0 05550372	AX-87353700	37348828	37623889	0
ssa21	39783813	LOC106582303	0 06025139	AX-87357390	39783523	39795504	0
ssa21	39832551	LOC106582303	0 06025139	AX-87037986	39783523	39795504	-37048
ssa21	40217682	LOC106582360	0 06871742	AX-87446378	39964984	40207791	-9892
ssa21	40217831	LOC106582360	0 07125277	AX-87828697	39964984	40207791	-10041
ssa21	40231291	tfcp2l1	0 06871742	AX-87665735	40243822	40253207	12531
ssa21	40253189	tfcp2l1	0 05718802	AX-87811610	40243822	40253207	0
ssa21	40308090	LOC106582358	0 06393190	AX-87467488	40256985	40402390	0
ssa21	40322481	LOC106582358	0 05962799	AX-87198501	40256985	40402390	0
ssa21	40326562	LOC106582358	0 06278423	AX-87176419	40256985	40402390	0
ssa21	40598504	LOC106582356	0 06278685	AX-87216463	40577559	40585832	-12673
ssa21	40635914	hspbap1	0 06400228	AX-87651880	40632339	40680632	0
ssa21	40681877	slc49a4	0 06296928	AX-87145601	40680673	40751648	0
ssa21	41142406	pdia5	0 07127668	AX-87729102	41097682	41156897	0
ssa21	41156387	pdia5	0 06945482	AX-87486164	41097682	41156897	0
ssa21	48046515	ahr2a	0 05746995	AX-87864119	48001819	48073703	0
ssa21	48516153	LOC106582442	0 05778559	AX-86914614	48527708	48530808	11555
ssa21	48580757	LOC106582425	0 05816358	AX-87649112	48618951	48639542	38194
ssa21	48647735	LOC106582425	0 05590268	AX-87280908	48618951	48639542	-8194
ssa21	48649168	LOC106582425	0 05590268	AX-86944447	48618951	48639542	-9627
ssa21	48668495	LOC106582444	0 05590268	AX-87119245	48675881	48688753	7386
ssa21	48706999	LOC106582430	0 05961317	AX-87421005	48704914	48708885	0
ssa21	48816499	LOC106582445	0 05613592	AX-87608125	48823136	48978699	6637

ssa21	48952639	LOC106582445	0	06472201	AX-87266854	48823136	48978699	0
ssa21	49005216	LOC106582447	0	07425242	AX-87140283	48991994	49040193	0
ssa21	49031893	LOC106582447	0	06407743	AX-87345620	48991994	49040193	0
ssa21	49042718	LOC106582448	0	06277015	AX-87502626	49040744	49067178	0
ssa21	49988688	LOC106582455	0	06719950	AX-87517449	49728061	49996463	0
ssa21	50999244	atp5g2	0	07350777	AX-87697613	51041210	51043589	41966
ssa21	51026674	atp5g2	0	08453751	AX-87583269	51041210	51043589	14536
ssa21	51027235	atp5g2	0	09616303	AX-87259633	51041210	51043589	13975
ssa21	51030134	atp5g2	0	08453751	AX-87540956	51041210	51043589	11076
ssa21	51030368	atp5g2	0	08557957	AX-87476193	51041210	51043589	10842
ssa21	51031416	atp5g2	0	08194380	AX-87392234	51041210	51043589	9794
ssa21	51034998	atp5g2	0	08458845	AX-87435105	51041210	51043589	6212
ssa21	51049046	LOC106582491	0	05544987	AX-86922730	51044546	51079803	0
ssa21	51080347	LOC106582491	0	08290057	AX-86913339	51044546	51079803	-545
ssa21	51199007	LOC106582477	0	06170022	AX-87382418	51197989	51199033	0
ssa21	51885760	LOC106582484	0	08061261	AX-87111669	51883820	51915422	0
ssa21	51896441	LOC106582484	0	08061261	AX-87473304	51883820	51915422	0
ssa21	51996917	LOC106582485	0	06186443	AX-87113618	51987086	51996585	-333
ssa22	11646910	LOC106582605	0	06509678	AX-87715363	11629489	11996527	0
ssa22	11647110	LOC106582605	0	06509678	AX-87767212	11629489	11996527	0
ssa22	12660305	fam83e	0	05988947	AX-87016849	12657565	12665303	0
ssa22	12660545	fam83e	0	05988947	AX-87481622	12657565	12665303	0
ssa22	12661513	fam83e	0	05988947	AX-87691934	12657565	12665303	0
ssa22	12661840	fam83e	0	05988947	AX-87696094	12657565	12665303	0
ssa22	34284353	LOC106583278	0	05779655	AX-87607926	34283011	34287683	0
ssa22	34464430	LOC106583274	0	05548355	AX-87347183	34444861	34476117	0
ssa22	60140188	LOC106583800	0	06010034	AX-87798098	60092243	60149764	0
ssa22	60140397	LOC106583800	0	05784830	AX-87566503	60092243	60149764	0
ssa23	32556368	LOC106584427	0	06518049	AX-87029177	32506354	32546482	-9887
ssa23	32581642	LOC106584428	0	05647954	AX-87384374	32571131	32581505	-138
ssa23	32581861	LOC106584428	0	05844574	AX-87545790	32571131	32581505	-357
ssa23	32588771	LOC106584428	0	06188086	AX-87052157	32571131	32581505	-7267
ssa23	32590402	LOC106584428	0	06188086	AX-87345126	32571131	32581505	-8898
ssa23	32602747	atp8b3	0	06992426	AX-87093878	32602378	32635561	0
ssa23	32602847	atp8b3	0	05983681	AX-87415685	32602378	32635561	0
ssa23	33120328	LOC106584443	0	05655939	AX-87467904	33080528	33404489	0
ssa23	33309374	LOC106584443	0	06286856	AX-87527521	33080528	33404489	0
ssa23	34766548	LOC106584460	0	05555351	AX-87802901	34764066	34780797	0
ssa23	34777656	LOC106584460	0	05553355	AX-87721182	34764066	34780797	0
ssa23	39960510	LOC106584592	0	05668869	AX-87858852	39864041	39972999	0
ssa23	39968382	LOC106584592	0	05668869	AX-87109237	39864041	39972999	0
ssa24	4769475	LOC106585216	0	05793915	AX-87338675	4659536	4769802	0

ssa24	7447177	LOC106585166	0	06976036	AX-87399258	7444503	7448563	0
ssa24	7480542	LOC106585164	0	05927338	AX-87716371	7476199	7525784	0
ssa24	7623716	LOC106585162	0	06098235	AX-87688959	7596861	7624440	0
ssa24	7699304	LOC106585159	0	05654908	AX-87395552	7668703	7706124	0
ssa24	11757238	arvcfb	0	05885513	AX-86961057	11700249	11950783	0
ssa24	17194084	LOC106585259	0	06593594	AX-87115452	17128004	17306874	0
ssa24	18119444	vps33a	0	05703091	AX-87268647	18107392	18119664	0
ssa24	18143457	LOC106585294	0	05703091	AX-87845431	18120256	18174655	0
ssa24	18153706	LOC106585294	0	05703091	AX-87532380	18120256	18174655	0
ssa24	18154032	LOC106585294	0	05703091	AX-87094247	18120256	18174655	0
ssa24	18166739	LOC106585294	0	05703091	AX-87827973	18120256	18174655	0
ssa24	24851866	LOC100194534	0	05548449	AX-87430447	24849186	24851977	0
ssa24	24855260	LOC100194534	0	05560580	AX-87451754	24849186	24851977	-3284
ssa24	24861074	LOC106585473	0	05751626	AX-87800555	24860344	24878428	0
ssa24	24872489	LOC106585473	0	05655363	AX-87315273	24860344	24878428	0
ssa24	24878071	LOC106585473	0	05856576	AX-87589753	24860344	24878428	0
ssa24	24884441	LOC106585308	0	05815292	AX-87865428	24881138	24895081	0
ssa24	24886646	LOC106585308	0	05815292	AX-87200681	24881138	24895081	0
ssa24	24903630	LOC106585472	0	06137071	AX-87696173	24898379	24903156	-475
ssa24	24922576	LOC106585469	0	05822309	AX-87504339	24922318	24952055	0
ssa24	25581674	olm2a	0	05598747	AX-86991353	25568070	25582974	0
ssa24	25588427	nr6a1a	0	05763830	AX-87749789	25584559	25725848	0
ssa24	25595507	nr6a1a	0	05564644	AX-87279868	25584559	25725848	0
ssa24	25596231	nr6a1a	0	05763830	AX-87237153	25584559	25725848	0
ssa24	25627927	nr6a1a	0	06034783	AX-87836826	25584559	25725848	0
ssa24	25640311	nr6a1a	0	06557249	AX-87510053	25584559	25725848	0
ssa24	25640311	LOC123730376	0	06557249	AX-87510053	25635836	25647748	0
ssa24	25644513	nr6a1a	0	06753644	AX-87355207	25584559	25725848	0
ssa24	25644513	LOC123730376	0	06753644	AX-87355207	25635836	25647748	0
ssa24	25676346	nr6a1a	0	05915243	AX-87503288	25584559	25725848	0
ssa24	25695841	nr6a1a	0	05915243	AX-87259379	25584559	25725848	0
ssa24	25867553	adgrd2	0	05575952	AX-87524876	25810721	25830333	-37221
ssa24	25867728	adgrd2	0	05575952	AX-87141761	25810721	25830333	-37396
ssa24	26090326	LOC106585440	0	06687579	AX-87436915	26129439	26160103	39113
ssa24	26091210	LOC106585440	0	06687579	AX-86939227	26129439	26160103	38229
ssa24	32185987	LOC106585775	0	06847027	AX-87393946	32138537	32141951	-44037
ssa24	32231337	LOC106585770	0	06809688	AX-87025090	32237265	32268278	5928
ssa24	32244803	LOC106585770	0	06737405	AX-87434888	32237265	32268278	0
ssa24	32286380	LOC106585770	0	06737405	AX-87012799	32237265	32268278	-18103
ssa24	32365642	LOC106585750	0	05783433	AX-87221863	32360085	32364890	-753
ssa24	32394501	LOC106585767	0	05783433	AX-86971920	32367530	32416035	0
ssa24	38035063	hpgds	0	06098869	AX-87381242	38027281	38038200	0

ssa24	42578071	LOC106585928	0 05828048	AX-87012717	42570463	42689555	0
ssa25	5167716	LOC106586067	0 06289126	AX-87314968	5078495	5356786	0
ssa25	5311775	LOC106586067	0 07099399	AX-87733045	5078495	5356786	0
ssa25	9804512	LOC106586125	0 06399072	AX-87117276	9646370	9843200	0
ssa25	10893638	LOC106586149	0 05639370	AX-87088391	10911007	11006758	17369
ssa25	15397574	hoxd3a	0 07552797	AX-87249845	15398624	15420896	1050
ssa25	15421404	hoxd3a	0 07463395	AX-87143463	15398624	15420896	-509
ssa25	15443282	hoxd1aa	0 07106621	AX-86938818	15423576	15425073	-18210
ssa25	15462007	hoxd1aa	0 06364059	AX-87228447	15423576	15425073	-36935
ssa25	15751685	osbpl6	0 05968035	AX-87684752	15703303	15762299	0
ssa25	21437174	LOC106586374	0 06482885	AX-87012593	21432380	21460623	0
ssa25	28408767	LOC106586499	0 05598024	AX-87635993	28405743	28411812	0
ssa25	28408767	LOC106586500	0 05598024	AX-87635993	28407279	28413079	0
ssa25	28475968	LOC106586502	0 06429765	AX-87278761	28461667	28472765	-3204
ssa25	29751031	ranbp2	0 05709901	AX-87714978	29734423	29756244	0
ssa25	33678778	LOC106586637	0 06809925	AX-87240281	33688052	33701734	9274
ssa25	33709188	LOC106586638	0 06809925	AX-87126744	33705299	33726234	0
ssa25	34059911	LOC106586572	0 06658410	AX-87050089	34049192	34059874	-38
ssa25	34064601	LOC106586572	0 06658410	AX-86932510	34049192	34059874	-4728
ssa25	36935537	impg2a	0 05994296	AX-87248711	36909343	36970538	0
ssa25	37114629	LOC106586706	0 05792948	AX-87345692	37102485	37136959	0
ssa25	37130102	LOC106586706	0 05792948	AX-87005718	37102485	37136959	0
ssa25	37130368	LOC106586706	0 05792948	AX-86908609	37102485	37136959	0
ssa25	37314351	cmss1	0 05785953	AX-87299328	37300134	37383983	0
ssa25	49917807	si:dkey-69o16.5	0 05640262	AX-87717849	49902833	49919300	0
ssa26	1719898	cfap77	0 06144634	AX-87117306	1672344	1801205	0
ssa26	1736460	cfap77	0 05546659	AX-86937636	1672344	1801205	0
ssa26	1798865	cfap77	0 05883945	AX-87140144	1672344	1801205	0
ssa26	1799484	cfap77	0 05883945	AX-87717629	1672344	1801205	0
ssa26	6252734	LOC106587057	0 06564438	AX-86944773	5916821	6410687	0
ssa26	6262206	LOC106587057	0 06438274	AX-87610305	5916821	6410687	0
ssa26	6262542	LOC106587057	0 06309825	AX-87537116	5916821	6410687	0
ssa26	6262902	LOC106587057	0 07017320	AX-86911733	5916821	6410687	0
ssa26	6443385	LOC106587057	0 05593961	AX-86965644	5916821	6410687	-32699
ssa26	11713331	LOC106587167	0 05674900	AX-87764224	11751956	11964727	38625
ssa26	11719291	LOC106587167	0 05674900	AX-86938011	11751956	11964727	32665
ssa26	11750565	LOC106587167	0 05674900	AX-87130043	11751956	11964727	1391
ssa26	12655151	LOC106587166	0 05717098	AX-87661461	12466640	12655927	0
ssa26	13715175	LOC106587291	0 05774370	AX-87420585	13625861	13698834	-16342
ssa26	14563017	LOC106587288	0 06746501	AX-87542417	14513675	14581151	0
ssa26	14563485	LOC106587288	0 06727008	AX-87843427	14513675	14581151	0
ssa26	14565093	LOC106587288	0 06693531	AX-87487507	14513675	14581151	0

ssa26	14571805	LOC106587288	0 06693531	AX-87627814	14513675	14581151	0
ssa26	14571916	LOC106587288	0 06693531	AX-87001073	14513675	14581151	0
ssa26	15005454	LOC106587279	0 06986090	AX-86967916	14997750	15011788	0
ssa26	15031186	LOC106587278	0 07719380	AX-87205058	15027068	15033290	0
ssa26	15094241	LOC106587277	0 07301245	AX-87169347	15075919	15089816	-4426
ssa26	15117403	LOC106587276	0 07070332	AX-87606229	15114163	15147428	0
ssa26	15132744	LOC106587276	0 07372317	AX-87161932	15114163	15147428	0
ssa26	15133161	LOC106587276	0 07381710	AX-87263300	15114163	15147428	0
ssa26	15141798	LOC106587276	0 07771772	AX-87153199	15114163	15147428	0
ssa26	15146952	LOC106587276	0 07762481	AX-87105338	15114163	15147428	0
ssa26	15211585	LOC106587272	0 05925540	AX-87126429	15196415	15213178	0
ssa26	15781347	LOC106587256	0 06202358	AX-87558769	15685437	15832291	0
ssa26	15783012	LOC106587256	0 06320641	AX-87559669	15685437	15832291	0
ssa26	16934679	LOC106587233	0 05900421	AX-86985670	16919834	16983818	0
ssa26	16960603	LOC106587233	0 05900421	AX-87052528	16919834	16983818	0
ssa26	19185650	pkd1l2a	0 06095351	AX-87447595	19170523	19198673	0
ssa26	21501638	LOC106587411	0 05636358	AX-87243310	21499498	21505544	0
ssa26	21505156	LOC106587411	0 05882916	AX-87498837	21499498	21505544	0
ssa26	21505770	LOC106587411	0 06293170	AX-87386947	21499498	21505544	-227
ssa26	21513757	LOC106587408	0 06377523	AX-87291803	21519095	21522864	5338
ssa26	22246546	LOC106587382	0 05899915	AX-87500567	22235275	22268582	0
ssa26	36724454	LOC106594141	0 05604232	AX-87439442	36657886	36721940	-2515
ssa26	36963067	LOC123730786	0 05618730	AX-87680568	36957353	37004563	0
ssa26	40307309	LOC106562785	0 05738117	AX-87570750	40275319	40325692	0
ssa26	41746022	LOC106562707	0 08376213	AX-87463525	41701489	41846170	0
ssa26	41843735	LOC106562707	0 08772258	AX-86930650	41701489	41846170	0
ssa26	51639597	LOC106588030	0 05762310	AX-87409196	51610173	51665691	0
ssa27	13702075	LOC106588299	0 05601585	AX-87679512	13708027	13737825	5952
ssa27	13702507	LOC106588299	0 05601585	AX-87384949	13708027	13737825	5520
ssa27	13713280	LOC106588299	0 05569246	AX-86944823	13708027	13737825	0
ssa27	17912939	trappc9	0 05651981	AX-87604730	17758972	18051630	0
ssa27	17912970	trappc9	0 06011897	AX-87785213	17758972	18051630	0
ssa27	17938608	trappc9	0 06157446	AX-87828076	17758972	18051630	0
ssa27	18005170	trappc9	0 06157446	AX-86939030	17758972	18051630	0
ssa27	18042601	trappc9	0 06112390	AX-87442105	17758972	18051630	0
ssa27	18044718	trappc9	0 06932735	AX-87691715	17758972	18051630	0
ssa27	18064986	LOC106588618	0 06362147	AX-87341007	18062080	18066378	0
ssa27	18067655	LOC106588618	0 05753796	AX-87213587	18062080	18066378	-1278
ssa27	18067789	LOC106588618	0 05753796	AX-87144843	18062080	18066378	-1412
ssa27	18184424	LOC106588613	0 07884136	AX-87409747	18180990	18187021	0
ssa27	18186365	LOC106588613	0 07450863	AX-87424179	18180990	18187021	0
ssa27	18194391	LOC106588611	0 07884136	AX-86948907	18190336	18197960	0

ssa27	18197225	LOC106588611	0 07873965	AX-87317155	18190336	18197960	0
ssa27	18239893	LOC106588610	0 07700700	AX-87359335	18224502	18228686	-11208
ssa27	18250382	LOC106588635	0 07700700	AX-87779598	18270194	18405031	19812
ssa27	18255607	LOC106588635	0 08110716	AX-87668201	18270194	18405031	14587
ssa27	18283492	LOC106588635	0 05821055	AX-87797128	18270194	18405031	0
ssa27	18294154	LOC106588635	0 05824332	AX-87070758	18270194	18405031	0
ssa27	25626620	tpd52	0 06133261	AX-87602293	25625582	25652513	0
ssa27	28378391	LOC106588852	0 05575905	AX-87414384	28377774	28383755	0
ssa27	28716053	LOC106588853	0 06096826	AX-86919159	28716396	28816302	343
ssa27	28844069	fam221a	0 05768718	AX-87515496	28846941	28870244	2872
ssa27	28902809	igf2bp3	0 06867550	AX-87176564	28904355	28945408	1546
ssa27	31331514	LOC106588944	0 05581746	AX-86973770	31291823	31454446	0
ssa27	31352501	LOC106588944	0 05933717	AX-87534327	31291823	31454446	0
ssa27	31354090	LOC106588944	0 05933717	AX-87177298	31291823	31454446	0
ssa27	31363788	LOC106588944	0 05933717	AX-87589133	31291823	31454446	0
ssa27	31430810	LOC106588944	0 05798486	AX-87116801	31291823	31454446	0
ssa27	31469827	LOC106588945	0 05906905	AX-87424258	31456115	31470289	0
ssa27	32814995	LOC106588970	0 06221420	AX-87274346	32801685	32824260	0
ssa27	32912198	LOC106588968	0 05827793	AX-87380796	32875658	32931983	0
ssa27	32927699	LOC106588968	0 07298946	AX-87843095	32875658	32931983	0
ssa27	33041746	LOC106588959	0 09743734	AX-87203068	33015608	33032783	-8964
ssa27	33066989	LOC106588963	0 10446145	AX-87641867	33056622	33081118	0
ssa27	33084935	LOC100380787	0 10877832	AX-87806881	33081425	33168401	0
ssa27	33092621	LOC100380787	0 10728671	AX-87165119	33081425	33168401	0
ssa27	33122902	LOC100380787	0 07479323	AX-87496822	33081425	33168401	0
ssa27	33125609	LOC100380787	0 06847871	AX-87839173	33081425	33168401	0
ssa27	33128171	LOC100380787	0 08616437	AX-86954648	33081425	33168401	0
ssa27	33180191	LOC100380787	0 08652551	AX-87417695	33081425	33168401	-11791
ssa27	33421397	LOC123730919	0 07554133	AX-87407260	33384395	33411012	-10386
ssa27	33505561	LOC106588981	0 06714697	AX-87021652	33510405	33514469	4844
ssa27	33529218	LOC123730920	0 06194128	AX-87658101	33539985	33543077	10767
ssa27	33995758	LOC106588992	0 07572343	AX-87529929	33998354	34251134	2596
ssa27	34005657	LOC106588992	0 07572343	AX-87162849	33998354	34251134	0
ssa27	34122496	LOC106588992	0 06662574	AX-87542672	33998354	34251134	0
ssa27	34122529	LOC106588992	0 06662574	AX-87172956	33998354	34251134	0
ssa27	34123318	LOC106588992	0 06735711	AX-87695760	33998354	34251134	0
ssa27	34126134	LOC106588992	0 06735711	AX-87851904	33998354	34251134	0
ssa27	34135752	LOC106588992	0 06735711	AX-86967829	33998354	34251134	0
ssa27	34173446	LOC106588992	0 05776341	AX-87068404	33998354	34251134	0
ssa27	34204811	LOC106588992	0 06680228	AX-87222737	33998354	34251134	0
ssa27	34254659	LOC106588994	0 07213079	AX-87842009	34254201	34256999	0
ssa27	34574344	LOC106588997	0 05741127	AX-86902743	34312704	35059099	0

ssa27	35317445	LOC106589004	0 06238684	AX-87062199	35312758	35520216	0
ssa27	35317686	LOC106589004	0 06238684	AX-86946939	35312758	35520216	0
ssa27	36130314	LOC106589016	0 06536233	AX-87568804	36126304	36221007	0
ssa27	36130734	LOC106589016	0 05951687	AX-87597382	36126304	36221007	0
ssa27	36266738	LOC106589019	0 07877373	AX-87436239	36259912	36270502	0
ssa27	36274233	LOC106589019	0 07016909	AX-87733528	36259912	36270502	-3732
ssa27	36309589	LOC106589020	0 06858375	AX-87451138	36286058	36301606	-7984
ssa27	36328547	LOC106589021	0 09231298	AX-87603410	36329227	36330637	680
ssa27	36328977	LOC106589021	0 08669364	AX-87388432	36329227	36330637	250
ssa27	36330422	LOC106589021	0 05624189	AX-87045850	36329227	36330637	0
ssa27	36331547	LOC106589022	0 09231298	AX-87661571	36330885	36335577	0
ssa27	36333263	LOC106589022	0 08371247	AX-87669967	36330885	36335577	0
ssa27	36489766	LOC106589030	0 08316653	AX-87258242	36520756	36546547	30990
ssa27	36511804	LOC106589030	0 09227724	AX-87800081	36520756	36546547	8952
ssa27	36567230	LOC106589030	0 08619728	AX-87688518	36520756	36546547	-20684
ssa27	36690112	LOC106589029	0 05852361	AX-87066661	36589093	36710638	0
ssa27	36873358	LOC106589038	0 08226331	AX-87014082	36882677	36966960	9319
ssa27	36879052	LOC106589038	0 08377550	AX-87275375	36882677	36966960	3625
ssa27	36906868	LOC106589038	0 07483757	AX-87239235	36882677	36966960	0
ssa27	36907944	LOC106589038	0 05774387	AX-87015551	36882677	36966960	0
ssa27	36908879	LOC106589038	0 05997782	AX-87566005	36882677	36966960	0
ssa27	36910080	LOC106589038	0 07483757	AX-87724539	36882677	36966960	0
ssa27	36929952	LOC106589038	0 06193526	AX-86955013	36882677	36966960	0
ssa27	36940902	LOC106589038	0 07043127	AX-87401429	36882677	36966960	0
ssa27	37071726	LOC106589037	0 06456938	AX-87787892	37097056	37108078	25330
ssa27	37071848	LOC106589037	0 05945562	AX-87064551	37097056	37108078	25208
ssa27	37088575	LOC106589037	0 05591117	AX-87490636	37097056	37108078	8481
ssa27	37109231	LOC106589035	0 07382156	AX-87059161	37108641	37143960	0
ssa27	37143925	LOC106589035	0 06702944	AX-87542340	37108641	37143960	0
ssa27	37143925	LOC106589031	0 06702944	AX-87542340	37141748	37158561	0
ssa27	37244163	LOC106589032	0 05961741	AX-87268624	37187270	37197318	-46846
ssa28	5116689	LOC106589361	0 05924692	AX-87433247	5099586	5129178	0
ssa28	5117516	LOC106589361	0 05924692	AX-87499074	5099586	5129178	0
ssa28	5126563	LOC106589361	0 05924692	AX-87659423	5099586	5129178	0
ssa28	5126866	LOC106589361	0 05924692	AX-87341194	5099586	5129178	0
ssa28	5127989	LOC106589361	0 05924692	AX-87499444	5099586	5129178	0
ssa28	5304538	LOC106589362	0 07003112	AX-87727713	5259925	5313442	0
ssa28	5465030	LOC106589364	0 05917258	AX-87257768	5451888	5905337	0
ssa28	5465598	LOC106589364	0 05917258	AX-86995130	5451888	5905337	0
ssa28	5465696	LOC106589364	0 05917258	AX-87709611	5451888	5905337	0
ssa28	5465757	LOC106589364	0 06211006	AX-87809912	5451888	5905337	0
ssa28	5478458	LOC106589364	0 05917258	AX-87081505	5451888	5905337	0

ssa28	12353759	adprm	0	05739862	AX-87563830	12351375	12354008	0
ssa28	12355317	adprm	0	05739862	AX-87518538	12351375	12354008	-1310
ssa28	12362855	LOC106589196	0	06302211	AX-87034604	12363364	12373118	509
ssa28	13442645	LOC106589513	0	05929024	AX-87594782	13409934	13466964	0
ssa28	13464213	LOC106589513	0	07149932	AX-87650139	13409934	13466964	0
ssa28	13465665	LOC106589513	0	06517853	AX-87282536	13409934	13466964	0
ssa28	13873624	LOC106589526	0	06838887	AX-87108387	13866276	13885456	0
ssa28	13885513	LOC106589198	0	05572094	AX-87768229	13885197	13902349	0
ssa28	13937270	LOC106589530	0	08090127	AX-87039399	13922948	13970050	0
ssa28	14195387	trnar-ccu	0	06008075	AX-87820528	14206211	14206283	10824
ssa28	14206784	cby1	0	06008075	AX-87718009	14206347	14216598	0
ssa28	14216299	cby1	0	05949300	AX-87554844	14206347	14216598	0
ssa28	14224934	LOC106589537	0	06008075	AX-87149396	14221597	14225518	0
ssa28	14376932	LOC106589542	0	05794075	AX-86949076	14368759	14377270	0
ssa28	14431244	LOC106589547	0	05767960	AX-87006151	14426167	14443753	0
ssa28	14536856	snx29	0	05657013	AX-87196351	14535861	14645458	0
ssa28	16233864	bahcc1b	0	06549287	AX-87417778	16230967	16307890	0
ssa28	21238985	lrpprc	0	06528114	AX-87616869	21190697	21273587	0
ssa28	26314128	LOC106589707	0	05860983	AX-87183748	26067916	26326044	0
ssa28	27140444	LOC106589779	0	06860489	AX-87315907	27099151	27203824	0
ssa28	27141262	LOC106589779	0	06860489	AX-87540963	27099151	27203824	0
ssa28	27741870	mlip	0	05557050	AX-87537952	27711207	27759643	0
ssa28	28053763	LOC106589815	0	05946570	AX-87395222	28066497	28584298	12734
ssa28	28392575	LOC106589815	0	05978162	AX-87351325	28066497	28584298	0
ssa28	32399465	LOC106589896	0	05726842	AX-86985326	32365018	32400715	0
ssa29	1646552	LOC123731586	0	06015678	AX-87050128	1691999	1692053	45447
ssa29	9088480	ptprn2	0	07100451	AX-87348177	9045131	9323653	0
ssa29	9095204	ptprn2	0	07396647	AX-87423938	9045131	9323653	0
ssa29	9106151	ptprn2	0	06932156	AX-87537191	9045131	9323653	0
ssa29	9139594	ptprn2	0	07736665	AX-86914519	9045131	9323653	0
ssa29	10038907	LOC123731526	0	06615778	AX-87732184	10037083	10241181	0
ssa29	10039409	LOC123731526	0	06148715	AX-87625191	10037083	10241181	0
ssa29	10589922	LOC106590116	0	06110974	AX-87565794	10587209	10591117	0
ssa29	11484264	cacnb2a	0	05584376	AX-87561794	11397927	11509607	0
ssa29	20887731	sycp2l	0	05695289	AX-87578613	20852859	20881104	-6628
ssa29	30293596	LOC106590585	0	07635271	AX-87793866	30293423	30296013	0
ssa29	30351224	LOC106590573	0	06644740	AX-87160947	30337119	30356023	0
ssa29	30412192	LOC106590575	0	05629194	AX-87700320	30382818	30412789	0
ssa29	38569237	LOC106590673	0	06654483	AX-87199481	38557404	38642178	0
ssa29	38569983	LOC106590673	0	07128671	AX-87613342	38557404	38642178	0
ssa29	38570252	LOC106590673	0	07538300	AX-87407058	38557404	38642178	0

Table S6 Associations with run timing in 11 populations of North American Atlantic salmon identified by both partial redundancy analysis (pRDA) and latent factor mixed models (LFMM), which account for population structure using the first three PC axes that describe population structure or three latent factors, respectively.

Gene	Phenotype	Chromosome
LOC106604940	early	ssa01
LOC106605225	early	ssa01
LOC106605231	early	ssa01
LOC106605759	early	ssa01
ntm	early	ssa04
ass1	early	ssa11
LOC106581870	early	ssa21
LOC106583926	early	ssa23
LOC106583924	early	ssa23
LOC106583920	early	ssa23
LOC106583913	early	ssa23
mk03	early	ssa23
LOC106583946	early	ssa23
LOC106583966	early	ssa23
LOC106583933	early	ssa23
mdga2a	late	ssa01
LOC106570236	late	ssa01
mideasb	late	ssa01
crip1	late	ssa01
LOC106605759	late	ssa01
LOC123726801	late	ssa01
rgs7a	late	ssa01
LOC123743785	late	ssa01
LOC106612554	late	ssa01
mcph1	late	ssa01
LOC106612532	late	ssa01
LOC106565454	late	ssa01
LOC106567744	late	ssa01
LOC106567759	late	ssa01
si:dkey-220k22.1	late	ssa01
LOC106580741	late	ssa02
LOC106580670	late	ssa02
LOC106580679	late	ssa02
LOC106596732	late	ssa03
LOC106596709	late	ssa03
LOC106598927	late	ssa03

LOC106598932	late	ssa03
LOC106598933	late	ssa03
LOC106598935	late	ssa03
pif1	late	ssa03
LOC106598943	late	ssa03
LOC106598945	late	ssa03
LOC106599513	late	ssa03
LOC106600004	late	ssa03
LOC106600570	late	ssa03
trnav-uac	late	ssa03
trnav-uac	late	ssa19
trnav-uac	late	ssa29
LOC106601251	late	ssa03
LOC106606620	late	ssa03
LOC106601740	late	ssa03
LOC106606607	late	ssa03
LOC106606605	late	ssa03
LOC123741859	late	ssa03
LOC106602365	late	ssa04
LOC106602757	late	ssa04
LOC106603282	late	ssa04
LOC106603284	late	ssa04
nf1b	late	ssa04
fdx1b	late	ssa04
kpna7	late	ssa04
LOC106604063	late	ssa04
LOC100196664	late	ssa05
LOC106604745	late	ssa05
LOC106604876	late	ssa05
LOC106604822	late	ssa05
LOC106605177	late	ssa05
LOC106605120	late	ssa05
LOC106605640	late	ssa05
LOC106605668	late	ssa05
LOC106605587	late	ssa05
nphs1	late	ssa05
LOC106592621	late	ssa06
LOC106606358	late	ssa06
LOC106606423	late	ssa06
LOC106606445	late	ssa06
LOC106606408	late	ssa06
LOC106606458	late	ssa06

LOC106606457	late	ssa06
LOC106606471	late	ssa06
LOC106606982	late	ssa06
rbp	late	ssa06
LOC123743536	late	ssa06
LOC100380669	late	ssa06
LOC106601199	late	ssa06
bglap	late	ssa06
dcam	late	ssa06
cd2l6	late	ssa06
LOC106607744	late	ssa06
LOC106607909	late	ssa06
ptprk	late	ssa06
slc35f1	late	ssa06
LOC123743558	late	ssa06
fam110c	late	ssa06
LOC106608234	late	ssa06
trnat-ugu	late	ssa06
LOC106608241	late	ssa06
LOC106608246	late	ssa06
LOC106608248	late	ssa06
trnae-uuc	late	ssa06
LOC106608231	late	ssa06
LOC106608232	late	ssa06
actc1a	late	ssa06
LOC106608260	late	ssa06
LOC106608256	late	ssa06
LOC106608253	late	ssa06
sec63	late	ssa06
LOC106591735	late	ssa06
LOC106608499	late	ssa07
LOC106608492	late	ssa07
LOC106608549	late	ssa07
LOC106608564	late	ssa07
rab1ba	late	ssa07
LOC106608611	late	ssa07
LOC106608606	late	ssa07
serpinh2	late	ssa07
LOC106608915	late	ssa07
LOC106608914	late	ssa07
LOC123723867	late	ssa07
LOC106609008	late	ssa07

LOC106609004	late	ssa07
col4a5	late	ssa07
LOC106609220	late	ssa07
LOC106609461	late	ssa07
LOC106610595	late	ssa09
LOC123744595	late	ssa09
LOC106610785	late	ssa09
LOC106611017	late	ssa09
LOC106611016	late	ssa09
LOC106611015	late	ssa09
msh4	late	ssa09
LOC106611014	late	ssa09
LOC106611090	late	ssa09
LOC106610995	late	ssa09
wdr89	late	ssa09
2a5e	late	ssa09
LOC106611167	late	ssa09
LOC106611216	late	ssa09
LOC123724121	late	ssa09
calm1a	late	ssa09
brms1la	late	ssa09
LOC106611173	late	ssa09
LOC106611555	late	ssa09
LOC106612010	late	ssa09
smad5	late	ssa09
LOC106612494	late	ssa09
LOC106613290	late	ssa09
LOC106613840	late	ssa10
LOC106613839	late	ssa10
LOC106613842	late	ssa10
LOC106613847	late	ssa10
LOC106613850	late	ssa10
bcar3	late	ssa10
ptprfa	late	ssa10
LOC106560735	late	ssa10
LOC123724974	late	ssa10
LOC123724775	late	ssa10
LOC106560972	late	ssa10
vps9d1	late	ssa10
LOC106560943	late	ssa10
LOC106560868	late	ssa10
LOC106560867	late	ssa10

LOC106561543	late	ssa10
LOC100380677	late	ssa10
LOC106593086	late	ssa10
sergef	late	ssa10
phkb	late	ssa11
LOC106561994	late	ssa11
LOC106562299	late	ssa11
LOC106563532	late	ssa11
LOC106563530	late	ssa11
LOC123725251	late	ssa11
LOC106562393	late	ssa11
LOC106563040	late	ssa11
LOC106563143	late	ssa11
LOC106563138	late	ssa11
trnap-ugg	late	ssa11
LOC106563176	late	ssa11
LOC106563170	late	ssa11
LOC106563731	late	ssa11
LOC106563730	late	ssa11
LOC106563726	late	ssa11
LOC106563813	late	ssa11
LOC106563798	late	ssa11
LOC106563656	late	ssa11
LOC106563645	late	ssa11
LOC123725421	late	ssa12
LOC106565623	late	ssa12
hemk1	late	ssa12
igfn1.1	late	ssa12
lama5	late	ssa13
LOC106566616	late	ssa13
LOC106566610	late	ssa13
slc48a1a	late	ssa13
LOC106567160	late	ssa13
LOC106567057	late	ssa13
LOC106567362	late	ssa13
LOC106567368	late	ssa13
LOC106567379	late	ssa13
LOC106567426	late	ssa13
LOC106567442	late	ssa13
LOC106567543	late	ssa13
LOC106567544	late	ssa13
LOC106567545	late	ssa13

LOC106567580	late	ssa13
vps11	late	ssa13
LOC106567637	late	ssa13
zpr1	late	ssa13
bsx	late	ssa13
lim2.1	late	ssa13
LOC106567659	late	ssa13
LOC106567647	late	ssa13
tmem218	late	ssa13
LOC106567652	late	ssa13
LOC106568105	late	ssa13
bmp1	late	ssa13
LOC106568226	late	ssa13
LOC106568207	late	ssa13
LOC106568220	late	ssa13
foxred1	late	ssa13
LOC106568196	late	ssa13
LOC106568532	late	ssa13
LOC106568538	late	ssa13
LOC106569075	late	ssa14
LOC106570301	late	ssa14
LOC106570298	late	ssa14
LOC106570290	late	ssa14
LOC106570311	late	ssa14
LOC106571107	late	ssa15
LOC123727248	late	ssa15
LOC106571120	late	ssa15
LOC106571124	late	ssa15
nt5d1	late	ssa15
LOC106571321	late	ssa15
marcs	late	ssa15
LOC106571347	late	ssa15
cdk19	late	ssa15
LOC106571924	late	ssa15
LOC106572392	late	ssa15
LOC106572671	late	ssa15
LOC106572669	late	ssa15
LOC106573061	late	ssa16
LOC106573083	late	ssa16
LOC106573156	late	ssa16
LOC106574307	late	ssa16
LOC106574300	late	ssa16

LOC106574298	late	ssa16
si:ch211-246m6.5	late	ssa16
aox5	late	ssa16
ahr2d	late	ssa16
LOC106574285	late	ssa16
ftcd	late	ssa16
LOC106574281	late	ssa16
LOC106574280	late	ssa16
LOC106574279	late	ssa16
LOC106575116	late	ssa17
plcc	late	ssa17
LOC106575234	late	ssa17
LOC106575378	late	ssa17
LOC106575379	late	ssa17
LOC106576215	late	ssa17
LOC106576214	late	ssa17
LOC106609571	late	ssa17
LOC106576572	late	ssa17
LOC106576768	late	ssa18
tisd	late	ssa18
LOC106576956	late	ssa18
LOC106576957	late	ssa18
LOC106576958	late	ssa18
LOC106577144	late	ssa18
LOC106577124	late	ssa18
LOC106577388	late	ssa18
LOC106577538	late	ssa18
LOC106577539	late	ssa18
LOC106577599	late	ssa18
LOC106578155	late	ssa18
LOC123729044	late	ssa19
LOC106578273	late	ssa19
pfkpa	late	ssa19
fars2	late	ssa19
slf1	late	ssa20
LOC106580145	late	ssa20
LOC106579912	late	ssa20
LOC123729170	late	ssa20
cbl	late	ssa20
LOC123729175	late	ssa20
LOC123729233	late	ssa20
LOC123729176	late	ssa20

LOC123729177	late	ssa20
LOC123729244	late	ssa20
LOC123729245	late	ssa20
calm3a	late	ssa20
LOC123729248	late	ssa20
LOC106580770	late	ssa20
spit2	late	ssa20
LOC100136491	late	ssa20
kcnj5	late	ssa20
LOC106581092	late	ssa20
b3gat1a	late	ssa20
siae	late	ssa20
LOC106581222	late	ssa20
LOC106581220	late	ssa20
LOC106581238	late	ssa20
LOC106581239	late	ssa20
LOC106581247	late	ssa20
LOC106581700	late	ssa21
LOC123729587	late	ssa21
LOC106581718	late	ssa21
LOC106581870	late	ssa21
hdac4	late	ssa21
LOC106581814	late	ssa21
LOC106581809	late	ssa21
LOC106581710	late	ssa21
kdm6a	late	ssa21
ddx3xa	late	ssa21
LOC106582286	late	ssa21
LOC100194642	late	ssa21
slc49a4	late	ssa21
LOC106582705	late	ssa22
LOC106583215	late	ssa22
LOC106583214	late	ssa22
eefsec	late	ssa22
LOC106583929	late	ssa23
LOC106583924	late	ssa23
LOC106583946	late	ssa23
LOC106583948	late	ssa23
LOC106584350	late	ssa23
dnaja	late	ssa23
gcnt3	late	ssa23
LOC106584844	late	ssa23

LOC106584981	late	ssa24
LOC106585508	late	ssa24
hibch	late	ssa25
LOC106586201	late	ssa25
LOC106586202	late	ssa25
LOC106586079	late	ssa25
LOC106586205	late	ssa25
LOC106586206	late	ssa25
LOC106586282	late	ssa25
LOC106586083	late	ssa25
sh3b4	late	ssa25
arl4c	late	ssa25
arhgap15	late	ssa25
map2	late	ssa25
ak8	late	ssa26
LOC106587216	late	ssa26
LOC106587212	late	ssa26
tbb3	late	ssa26
ext1b	late	ssa27
LOC106588469	late	ssa27
coea1	late	ssa27
tpd52	late	ssa27
LOC106588858	late	ssa27
LOC123730919	late	ssa27
LOC106589273	late	ssa28
LOC106589308	late	ssa28
LOC106589429	late	ssa28
LOC106589815	late	ssa28
LOC106590042	late	ssa29
LOC106590040	late	ssa29
LOC106590155	late	ssa29
LOC106590156	late	ssa29
LOC106590287	late	ssa29
LOC106590681	late	ssa29
pgcp	modality	ssa02
ccnd2	modality	ssa07
LOC106570311	modality	ssa14

Table S7 Function enrichment of genes associated with run timing (early, late and modality) in 11 populations of North American Atlantic salmon.

Background	Database	Phenotype	Outlier method	Enrichment FDR	Fold Enrichment	Pathway	Genes
Limited: 220K	KEGG	Modality	RDA	0.04	5.80	Carbon metabolism	ECHS1 PGAM1 LOC106564857 SHMT2 LOC106580202 LOC106602756
Default: Ssal_v3.1	KEGG	Early	pRDA	0.02	11.76	Peroxisome	LOC106573701 LOC106574138 LOC106578486
Default: Ssal_v3.1	KEGG	Early	pRDA	0.01	9.92	Gap junction	MK03 LOC106562384 HTR2B LOC106602731
Default: Ssal_v3.1	KEGG	Early	pRDA	0.01	9.68	Adherens junction	MK03 LOC106578541 LOC106584181 LOC106589900
Default: Ssal_v3.1	KEGG	Early	pRDA	0.01	9.33	Oocyte meiosis	MK03 LOC106562384 LOC106589820 LOC106602935
Default: Ssal_v3.1	KEGG	Early	pRDA	0.04	8.40	Progesterone-mediated oocyte maturation	MK03 LOC106562384 LOC106589820
Default: Ssal_v3.1	KEGG	Early	pRDA	0.00	8.28	Adrenergic signaling in cardiomyocytes	MK03 LOC106562384 LOC106563219 LOC106572513 LOC106583902 LOC106602935
Default: Ssal_v3.1	KEGG	Early	pRDA	0.04	7.54	ErbB signaling pathway	MK03 LOC106584354 LOC106602935
Default: Ssal_v3.1	KEGG	Early	pRDA	0.05	7.17	Melanogenesis	MK03 LOC106562384 LOC106602935
Default: Ssal_v3.1	KEGG	Early	pRDA	0.05	7.00	GnRH signaling pathway	MK03 LOC106562384 LOC106602935
Default: Ssal_v3.1	KEGG	Early	pRDA	0.03	5.94	Cell adhesion molecules	LOC106575199 LOC106581720 LOC106584181 LOC106584306
Default: Ssal_v3.1	KEGG	Early	pRDA	0.01	5.44	Calcium signaling pathway	LOC106562384 HTR2B LOC106574152 LOC106583902 LOC106602935 LOC106604681
Default: Ssal_v3.1	KEGG	Early	pRDA	0.00	5.30	Neuroactive ligand-receptor interaction	LOC106561032 HTR2B LOC106574152 LOC106575271 LOC106578541 LOC106586649 LOC106589304 LOC106604681
Default: Ssal_v3.1	KEGG	Early	pRDA	0.03	4.00	MAPK signaling pathway	TNFL6 MK03 LOC106561692 LOC106583946 LOC106584354 LOC106589820

Default: Ssal_v3.1	Biological processes	Early	pRDA	0.05	1.84	Cell communication	SPRED1 LOC106574143 LOC106602731 LOC106583180 LOC106589820 LOC106602845 LOC106563207 LOC106602818 LOC106606440 LOC106567541 ETBR2 LOC106583226 LOC106575271 LOC106568006 LOC106584283 LOC106606050 LOC106586649 LOC106572513 LOC106606127 LOC106580556 LOC106584306 LOC106562384 LOC106561616 LOC106587577 LOC106567964 SYK LOC106574152 LOC106561032 HTR2B LOC106585561 LOC106568179 LOC106565372 LOC106585546 LOC106582440 LOC106563326 LOC106570566 LOC106567065 RCAN3 LOC106610964 GJA3
Default: Ssal_v3.1	Biological processes	Early	pRDA	0.05	1.83	Signaling	SPRED1 LOC106574143 LOC106602731 LOC106583180 LOC106589820 LOC106602845 LOC106563207 LOC106602818 LOC106606440 LOC106567541 ETBR2 LOC106583226 LOC106575271 LOC106568006 LOC106584283 LOC106606050 LOC106586649 LOC106572513 LOC106606127 LOC106580556 LOC106584306 LOC106562384 LOC106561616 LOC106587577 LOC106567964 SYK LOC106574152 LOC106561032 HTR2B LOC106585561 LOC106568179 LOC106565372 LOC106585546 LOC106582440 LOC106563326 LOC106570566 LOC106567065 RCAN3 LOC106610964
Default: Ssal_v3.1	KEGG	Late	pRDA	0.05	10.91	Glycerolipid metabolism	PLCC LOC106582334
Default: Ssal_v3.1	KEGG	Late	pRDA	0.04	10.05	Glycerophospholipid metabolism	PLCC LOC106582334 LOC106583972
Default: Ssal_v3.1	KEGG	Late	pRDA	0.05	6.59	Apelin signaling pathway	MK03 HDAC4 LOC106583918 LOC106605316
Default: Ssal_v3.1	KEGG	Late	pRDA	0.05	6.39	Cell adhesion molecules	CLDX LOC106563813 LOC106573083 LOC106584181
Default: Ssal_v3.1	KEGG	Late	pRDA	0.05	5.94	Adrenergic signaling in cardiomyocytes	2A5E MK03 LOC106573547 LOC106589429

Default: Ssal_v3.1	KEGG	Late	pRDA	0.05	2.52	Metabolic pathways	LOC106571323 LOC106574285 PLCC LOC106575089 LOC106586568 LOC106582334 LOC106583904 LOC106583972 LOC106584037 LOC106585720 LOC106586201 AK8
Default: Ssal_v3.1	Biological processes	Modality	pRDA	0.05	34.98	Mo-molybdopterin cofactor metabolic proc.	LOC106610594 LOC106571666
Default: Ssal_v3.1	Biological processes	Modality	pRDA	0.05	34.98	Molybdopterin cofactor metabolic proc.	LOC106610594 LOC106571666
Default: Ssal_v3.1	Biological processes	Modality	pRDA	0.05	34.98	Prosthetic group metabolic proc.	LOC106610594 LOC106571666
Default: Ssal_v3.1	Biological processes	Modality	pRDA	0.05	34.98	Mo-molybdopterin cofactor biosynthetic proc.	LOC106610594 LOC106571666
Default: Ssal_v3.1	Biological processes	Modality	pRDA	0.02	1.99	Organic cyclic compound biosynthetic proc.	LOC106610594 LOC106568846 LOC106603444 LOC106568843 LOC106607468 EMX2 TP73 LOC106588963 IMDH1 KPRA LOC106588610 LOC106606805 LOC106588852 POLR1B LOC106571799 LOC106600304 HOXD1AA LOC106589004 LOC106605519 LOC106571666 LOC106604248 LOC106568088 LOC106598907 LOC106598913 LOC106571644 TPK1 LOC106582356 LOC106613630 LOC106593878 LOC106584428 LOC106563176 LOC106582442

Default: Ssal_v3.1	Biological processes	Modality	pRDA	0.00	1.98	Cellular biosynthetic proc.	LOC106610594 LOC106568846 LOC106603444 LOC106568843 LOC106574022 LOC106607468 EMX2 LOC106589173 TP73 LOC106588963 IMDH1 KPRA LOC106566938 LOC106588610 LOC106606805 DDAH1 LOC106565027 LOC106588852 POLR1B LOC106571799 LOC106588994 LOC106600304 HOXD1AA LOC106589004 LOC106605519 LOC106571666 LOC106598945 LOC106579029 LOC106568088 LOC106571665 LOC106598907 LOC106598913 LOC106571644 TPK1 LOC106582356 LOC106613630 LOC106612828 LOC106593878 LOC106576502 LOC106584428 LOC106563176 LOC106582442 LOC106568328
Default: Ssal_v3.1	Biological processes	Modality	pRDA	0.00	1.98	Biosynthetic proc.	LOC106610594 LOC106568846 LOC106603444 LOC106568843 LOC106574022 LOC106607468 EMX2 LOC106589173 TP73 LOC106588963 IMDH1 KPRA LOC106566938 LOC106588610 LOC106606805 DDAH1 LOC106565027 LOC106588852 POLR1B LOC106571799 LOC106588994 LOC106600304 HOXD1AA LOC106589004 LOC106605519 LOC106571666 LOC106604248 LOC106598945 LOC106579029 LOC106568088 LOC106571433 LOC106571665 LOC106598907 LOC106598913 LOC106571644 TPK1 LOC106582356 LOC106613630 LOC106612828 LOC106593878 LOC106576502 LOC106584428 LOC106563176 LOC106582442 LOC106568328

Default: Ssal_v3.1	Biological processes	Modality	pRDA	0.03	1.97	Reg. of cellular metabolic proc.	LOC106568846 LOC106603444 LOC106568843 LOC106607468 EMX2 TP73 LOC106588963 LOC106574038 LOC106588610 DDAH1 LOC106588852 LOC106571799 LOC106568835 LOC106600304 HOXD1AA LOC106589004 LOC106605519 LOC106568088 LOC106598907 LOC106598913 LOC106571644 LOC106582356 LOC106613630 LOC106606431 LOC106593878 LOC106576502 LOC106584428 LOC106563176 LOC106582442
Default: Ssal_v3.1	Biological processes	Modality	pRDA	0.00	1.97	Organic substance biosynthetic proc.	LOC106610594 LOC106568846 LOC106603444 LOC106568843 LOC106574022 LOC106607468 EMX2 LOC106589173 TP73 LOC106588963 IMDH1 KPRA LOC106566938 LOC106588610 LOC106606805 LOC106565027 LOC106588852 POLR1B LOC106571799 LOC106588994 LOC106600304 HOXD1AA LOC106589004 LOC106605519 LOC106571666 LOC106604248 LOC106598945 LOC106579029 LOC106568088 LOC106571433 LOC106571665 LOC106598907 LOC106598913 LOC106571644 TPK1 LOC106582356 LOC106613630 LOC106612828 LOC106593878 LOC106576502 LOC106584428 LOC106563176 LOC106582442 LOC106568328
Default: Ssal_v3.1	Biological processes	Modality	pRDA	0.03	1.96	Heterocycle biosynthetic proc.	LOC106610594 LOC106568846 LOC106603444 LOC106568843 LOC106607468 EMX2 TP73 LOC106588963 IMDH1 KPRA LOC106588610 LOC106606805 LOC106588852 POLR1B LOC106571799 LOC106600304 HOXD1AA LOC106589004 LOC106605519 LOC106571666 LOC106568088 LOC106598907 LOC106598913 LOC106571644 TPK1 LOC106582356 LOC106613630 LOC106593878 LOC106584428 LOC106563176 LOC106582442

Default: Ssal_v3.1	Biological processes	Modality	pRDA	0.04	1.95	Reg. of cellular biosynthetic proc.	LOC106568846 LOC106603444 LOC106568843 LOC106607468 EMX2 TP73 LOC106588963 LOC106588610 DDAH1 LOC106588852 LOC106571799 LOC106600304 HOXD1AA LOC106589004 LOC106605519 LOC106568088 LOC106598907 LOC106598913 LOC106571644 LOC106582356 LOC106613630 LOC106593878 LOC106576502 LOC106584428 LOC106563176 LOC106582442
Default: Ssal_v3.1	Biological processes	Modality	pRDA	0.05	1.90	Reg. of nucleic acid-templated transcription	LOC106568846 LOC106603444 LOC106568843 LOC106607468 EMX2 TP73 LOC106588963 LOC106588610 LOC106588852 LOC106571799 LOC106600304 HOXD1AA LOC106589004 LOC106605519 LOC106568088 LOC106598907 LOC106598913 LOC106571644 LOC106582356 LOC106613630 LOC106593878 LOC106584428 LOC106563176 LOC106582442
Default: Ssal_v3.1	Biological processes	Modality	pRDA	0.04	1.89	Reg. of nitrogen compound metabolic proc.	LOC106568846 LOC106603444 LOC106568843 LOC106607468 EMX2 TP73 LOC106588963 LOC106588610 DDAH1 LOC106588852 LOC106571799 LOC106568835 LOC106600304 HOXD1AA LOC106589004 LOC106605519 LOC106568088 LOC106598907 LOC106598913 LOC106571644 LOC106582356 LOC106613630 LOC106606431 LOC106593878 LOC106584428 LOC106563176 LOC106582442
Default: Ssal_v3.1	Biological processes	Modality	pRDA	0.04	1.88	Reg. of primary metabolic proc.	LOC106568846 LOC106603444 LOC106568843 LOC106607468 EMX2 TP73 LOC106588963 LOC106588610 LOC106588852 LOC106571799 LOC106568835 LOC106600304 HOXD1AA LOC106589004 LOC106605519 LOC106568088 LOC106598907 LOC106598913 LOC106571644 LOC106582356 LOC106613630 LOC106606431 LOC106593878 LOC106576502 LOC106584428 LOC106563176 LOC106582442

Default: Ssal_v3.1	Biological processes	Modality	pRDA	0.05	1.85	Aromatic compound biosynthetic proc.	LOC106568846 LOC106603444 LOC106568843 LOC106607468 EMX2 TP73 LOC106588963 IMDH1 KPRA LOC106588610 LOC106606805 LOC106588852 POLR1B LOC106571799 LOC106600304 HOXD1AA LOC106589004 LOC106605519 LOC106568088 LOC106598907 LOC106598913 LOC106571644 TPK1 LOC106582356 LOC106613630 LOC106593878 LOC106584428 LOC106563176 LOC106582442
Default: Ssal_v3.1	Biological processes	Modality	pRDA	0.05	1.82	Nucleobase-containing compound biosynthetic proc.	LOC106568846 LOC106603444 LOC106568843 LOC106607468 EMX2 TP73 LOC106588963 IMDH1 KPRA LOC106588610 LOC106606805 LOC106588852 POLR1B LOC106571799 LOC106600304 HOXD1AA LOC106589004 LOC106605519 LOC106568088 LOC106598907 LOC106598913 LOC106571644 LOC106582356 LOC106613630 LOC106593878 LOC106584428 LOC106563176 LOC106582442
Default: Ssal_v3.1	Biological processes	Modality	pRDA	0.04	1.81	Macromolecule biosynthetic proc.	LOC106568846 LOC106603444 LOC106568843 LOC106574022 LOC106607468 EMX2 LOC106589173 TP73 LOC106588963 LOC106588610 LOC106565027 LOC106588852 POLR1B LOC106571799 LOC106588994 LOC106600304 HOXD1AA LOC106589004 LOC106605519 LOC106598945 LOC106568088 LOC106571665 LOC106598907 LOC106598913 LOC106571644 LOC106582356 LOC106613630 LOC106612828 LOC106593878 LOC106584428 LOC106563176 LOC106582442

Default: Ssal_v3.1	Biological processes	Modality	pRDA	0.05	1.73	Cellular nitrogen compound biosynthetic proc.	LOC106568846 LOC106603444 LOC106568843 LOC106607468 EMX2 LOC106589173 TP73 LOC106588963 IMDH1 KPRA LOC106588610 LOC106606805 DDAH1 LOC106588852 POLR1B LOC106571799 LOC106588994 LOC106600304 HOXD1AA LOC106589004 LOC106605519 LOC106568088 LOC106598907 LOC106598913 LOC106571644 TPK1 LOC106582356 LOC106613630 LOC106593878 LOC106584428 LOC106563176 LOC106582442

Table S8 Diversity metrics of loci associated with late run timing (without population structure correction) in 11 populations of North American Atlantic salmon.

	Site Code	Region	River	Latitude	Longitude	Fis	Fst	n	Ho	He	n_loci	π per SNP
**Late run timing loci**	CMP	Newfoundland	Campbellton	49 27	-54 93	0 03	0 51	25	0 21	0 21	1160	0 18
	CNR	Newfoundland	Conne	47 92	-55 68	0 00	0 60	30	0 17	0 17	1216	0 15
	ENG	Labrador	English	54 97	-59 75	-0 01	0 47	27	0 26	0 25	1053	0 19
	MSW	Maritimes	Southwest Miramichi	46 88	-65 66	-0 01	-0 23	23	0 46	0 44	1413	0 45
	MUN	Maritimes	Northwest Miramichi	46 94	-65 78	-0 01	-0 22	24	0 45	0 44	1413	0 45
	NPR	Newfoundland	Northeast (Placentia)	47 29	-53 79	0 01	0 37	30	0 25	0 25	1297	0 23
	NSH	Maritimes	Nashwaak	46 12	-66 61	-0 03	-0 19	44	0 45	0 43	1413	0 44
	SH	Labrador	Sand Hill	53 56	-56 37	0 02	0 39	19	0 27	0 27	1142	0 22
	TNR	Newfoundland	Terra Nova	48 55	-54 18	0 00	0 47	29	0 22	0 22	1221	0 19
	UPS	Maritimes	Upsalquitch	47 57	-66 54	0 01	-0 17	28	0 43	0 42	1413	0 43
	WAB	Newfoundland	Western Arm Brook	51 19	-56 77	0 00	0 39	18	0 26	0 26	1201	0 22

n, number of individuals; Ho, mean observed heterozygosity; He, mean expected heterozygosity; π per SNP, average nucleotide diversity per SNP

Table S9 Diversity metrics of loci associated with modality (without population structure correction) in 11 populations of North American Atlantic salmon.

	Site Code	Region	River	Latitude	Longitude	Fis	Fst	n	Ho	He	n_loci	π per SNP
**Modality**	CMP	Newfoundland	Campbellton	49 27	-54 93	0 01	0 54	25	####	####	1136	0 16
	CNR	Newfoundland	Conne	47 92	-55 68	0 00	0 52	30	####	####	1237	0 17
	ENG	Labrador	English	54 97	-59 75	0 02	0 60	27	####	####	946	0 14
	MSW	Maritimes	Southwest Miramichi	46 88	-65 66	0 00	####	23	####	####	1413	0 45
	MUN	Maritimes	Northwest Miramichi	46 94	-65 78	####	####	24	####	####	1413	0 44
	NPR	Newfoundland	Northeast (Placentia)	47 29	-53 79	0 00	0 36	30	####	####	1283	0 23
	NSH	Maritimes	Nashwaak	46 12	-66 61	####	####	44	####	####	1413	0 43
	SH	Labrador	Sand Hill	53 56	-56 37	0 01	0 46	19	####	####	1076	0 19
	TNR	Newfoundland	Terra Nova	48 55	-54 18	0 01	0 49	29	####	####	1183	0 18
	UPS	Maritimes	Upsalquitch	47 57	-66 54	0 01	####	28	####	####	1413	0 43
	WAB	Newfoundland	Western Arm Brook	51 19	-56 77	####	0 40	18	####	####	1198	0 22

n, number of individuals; Ho, mean observed heterozygosity; He, mean expected heterozygosity; π per SNP, average nucleotide diversity per SNP

Table S10 Diversity metrics of population structure corrected loci associated with late run timin
in 11 populations of North American Atlantic salmon.

	Site Code	Region	River	Latitude	Longitude	Fis	Fst	n	Ho	He	n_loci	π per SNP
Late run timing loci	CMP	Newfoundland	Campbellton	49 27	-54 93	-0 04	0 49	25	0 15	0 14	1033	0 10
	CNR	Newfoundland	Conne	47 92	-55 68	-0 02	-0 60	30	0 36	0 34	1288	0 32
	ENG	Labrador	English	54 97	-59 75	-0 08	0 42	27	0 25	0 23	718	0 12
	MSW	Maritimes	Southwest Miramichi	46 88	-65 66	-0 02	0 54	23	0 21	0 20	627	0 09
	MUN	Maritimes	Northwest Miramichi	46 94	-65 78	-0 04	0 55	24	0 21	0 20	624	0 09
	NPR	Newfoundland	Northeast (Placentia)	47 29	-53 79	-0 05	-0 63	30	0 38	0 35	1287	0 33
	NSH	Maritimes	Nashwaak	46 12	-66 61	-0 01	0 54	44	0 17	0 16	787	0 09
	SH	Labrador	Sand Hill	53 56	-56 37	-0 03	0 00	19	0 27	0 25	1089	0 20
	TNR	Newfoundland	Terra Nova	48 55	-54 18	0 03	0 46	29	0 14	0 14	1095	0 11
	UPS	Maritimes	Upsalquitch	47 57	-66 54	-0 02	0 14	28	0 23	0 22	1062	0 17
	WAB	Newfoundland	Western Arm Brook	51 19	-56 77	0 00	0 18	18	0 28	0 27	839	0 16

n, number of individuals; Ho, mean observed heterozygosity; He, mean expected heterozygosity; π per SNP, average nucleotide diversity per SNP

Table S11 Diversity metrics of population structure corrected loci associated with modality in 11 populations of North American Atlantic salmon.

	Site Code	Region	River	Latitude	Longitude	Fis	Fst	n	Ho	He	n_loci	π per SNP
**Modality loci**	CMP	Newfoundland	Campbellton	49 27	-54 93	-0 01	0 43	25	0 16	0 16	862	0 10
	CNR	Newfoundland	Conne	47 92	-55 68	0 01	0 30	30	0 18	0 18	960	0 12
	ENG	Labrador	English	54 97	-59 75	0 05	0 13	27	0 21	0 22	943	0 15
	MSW	Maritimes	Southwest Miramichi	46 88	-65 66	0 00	0 48	23	0 17	0 17	740	0 09
	MUN	Maritimes	Northwest Miramichi	46 94	-65 78	0 04	0 47	24	0 17	0 18	721	0 09
	NPR	Newfoundland	Northeast (Placentia)	47 29	-53 79	0 00	0 23	30	0 17	0 17	1103	0 13
	NSH	Maritimes	Nashwaak	46 12	-66 61	0 00	0 55	44	0 13	0 13	837	0 08
	SH	Labrador	Sand Hill	53 56	-56 37	-0 02	-0 49	19	0 30	0 29	1234	0 26
	TNR	Newfoundland	Terra Nova	48 55	-54 18	0 00	0 42	29	0 15	0 15	951	0 10
	UPS	Maritimes	Upsalquitch	47 57	-66 54	0 01	0 43	28	0 16	0 16	863	0 10
	WAB	Newfoundland	Western Arm Brook	51 19	-56 77	-0 04	-1 21	18	0 41	0 38	1385	0 38

n, number of individuals; Ho, mean observed heterozygosity; He, mean expected heterozygosity; π per SNP, average nucleotide diversity per SNP
